# Supplementary material for: Cordycepin Triphosphate as a Potential Modulator of Cellular Plasticity in Cancer via cAMP-Dependent Pathways: An In Silico Approach
Source: Int J Mol Sci. 2024 May 23;25(11):5692. doi: 10.3390/ijms25115692 (PMC11171877; doi:10.3390/ijms25115692)
Supplement: Supplementary file 1 [file ijms-25-05692-s001.zip › Supplementary material (File S1).pdf]

Cordycepin triphosphate as a potential modulator of cellular plasticity in cancer via cAMP-dependent pathways: an *in silico* approach.

Jose Luis Gonzalez-Llerena [1,2], Bryan Alejandro Espinosa-Rodriguez [1], Daniela Treviño-Almaguer [1], Patricia Gonzalez-Barranco [1], Luis Fernando Mendez-Lopez [2], Pilar Carranza-Rosales [3], Nancy Elena Guzman-Delgado [4], Antonio Romo-Mancillas [5,*] and Isaias Balderas-Renteria [1,*]

Authors affiliation:

[1]Laboratory of Molecular Pharmacology and Biological Models, School of Chemistry, Autonomous University of Nuevo Leon, San Nicolas de los Garza 66451, Mexico; jose.gonzalezll@uanl.edu.mx (J.L.G.-L.); bryan.espinosardr@uanl.edu.mx (B.A.E.-R.); daniela.trevinoal@uanl.edu.mx (D.T.-A.); patricia.gonzalezbrn@uanl.edu.mx (P.G.-B.)

[2]Center for Research on Nutrition and Public Health, School of Public Health and Nutrition, Autonomous University of Nuevo Leon, Monterrey 66460, Mexico; jose.gonzalezll@uanl.edu.mx (J.L.G.-L.); luis.mendezlop@uanl.edu.mx (L.F.M.-L.)

[3]Laboratory of Cell Biology, Northeast Biomedical Research Center. Mexican Social Security Institute, Monterrey 64720, Mexico; carranza60@yahoo.com.mx

[4]Health Research Division, High Specialty Medical Unit, Cardiology Hospital N. 34. Mexican Social Security Institute, Monterrey 64360, Mexico; nancyegd@gmail.com

[5]Computer Aided Drug Design and Synthesis Group, School of Chemistry, Autonomous University of Queretaro, Queretaro 76010, Mexico

**Correspondence:*

1. Isaias Balderas Renteria

School of Chemistry, Autonomous University of Nuevo Leon, 66451, San Nicolas de los Garza, Nuevo Leon, 66451, Mexico.

E-mail: isaias.balderasrn@uanl.edu.mx

Telephone number: +52 81 1033 6764

2. Antonio Romo Mancillas

Computer Aided Drug Design and Synthesis Group, School of Chemistry, Autonomous University of Queretaro, Queretaro 76010, Mexico.

E-mail: ruben.romo@uaq.mx

Telephone number: +52 442-1921200 ext. 75032

Complementary results of the structural comparison at 2D and 3D levels

The results not shown in the main text regarding the 2D, and 3D comparisons are shown in the Supplementary material part B, where the TC, ST, and Combo T scores obtained in the comparison between cordycepin and its derivatives against adenosine and its derivatives can be observed.

Complementary results of physicochemical parameters

The physicochemical parameters obtained for all the molecules such as topological polar surface area (TPSA), molecular weight, consensus Log P, sp3 carbon fraction, hydrogen bond donors and acceptors, and rotational bonds are shown in supplementary material part B.

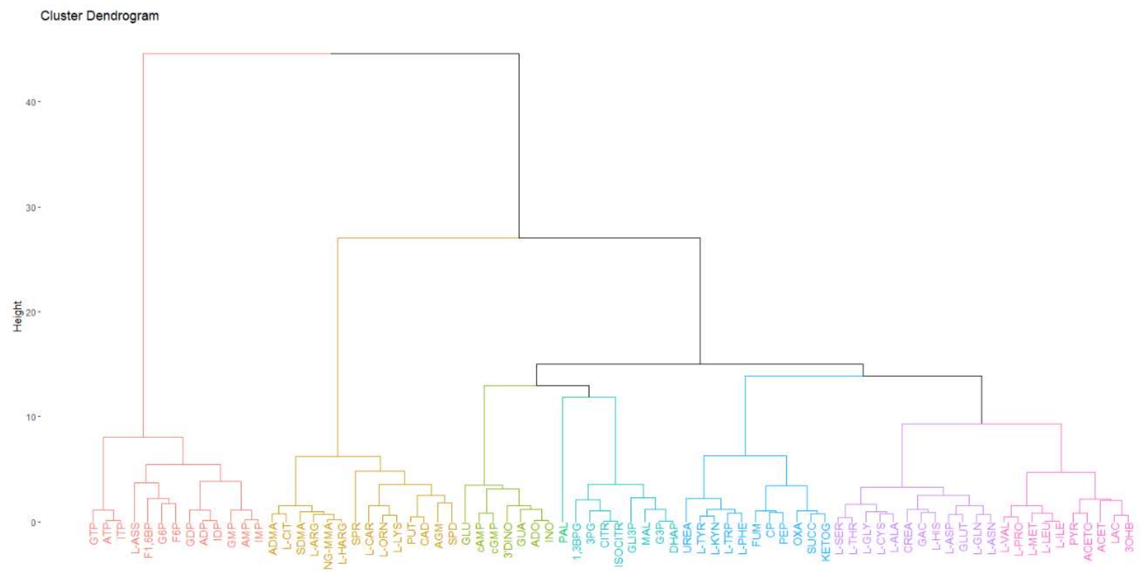

Figure S1. Hierarchical clustering of 3’-dINO. From left to right the percentual distribution is as follows: cluster red (17.11%); cluster yellow (18.42%); cluster light green (9.21%); cluster dark green (1.32%); cluster cyan 8 (10.52%); cluster sky blue (14.47); cluster purple (15.79%); cluster pink (13.16%).

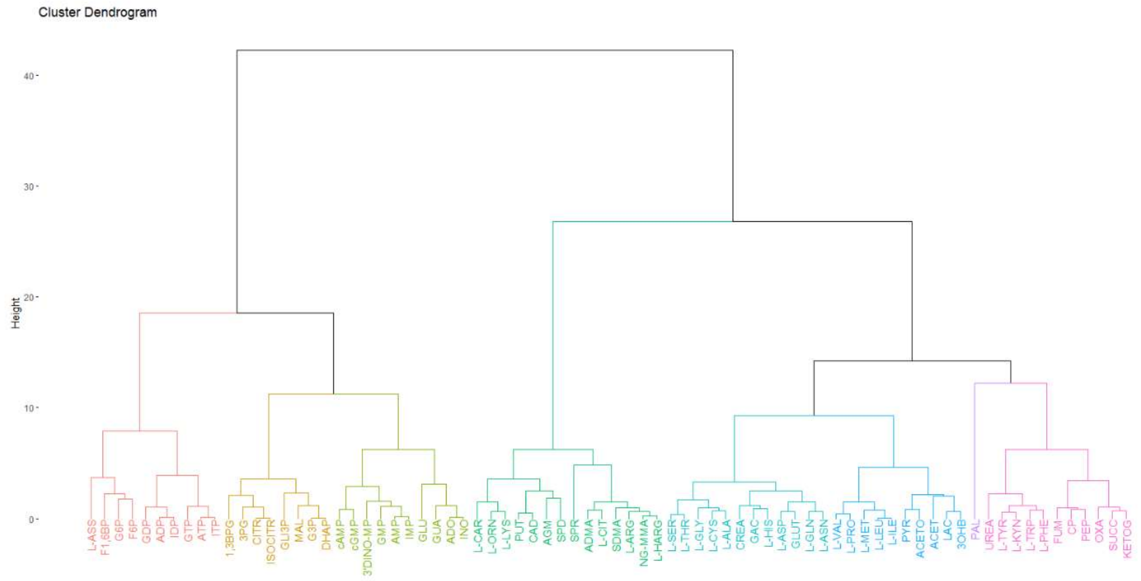

Figure S2. Hierarchical clustering of 3'-dIMP. From left to right the percentual distribution is as follows: cluster red (13.16%); cluster yellow (10.53%); cluster light green (13.16%); cluster dark green (18.42%); cluster cyan (15.97%); cluster sky blue (13.15%); cluster purple (1.32%); cluster pink (14.47%).

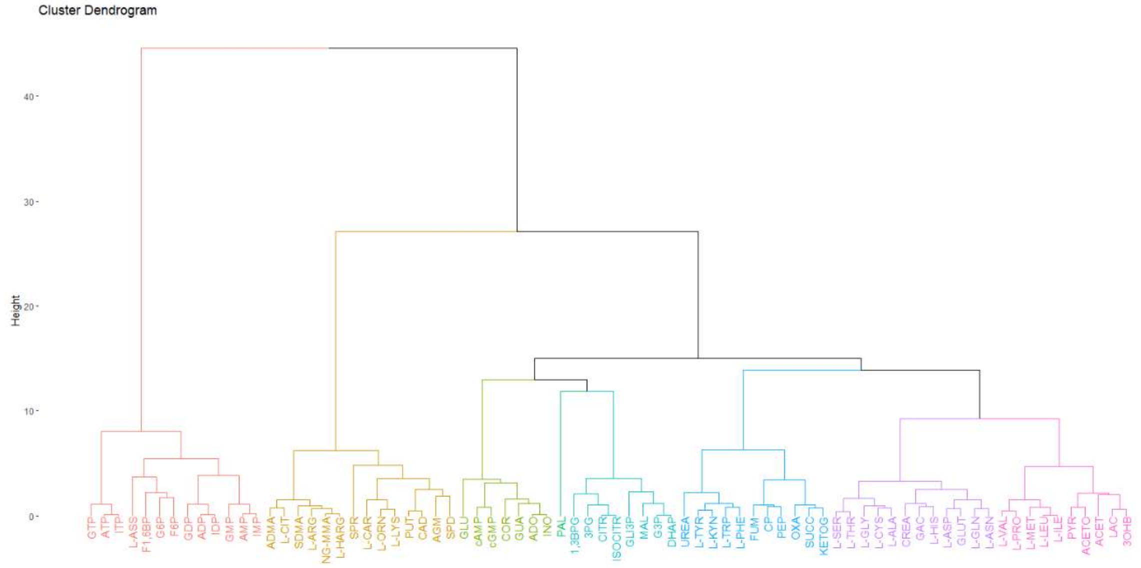

Figure S3. Hierarchical clustering of COR. From left to right the percentual distribution is as follows: cluster red (17.1%); cluster yellow (18.42%); cluster light green (9.21%); cluster dark green (1.32%); cluster cyan (10.52%); cluster sky blue (14.47%); cluster purple (15.79%); cluster pink (13.16%).

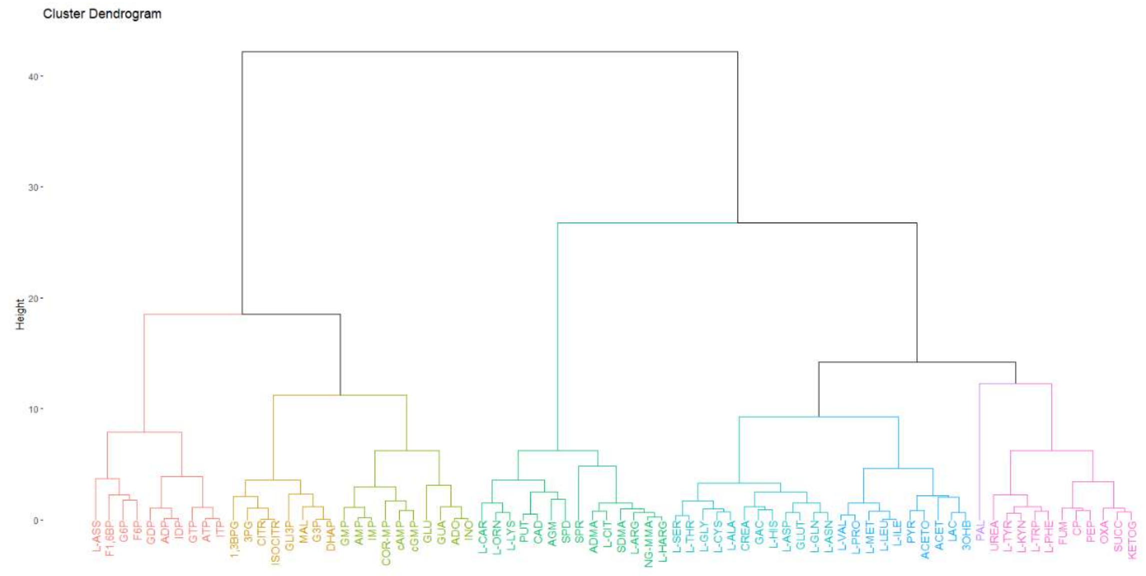

Figure S4. Hierarchical clustering of COR-MP. From left to right the percentual distribution is as follows: cluster red (13.16%); cluster yellow (10.53%); cluster light green (13.16%); cluster dark green (18.42%); cluster cyan (15.79%); cluster sky blue (13.16%); cluster purple (1.32%); cluster pink (14.47%).

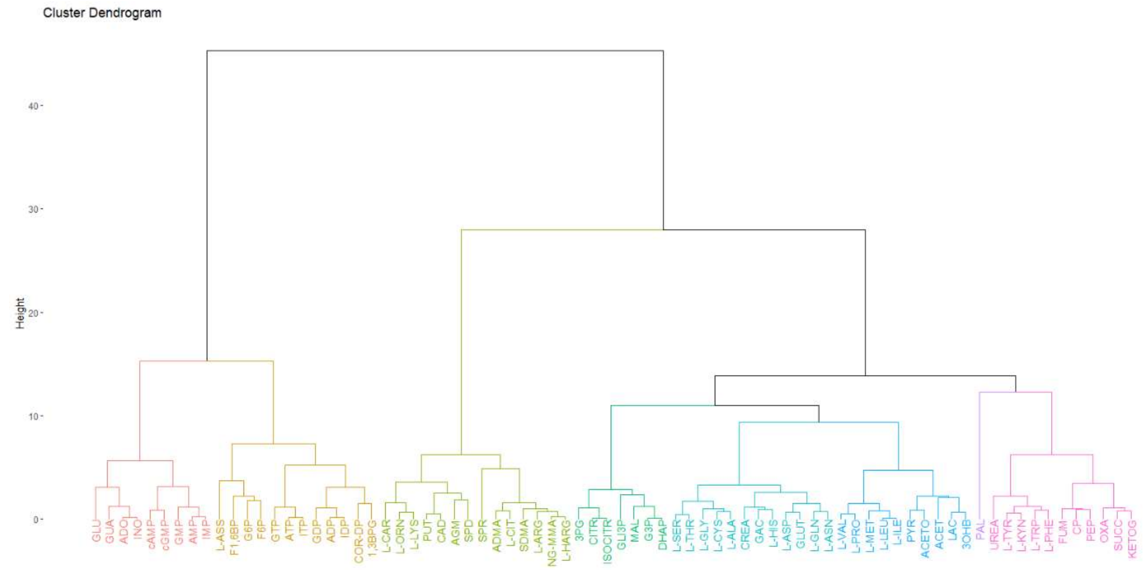

Figure S5. Hierarchical clustering of COR-DP. From left to right the percentual distribution is as follows: cluster red (11.84%); cluster yellow (15.79%); cluster light green (18.42%); cluster dark green (9.21%); cluster cyan (15.79%); cluster sky blue (13.16%); cluster purple (1.32%); cluster pink (14.47%).

Target prediction for molecular docking:

Possible metabolite targets were predicted using the SwissTargetPrediction tool (See Supplementary material part B). According to the SwissTargetPrediction tool cordycepin and 3'deoxyinosine do not have similar actives found and no targets were predicted. We complement this prediction using the SEA Search Server (See Supplementary material part B).

Complementary molecular docking analysis methodology and results:

After obtaining possible cordycepin targets and derivatives using the SwissTargetPrediction tool (Shown in the Supplementary material part B), sought to evaluate the similarity regarding energy affinity and binding modes between some targets whose substrates belong to the metabolism of purine compounds derived from adenosine (SAM, INO, ADO, AMP, ADP, and ATP) with their respective exogenous analogs derived from COR (3'-dSAM, 3'-dINO, COR, COR-MP, COR-DP and COR-TP). The following proteins were used to perform ADO/COR and ADO/3'-dINO comparisons: adenosine kinase (ADK); A_1R: Adenosine A1 receptor 1; $A_{2A}R$: adenosine A2A receptor; $A_{2B}R$: adenosine A2B receptor; ADA: adenosine deaminase; PNP: purine nucleoside phosphorylase. To make comparisons of the monophosphate compounds AMP/COR-MP we use CD73, and we also use the two NT5C2 sites: cytosolic 5'-nucleotidase II. It should be noted that in the latter we also performed the 3'dIMP/AMP comparison and added GMP because this is an endogenous ligand of the enzyme. For the ATP/COR-TP comparison, we use CD39. In addition, ADP can also bind CD39 as well as ATP; sAC: soluble adenylate cyclase; MAT: methionine adenosyl transferase; ENPP3: phosphatase ectonucleotide/phosphodiesterase 3. For SAM/3'-dSAM we use DNMT3A: DNA methyltransferase 3 alpha; DNMT1: DNA methyltransferase 1. We performed a search within the Protein Data Bank (PDB) to find potential targets. We limited our search to proteins with resolutions less than 3 Å and to humans (*Homo sapiens*). Table S1 provides an overview of the chosen targets and the PDB codes that correspond to them.

Table S1. Selected targets for molecular docking and their corresponding PDB codes.

**Target**	**PDB**
A_1R	7LD3
$A_{2a}R$	2YDO
$A_{2b}R$	8HDP
ADA	3IAR
ADK (site 1)	1BX4
ADK (site 2)	1BX4
sAC	4USW
CD39	1S1D
CD73	6TVG
DNMT1	4WXX
DNMT3A	6F57
ENPP3	6C02
MAT	4NDN
NT5C2 (site 1)	2JC9
NT5C2 (site 2)	2JC9
PNP	1RFG

Validation of protein targets

To validate our assay, firstly, we downloaded the proteins in a .pdb format file and we began the quality analysis after determining which ones to use for molecular docking. Initially, to assess the quality of the crystallized

protein models, we uploaded the various .pdb files to the MolProbity portal. Below the quality reports that we were able to receive from MolProbity are shown.

Summary statistics

All-Atom Contacts	Clashscore, all atoms:	1.49		99th percentile* (N=598, 1.50Å ± 0.25Å)
	Clashscore is the number of serious steric overlaps (> 0.4 Å) per 1000 atoms.			
Protein Geometry	Poor rotamers	4	1.39%	Goal: <0.3%
	Favored rotamers	276	95.83%	Goal: >98%
	Ramachandran outliers	0	0.00%	Goal: <0.05%
	Ramachandran favored	332	97.65%	Goal: >98%
	Rama distribution Z-score	-1.47 ± 0.40		Goal: abs(Z score) < 2
	MolProbity score^	1.07		99th percentile* (N=4836, 1.50Å ± 0.25Å)
	Cβ deviations >0.25Å	1	0.31%	Goal: 0
	Bad bonds:	0 / 2749	0.00%	Goal: 0%
	Bad angles:	0 / 3708	0.00%	Goal: <0.1%
Peptide Omegas	Cis Prolines:	1 / 11	9.09%	Expected: ≤1 per chain, or ≤5%
Additional validations	Chiral volume outliers	0/411		
	Waters with clashes	0/0	0.00%	See UnDowser table for details

In the two column results, the left column gives the raw count, right column gives the percentage.
* 100th percentile is the best among structures of comparable resolution; 0th percentile is the worst. For clashscore the comparative set of structures was selected in 2004, for MolProbity score in 2006.
^ MolProbity score combines the clashscore, rotamer, and Ramachandran evaluations into a single score, normalized to be on the same scale as X-ray resolution.
Key to table colors and cutoffs here: ?

Figure S6. Quality report for 1BX4 (ADK) obtained from MolProbity.

Summary statistics

All-Atom Contacts	Clashscore, all atoms:	50.29		25th percentile* (N=97, 2.90Å ± 0.25Å)
	Clashscore is the number of serious steric overlaps (> 0.4 Å) per 1000 atoms.			
Protein Geometry	Poor rotamers	37	15.48%	Goal: <0.3%
	Favored rotamers	162	67.78%	Goal: >98%
	Ramachandran outliers	13	4.55%	Goal: <0.05%
	Ramachandran favored	233	81.47%	Goal: >98%
	Rama distribution Z-score	-4.72 ± 0.39		Goal: abs(Z score) < 2
	MolProbity score^	3.80		16th percentile* (N=3760, 2.90Å ± 0.25Å)
	Cβ deviations >0.25Å	2	0.77%	Goal: 0
	Bad bonds:	4 / 2303	0.17%	Goal: 0%
	Bad angles:	9 / 3117	0.29%	Goal: <0.1%
Peptide Omegas	Cis Prolines:	0 / 15	0.00%	Expected: ≤1 per chain, or ≤5%
Low-resolution Criteria	CaBLAM outliers	19	6.7%	Goal: <1.0%
	CA Geometry outliers	3	1.06%	Goal: <0.5%
Additional validations	Chiral volume outliers	0/337		
	Waters with clashes	0/0	0.00%	See UnDowser table for details

In the two column results, the left column gives the raw count, right column gives the percentage.
* 100th percentile is the best among structures of comparable resolution; 0th percentile is the worst. For clashscore the comparative set of structures was selected in 2004, for MolProbity score in 2006.
^ MolProbity score combines the clashscore, rotamer, and Ramachandran evaluations into a single score, normalized to be on the same scale as X-ray resolution.
Key to table colors and cutoffs here: ?

Figure S7. Quality report for 1RFG (PNP) obtained from MolProbity.

Summary statistics

All-Atom Contacts	Clashscore, all atoms:	1.22		99th percentile* (N=718, 1.60Å ± 0.25Å)
	Clashscore is the number of serious steric overlaps (> 0.4 Å) per 1000 atoms.			
Protein Geometry	Poor rotamers	8	3.00%	Goal: <0.3%
	Favored rotamers	255	95.51%	Goal: >98%
	Ramachandran outliers	0	0.00%	Goal: <0.05%
	Ramachandran favored	312	99.05%	Goal: >98%
	Rama distribution Z-score	0.16 ± 0.44		Goal: abs(Z score) < 2
	MolProbity score^	1.20		98th percentile* (N=7200, 1.60Å ± 0.25Å)
	Cβ deviations >0.25Å	1	0.35%	Goal: 0
	Bad bonds:	0 / 2574	0.00%	Goal: 0%
	Bad angles:	0 / 3498	0.00%	Goal: <0.1%
Peptide Omegas	Cis Prolines:	2 / 13	15.38%	Expected: ≤1 per chain, or ≤5%
Additional validations	Chiral volume outliers	0/373		
	Waters with clashes	0/0	0.00%	See UnDowser table for details

In the two column results, the left column gives the raw count, right column gives the percentage.
* 100th percentile is the best among structures of comparable resolution; 0th percentile is the worst. For clashscore the comparative set of structures was selected in 2004, for MolProbity score in 2006.
^ MolProbity score combines the clashscore, rotamer, and Ramachandran evaluations into a single score, normalized to be on the same scale as X-ray resolution.
Key to table colors and cutoffs here: ?

Figure S8. Quality report for 1S1D (CD39) obtained from MolProbity.

Summary statistics

All-Atom Contacts	Clashscore, all atoms:	2.69		98th percentile* (N=598, 1.5Å ± 0.25Å)
	Clashscore is the number of serious steric overlaps (> 0.4 Å) per 1000 atoms.			
Protein Geometry	Poor rotamers	5	1.17%	Goal: <0.3%
	Favored rotamers	416	97.20%	Goal: >98%
	Ramachandran outliers	0	0.00%	Goal: <0.05%
	Ramachandran favored	457	98.70%	Goal: >98%
	Rama distribution Z-score	0.44 ± 0.35		Goal: abs(Z score) < 2
	MolProbity score^	1.11		99th percentile* (N=4836, 1.5Å ± 0.25Å)
	Cβ deviations >0.25Å	0	0.00%	Goal: 0
	Bad bonds:	0 / 4036	0.00%	Goal: 0%
	Bad angles:	1 / 5468	0.02%	Goal: <0.1%
Peptide Omegas	Cis Prolines:	2 / 19	10.53%	Expected: ≤1 per chain, or ≤5%
Additional validations	Chiral volume outliers	0/577		
	Waters with clashes	0/0	0.00%	See UnDowser table for details

In the two column results, the left column gives the raw count, right column gives the percentage.

* 100th percentile is the best among structures of comparable resolution; 0th percentile is the worst. For clashscore the comparative set of structures was selected in 2004, for MolProbity score in 2006.

^ MolProbity score combines the clashscore, rotamer, and Ramachandran evaluations into a single score, normalized to be on the same scale as X-ray resolution.

Key to table colors and cutoffs here: ?

Figure S9. Quality report for 2JC9 (NT5C2) obtained from MolProbity.

Summary statistics

All-Atom Contacts	Clashscore, all atoms:	4.5		100th percentile* (N=75, 3.00Å ± 0.25Å)
	Clashscore is the number of serious steric overlaps (> 0.4 Å) per 1000 atoms.			
Protein Geometry	Poor rotamers	6	2.34%	Goal: <0.3%
	Favored rotamers	231	90.23%	Goal: >98%
	Ramachandran outliers	0	0.00%	Goal: <0.05%
	Ramachandran favored	296	97.05%	Goal: >98%
	Rama distribution Z-score	-2.95 ± 0.40		Goal: abs(Z score) < 2
	MolProbity score^	1.67		100th percentile* (N=3130, 3.00Å ± 0.25Å)
	Cβ deviations >0.25Å	0	0.00%	Goal: 0
	Bad bonds:	0 / 2470	0.00%	Goal: 0%
	Bad angles:	0 / 3365	0.00%	Goal: <0.1%
Peptide Omegas	Cis Prolines:	1 / 11	9.09%	Expected: ≤1 per chain, or ≤5%
Low-resolution Criteria	CaBLAM outliers	0	0.0%	Goal: <1.0%
	CA Geometry outliers	0	0.00%	Goal: <0.5%
Additional validations	Chiral volume outliers	0/399		
	Waters with clashes	0/0	0.00%	See UnDowser table for details

In the two column results, the left column gives the raw count, right column gives the percentage.

* 100th percentile is the best among structures of comparable resolution; 0th percentile is the worst. For clashscore the comparative set of structures was selected in 2004, for MolProbity score in 2006.

^ MolProbity score combines the clashscore, rotamer, and Ramachandran evaluations into a single score, normalized to be on the same scale as X-ray resolution.

Key to table colors and cutoffs here: ?

Figure S10. Quality report for 2YDO ($A_{2A}R$) obtained from MolProbity.

Summary statistics

All-Atom Contacts	Clashscore, all atoms:	1.03		99th percentile* (N=588, 1.52Å ± 0.25Å)
	Clashscore is the number of serious steric overlaps (> 0.4 Å) per 1000 atoms.			
Protein Geometry	Poor rotamers	0	0.00%	Goal: <0.3%
	Favored rotamers	306	98.71%	Goal: >98%
	Ramachandran outliers	1	0.28%	Goal: <0.05%
	Ramachandran favored	353	98.60%	Goal: >98%
	Rama distribution Z-score	-0.25 ± 0.39		Goal: abs(Z score) < 2
	MolProbity score^	0.80		100th percentile* (N=4870, 1.52Å ± 0.25Å)
	Cβ deviations >0.25Å	0	0.00%	Goal: 0
	Bad bonds:	2 / 2989	0.07%	Goal: 0%
	Bad angles:	3 / 4066	0.07%	Goal: <0.1%
Peptide Omegas	Cis Prolines:	1 / 20	5.00%	Expected: ≤1 per chain, or ≤5%
Additional validations	Chiral volume outliers	0/441		
	Waters with clashes	0/0	0.00%	See UnDowser table for details

In the two column results, the left column gives the raw count, right column gives the percentage.

* 100th percentile is the best among structures of comparable resolution; 0th percentile is the worst. For clashscore the comparative set of structures was selected in 2004, for MolProbity score in 2006.

^ MolProbity score combines the clashscore, rotamer, and Ramachandran evaluations into a single score, normalized to be on the same scale as X-ray resolution.

Key to table colors and cutoffs here: ?

Figure S11. Quality report for 3IAR (ADA) obtained from MolProbity.

Summary statistics

All-Atom Contacts	Clashscore, all atoms:	2.07		100th percentile* (N=351, 2.34Å ± 0.25Å)
	Clashscore is the number of serious steric overlaps (> 0.4 Å) per 1000 atoms.			
Protein Geometry	Poor rotamers	22	3.55%	Goal: <0.3%
	Favored rotamers	561	90.48%	Goal: >98%
	Ramachandran outliers	0	0.00%	Goal: <0.05%
	Ramachandran favored	710	96.08%	Goal: >98%
	Rama distribution Z-score	-1.26 ± 0.28		Goal: abs(Z score) < 2
	MolProbity score^	1.66		98th percentile* (N=8616, 2.34Å ± 0.25Å)
	Cβ deviations >0.25Å	0	0.00%	Goal: 0
	Bad bonds:	0 / 5918	0.00%	Goal: 0%
	Bad angles:	0 / 8008	0.00%	Goal: <0.1%
Peptide Omegas	Cis Prolines:	0 / 30	0.00%	Expected: ≤1 per chain, or ≤5%
Additional validations	Chiral volume outliers	0/893		
	Waters with clashes	0/0	0.00%	See UnDowser table for details

In the two column results, the left column gives the raw count, right column gives the percentage.
* 100th percentile is the best among structures of comparable resolution; 0th percentile is the worst. For clashscore the comparative set of structures was selected in 2004, for MolProbity score in 2006.
^ MolProbity score combines the clashscore, rotamer, and Ramachandran evaluations into a single score, normalized to be on the same scale as X-ray resolution.
Key to table colors and cutoffs here:

Figure S12. Quality report for 4NDN (MAT) obtained from MolProbity.

Summary statistics

All-Atom Contacts	Clashscore, all atoms:	5.39		97th percentile* (N=725, 2.05Å ± 0.25Å)
	Clashscore is the number of serious steric overlaps (> 0.4 Å) per 1000 atoms.			
Protein Geometry	Poor rotamers	14	3.52%	Goal: <0.3%
	Favored rotamers	356	89.45%	Goal: >98%
	Ramachandran outliers	0	0.00%	Goal: <0.05%
	Ramachandran favored	442	98.44%	Goal: >98%
	Rama distribution Z-score	-1.00 ± 0.34		Goal: abs(Z score) < 2
	MolProbity score^	1.70		92nd percentile* (N=13512, 2.05Å ± 0.25Å)
	Cβ deviations >0.25Å	6	1.38%	Goal: 0
	Bad bonds:	2 / 3725	0.05%	Goal: 0%
	Bad angles:	8 / 5054	0.16%	Goal: <0.1%
Peptide Omegas	Cis Prolines:	1 / 20	5.00%	Expected: ≤1 per chain, or ≤5%
Additional validations	Chiral volume outliers	0/567		
	Waters with clashes	0/0	0.00%	See UnDowser table for details

In the two column results, the left column gives the raw count, right column gives the percentage.
* 100th percentile is the best among structures of comparable resolution; 0th percentile is the worst. For clashscore the comparative set of structures was selected in 2004, for MolProbity score in 2006.
^ MolProbity score combines the clashscore, rotamer, and Ramachandran evaluations into a single score, normalized to be on the same scale as X-ray resolution.
Key to table colors and cutoffs here:

Figure S13. Quality report for 4USW (sAC) obtained from MolProbity.

Summary statistics

All-Atom Contacts	Clashscore, all atoms:	5.05		99th percentile* (N=226, 2.62Å ± 0.25Å)
	Clashscore is the number of serious steric overlaps (> 0.4 Å) per 1000 atoms.			
Protein Geometry	Poor rotamers	26	2.68%	Goal: <0.3%
	Favored rotamers	864	88.98%	Goal: >98%
	Ramachandran outliers	9	0.78%	Goal: <0.05%
	Ramachandran favored	1075	93.32%	Goal: >98%
	Rama distribution Z-score	-1.85 ± 0.22		Goal: abs(Z score) < 2
	MolProbity score^	2.03		97th percentile* (N=6055, 2.62Å ± 0.25Å)
	Cβ deviations >0.25Å	0	0.00%	Goal: 0
	Bad bonds:	0 / 9377	0.00%	Goal: 0%
	Bad angles:	3 / 12692	0.02%	Goal: <0.1%
Peptide Omegas	Cis Prolines:	3 / 73	4.11%	Expected: ≤1 per chain, or ≤5%
	Cis nonProlines:	8 / 1087	0.74%	Goal: <0.05%
Low-resolution Criteria	CaBLAM outliers	37	3.3%	Goal: <1.0%
	CA Geometry outliers	16	1.41%	Goal: <0.5%
Additional validations	Chiral volume outliers	0/1356		
	Waters with clashes	0/0	0.00%	See UnDowser table for details

In the two column results, the left column gives the raw count, right column gives the percentage.
* 100th percentile is the best among structures of comparable resolution; 0th percentile is the worst. For clashscore the comparative set of structures was selected in 2004, for MolProbity score in 2006.
^ MolProbity score combines the clashscore, rotamer, and Ramachandran evaluations into a single score, normalized to be on the same scale as X-ray resolution.
Key to table colors and cutoffs here:

Figure S14. Quality report for 4WXX (DNMT1) obtained from MolProbity.

Summary statistics

All-Atom Contacts	Clashscore, all atoms:	1.95		100th percentile* (N=744, 1.94Å ± 0.25Å)
	Clashscore is the number of serious steric overlaps (> 0.4 Å) per 1000 atoms.			
Protein Geometry	Poor rotamers	1	0.14%	Goal: <0.3%
	Favored rotamers	708	96.33%	Goal: >98%
	Ramachandran outliers	1	0.12%	Goal: <0.05%
	Ramachandran favored	782	96.42%	Goal: >98%
	Rama distribution Z-score	-1.05 ± 0.27		Goal: abs(Z score) < 2
	MolProbity score^	1.20		99th percentile* (N=11856, 1.94Å ± 0.25Å)
	Cβ deviations >0.25Å	0	0.00%	Goal: 0
	Bad bonds:	0 / 6769	0.00%	Goal: 0%
	Bad angles:	0 / 9202	0.00%	Goal: <0.1%
Peptide Omegas	Cis Prolines:	3 / 60	5.00%	Expected: ≤1 per chain, or ≤5%
Additional validations	Chiral volume outliers	0/964		
	Waters with clashes	0/0	0.00%	See UnDowser table for details

In the two column results, the left column gives the raw count, right column gives the percentage.
* 100th percentile is the best among structures of comparable resolution; 0th percentile is the worst. For clashscore the comparative set of structures was selected in 2004, for MolProbity score in 2006.
^ MolProbity score combines the clashscore, rotamer, and Ramachandran evaluations into a single score, normalized to be on the same scale as X-ray resolution.
Key to table colors and cutoffs here: ?

Figure S15. Quality report for 6C02 (ENPP3) obtained from MolProbity.

Summary statistics

All-Atom Contacts	Clashscore, all atoms:	5.36		100th percentile* (N=59, 2.85Å - 9999Å)
	Clashscore is the number of serious steric overlaps (> 0.4 Å) per 1000 atoms.			
Protein Geometry	Poor rotamers	5	2.09%	Goal: <0.3%
	Favored rotamers	218	91.21%	Goal: >98%
	Ramachandran outliers	1	0.35%	Goal: <0.05%
	Ramachandran favored	268	94.70%	Goal: >98%
	Rama distribution Z-score	-1.53 ± 0.45		Goal: abs(Z score) < 2
	MolProbity score^	1.90		100th percentile* (N=2114, 3.10Å ± 0.25Å)
	Cβ deviations >0.25Å	0	0.00%	Goal: 0
	Bad bonds:	0 / 2317	0.00%	Goal: 0%
	Bad angles:	0 / 3136	0.00%	Goal: <0.1%
Peptide Omegas	Cis Prolines:	0 / 13	0.00%	Expected: ≤1 per chain, or ≤5%
Low-resolution Criteria	CaBLAM outliers	4	1.4%	Goal: <1.0%
	CA Geometry outliers	1	0.36%	Goal: <0.5%
Additional validations	Chiral volume outliers	0/340		
	Waters with clashes	0/0	0.00%	See UnDowser table for details

In the two column results, the left column gives the raw count, right column gives the percentage.
* 100th percentile is the best among structures of comparable resolution; 0th percentile is the worst. For clashscore the comparative set of structures was selected in 2004, for MolProbity score in 2006.
^ MolProbity score combines the clashscore, rotamer, and Ramachandran evaluations into a single score, normalized to be on the same scale as X-ray resolution.
Key to table colors and cutoffs here: ?

Figure S16. Quality report for 6F57 (DNMT3A) obtained from MolProbity.

Summary statistics

All-Atom Contacts	Clashscore, all atoms:	1.31		99th percentile* (N=558, 1.48Å ± 0.25Å)
	Clashscore is the number of serious steric overlaps (> 0.4 Å) per 1000 atoms.			
Protein Geometry	Poor rotamers	4	0.85%	Goal: <0.3%
	Favored rotamers	452	96.17%	Goal: >98%
	Ramachandran outliers	1	0.19%	Goal: <0.05%
	Ramachandran favored	504	96.55%	Goal: >98%
	Rama distribution Z-score	-0.86 ± 0.32		Goal: abs(Z score) < 2
	MolProbity score^	1.08		99th percentile* (N=4443, 1.48Å ± 0.25Å)
	Cβ deviations >0.25Å	0	0.00%	Goal: 0
	Bad bonds:	0 / 4297	0.00%	Goal: 0%
	Bad angles:	0 / 5873	0.00%	Goal: <0.1%
Peptide Omegas	Cis Prolines:	2 / 25	8.00%	Expected: ≤1 per chain, or ≤5%
Additional validations	Chiral volume outliers	0/666		
	Waters with clashes	0/0	0.00%	See UnDowser table for details

In the two column results, the left column gives the raw count, right column gives the percentage.
* 100th percentile is the best among structures of comparable resolution; 0th percentile is the worst. For clashscore the comparative set of structures was selected in 2004, for MolProbity score in 2006.
^ MolProbity score combines the clashscore, rotamer, and Ramachandran evaluations into a single score, normalized to be on the same scale as X-ray resolution.
Key to table colors and cutoffs here: ?

Figure S17. Quality report for 6TVG (CD73) obtained from MolProbity.

Summary statistics

All-Atom Contacts	Clashscore, all atoms:	2.77		100th percentile* (N=41, 2.95Å - 9999Å)
	Clashscore is the number of serious steric overlaps (> 0.4 Å) per 1000 atoms.			
Protein Geometry	Poor rotamers	0	0.00%	Goal: <0.3%
	Favored rotamers	242	97.98%	Goal: >98%
	Ramachandran outliers	0	0.00%	Goal: <0.05%
	Ramachandran favored	273	96.13%	Goal: >98%
	Rama distribution Z-score	-4.18 ± 0.36		Goal: abs(Z score) < 2
	MolProbity score^	1.33		100th percentile* (N=1544, 3.20Å ± 0.25Å)
	Cβ deviations >0.25Å	0	0.00%	Goal: 0
	Bad bonds:	0 / 2331	0.00%	Goal: 0%
	Bad angles:	5 / 3178	0.16%	Goal: <0.1%
Peptide Omegas	Cis Prolines:	0 / 14	0.00%	Expected: ≤1 per chain, or ≤5%
Low-resolution Criteria	CaBLAM outliers	1	0.4%	Goal: <1.0%
	CA Geometry outliers	0	0.00%	Goal: <0.5%
Additional validations	Chiral volume outliers	0/387		
	Waters with clashes	0/0	0.00%	See UnDowser table for details

In the two column results, the left column gives the raw count, right column gives the percentage.

* 100th percentile is the best among structures of comparable resolution; 0th percentile is the worst. For clashscore the comparative set of structures was selected in 2004, for MolProbity score in 2006.

^ MolProbity score combines the clashscore, rotamer, and Ramachandran evaluations into a single score, normalized to be on the same scale as X-ray resolution.

Key to table colors and cutoffs here:

Figure S18. Quality report for 7LD3 (A_1R) obtained from MolProbity.

Summary statistics

All-Atom Contacts	Clashscore, all atoms:	7.26		97th percentile* (N=41, 2.95Å - 9999Å)
	Clashscore is the number of serious steric overlaps (> 0.4 Å) per 1000 atoms.			
Protein Geometry	Poor rotamers	5	2.16%	Goal: <0.3%
	Favored rotamers	208	90.04%	Goal: >98%
	Ramachandran outliers	0	0.00%	Goal: <0.05%
	Ramachandran favored	261	95.26%	Goal: >98%
	Rama distribution Z-score	-1.15 ± 0.50		Goal: abs(Z score) < 2
	MolProbity score^	1.98		100th percentile* (N=1544, 3.20Å ± 0.25Å)
	Cβ deviations >0.25Å	0	0.00%	Goal: 0
	Bad bonds:	0 / 2216	0.00%	Goal: 0%
	Bad angles:	0 / 3027	0.00%	Goal: <0.1%
Peptide Omegas	Cis Prolines:	0 / 11	0.00%	Expected: ≤1 per chain, or ≤5%
Low-resolution Criteria	CaBLAM outliers	2	0.7%	Goal: <1.0%
	CA Geometry outliers	2	0.75%	Goal: <0.5%
Additional validations	Tetrahedral geometry outliers	1		
	Waters with clashes	0/0	0.00%	See UnDowser table for details

In the two column results, the left column gives the raw count, right column gives the percentage.

* 100th percentile is the best among structures of comparable resolution; 0th percentile is the worst. For clashscore the comparative set of structures was selected in 2004, for MolProbity score in 2006.

^ MolProbity score combines the clashscore, rotamer, and Ramachandran evaluations into a single score, normalized to be on the same scale as X-ray resolution.

Key to table colors and cutoffs here:

Figure S19. Quality report for 8HDQ ($A_{2B}R$) obtained from MolProbity.

Identification of the docking site

To conduct molecular docking studies, we needed to identify suitable docking sites. To achieve this, we searched in the UniProt database for amino acid residues reported in the function section of our respective selected targets. These residues play a crucial role in the active sites. Next, we realized the prediction of pockets to further refine the active site boundaries. To do this, we utilized the DoGSiteScorer tool. This tool predicts potential binding pockets within the protein structures. Our criteria for selecting pockets were twofold:

1. The pocket had to include the amino acid residues previously identified in UniProt.
2. We prioritized pockets based on their drug score, choosing the one with the highest suitability.

The pockets selected for our candidate targets can be observed below.

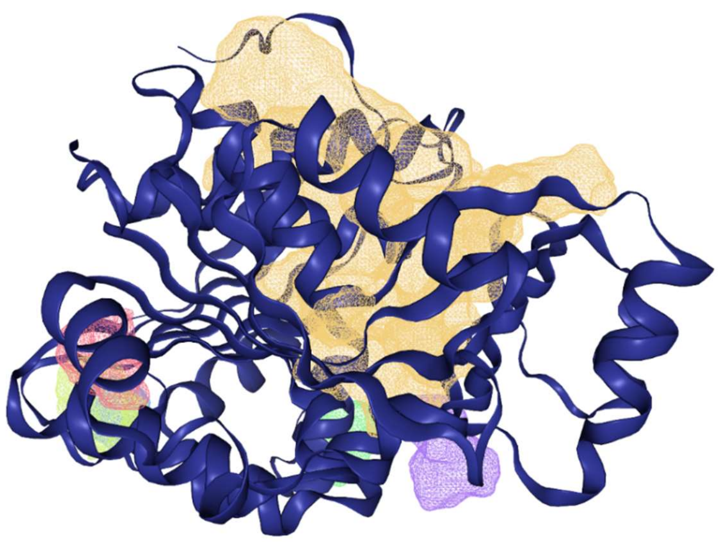

Figure S20. Predicted pockets for 1BX4 (ADK) using DoGSiteScorer.

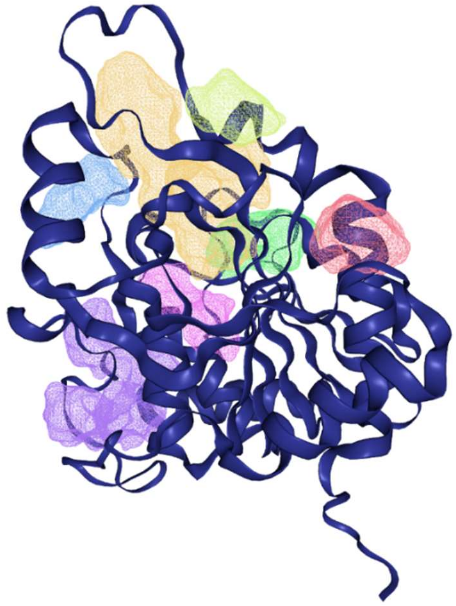

Figure S21. Predicted pockets for 1RFG (PNP) using DoGSiteScorer.

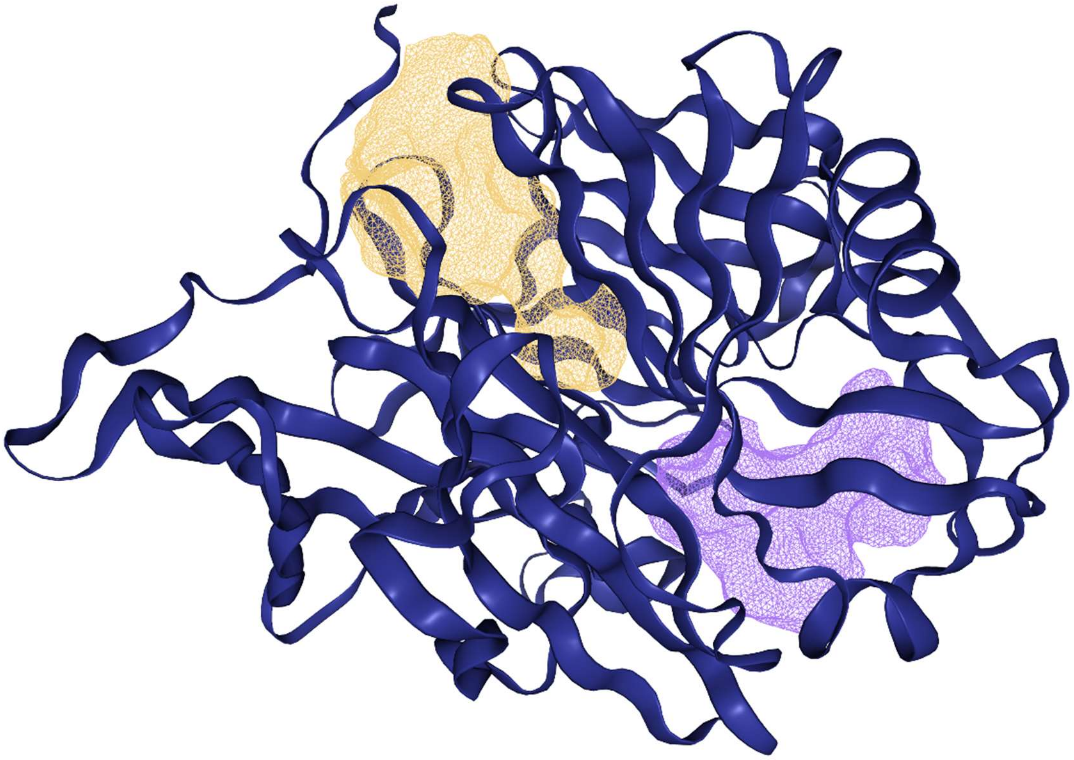

Figure S22. Predicted pockets for 1S1D (CD39) using DoGSiteScorer.

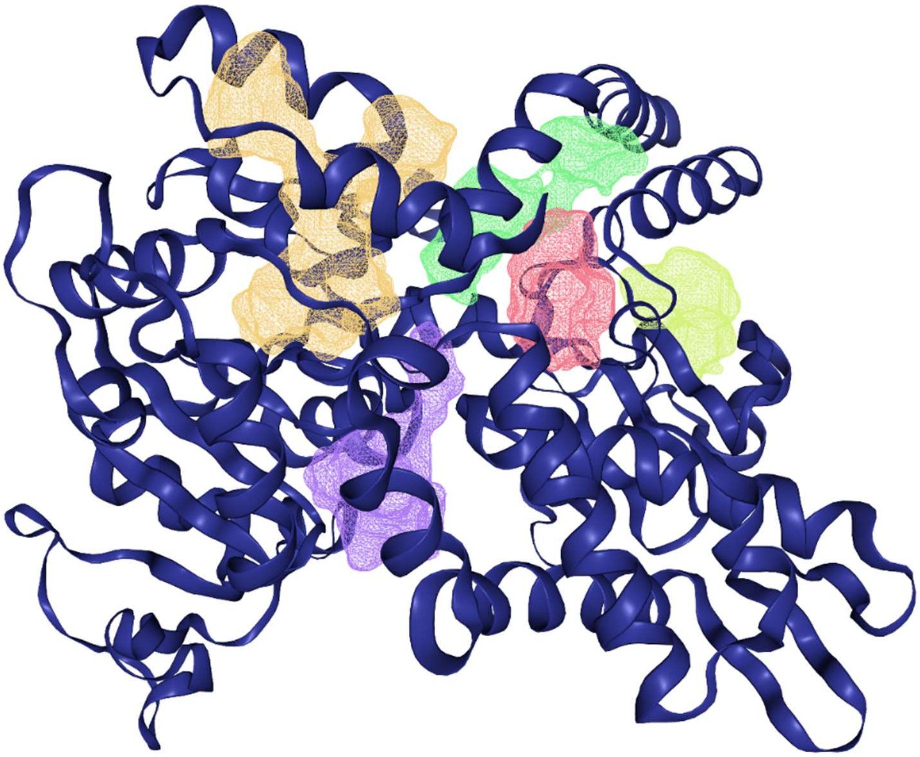

Figure S23. Predicted pockets for 2JC9 (NT5C2) using DoGSiteScorer.

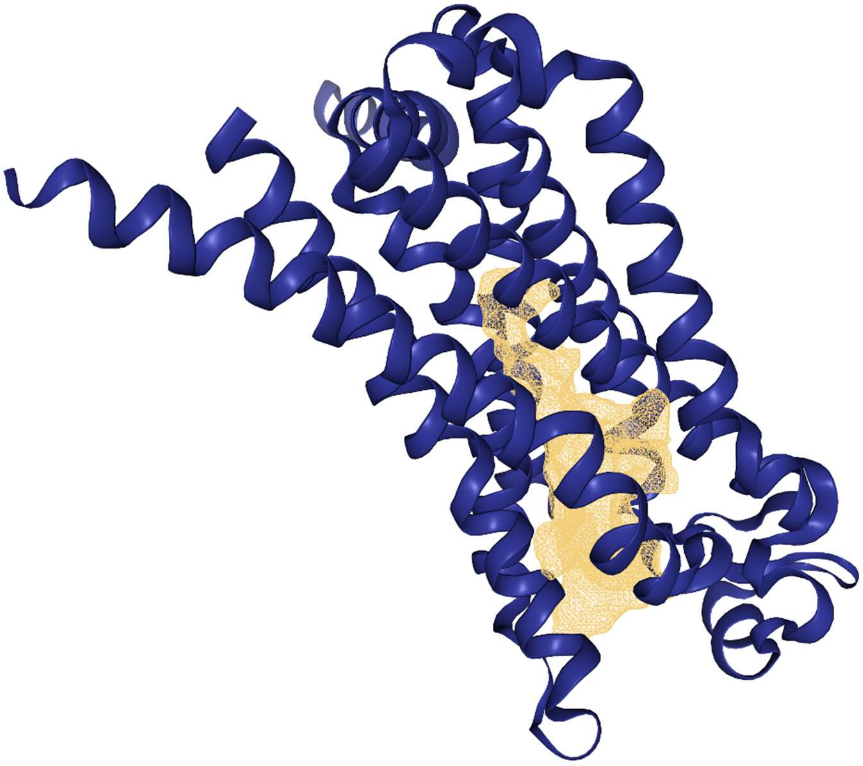

Figure S24. Predicted pockets for 2YDO ($A_{2A}R$) using DoGSiteScorer.

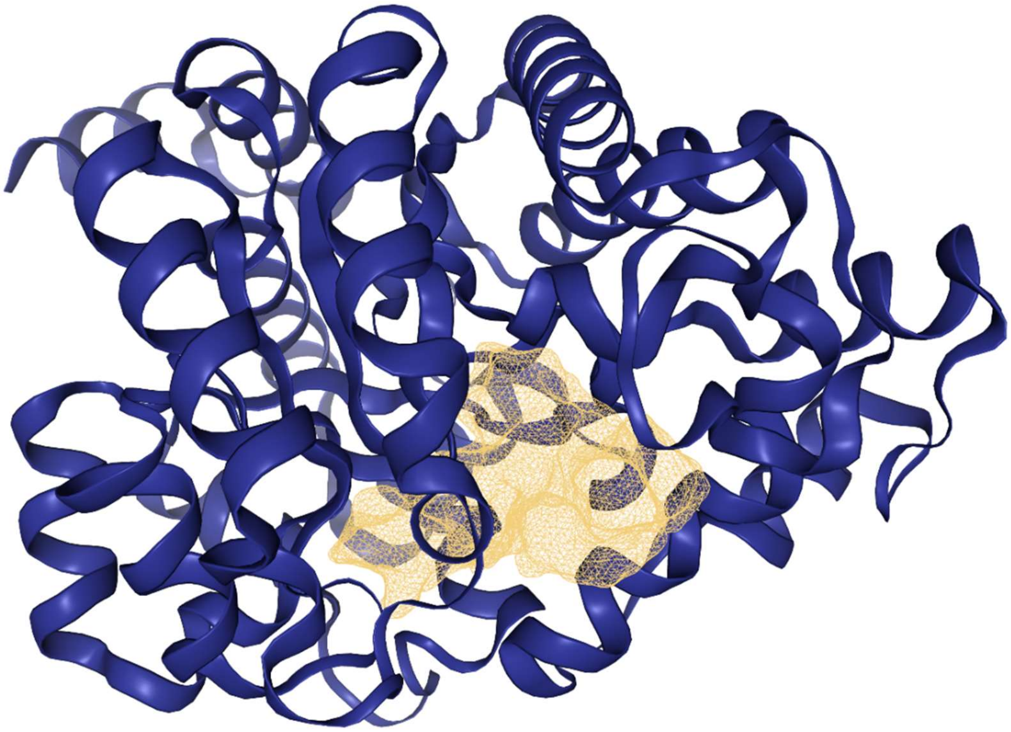

Figure S25. Predicted pockets for 3IAR (ADA) using DoGSiteScorer.

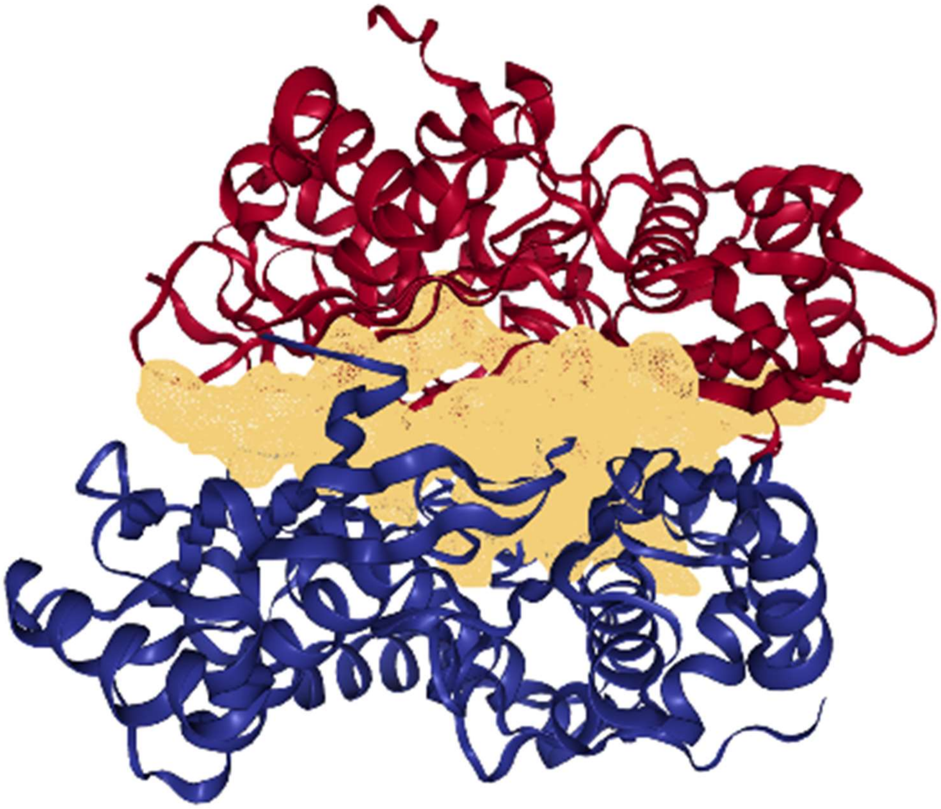

Figure S26. Predicted pockets for 4NDN (MAT) using DoGSiteScorer.

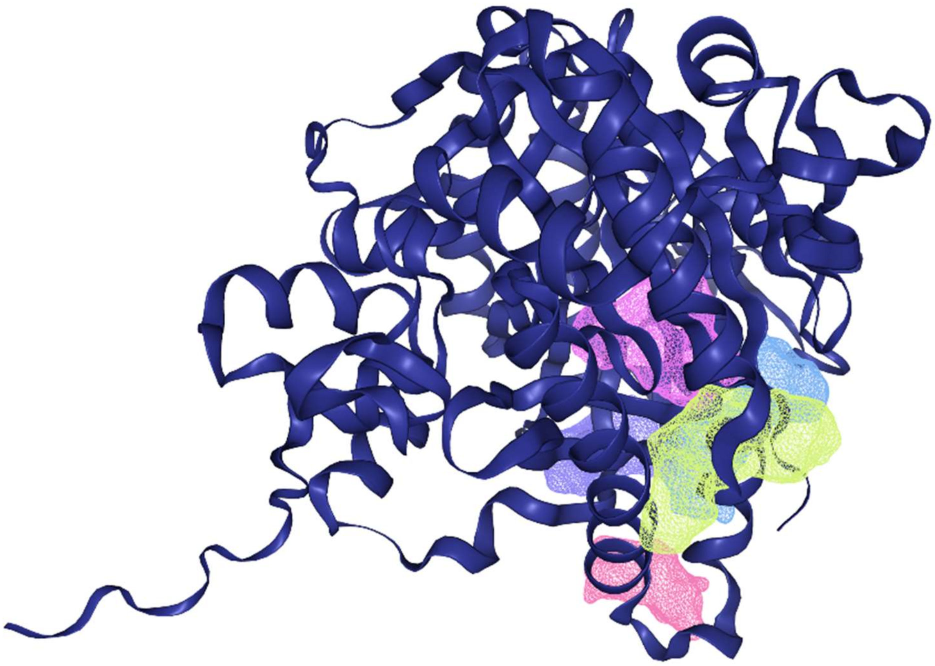

Figure S27. Predicted pockets for 4USW (sAC) using DoGSiteScorer.

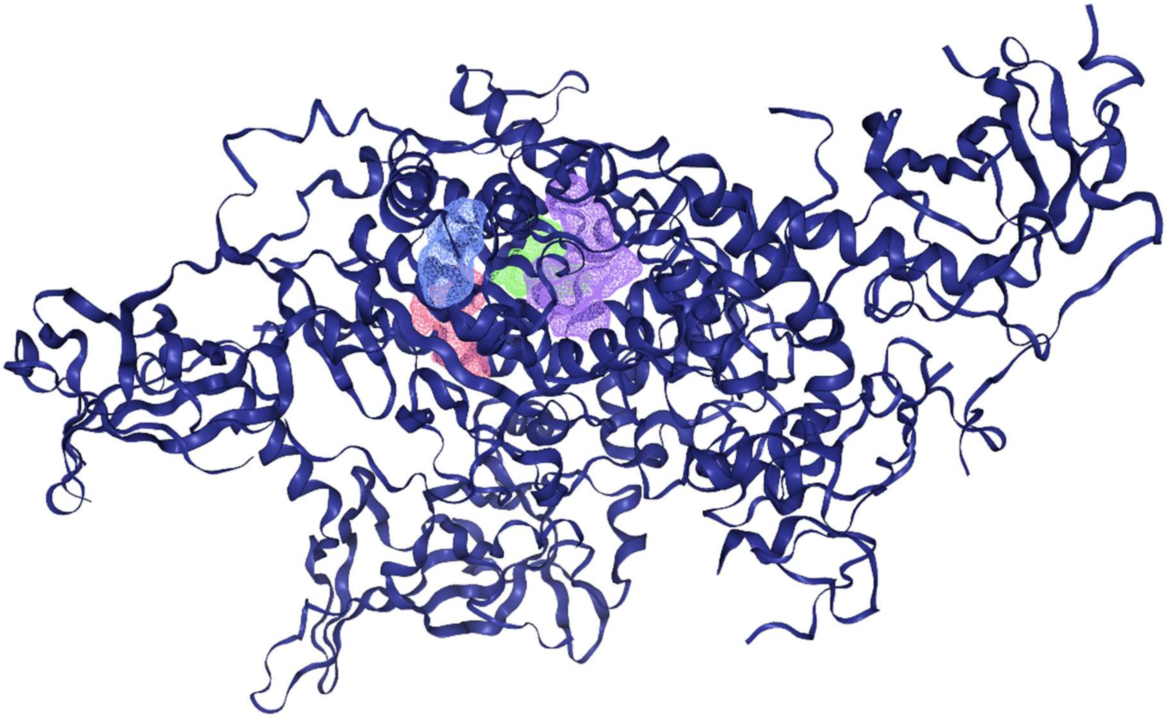

Figure S28. Predicted pockets for 4WXX (DNMT1) using DoGSiteScorer.

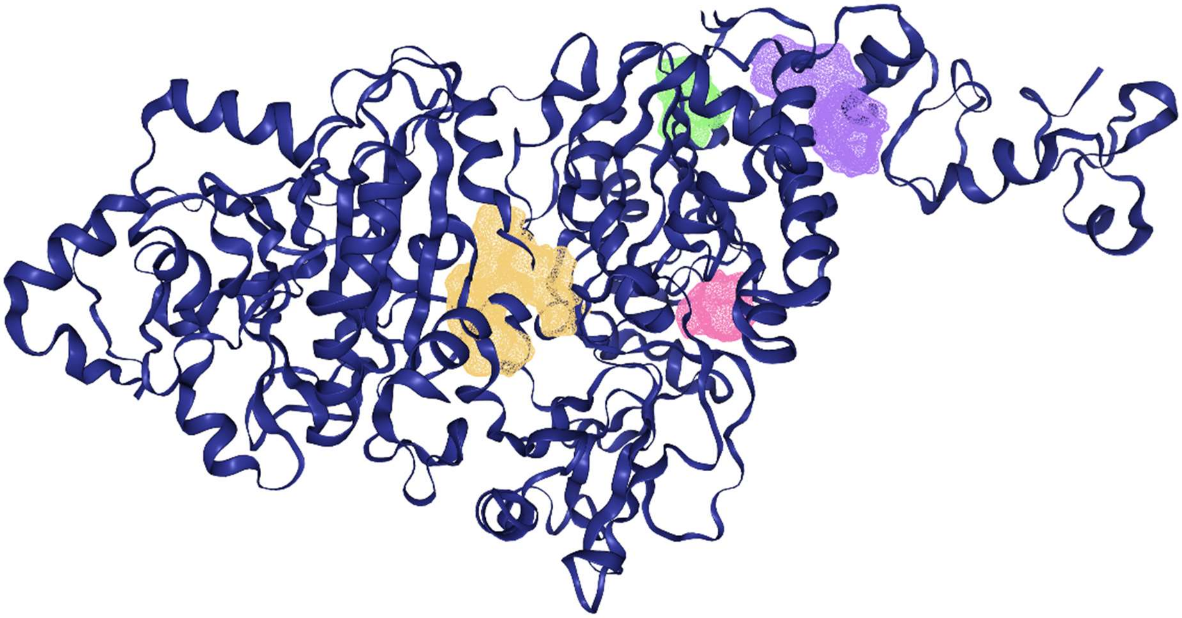

Figure S29. Predicted pockets for 6C02 (ENPP3) using DoGSiteScorer.

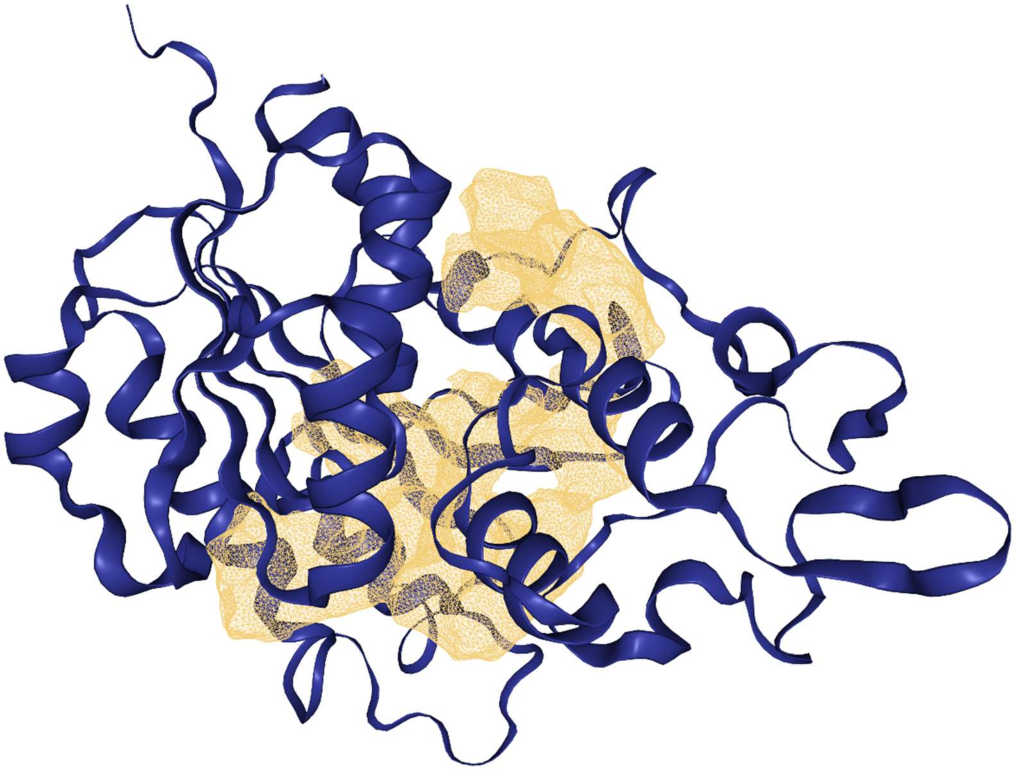

Figure S30. Predicted pockets for 6F57 (DNMT3A) using DoGSiteScorer.

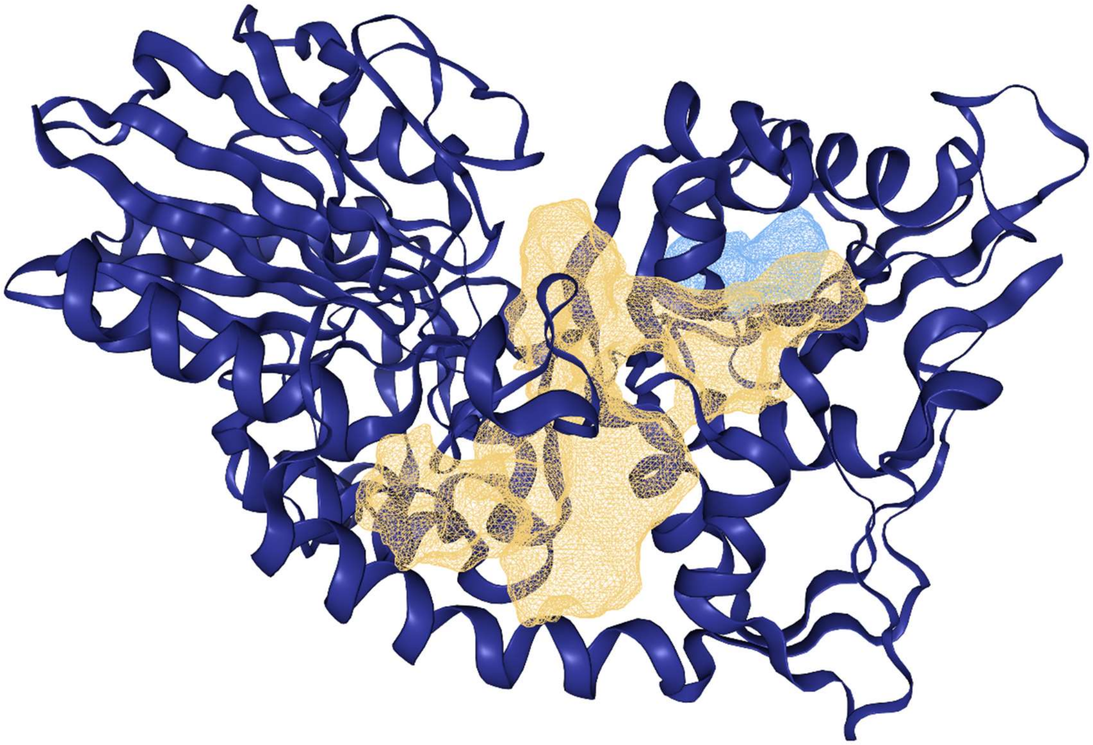

Figure S31. Predicted pockets for 6TVG (CD73) using DoGSiteScorer.

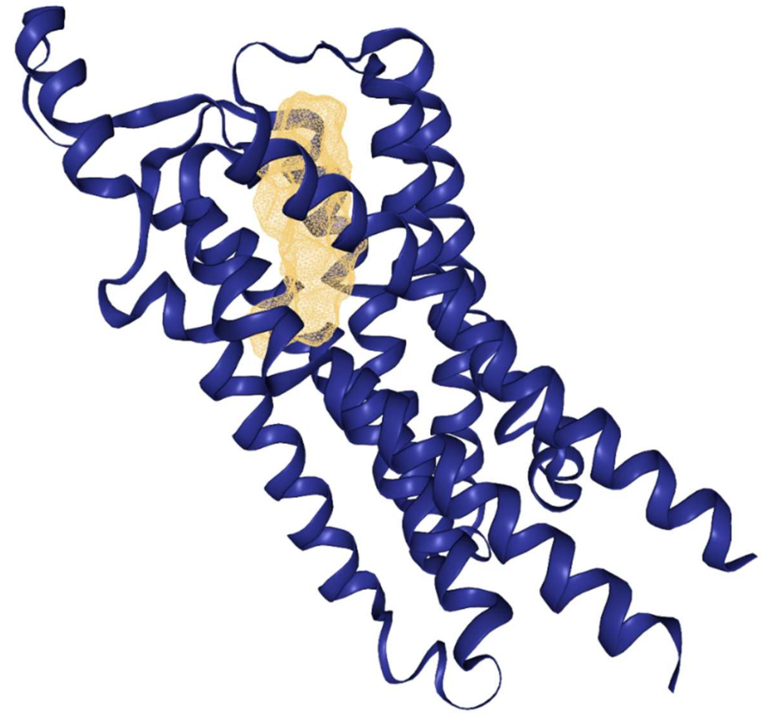

Figure S32. Predicted pockets for 7LD3 (A_1R) using DoGSiteScorer.

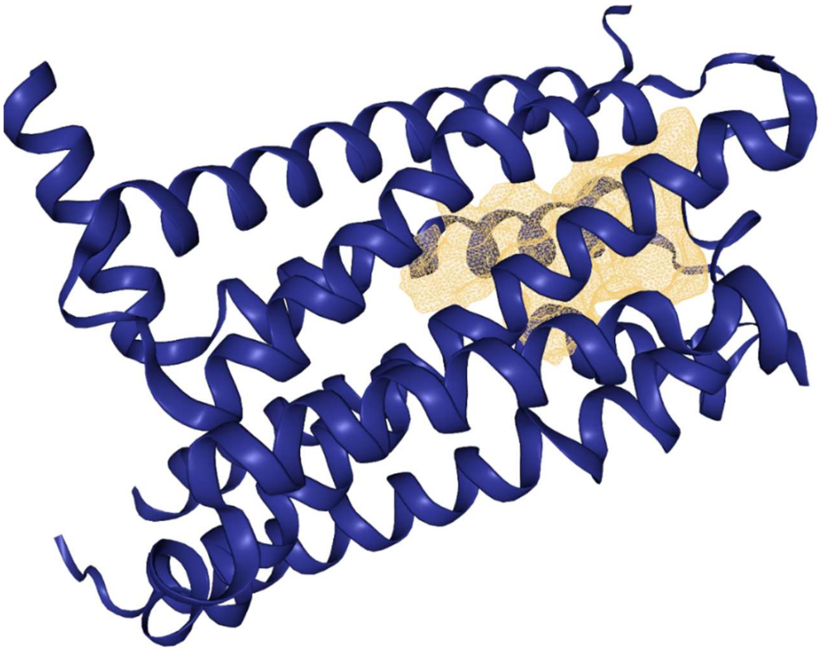

Figure S33. Predicted pockets for 8HDQ ($A_{2B}R$) using DoGSiteScorer.

Validation of Molecular Docking Methodology

After identifying the docking site for our candidate targets, we validated the molecular docking methodology using the redocking approach. Our docking studies, performed in AutoDock 4.2 with a rigid modality, followed our established methodology. We utilized selected candidate targets and their corresponding crystallized ligands for the simulation. Subsequently, we compared our simulated conformation with the original ligand's crystallized conformation in UCSF Chimera. The coordinates employed in the docking studies, and the low binding energy, high binding energy, and inhibition constant values obtained in the docking studies are shown below. To complement this section, we performed the docking studies in AutoDock Vina and SwissDock to compare with the results obtained as described previously.

Table S2. 3D sizes and coordinates for the grid boxes employed in the docking studies.

Target	Grid box size			Grid box coordinates			RMSD for the redocked ligand	Molecule used in validation
	X	Y	Z	X	Y	Z		
A_1R	36	24	28	88.427	116.87	115.864	0.707	ADO
$A_{2a}R$	30	34	34	-29.855	8.243	-25.266	2.689	ADO
$A_{2b}R$	34	30	34	116.605	101.097	78.273	1.184	ADO
ADA	40	38	32	4.072	-3.518	-1.488	0.476	2'-deoxyadenosine
ADK (site 1)	38	38	34	40.394	16.142	38.218	0.308	ADO
ADK (site 2)	34	36	30	45.069	24.176	50.295	0.7	ADO
sAC	38	34	34	-20.962	-34.085	3.951	4.603	ATP
CD39	24	40	30	21.451	-3.302	49.989	4.377	GMP-CP
CD73	42	32	32	10.523	15.296	-35.314	4.79	AMPCP
DNMT1	26	40	28	-46.819	62.104	7.496	0.966	SAH
DNMT3A	42	42	46	553.891	16.493	170.868	3.247	SAH
ENPP3	28	34	50	-23.838	28.459	-33.017	4.9	AMPCPP
MAT	32	32	32	-8.979	-5.53	-81.314	0.776	SAM
NT5C2 (site 1)	32	26	42	-21.567	32.596	49.902	2.287	ADO
NT5C2 (site 2)	20	20	26	-2.403	18.136	58.82	1.303	ADO
PNP	40	34	46	54.63	128.058	58.091	0.912	GMP-CP

Table S3. Low binding energy, high binding energy, and inhibition constant were obtained in the docking studies by AutoDock 4.2.

Target	Molecules	Low binding energy	High binding energy	Inhibition constant
A_1R	3'-dINO	-5.17	-5.44	103.63 µM
	ADO	-4.38	-4.81	300.18 µM
	COR	-5.73	-5.84	52.62 µM
$A_{2a}R$	3'-dINO	-5.01	-5.06	195.64 µM
	ADO	-4.65	-5.26	140.14 µM

	COR	-5.32	-5.44	103.63 µM
$A_{2b}R$	3’-dINO	-4.05	-4.31	693.89 µM
	ADO	-3.18	-3.9	1.39 µM
	COR	-4.18	-5.35	118.85 µM
ADA	3’-dINO	-5.33	-6.78	10.63 µM
	ADO	-4.7	-7.84	1.8 µM
	COR	-7.03	-7.94	1.51 µM
ADK (site 1)	3’-dINO	-5.54	-5.95	43.48 µM
	ADO	-4.27	-6.24	26.46 µM
	COR	-6.61	-6.92	8.53 µM
ADK (site 2)	3’-dINO	-4.37	-5.23	147.84 µM
	ADO	-3.8	-4.25	765.09 µM
	COR	-4.86	-5.56	83.86 µM
sAC	ATP	-2.49	-5.78	57.89 µM
	COR-TP	-3.04	-7.04	6.9 µM
CD39	ATP	-2.59	-6.12	32.89 µM
	ADP	-4.63	-7.51	3.11 µM
	COR-TP	-1.17	-7.25	4.87 µM
	COR-DP	-5.1	-7.92	1.58 µM
CD73	AMP	-5.42	-7.52	3.08 µM
	COR-MP	-5.9	-7.21	5.22 µM
DNMT1	3’-dSAM	-5.85	-10.45	21.75 nM
	SAM	-4.59	-9.1	213.83 nM
DNMT3A	3’-dSAM	-6.3	-9.94	51.94 nM
	SAM	-5.35	-8.34	774.24 nM
ENPP3	ATP	-1.14	-4.15	906.02 µM
	COR-TP	-0.65	-4.48	521.62 µM
MAT	ATP	-1.42	-3.52	2.61 mM
	COR-TP	0.9	-3.75	1.79 mM
NT5C2 (site 1)	3’-dIMP	-5.53	-6.88	9.09 µM
	AMP	-5.04	-5.71	65.11 µM
	COR-MP	-6.09	-7.2	5.52 µM
	GMP	-4.98	-6.5	17.31 µM
	IMP	-4.75	-6.6	14.45 µM
NT5C2 (site 2)	3’-dIMP	-5.34	-6.34	22.55 µM
	AMP	-5.26	-6.55	15.93 µM
	COR-MP	-6.76	-7.04	6.94 µM
	GMP	-3.48	-6.93	8.27 µM
	IMP	-4.74	-7.11	6.1 µM
PNP	3’-dINO	-6.68	-7.04	6.95 µM

	COR	-6.46	-7.2	5.25 µM
	GUA	-6.1	-7.23	5.02 µM
	INO	-5.54	-6.58	15.12 µM

Table S4. Comparison between the high binding energy obtained in the docking studies by AutoDock 4.2, Autodock Vina, and SwissDock, and the RMSD values between the conformers obtained from Autodock 4.2 and Autodock Vina.

Target	Molecules	High binding energy according to AutoDock 4.2	High binding energy according to AutoDock Vina	High binding energy according to SwissDock	RMSD
A_1R	3'-dINO	-5.44	-7.3	-7.7079	0.712
	ADO	-4.81	-7.5	-7.7092	2.719
	COR	-5.84	-7.3	-7.7048	0.683
$A_{2a}R$	3'-dINO	-5.06	-7.4	-7.5525	2.532
	ADO	-5.26	-7.1	-7.9842	2.811
	COR	-5.44	-7.1	-7.7847	3.123
$A_{2b}R$	3'-dINO	-4.31	-5.9	-6.7642	3.511
	ADO	-3.9	-6.2	-6.8946	3.306
	COR	-5.35	-5.6	-6.9494	10.283
ADA	3'-dINO	-6.78	-6.2	-7.1670	6.147
	ADO	-7.84	-6.2	-7.2917	22.53
	COR	-7.94	-6.5	-7.2591	6.377
ADK (site 1)	3'-dINO	-5.95	-7.3	*	3.644
	ADO	-6.24	-7.6	*	1.34
	COR	-6.92	-7.1	*	3.538
ADK (site 2)	3'-dINO	-5.23	-7.3	*	10.365
	ADO	-4.25	-7.6	*	9.872
	COR	-5.56	-7.4	*	9.787
sAC	ATP	-5.78	-8.1	*	5.568
	COR-TP	-5.73	-8.2	*	3.715
CD39	ATP	-7.51	-8.4	-8.2051	3.77
	ADP	-6.12	-8.3	-7.5128	6.617
	COR-TP	-7.92	-8	-7.9662	3.626
	COR-DP	-7.43	-8.1	-7.7227	3.359
CD73	AMP	7.52	-7.3	*	9.731
	COR-MP	-7.21	-7.5	*	11.15
DNMT1	3'-dSAM	-10.45	-7.7	*	4.13
	SAM	-9.1	-7.9	*	3.981
DNMT3A	3'-dSAM	-9.94	-6.9	*	4.728
	SAM	-8.34	-8.1	*	1.845

ENPP3	ATP	-4.15	-8.5	*	2.962
	COR-TP	-5.08	-8.1	*	2.682
MAT	ATP	-3.52	-9.3	*	4.314
	COR-TP	-2.93	-9.3	*	4.197
NT5C2 (site 1)	3’-dIMP	-6.88	-7	-7.2170	11.11
	AMP	-5.71	-7.6	-7.7258	10.875
	COR-MP	-7.2	-7.6	-7.4326	9.273
	GMP	-6.5	-8.3	-7.7291	5.352
	IMP	-6.6	-7.7	-7.6382	9.471
NT5C2 (site 2)	3’-dIMP	-6.34	-6.1	-6.3821	2.565
	AMP	-6.55	-7.1	-7.0200	3.017
	COR-MP	-7.04	-7.2	-6.4609	3.591
	GMP	-6.93	-6.9	-6.5918	3.766
	IMP	-7.11	-6.7	-6.2997	2.256
PNP	3’-dINO	-7.04	-7.9	-7.5835	3.329
	COR	-7.2	-7.6	-7.1617	2.329
	GUA	-7.23	-7	-7.6316	2.482
	INO	-6.58	-7.1	-7.6690	3.226

*The docking studies could not be performed by SwissDock because the molecule exceeded the time according to the platform.

Binding modes of biguanides and candidate metabolites in the docked target according to Autodock 4.2.

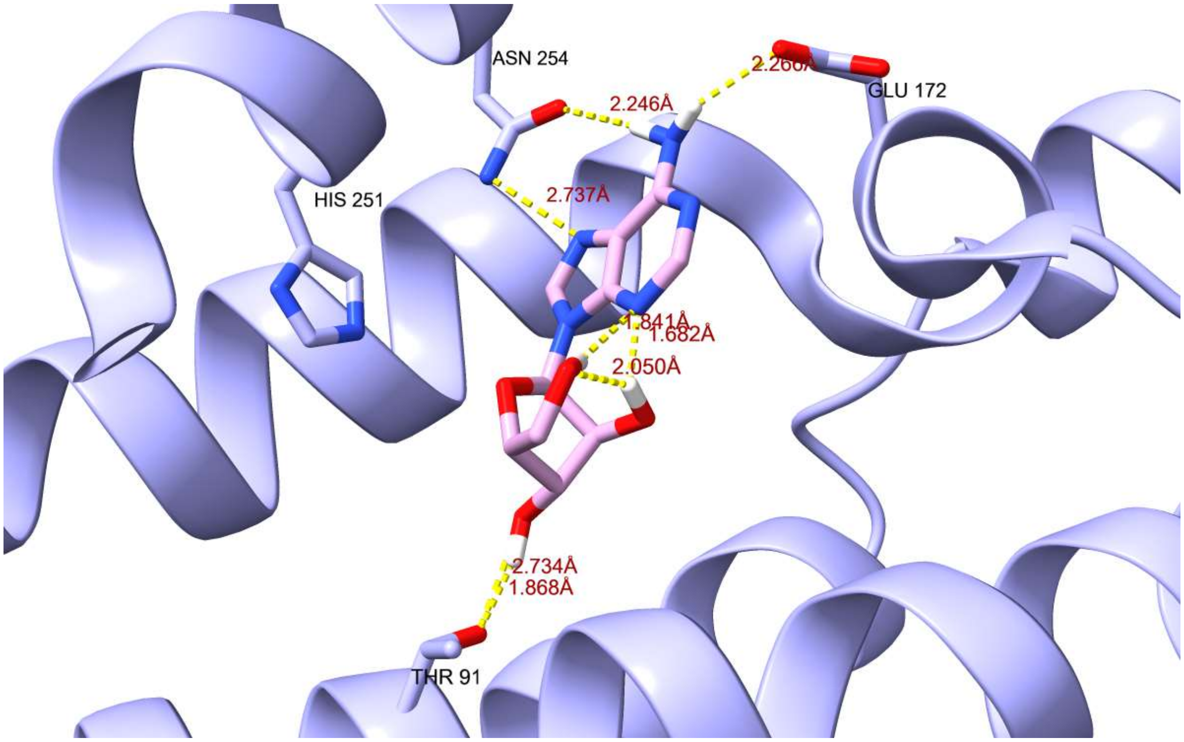

Figure S34. Predicted binding mode of 3’-dINO with A_1R.

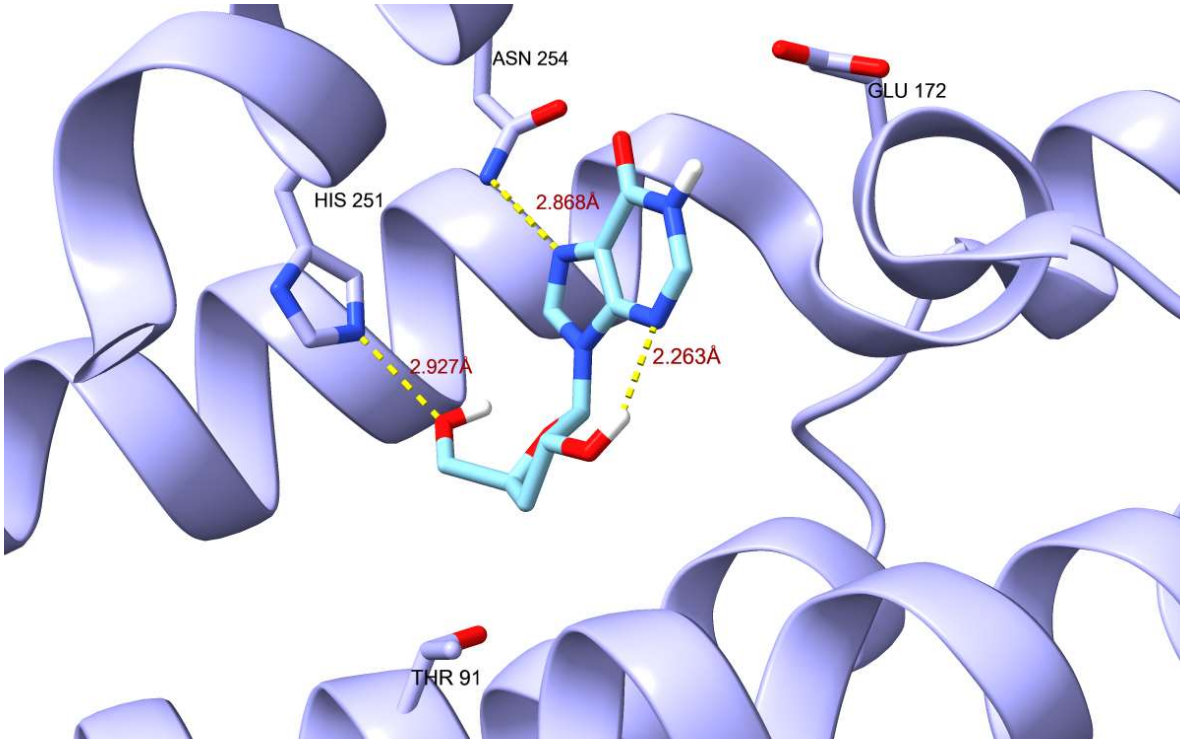

Figure S35. Predicted binding mode of ADO with A_1R.

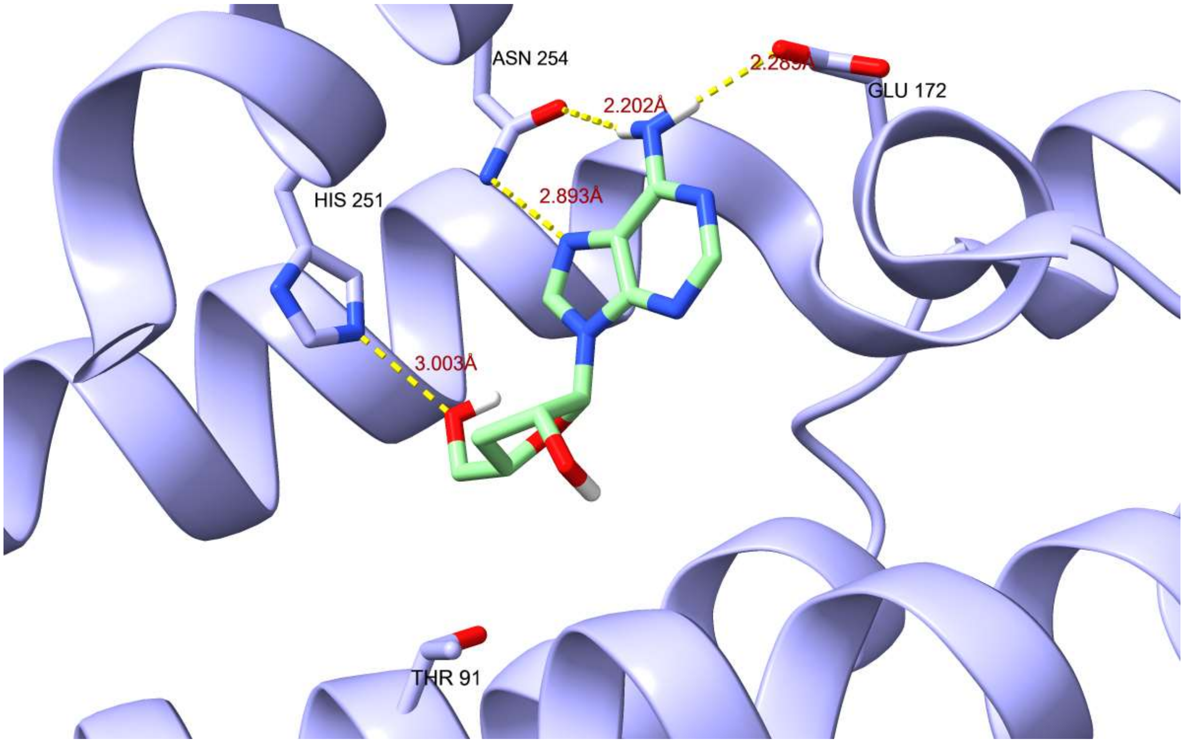

Figure S36. Predicted binding mode of COR with A_1R.

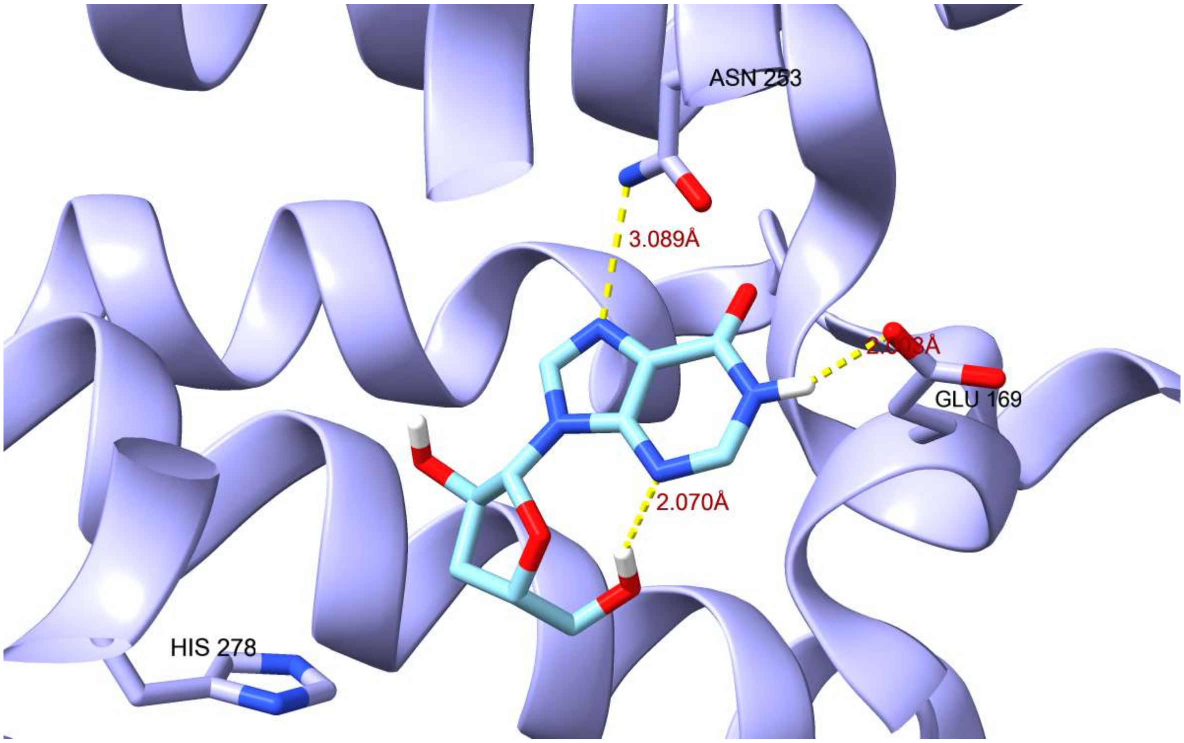

Figure S37. Predicted binding mode of 3'-dINO with $A_{2A}R$.

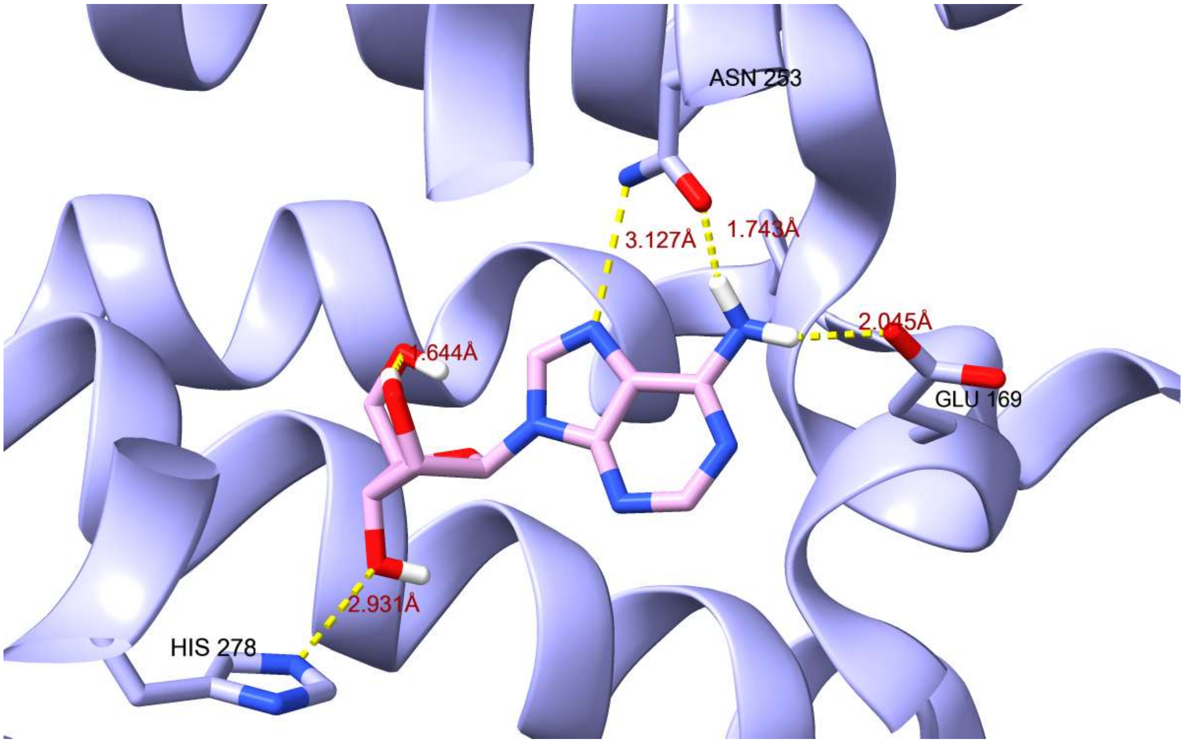

Figure S38. Predicted binding mode of ADO with $A_{2A}R$.

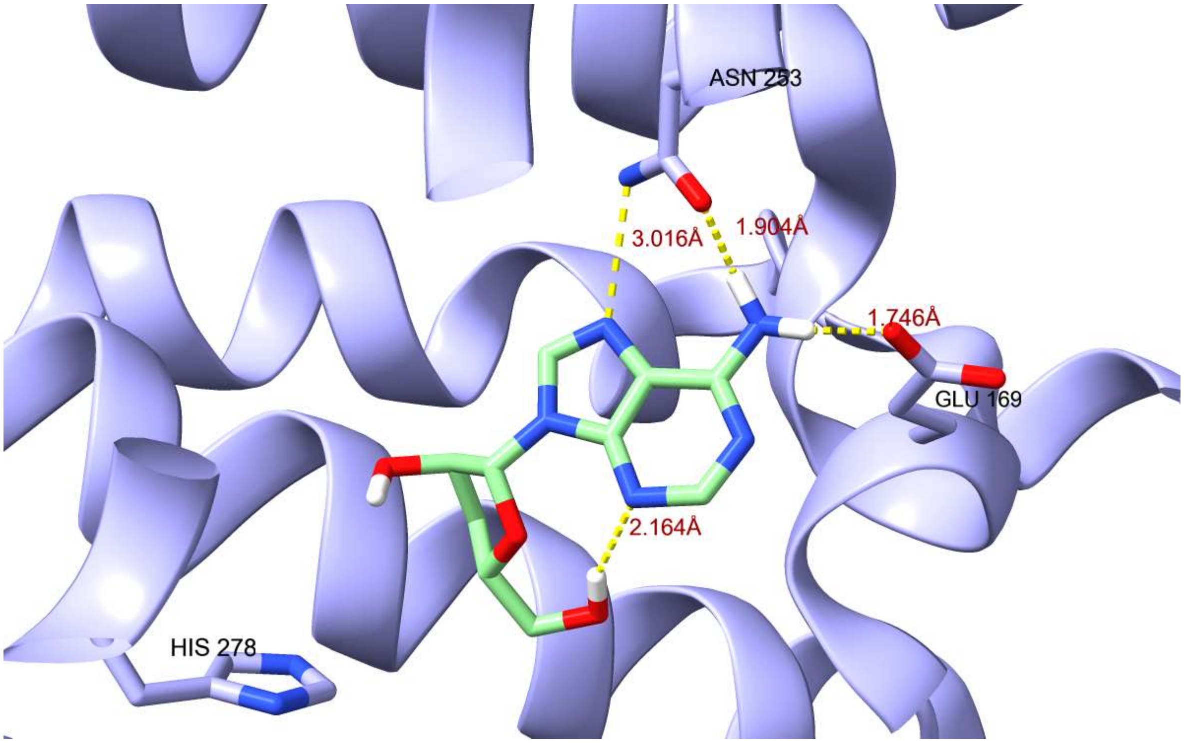

Figure S39. Predicted binding mode of COR with $A_{2A}R$.

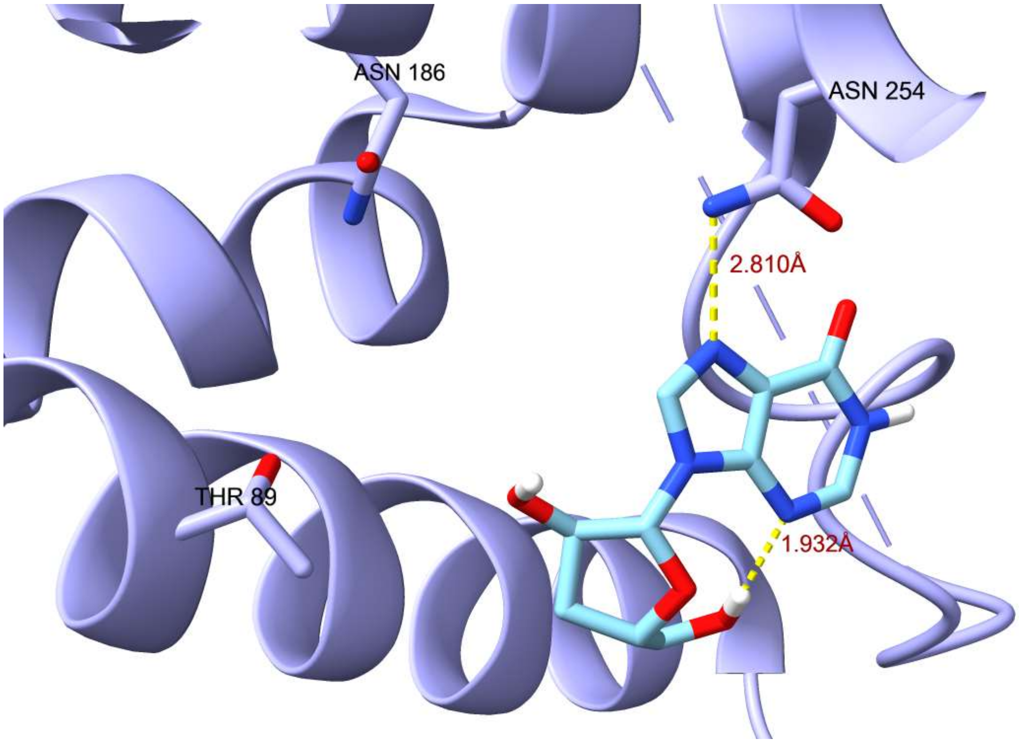

Figure S40. Predicted binding mode of 3’-dINO with $A_{2B}R$.

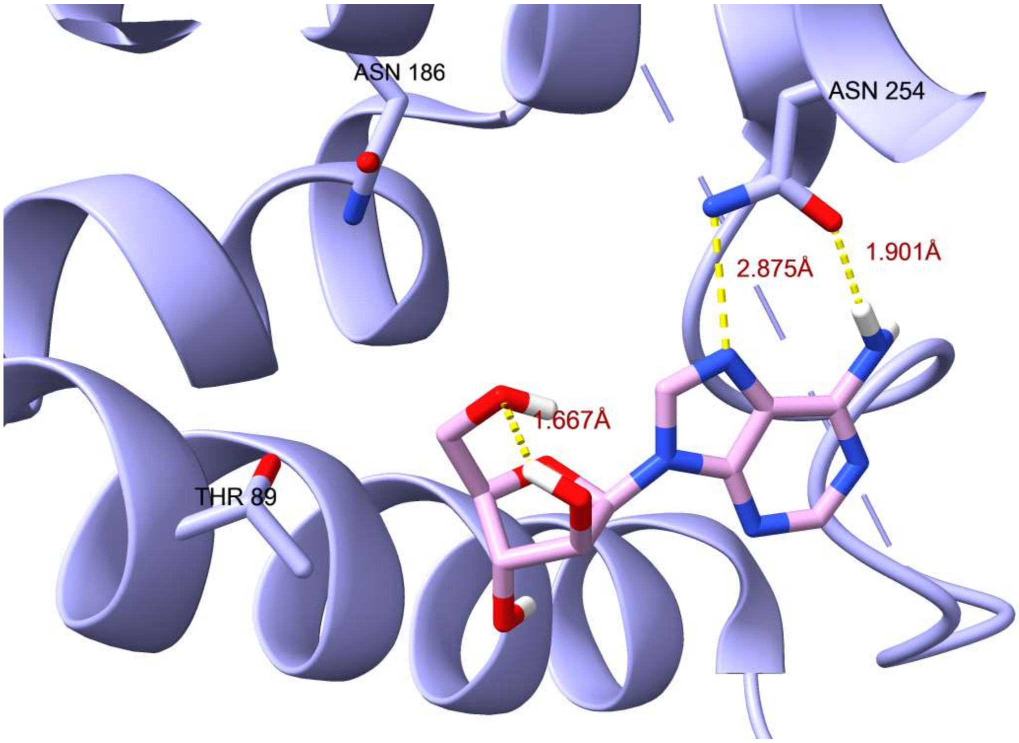

Figure S41. Predicted binding mode of ADO with $A_{2B}R$.

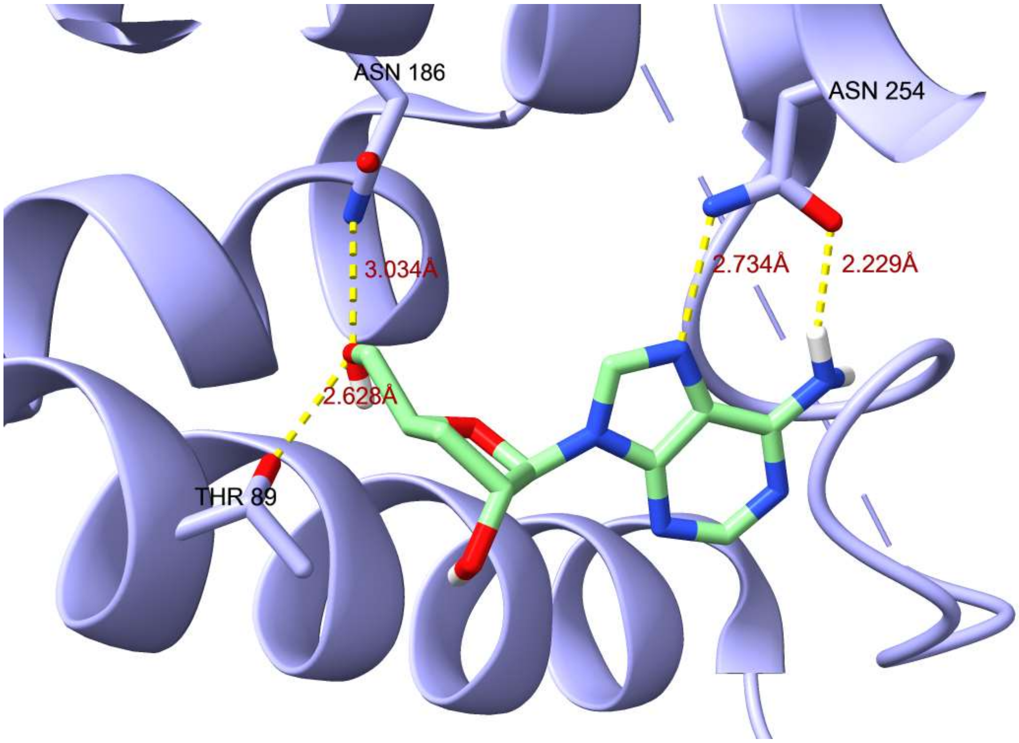

Figure S42. Predicted binding mode of COR with $A_{2B}R$.

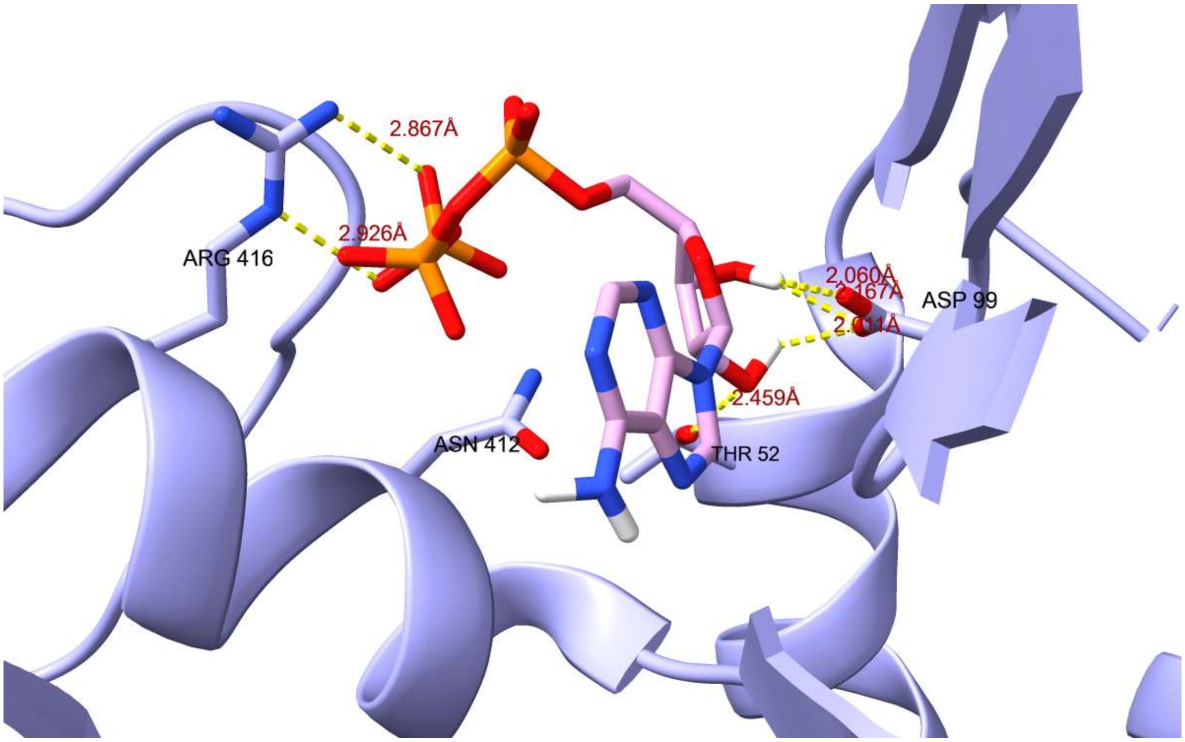

Figure S43. Predicted binding mode of ATP with sAC.

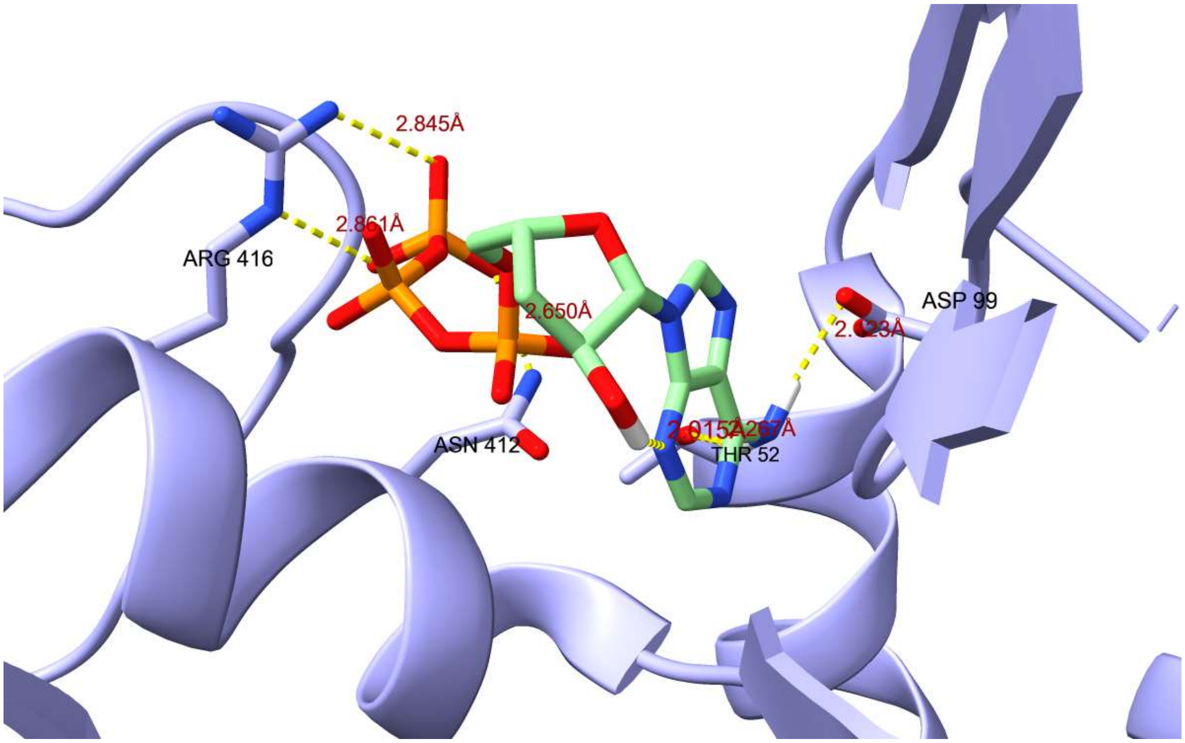

Figure S44. Predicted binding mode of COR-TP with sAC.

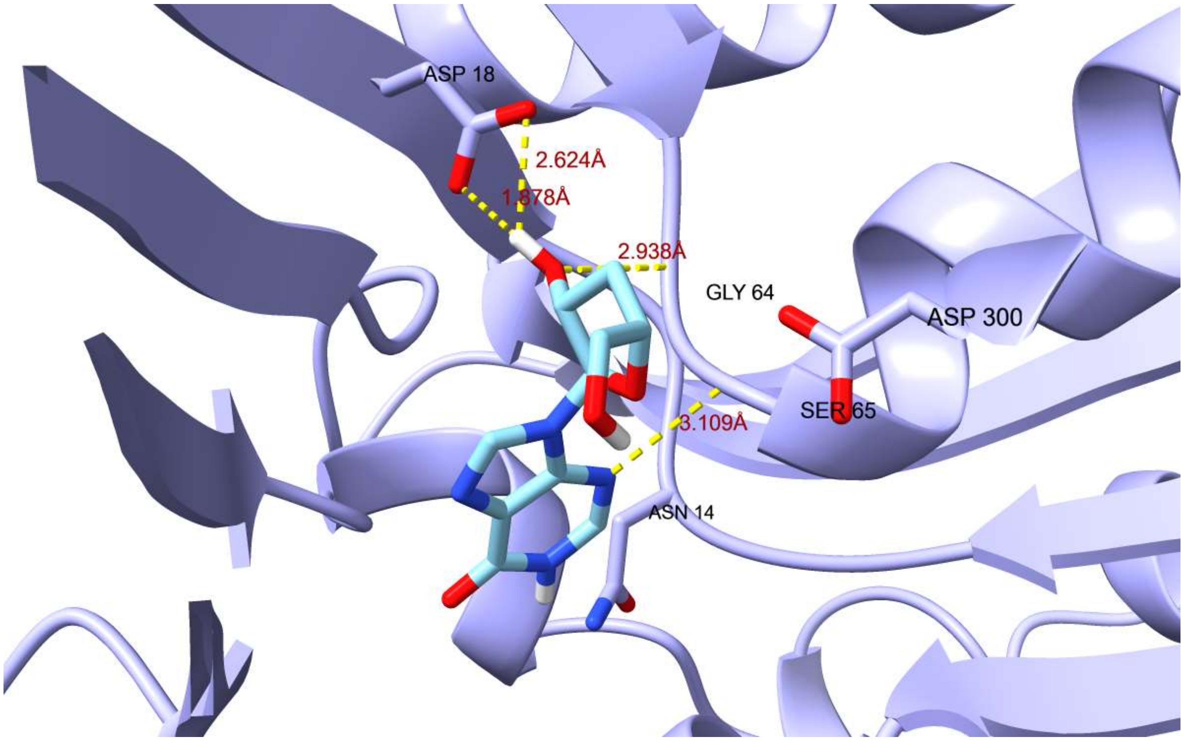

Figure S45. Predicted binding mode of 3’-dINO with ADK (site 1).

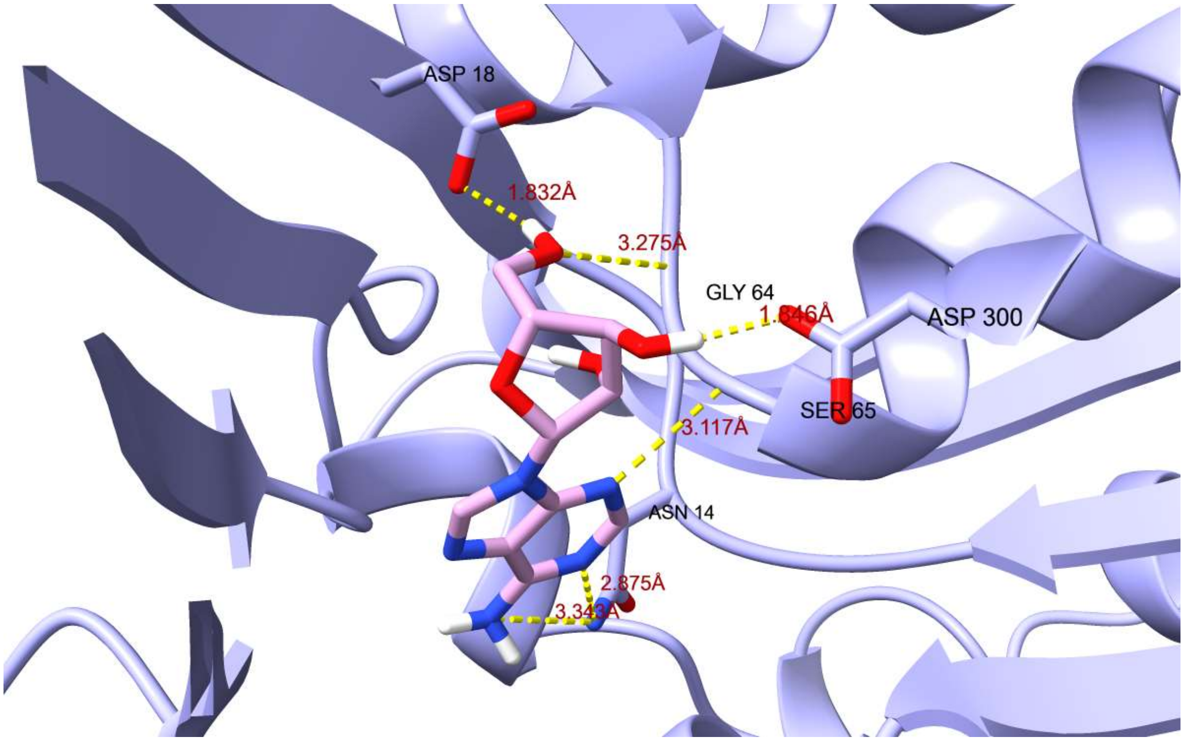

Figure S46. Predicted binding mode of ADO with ADK (site 1).

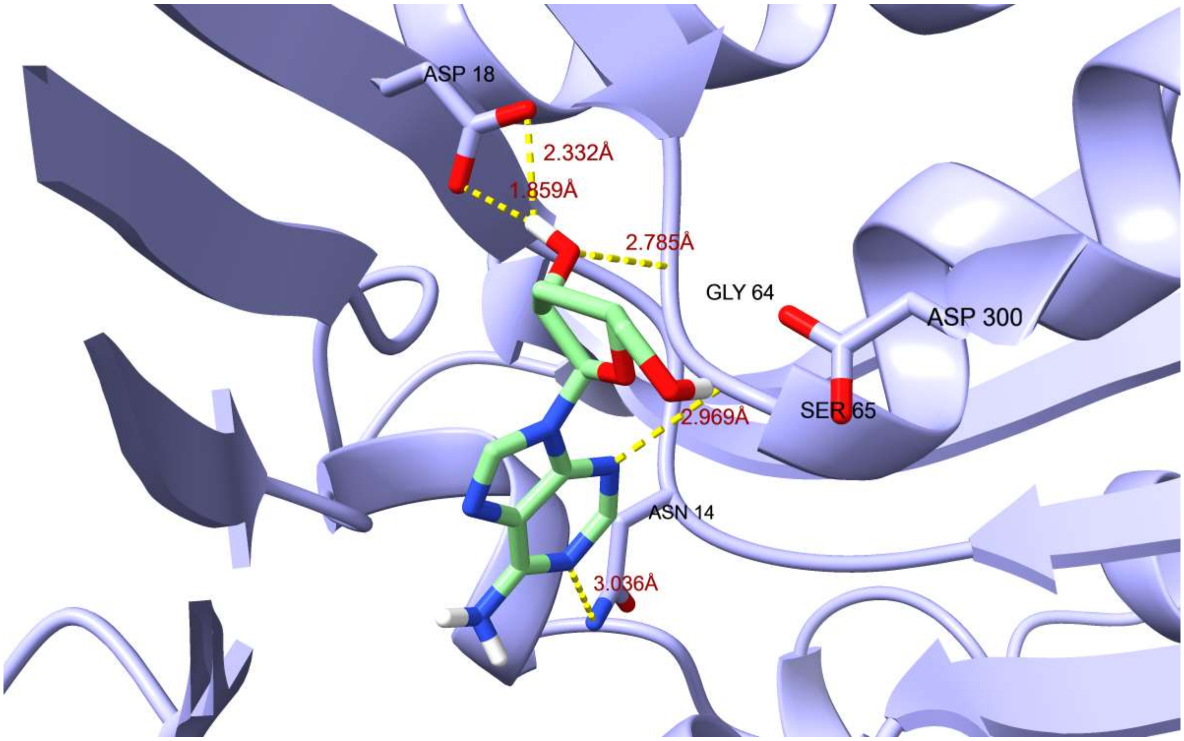

Figure S47. Predicted binding mode of COR with ADK (site 1).

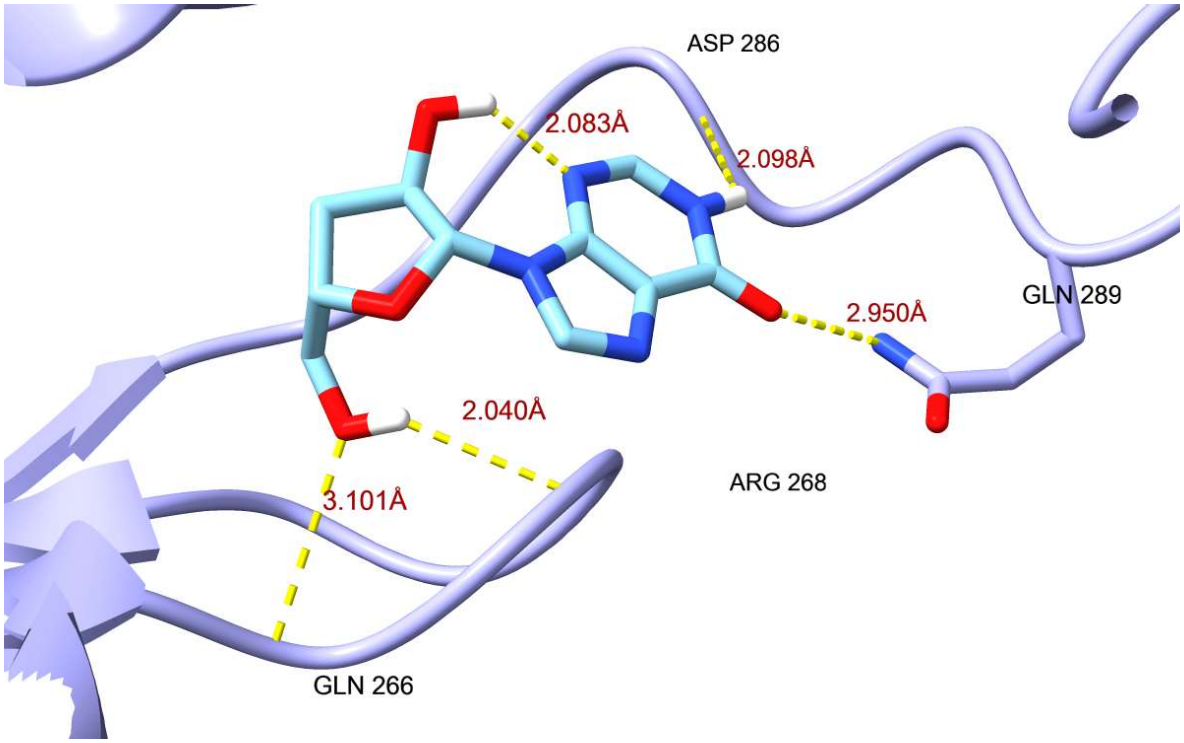

Figure S48. Predicted binding mode of 3’-dINO with ADK (site 2).

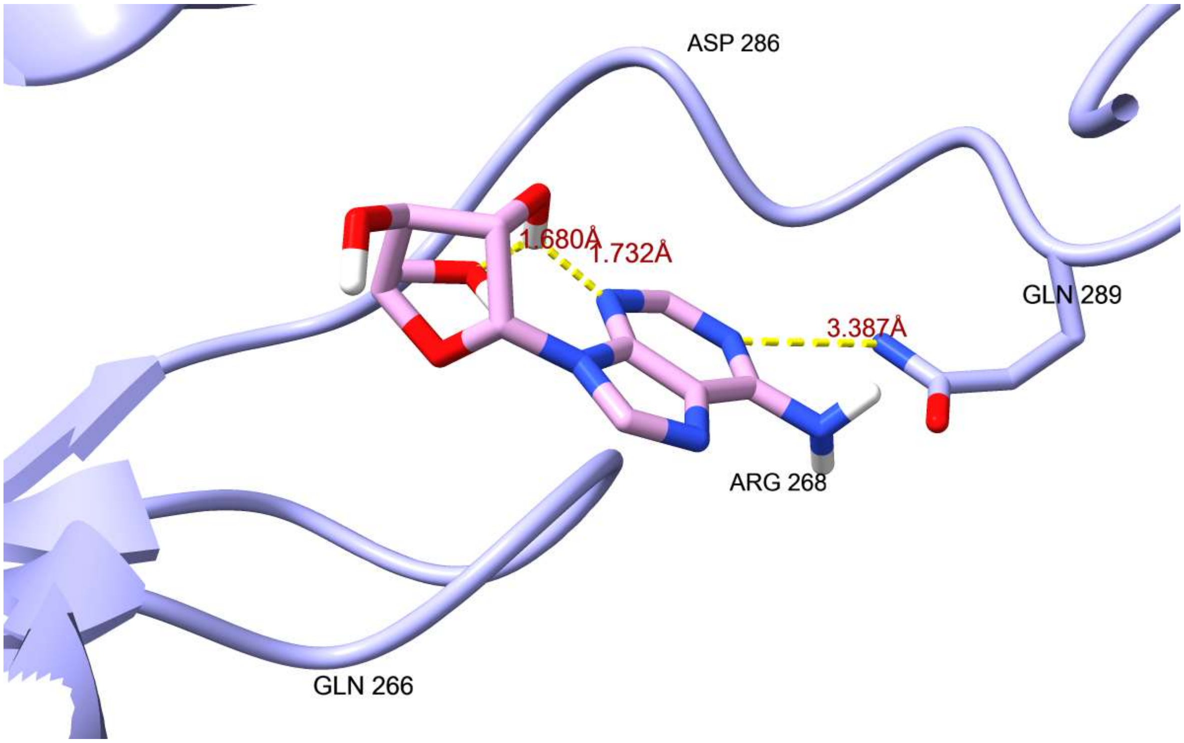

Figure S49. Predicted binding mode of ADO with ADK (site 2).

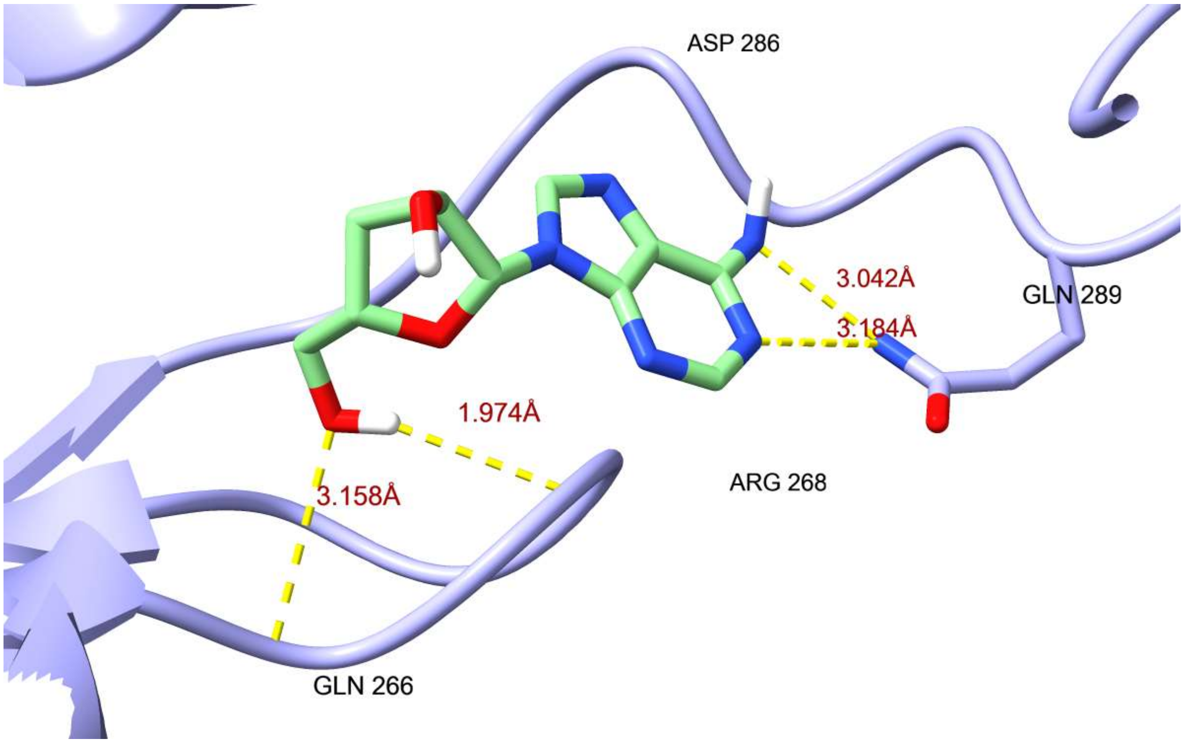

Figure S50. Predicted binding mode of COR with ADK (site 2).

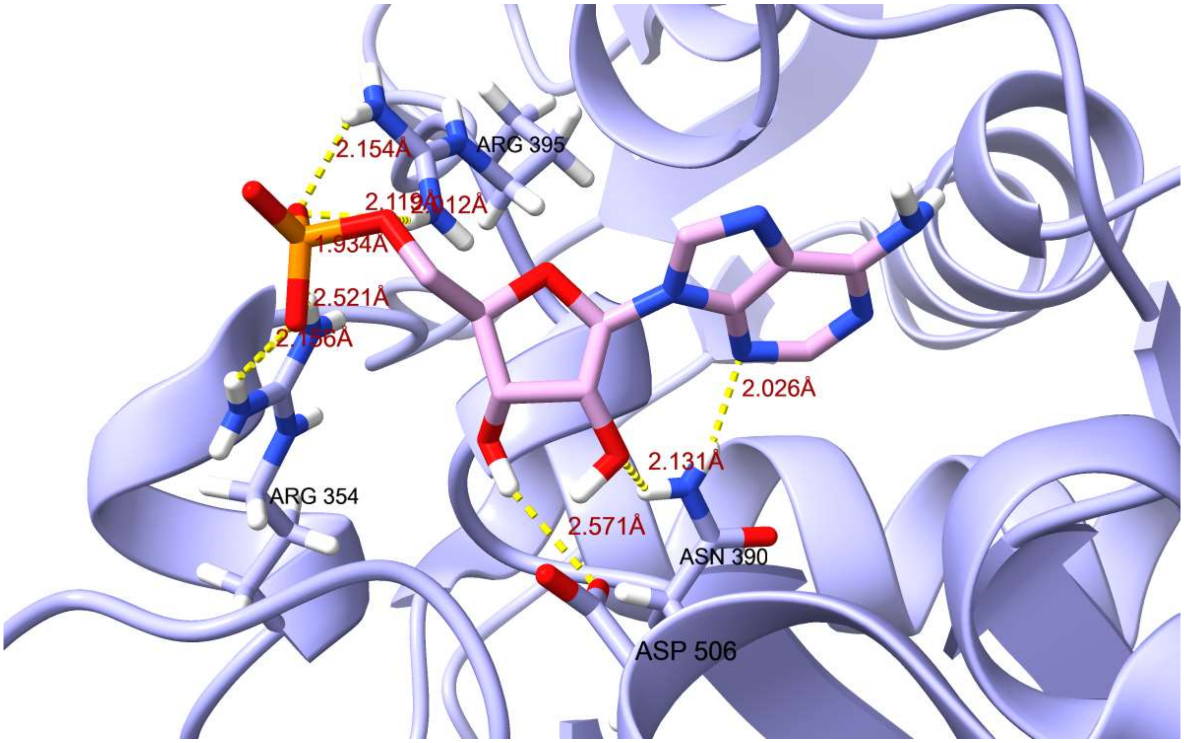

Figure S51. Predicted binding mode of AMP with CD73.

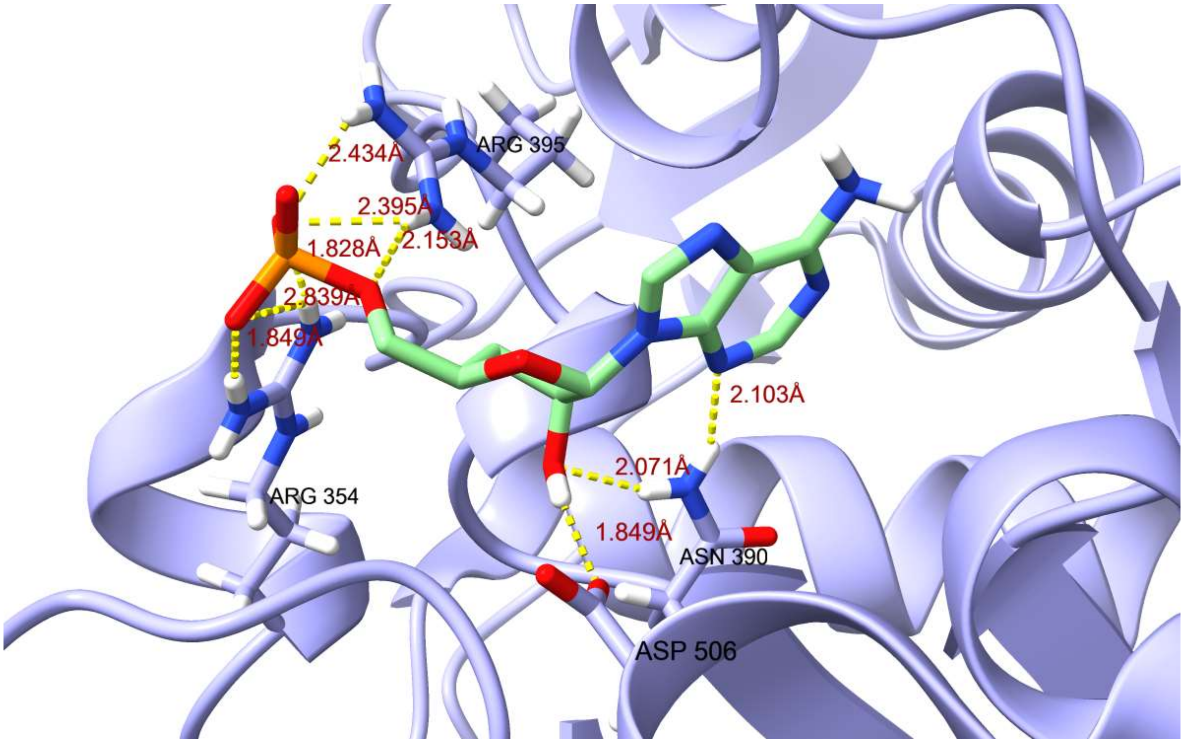

Figure S52. Predicted binding mode of COR-MP with CD73.

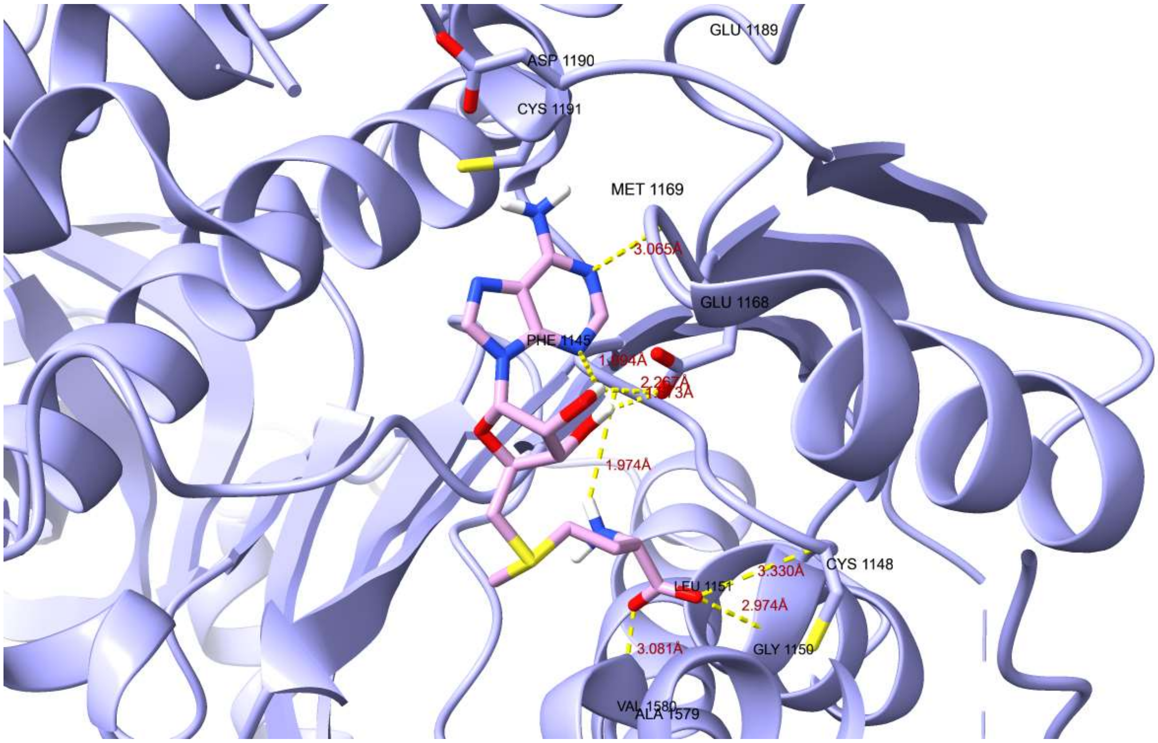

Figure S53. Predicted binding mode of SAM with DNMT1.

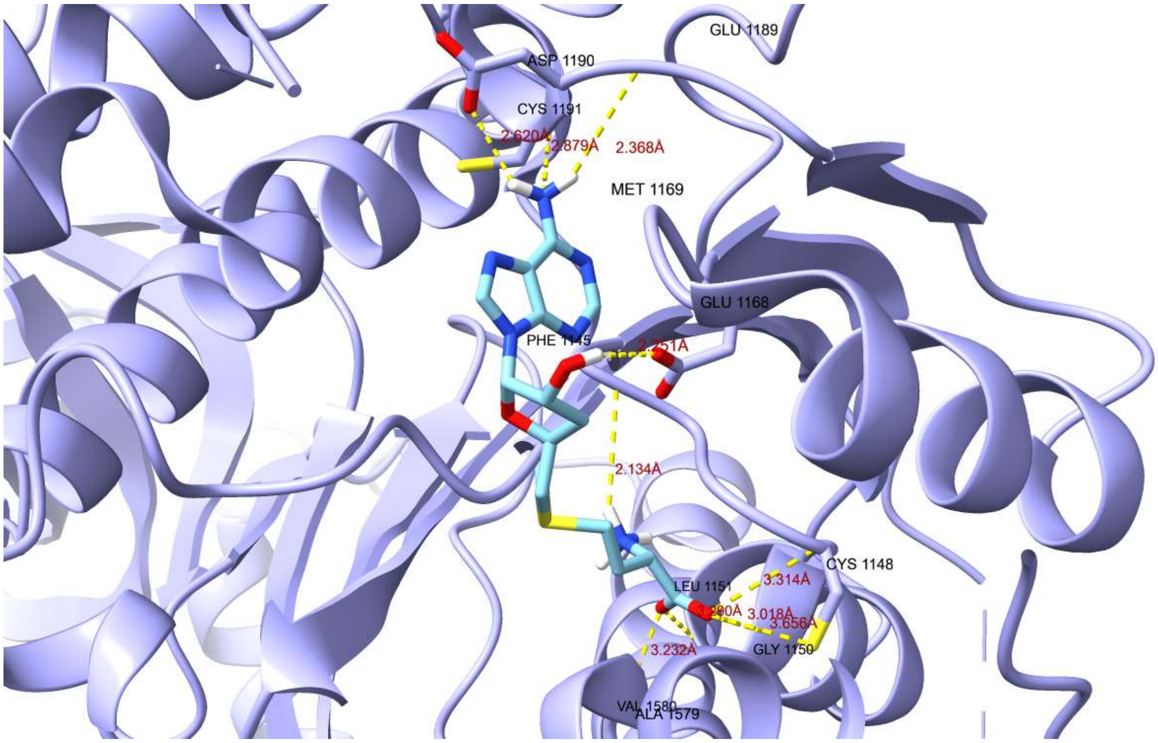

Figure S54. Predicted binding mode of 3’-dSAM with DNMT1.

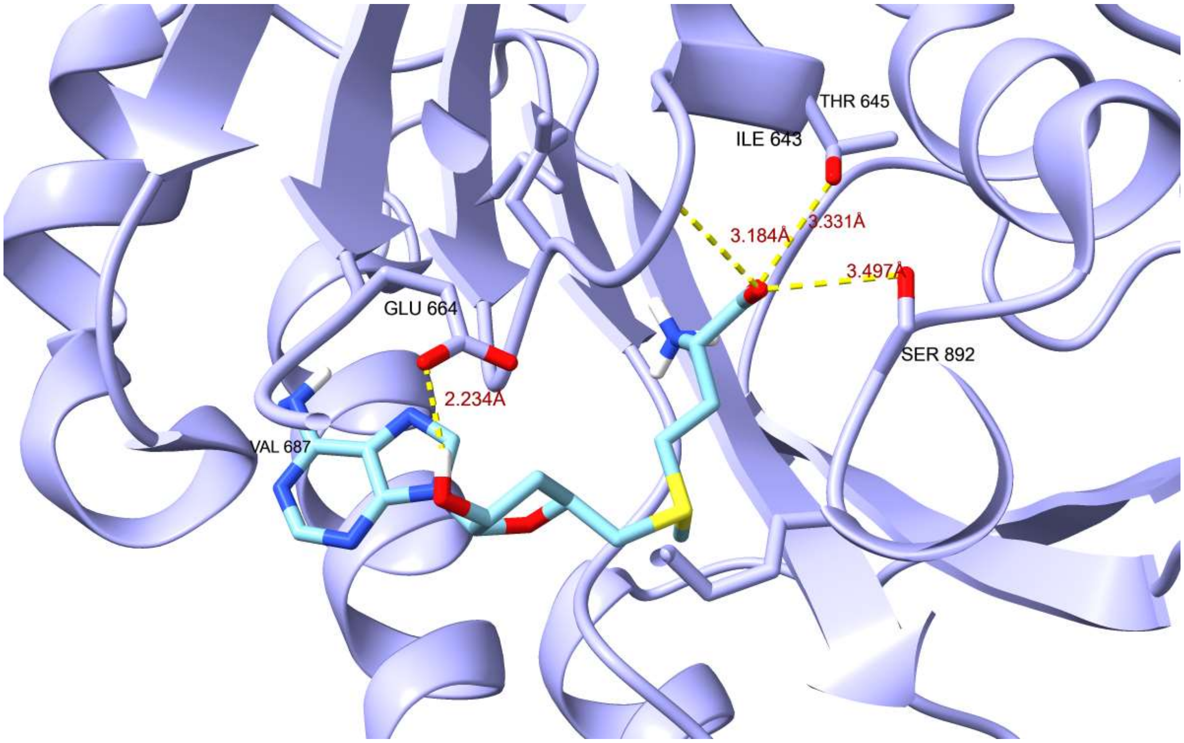

Figure S55. Predicted binding mode of SAM with DNMT3A.

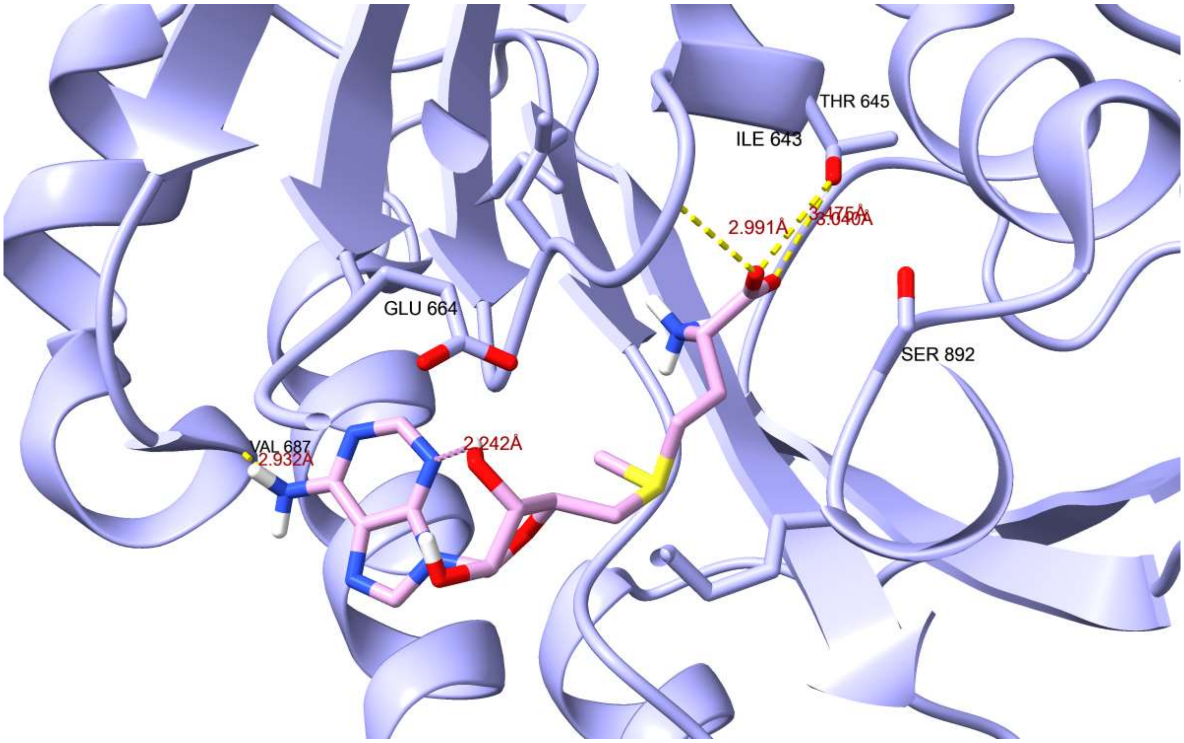

Figure S56. Predicted binding mode of 3’-dSAM with DNMT3A.

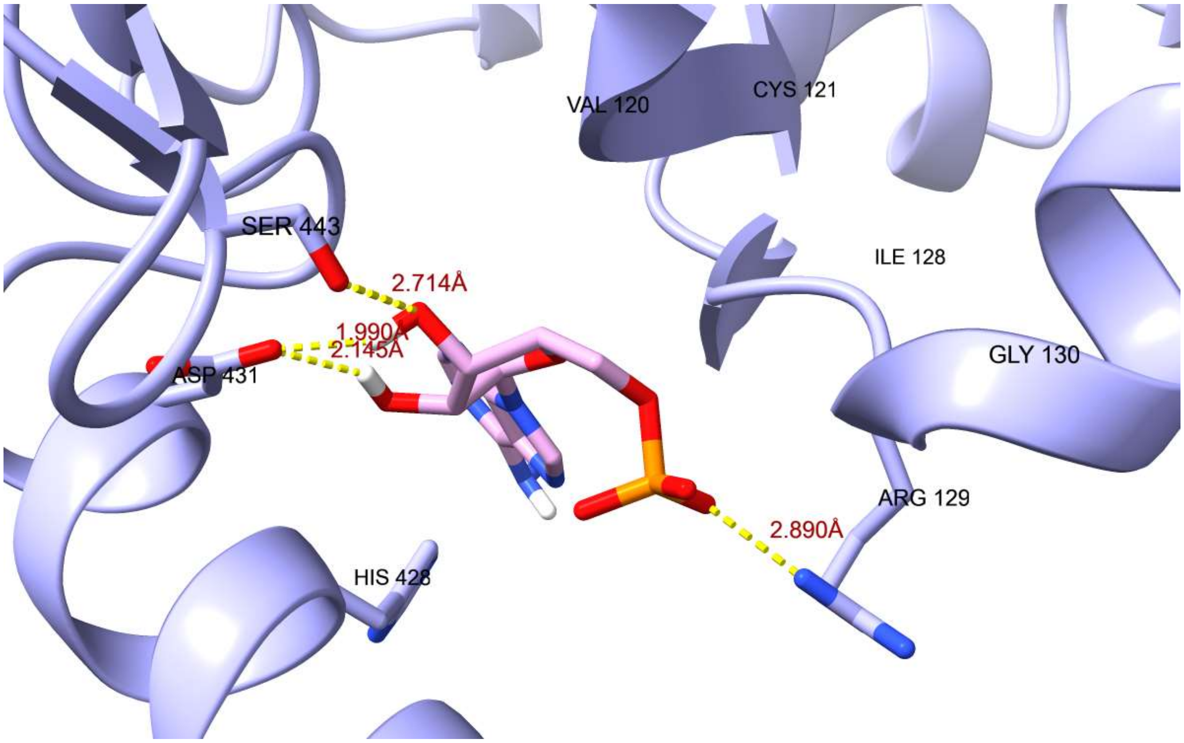

Figure S57. Predicted binding mode of AMP with NT5C2 (site 1).

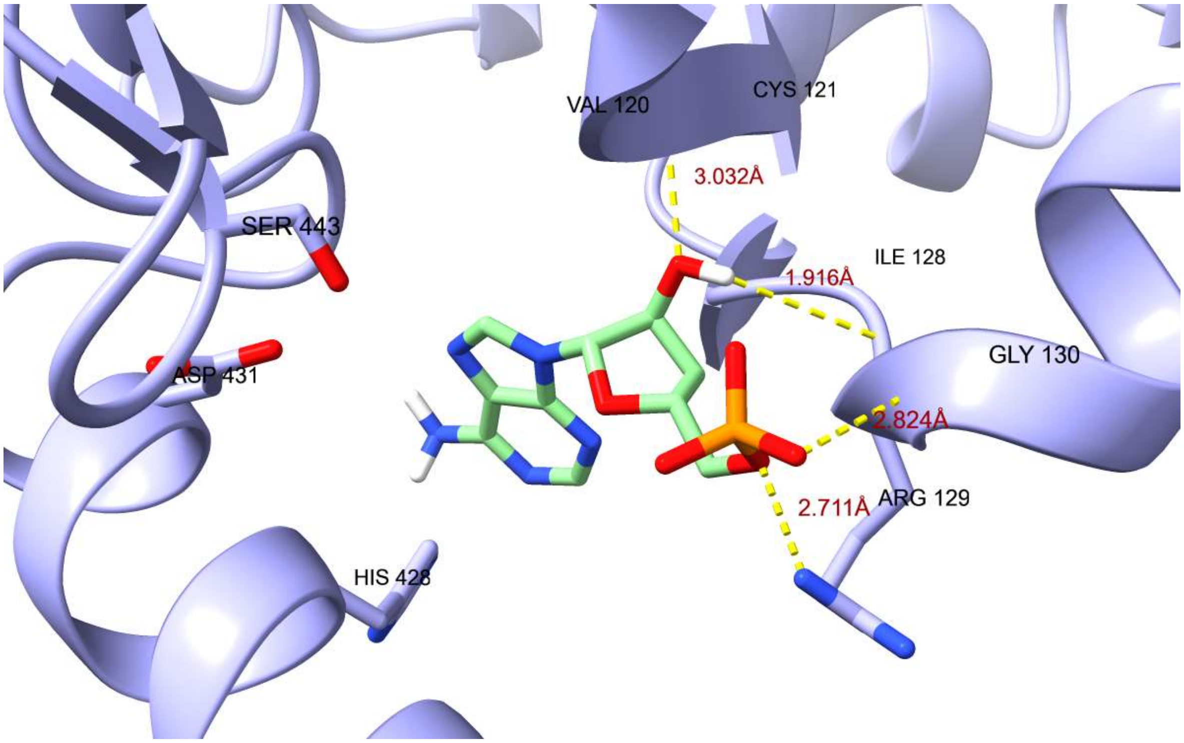

Figure S58. Predicted binding mode of COR-MP with NT5C2 (site 1).

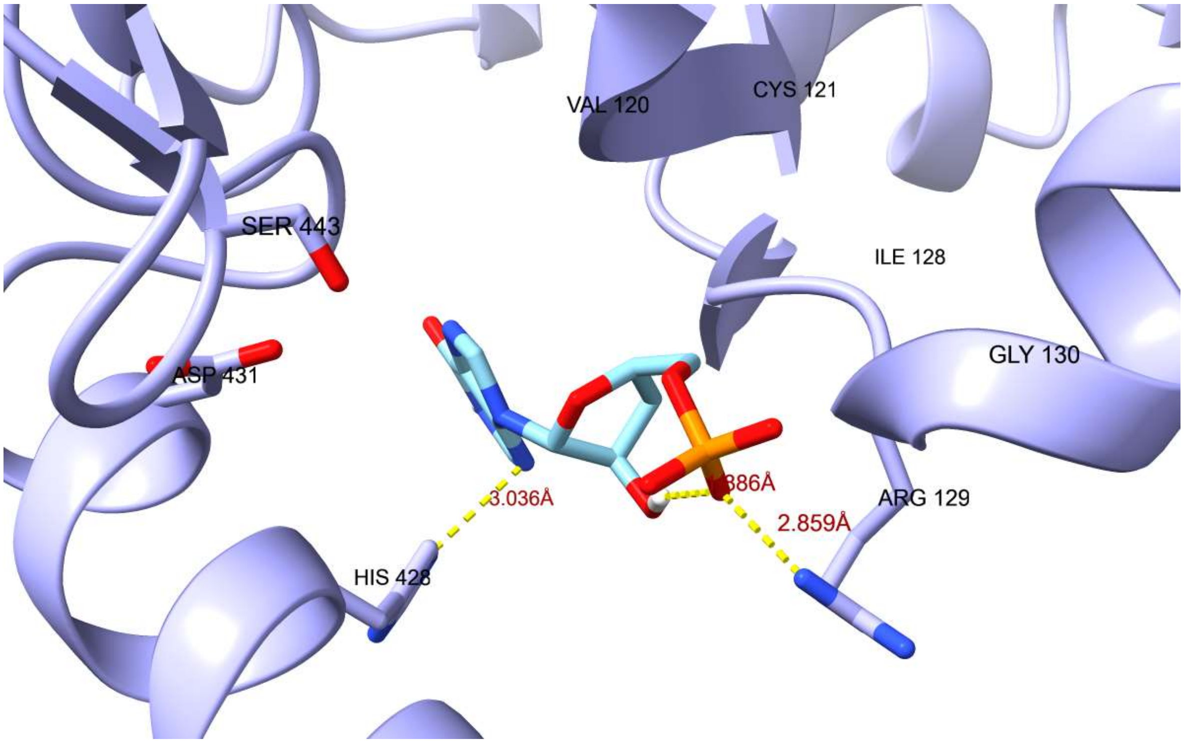

Figure S59. Predicted binding mode of 3’-dIMP with NT5C2 (site 1).

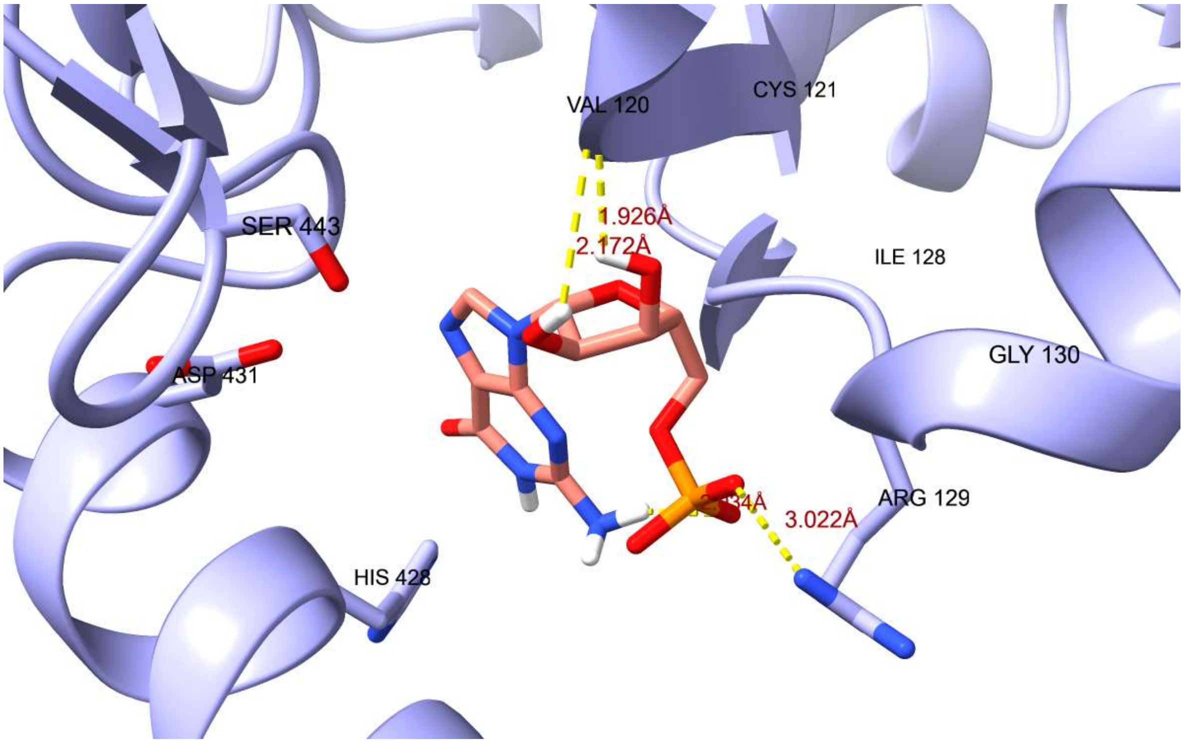

Figure S60. Predicted binding mode of GMP with NT5C2 (site 1).

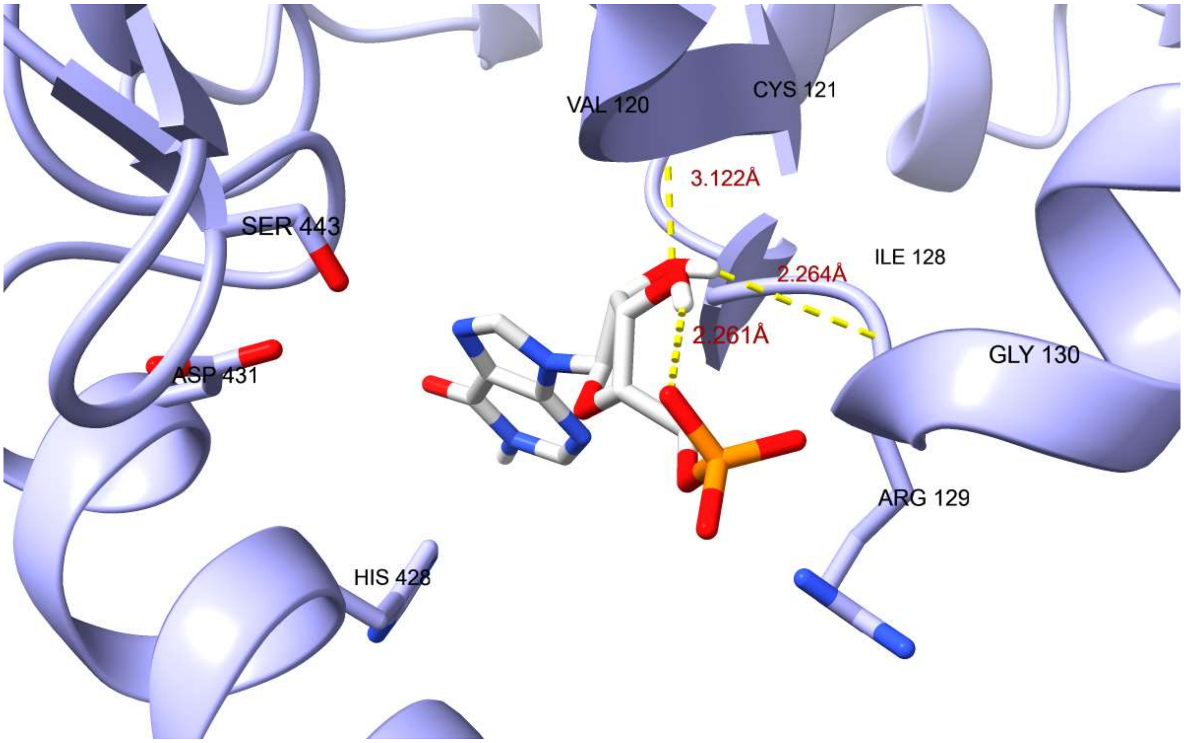

Figure S61. Predicted binding mode of IMP with NT5C2 (site 1).

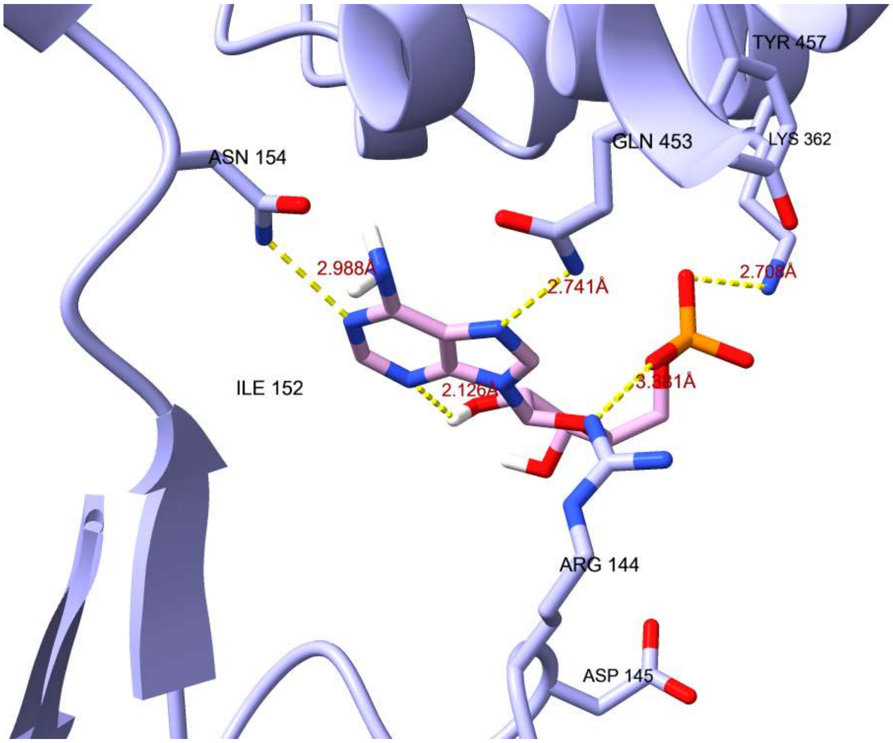

Figure S62. Predicted binding mode of AMP with NT5C2 (site 2).

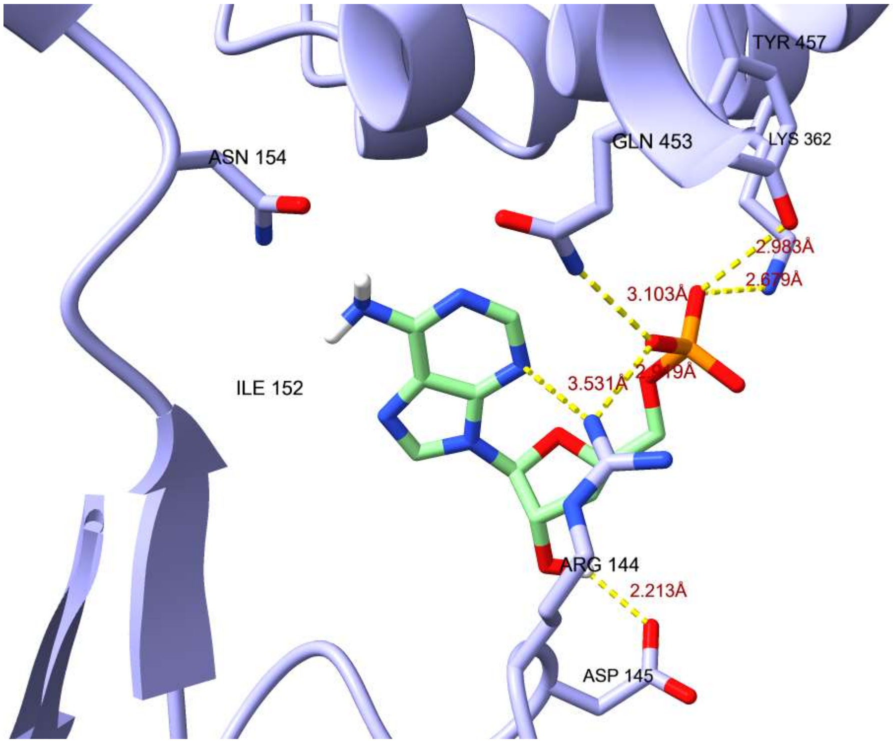

Figure S63. Predicted binding mode of COR-MP with NT5C2 (site 2).

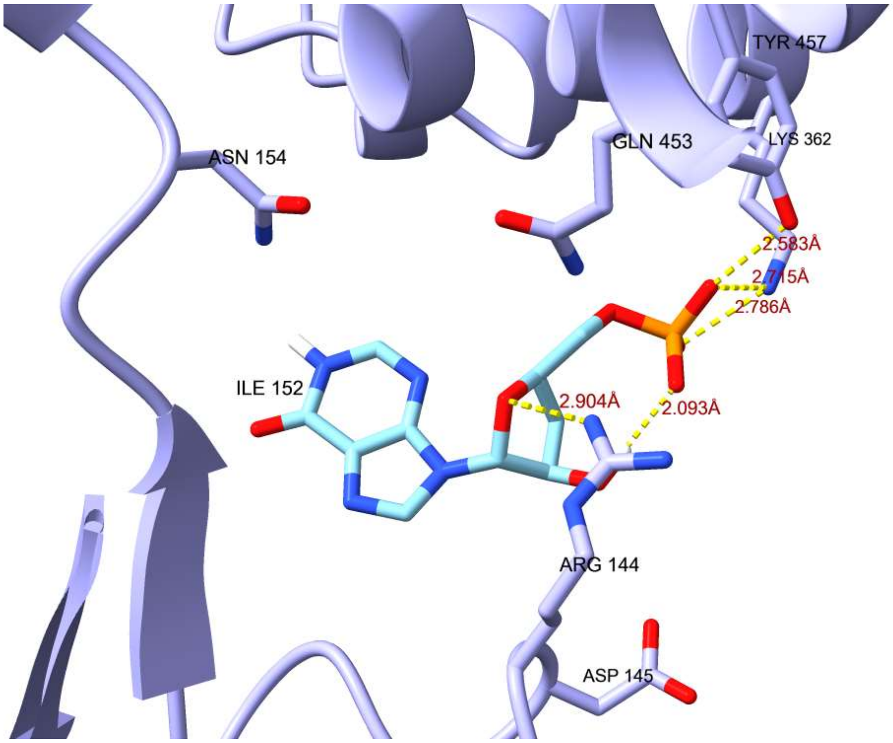

Figure S64. Predicted binding mode of 3’-dIMP with NT5C2 (site 2).

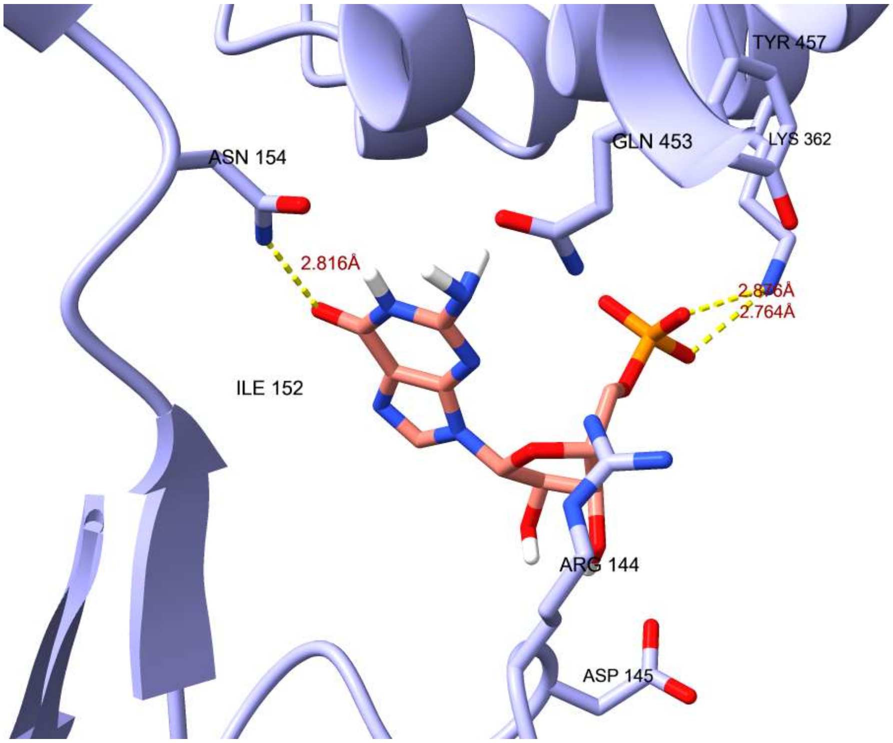

Figure S65. Predicted binding mode of GMP with NT5C2 (site 2).

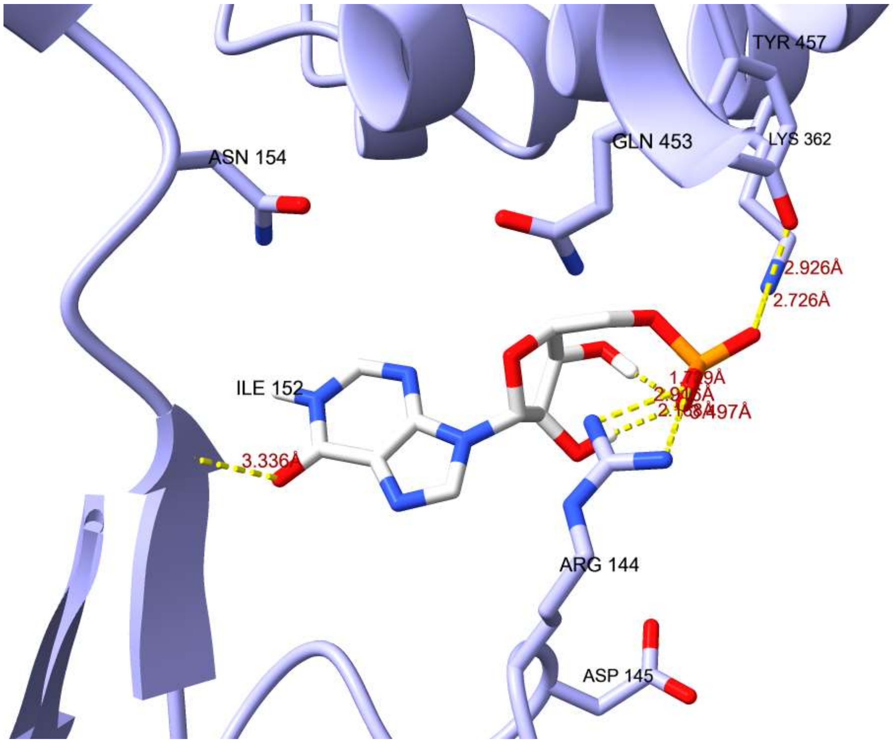

Figure S66. Predicted binding mode of IMP with NT5C2 (site 2).

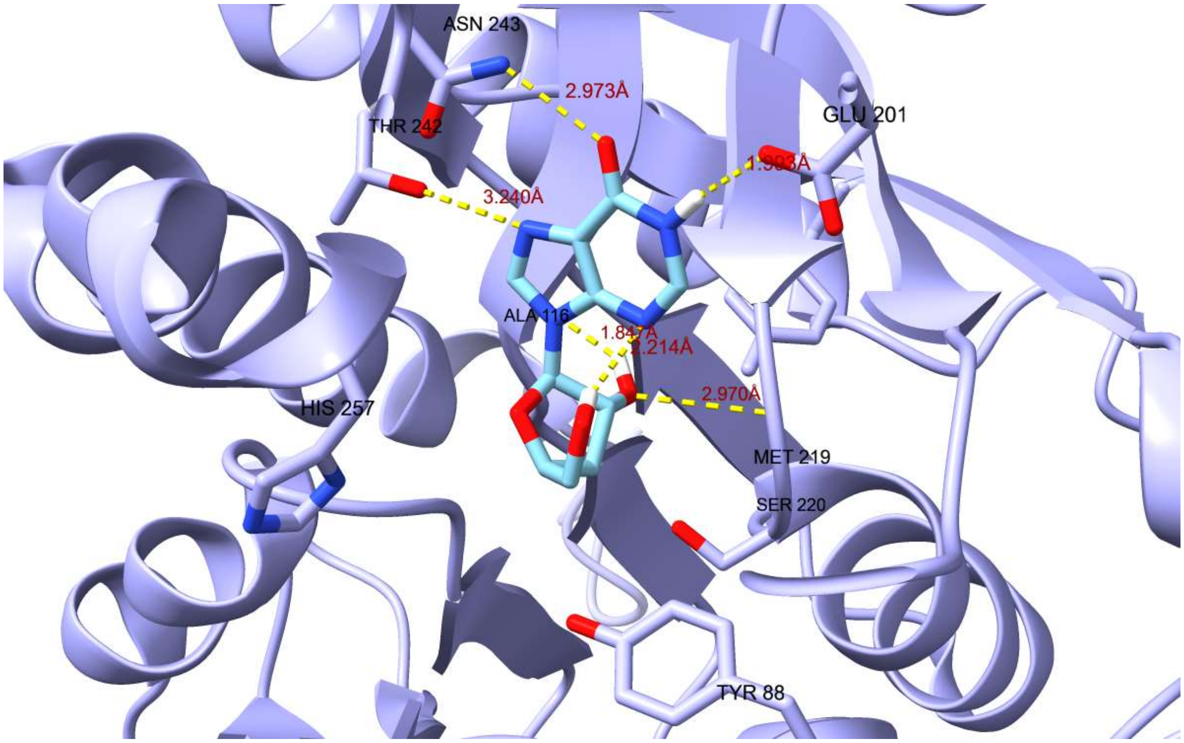

Figure S67. Predicted binding mode of 3’-dINO with PNP.

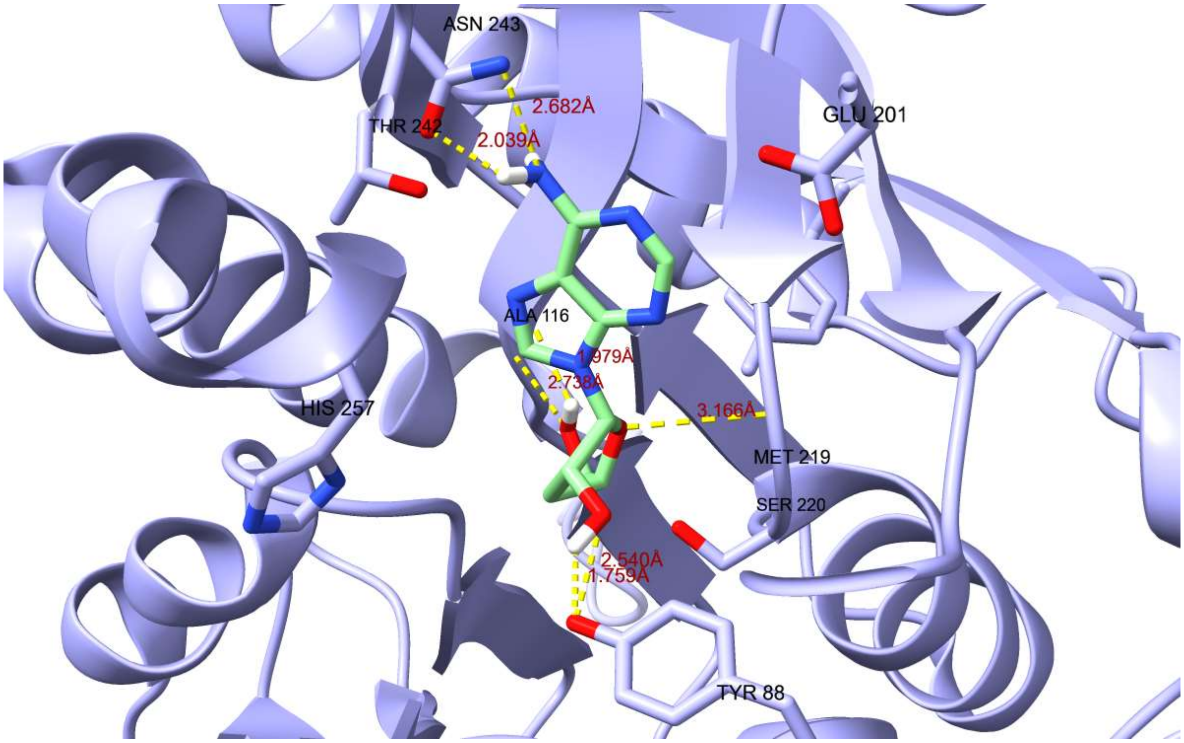

Figure S68. Predicted binding mode of COR with PNP.

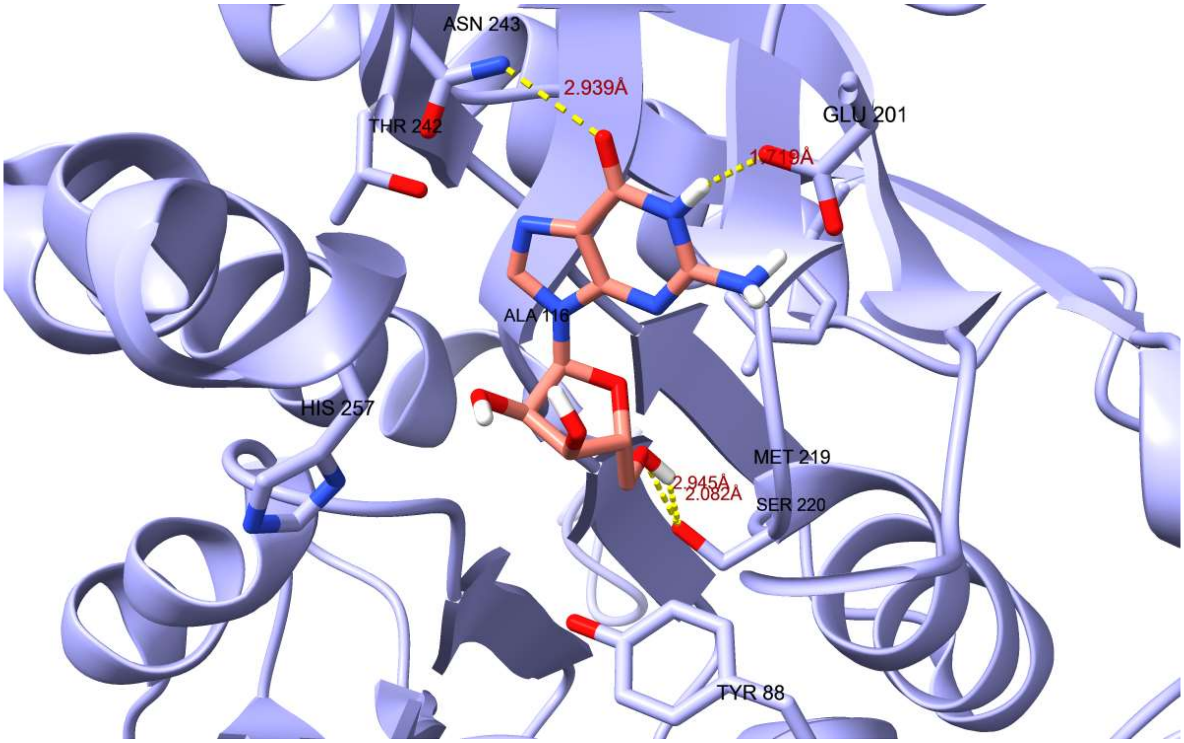

Figure S69. Predicted binding mode of GUA with PNP.

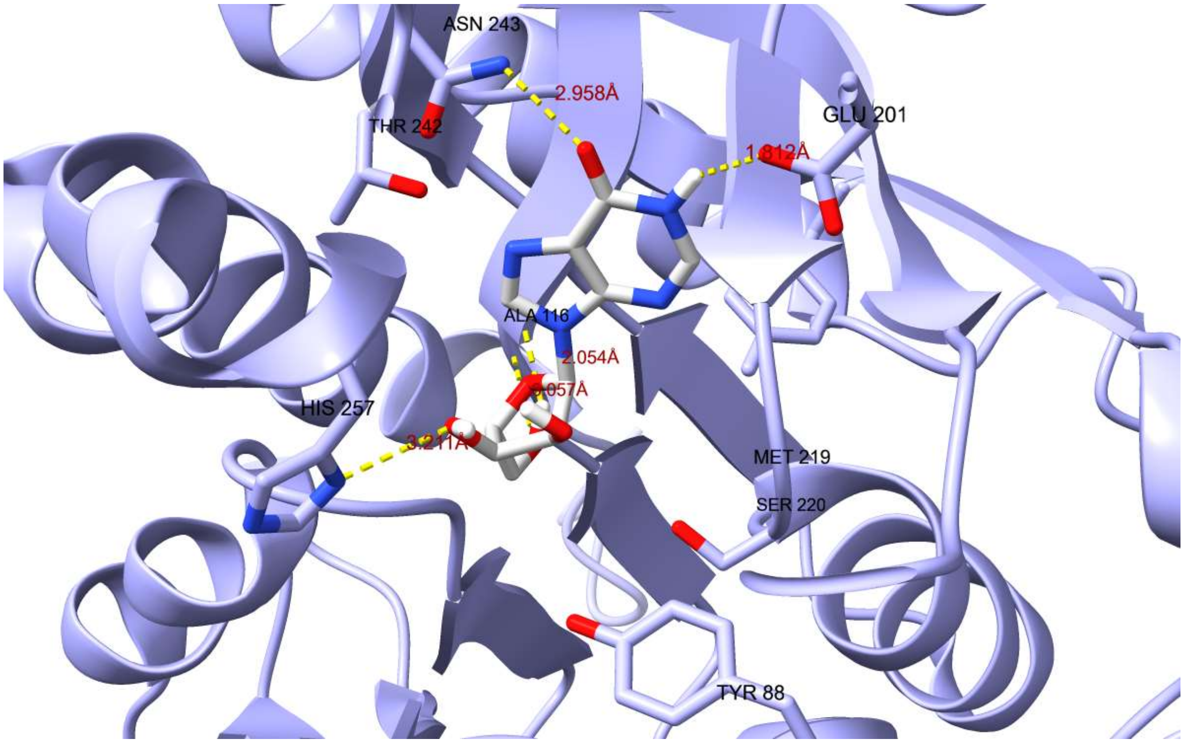

Figure S70. Predicted binding mode of INO with PNP.

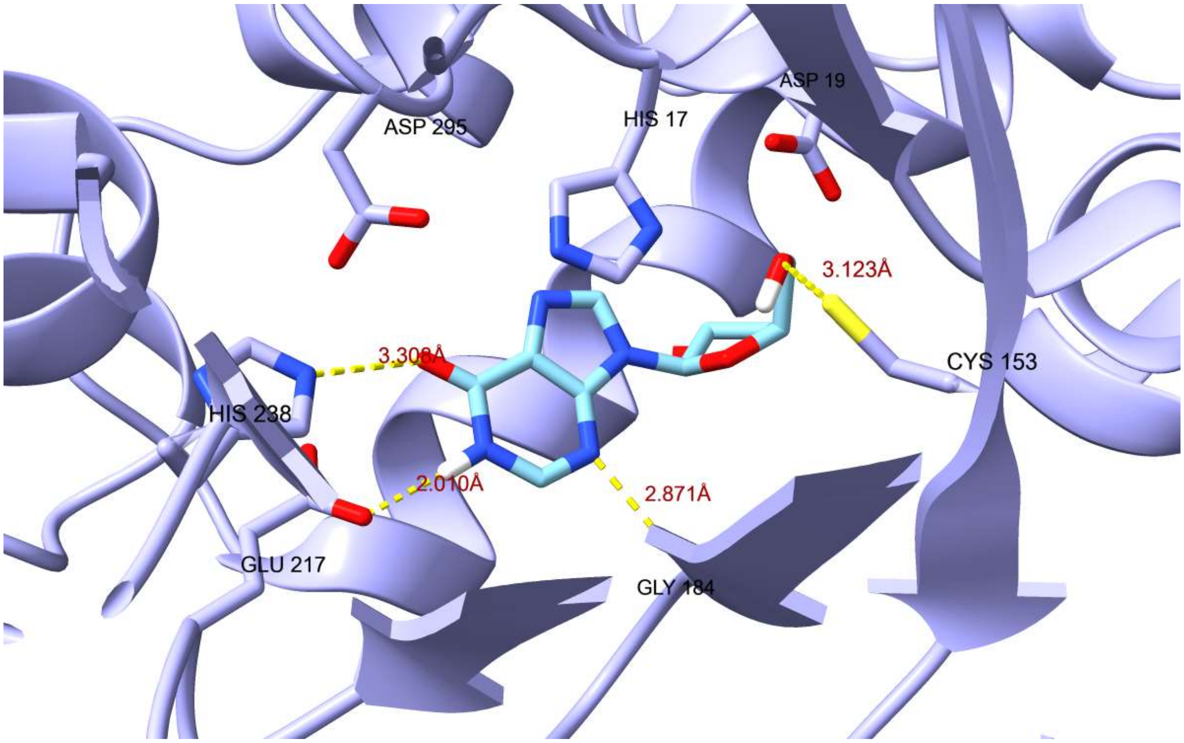

Figure S71. Predicted binding mode of 3'-dINO with ADA.

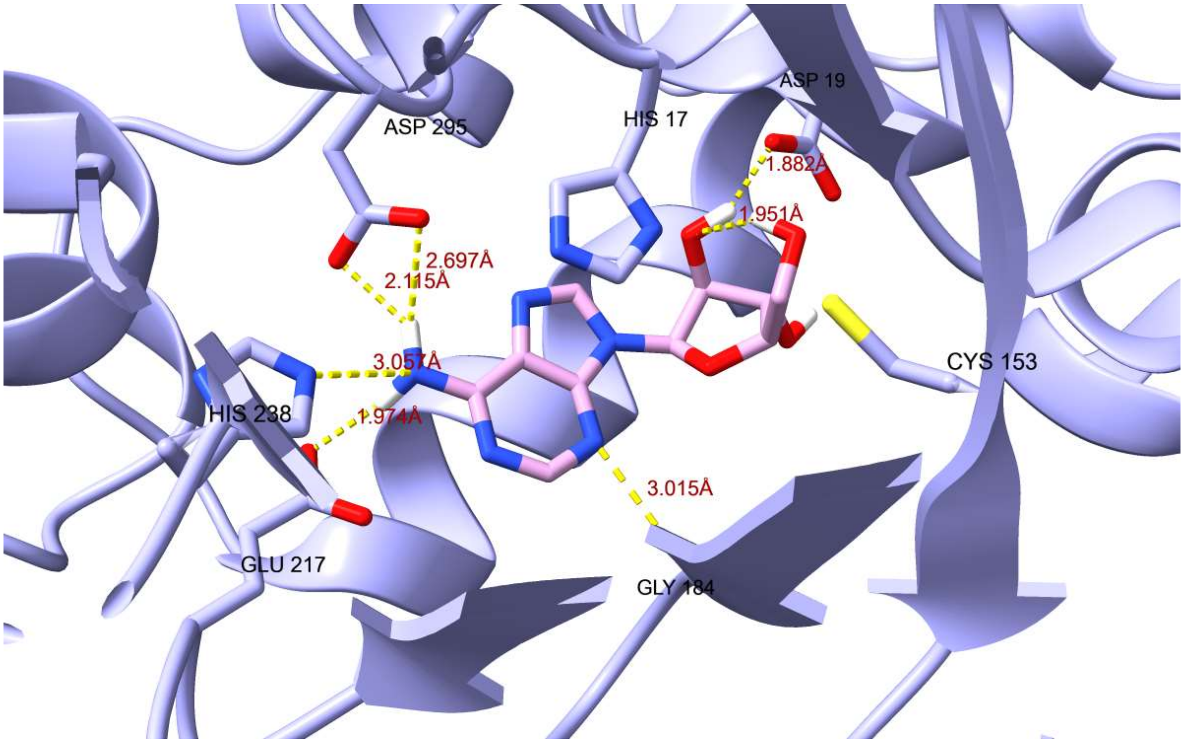

Figure S72. Predicted binding mode of ADO with ADA.

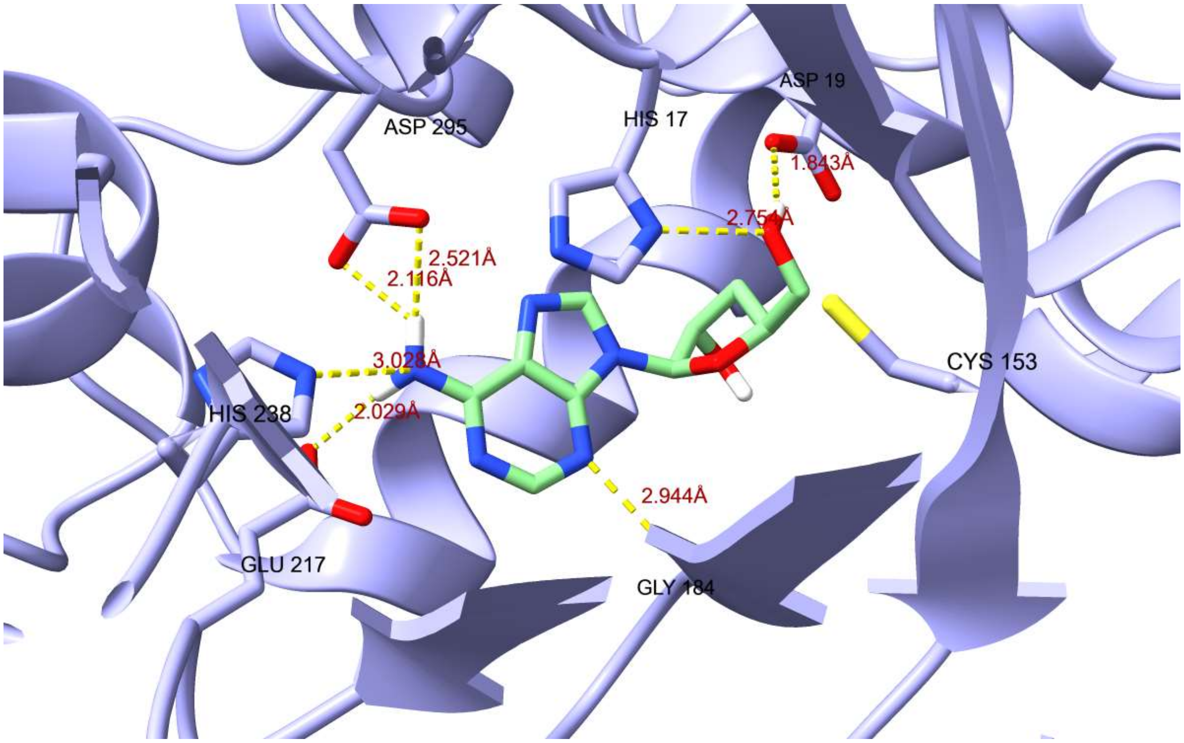

Figure S73. Predicted binding mode of COR with ADA.

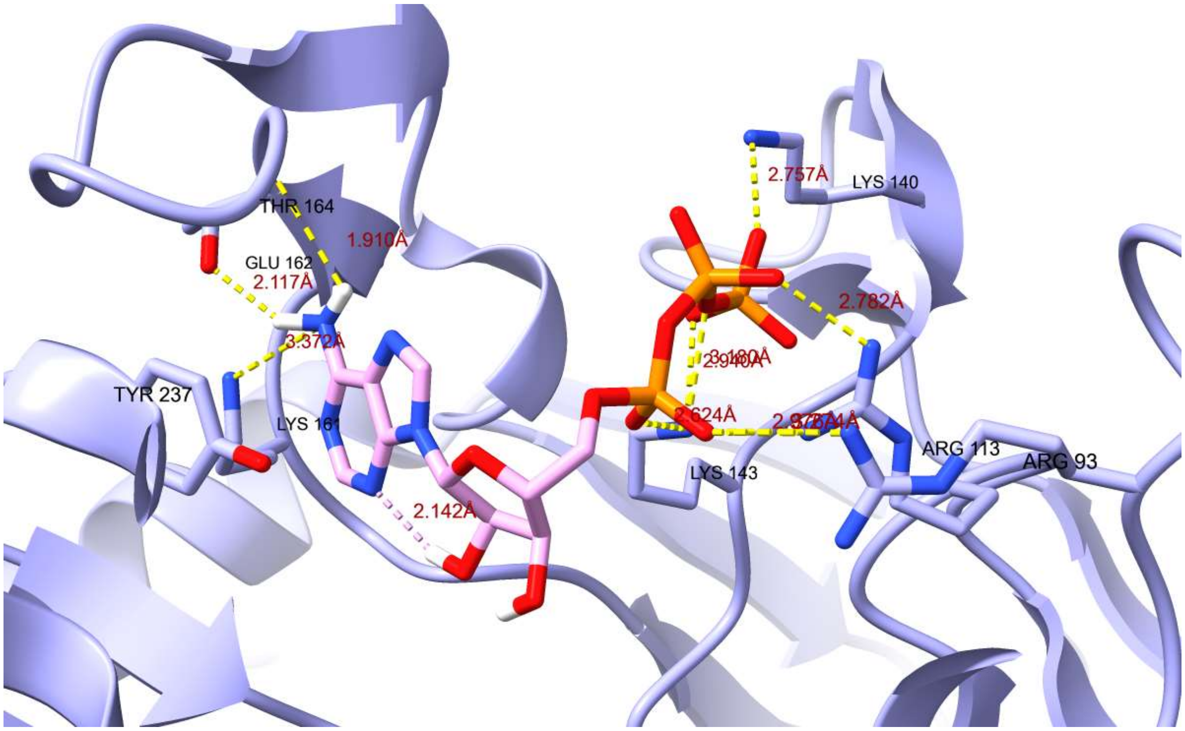

Figure S74. Predicted binding mode of ATP with CD39.

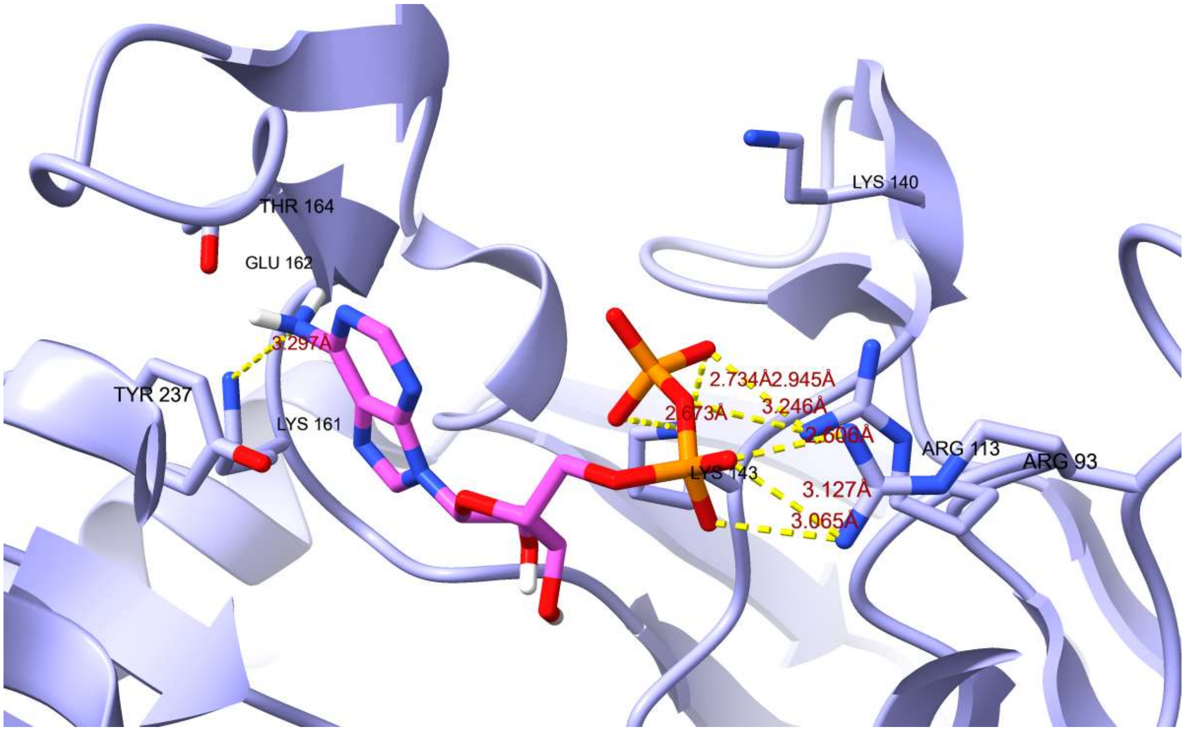

Figure S75. Predicted binding mode of ADP with CD39.

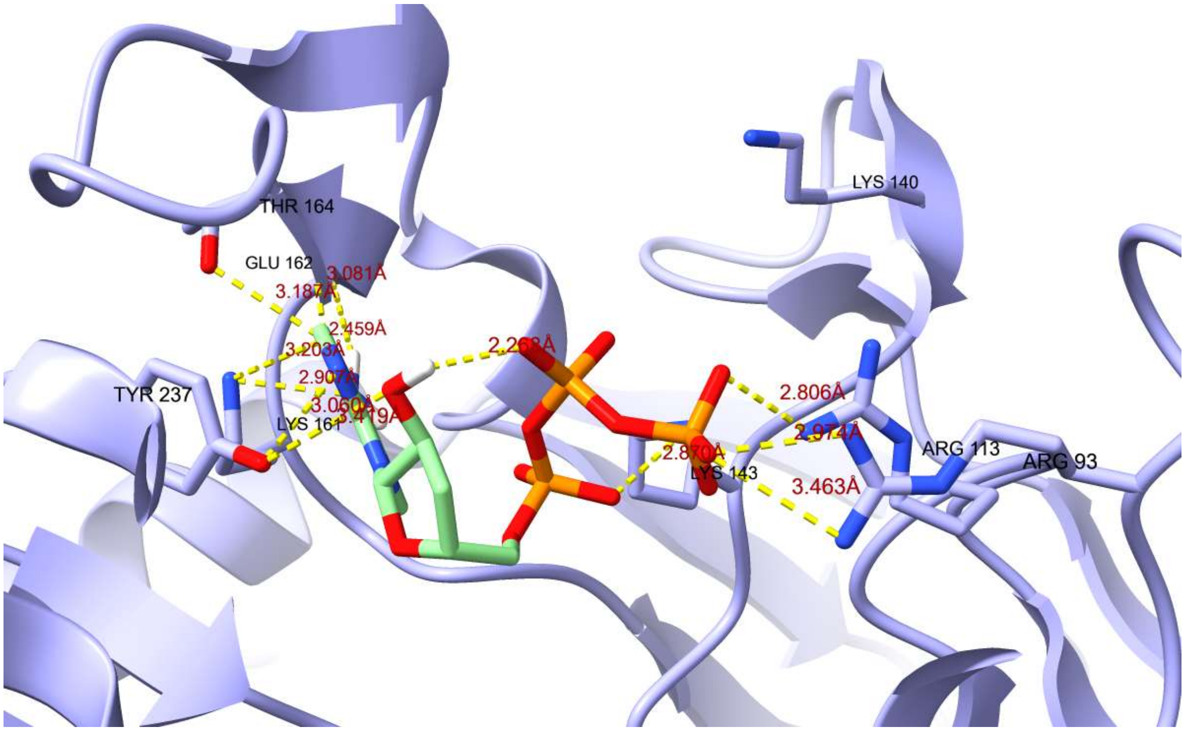

Figure S76. Predicted binding mode of COR-TP with CD39.

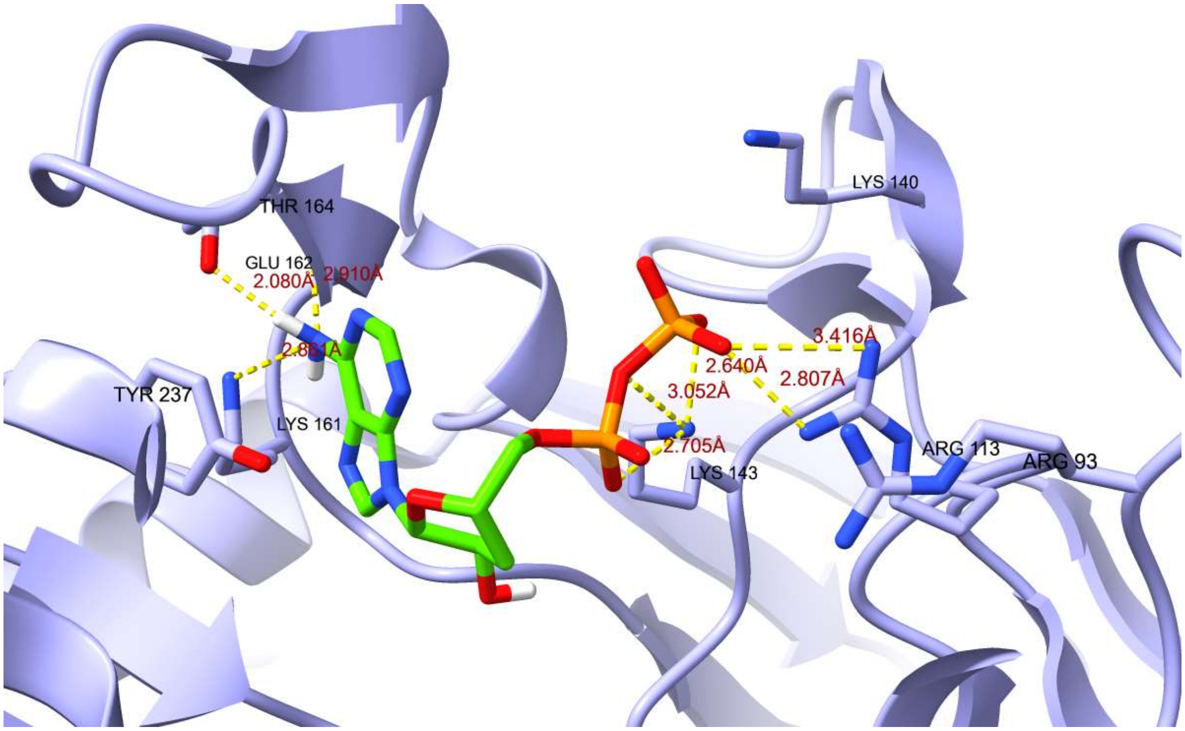

Figure S77. Predicted binding mode of COR-DP with CD39.

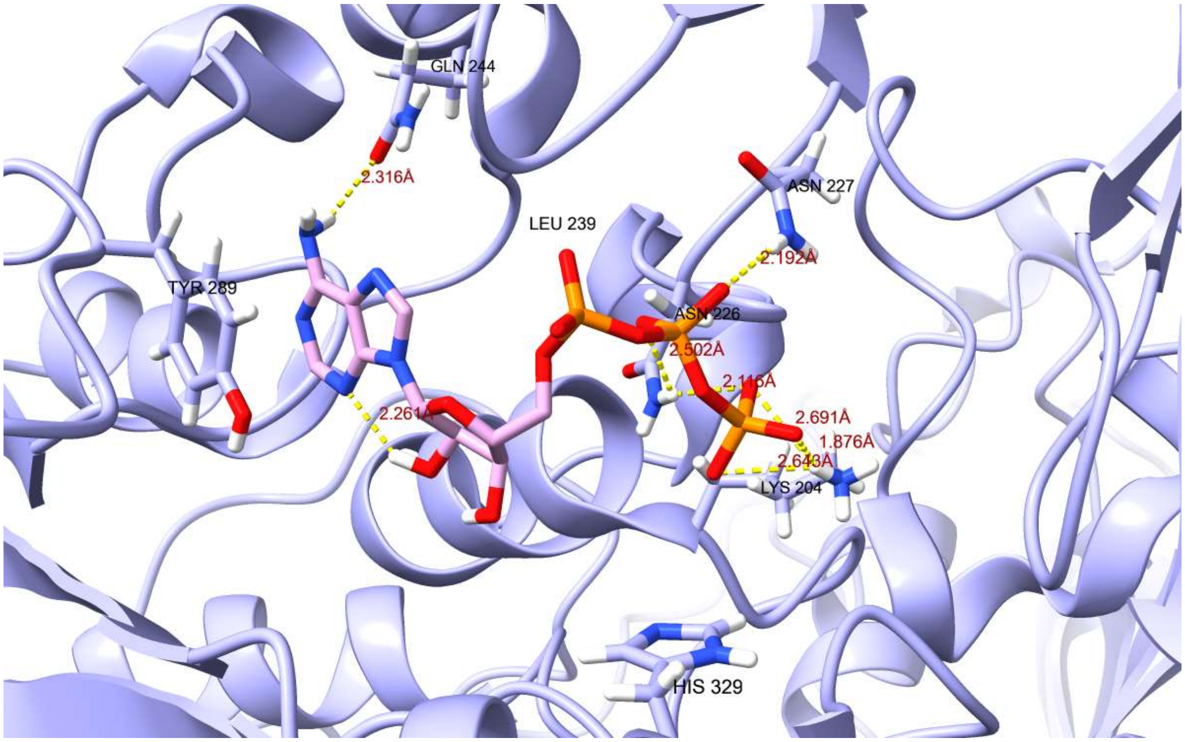

Figure S78. Predicted binding mode of ATP with ENPP3.

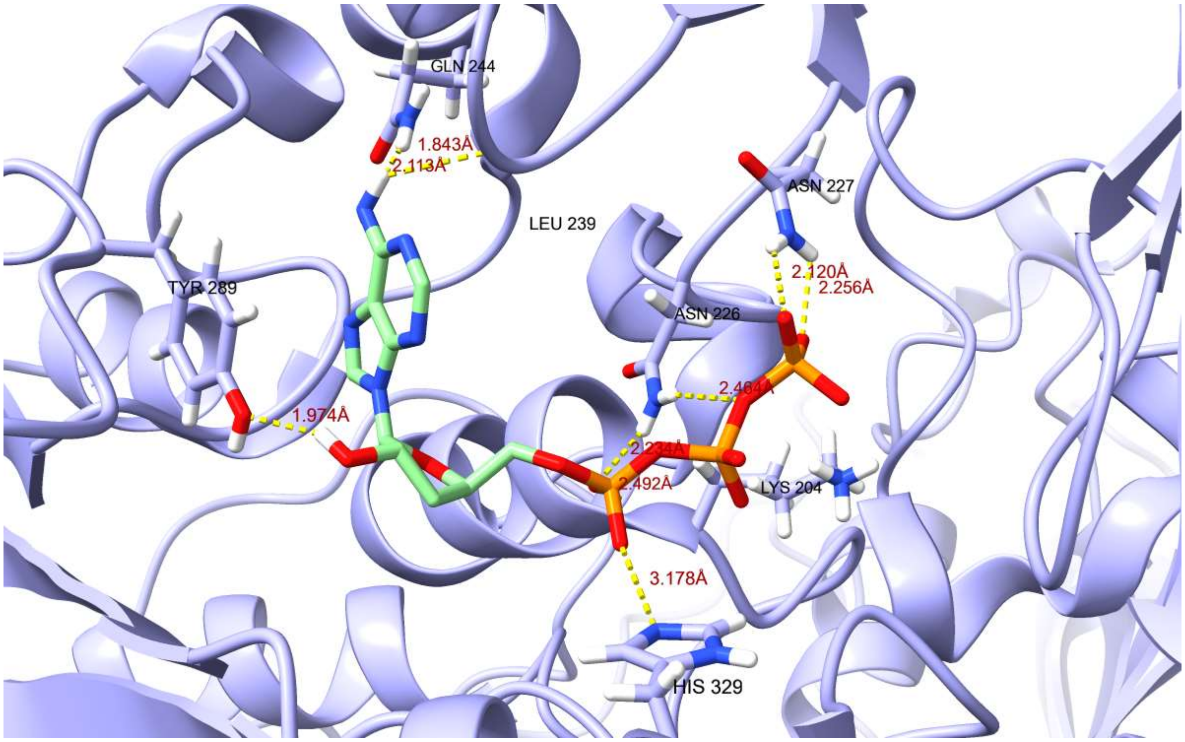

Figure S79. Predicted binding mode of COR-TP with ENPP3.

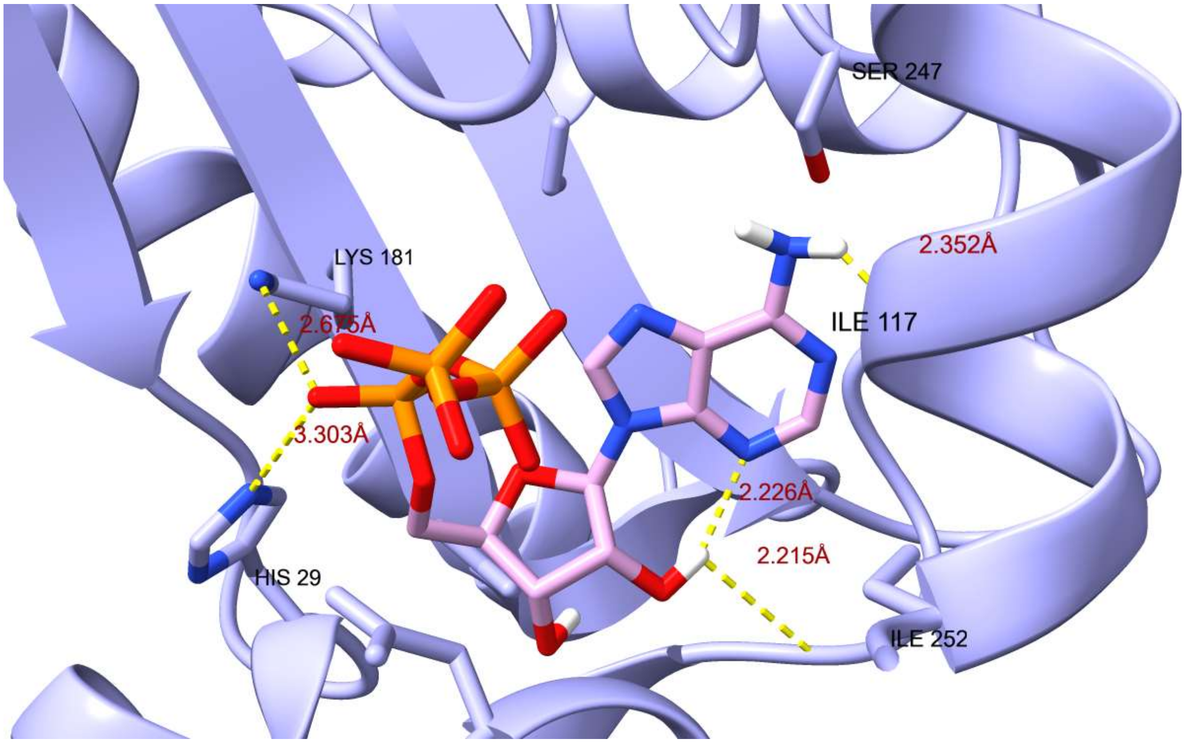

Figure S80. Predicted binding mode of ATP with MAT.

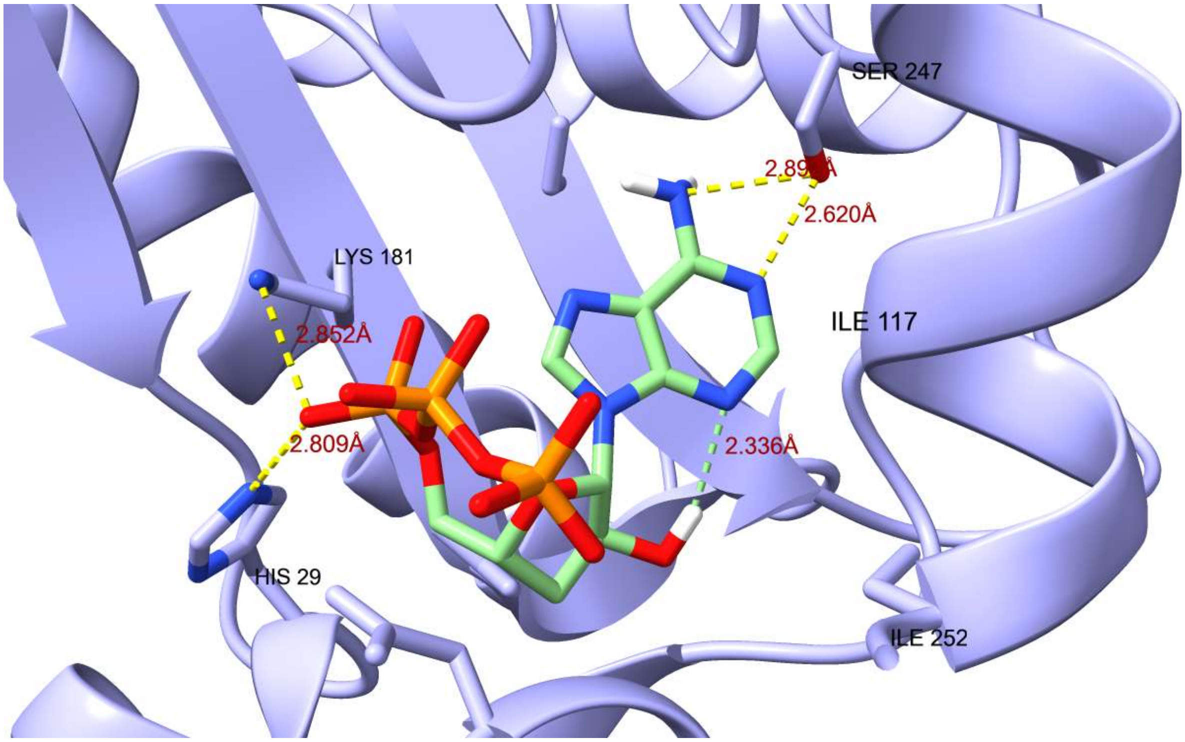

Figure S81. Predicted binding mode of COR-TP with MAT.

Binding modes of biguanides and candidate metabolites in the docked targets according to AutoDock Vina

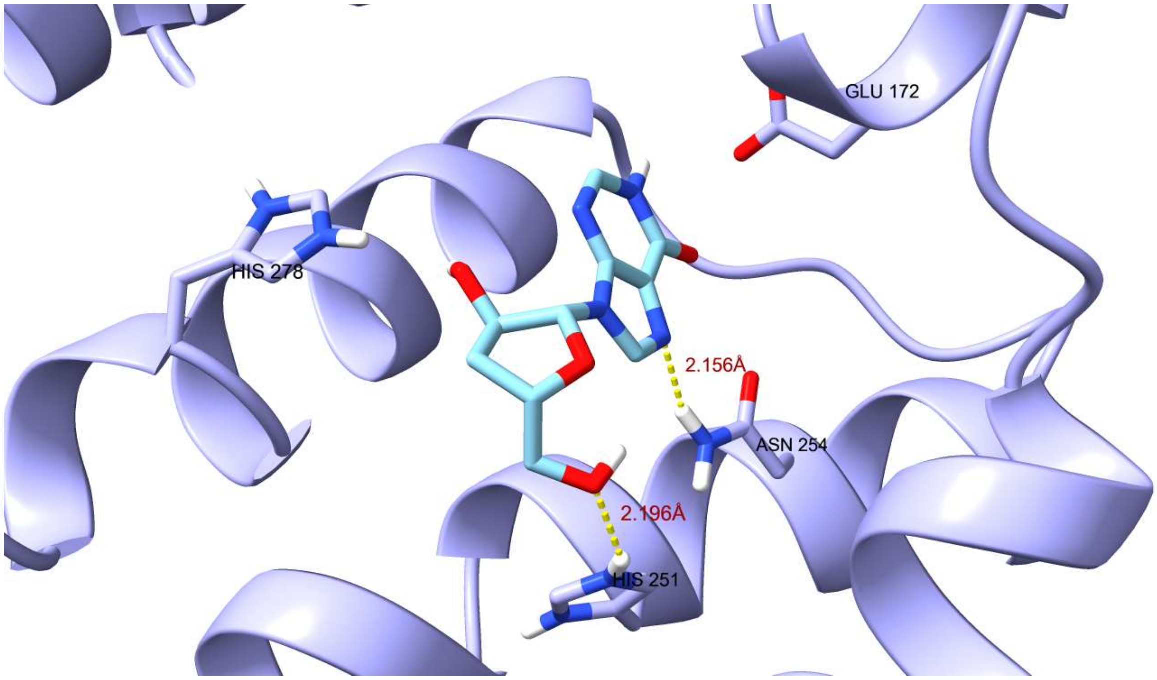

Figure S82. Predicted binding mode of 3’-dINO with A_1R.

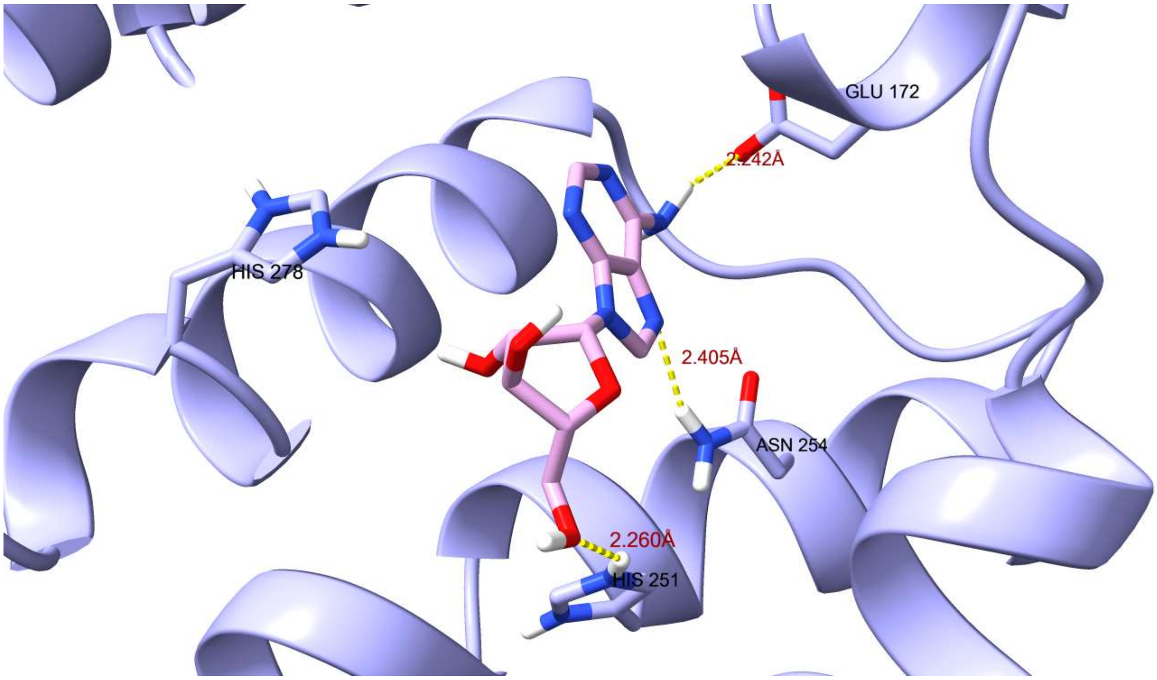

Figure S83. Predicted binding mode of ADO with A_1R.

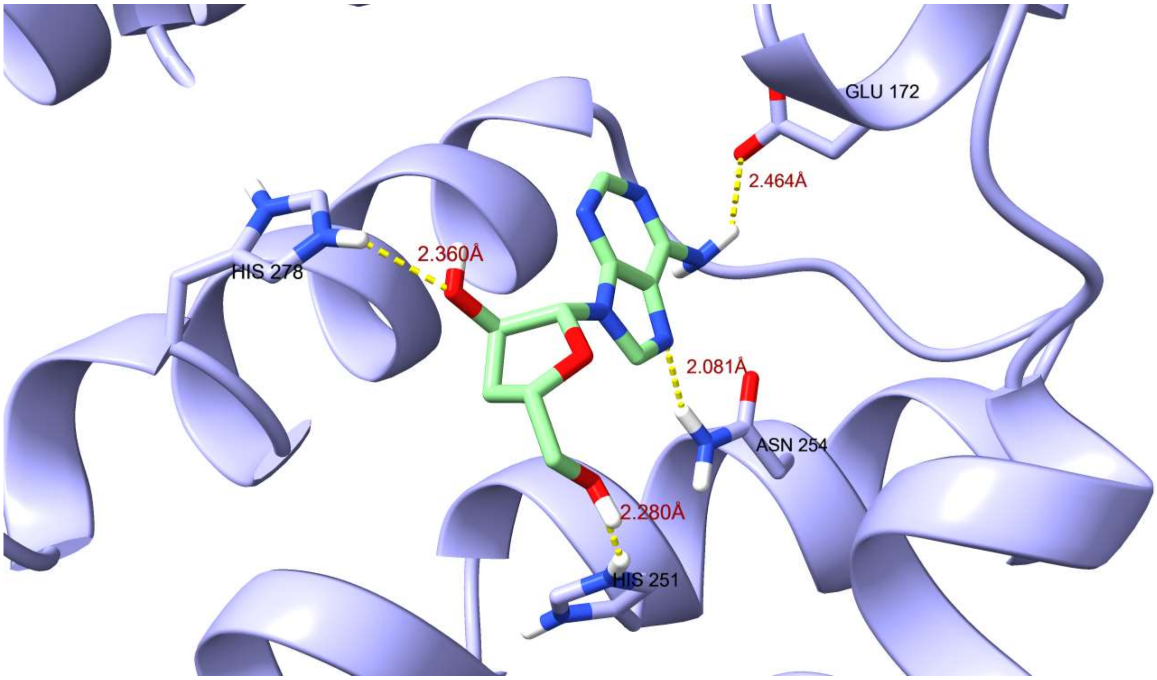

Figure S84. Predicted binding mode of COR with A_1R.

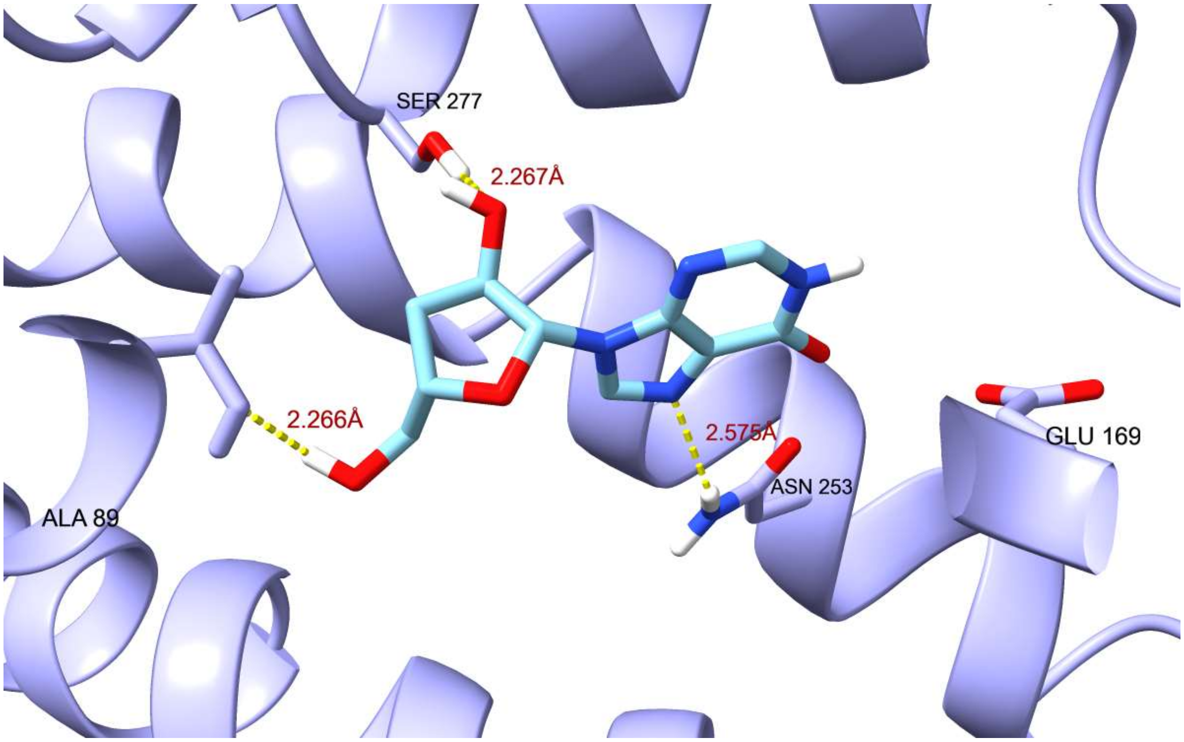

Figure S85. Predicted binding mode of 3'-dINO with $A_{2A}R$.

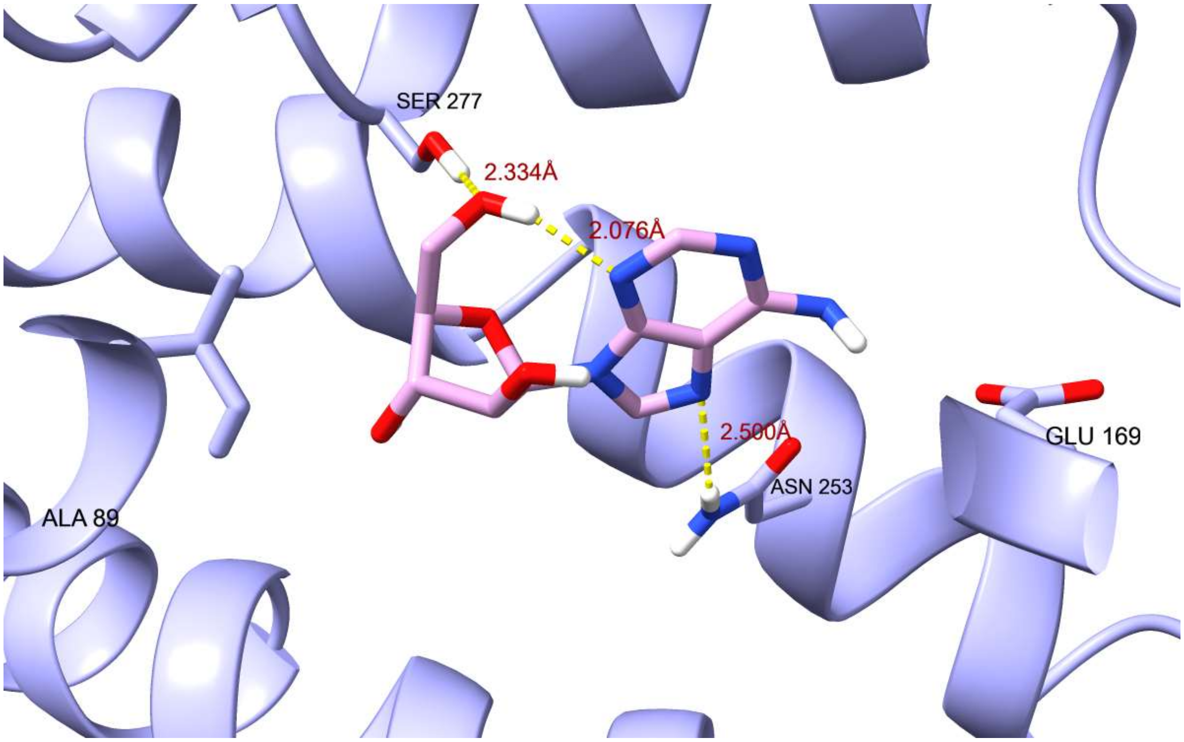

Figure S86. Predicted binding mode of ADO with $A_{2A}R$.

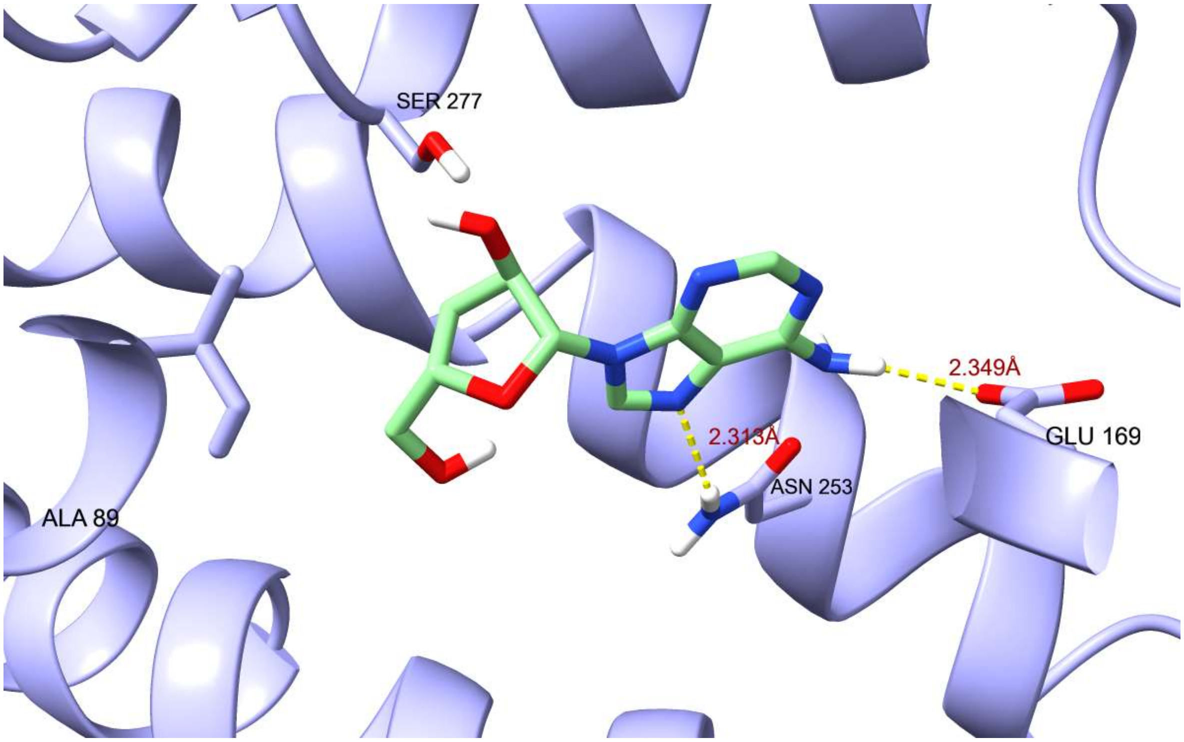

Figure S87. Predicted binding mode of COR with $A_{2A}R$.

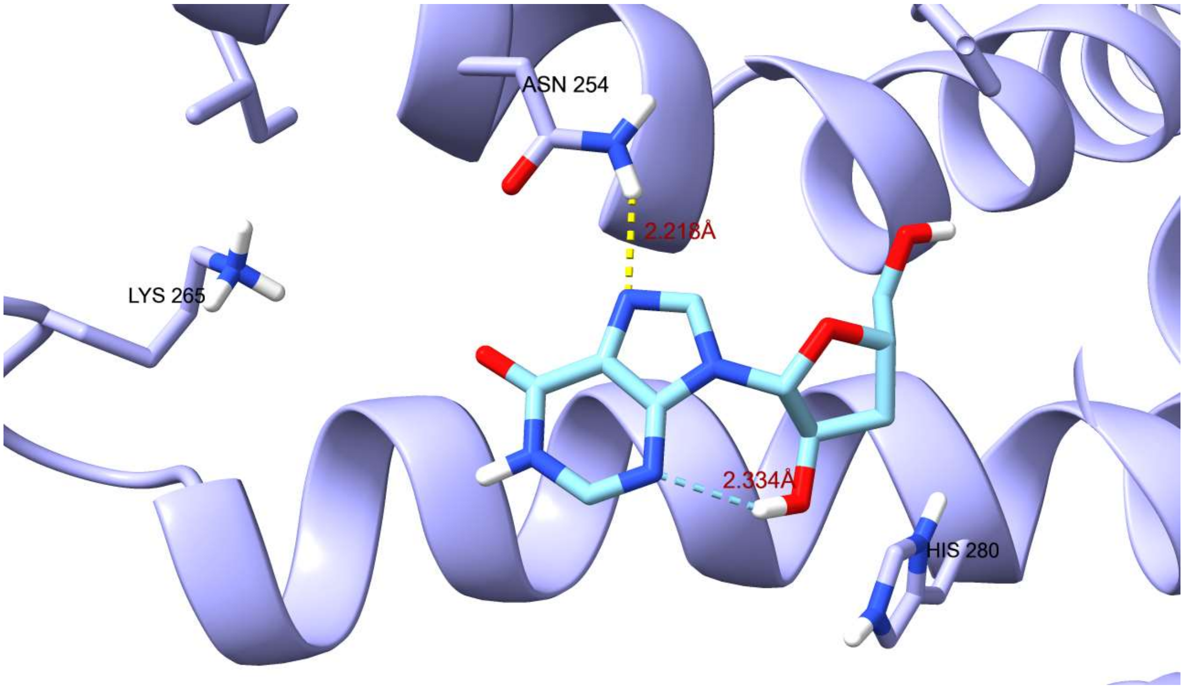

Figure S88. Predicted binding mode of 3’-dINO with $A_{2B}R$.

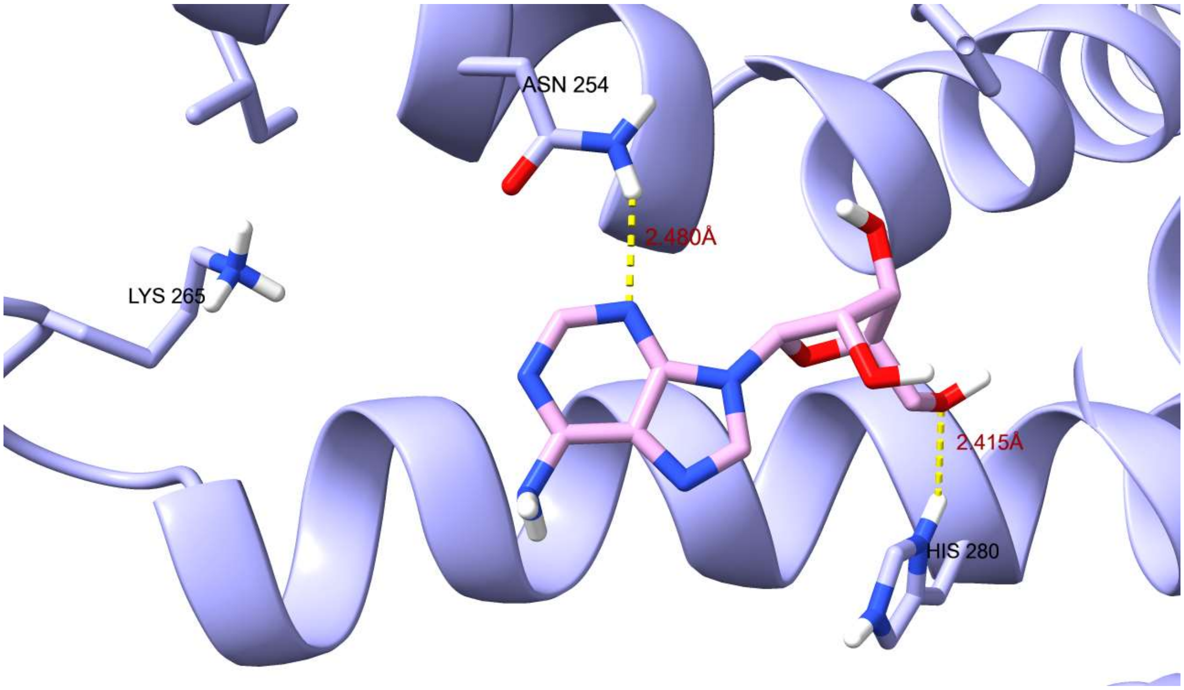

Figure S89. Predicted binding mode of ADO with $A_{2B}R$.

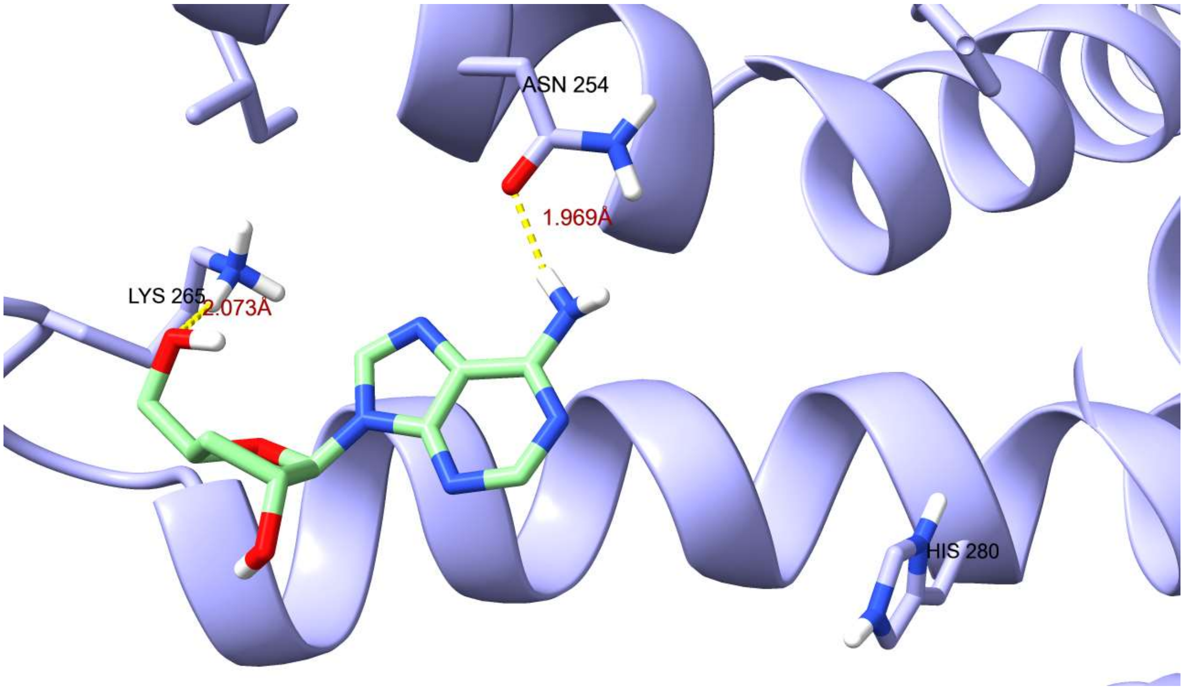

Figure S90. Predicted binding mode of COR with $A_{2B}R$.

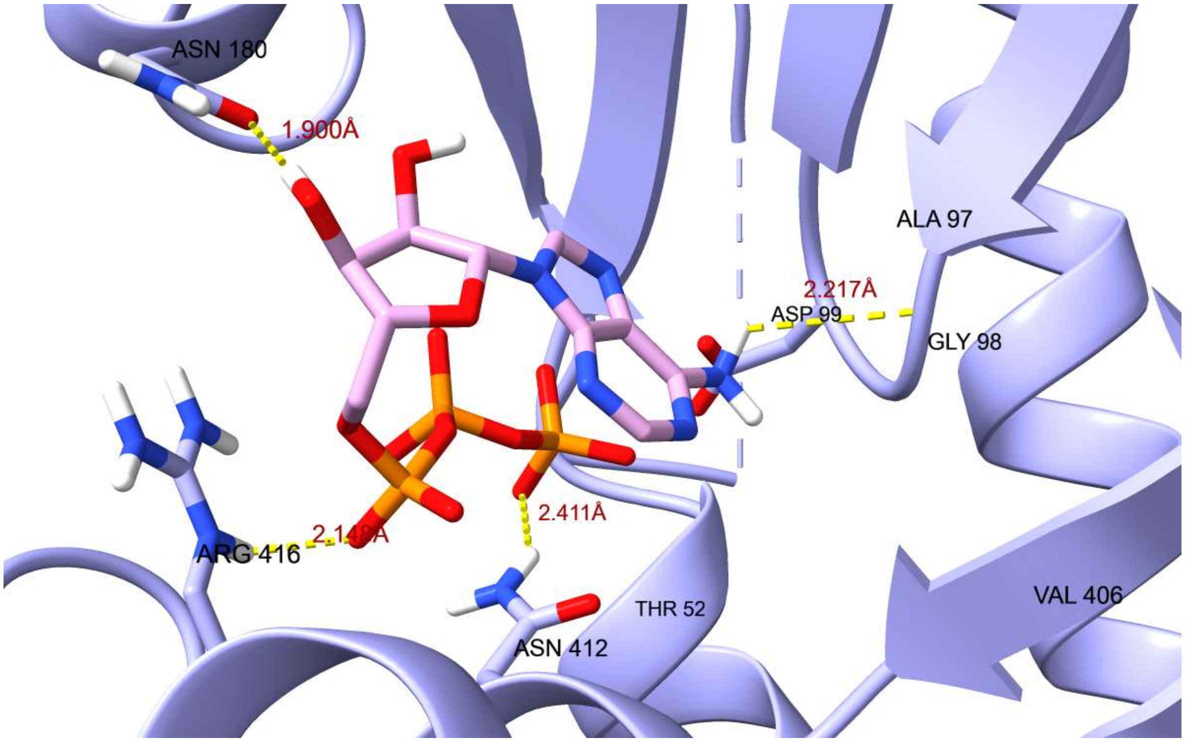

Figure S91. Predicted binding mode of ATP with sAC.

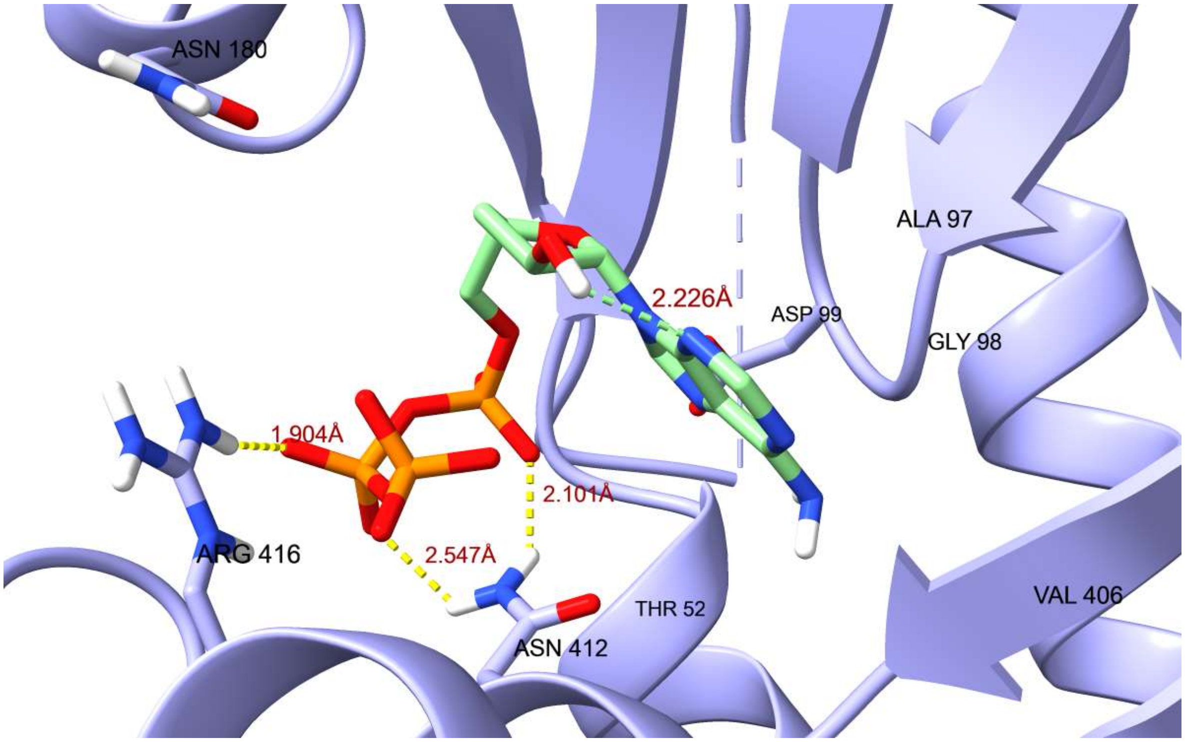

Figure S92. Predicted binding mode of COR-TP with sAC.

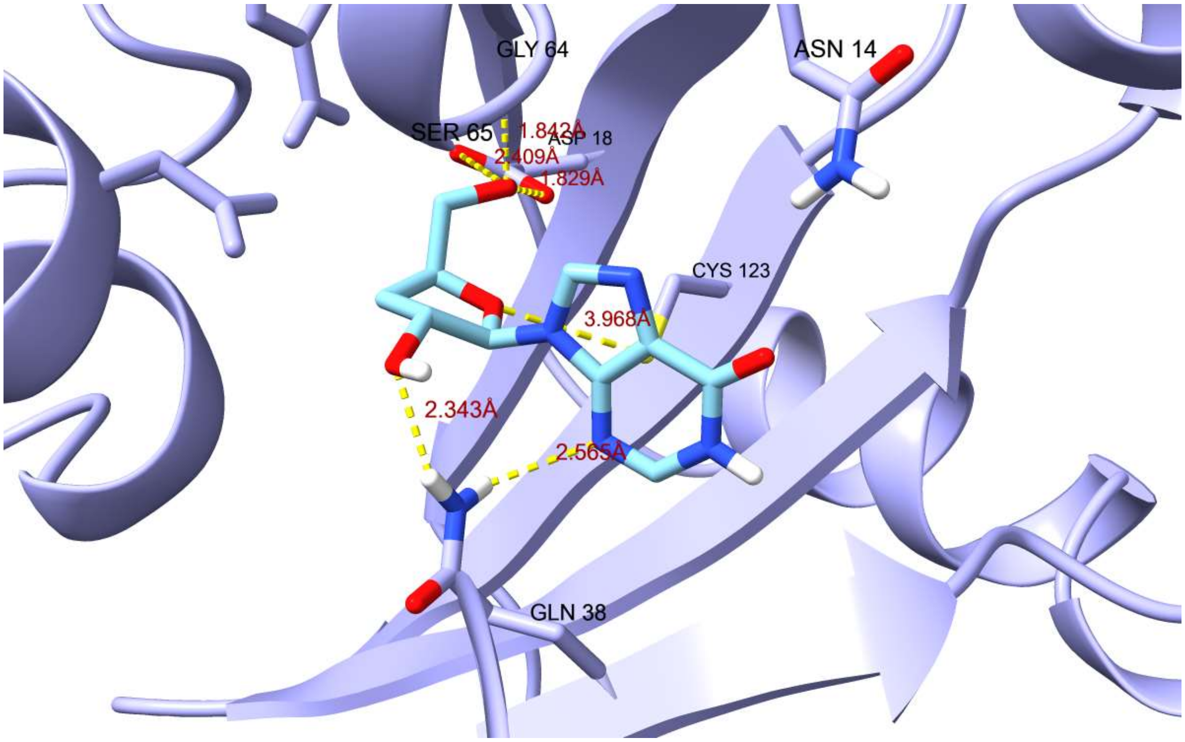

Figure S93. Predicted binding mode of 3’-dINO with ADK (site 1).

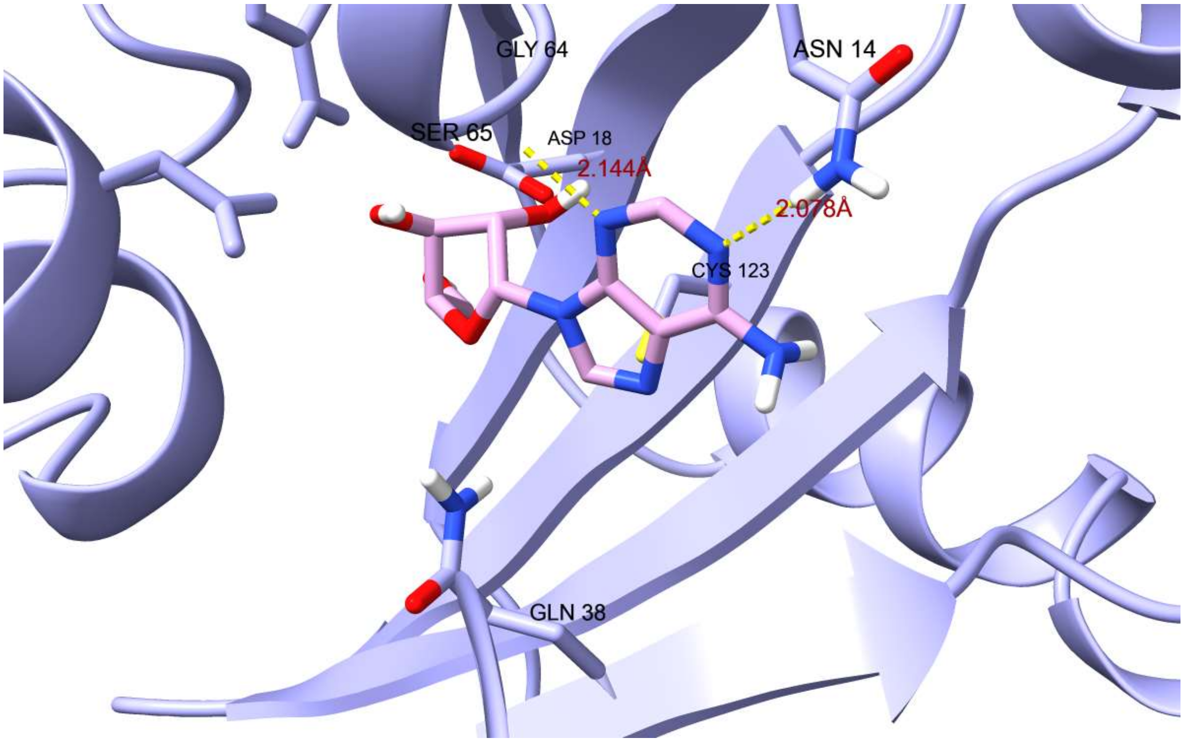

Figure S94. Predicted binding mode of ADO with ADK (site 1).

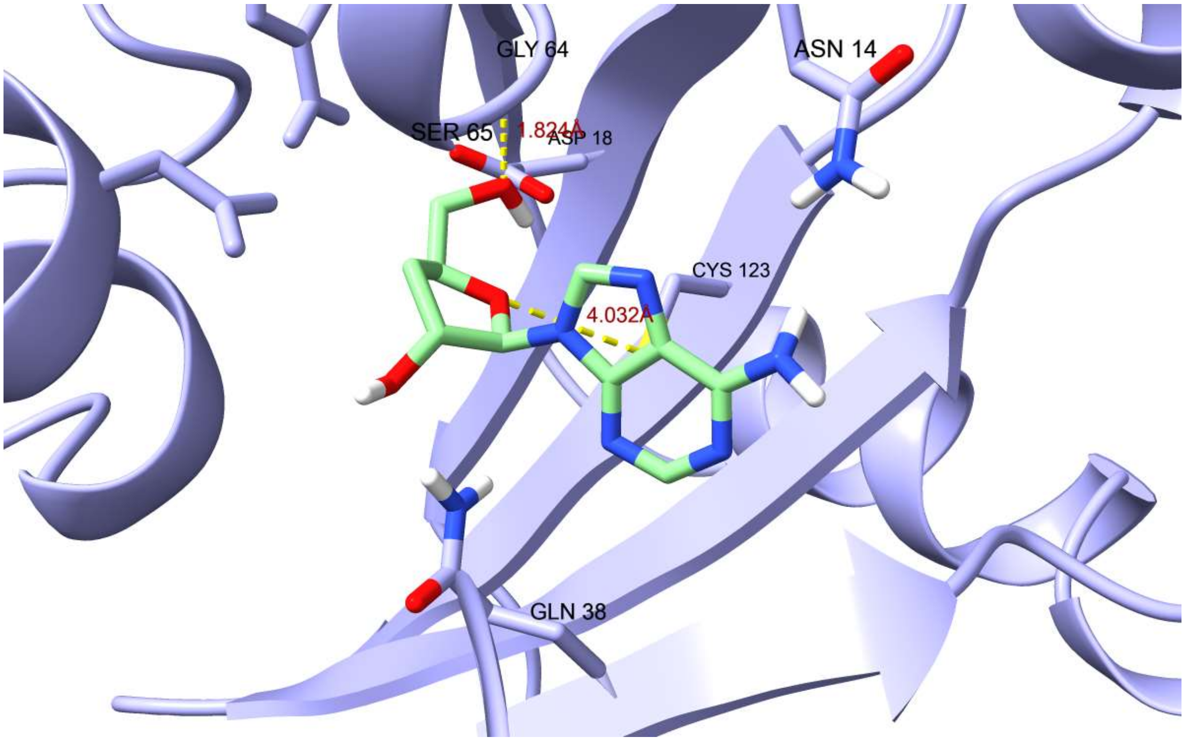

Figure S95. Predicted binding mode of COR with ADK (site 1).

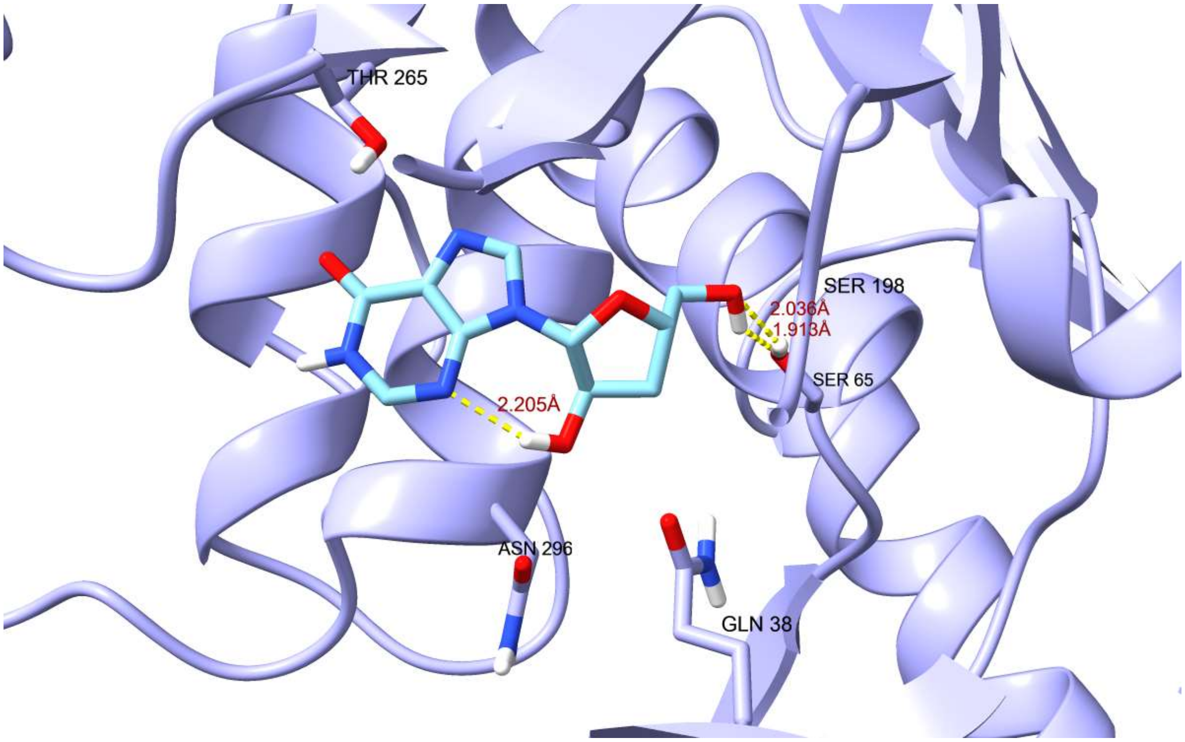

Figure S96. Predicted binding mode of 3’-dINO with ADK (site 2).

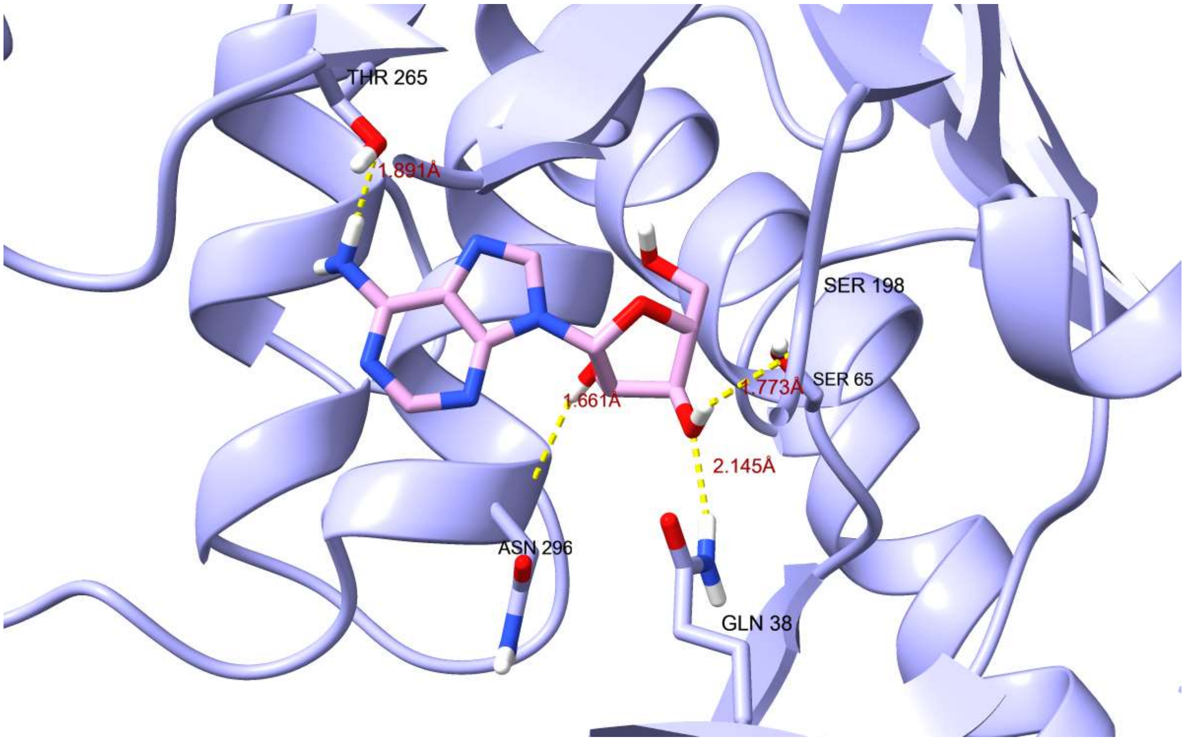

Figure S97. Predicted binding mode of ADO with ADK (site 2).

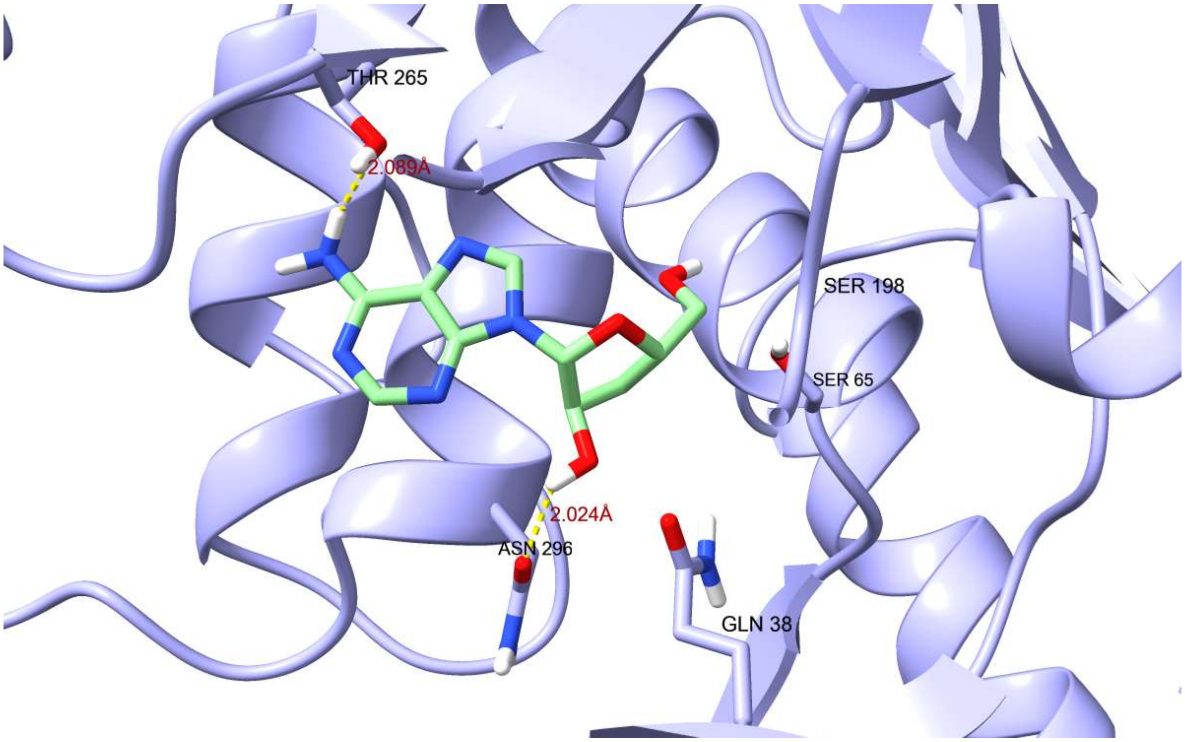

Figure S98. Predicted binding mode of COR with ADK (site 2).

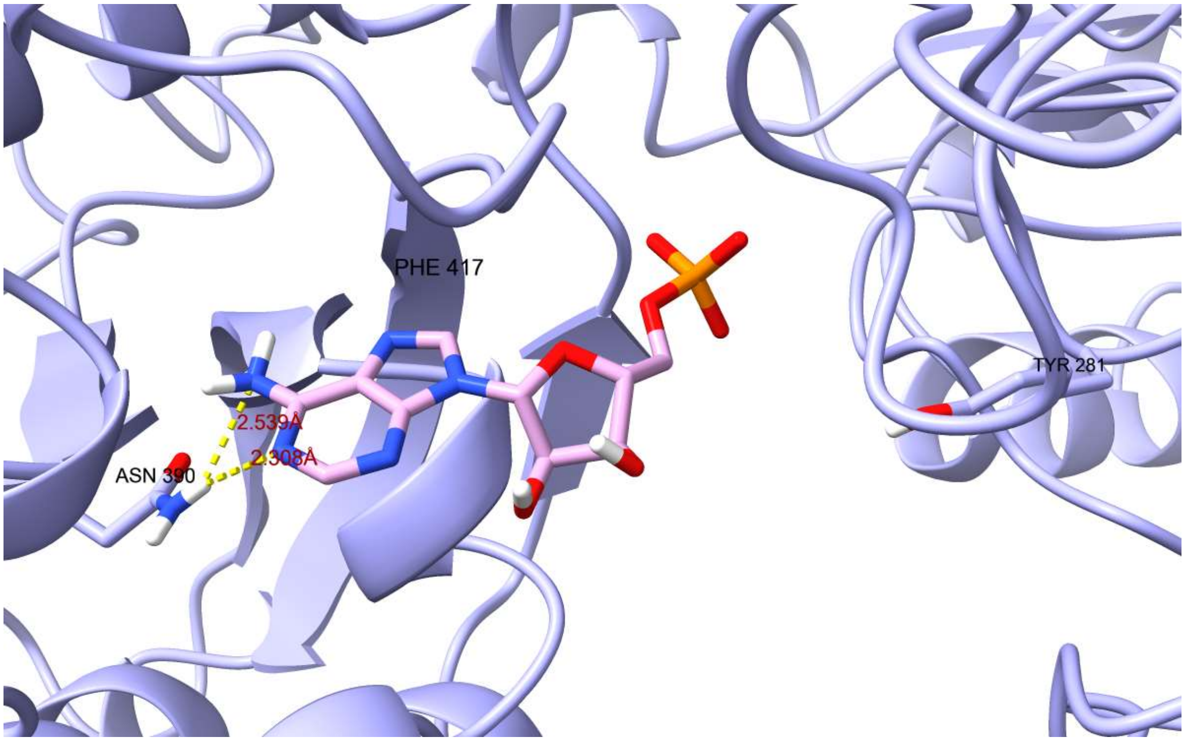

Figure S99. Predicted binding mode of AMP with CD73.

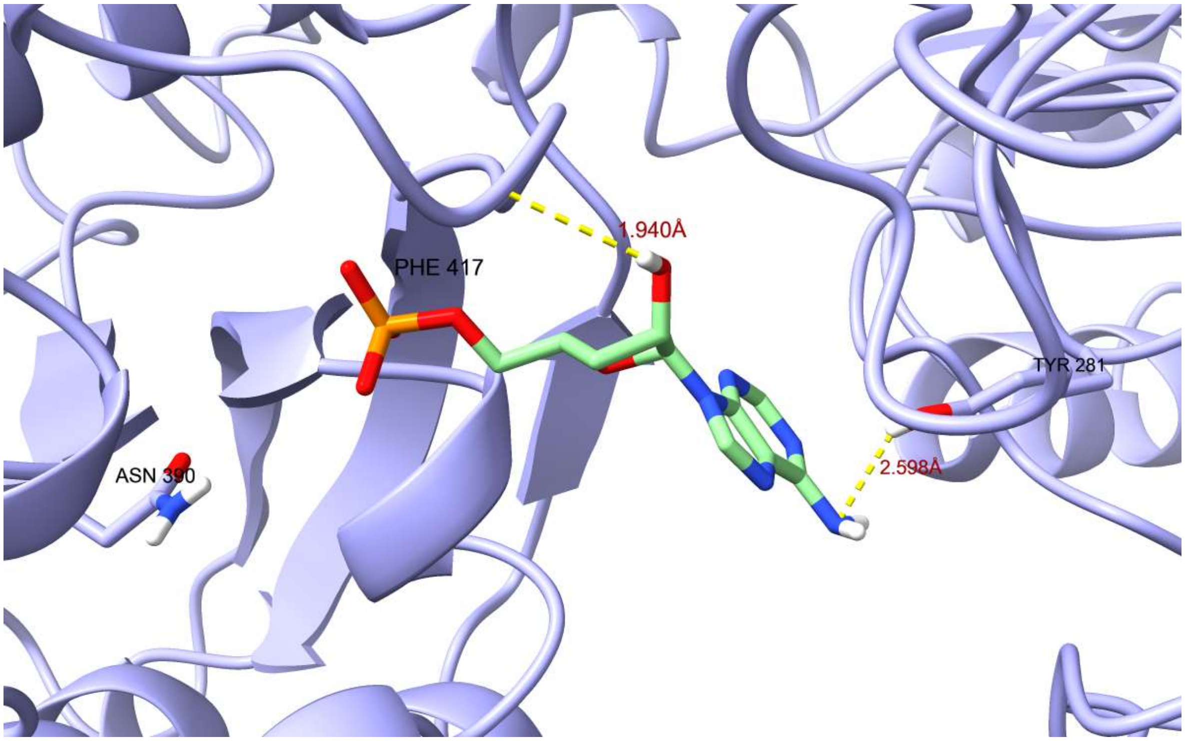

Figure S100. Predicted binding mode of COR-MP with CD73.

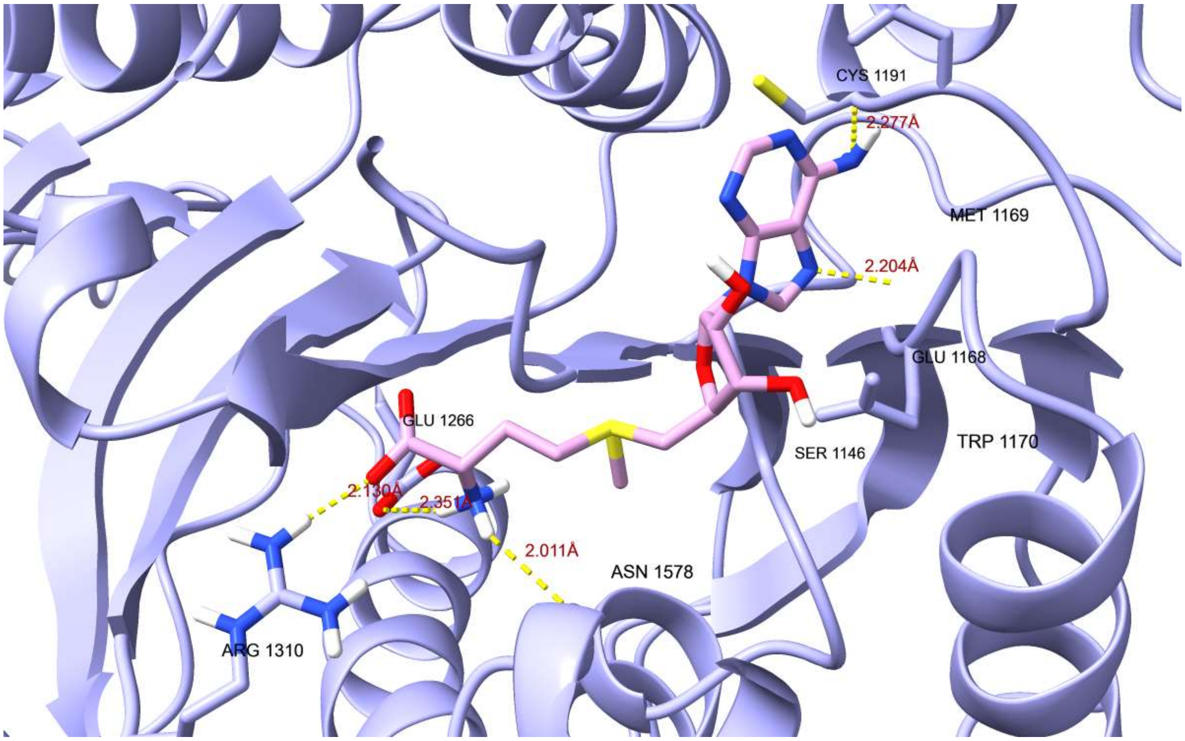

Figure S101. Predicted binding mode of SAM with DNMT1.

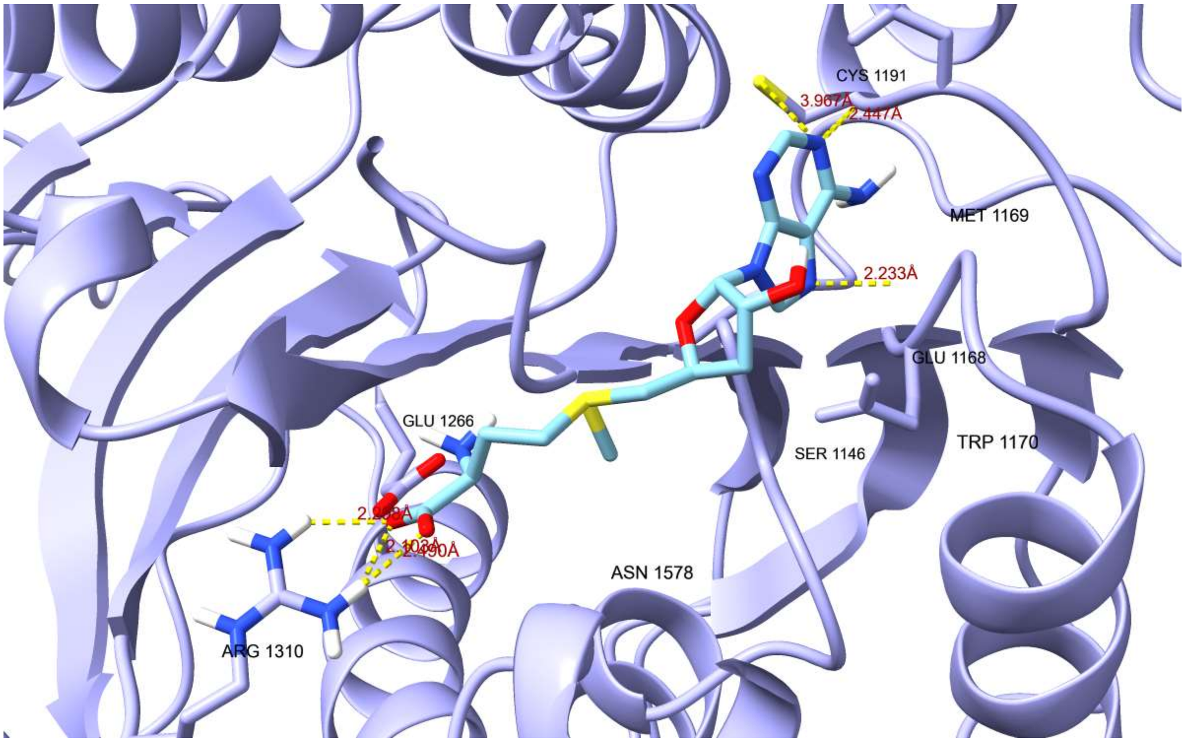

Figure S102. Predicted binding mode of 3’-dSAM with DNMT1.

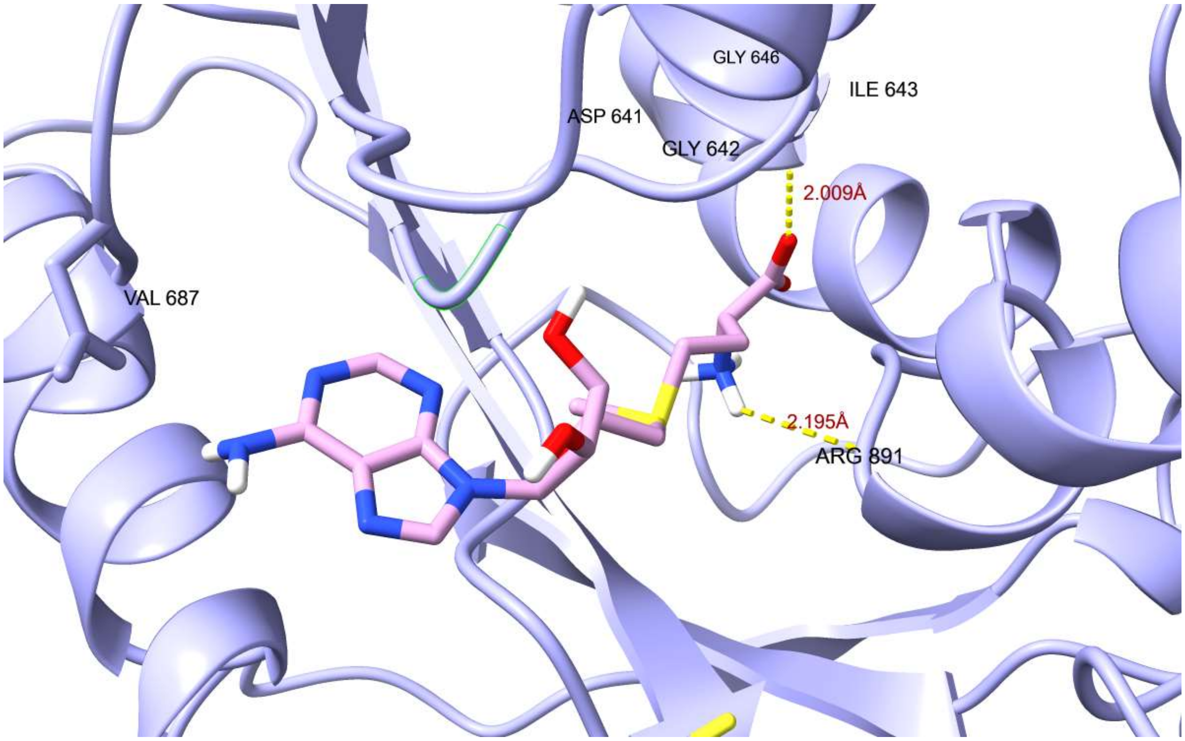

Figure S103. Predicted binding mode of SAM with DNMT3A.

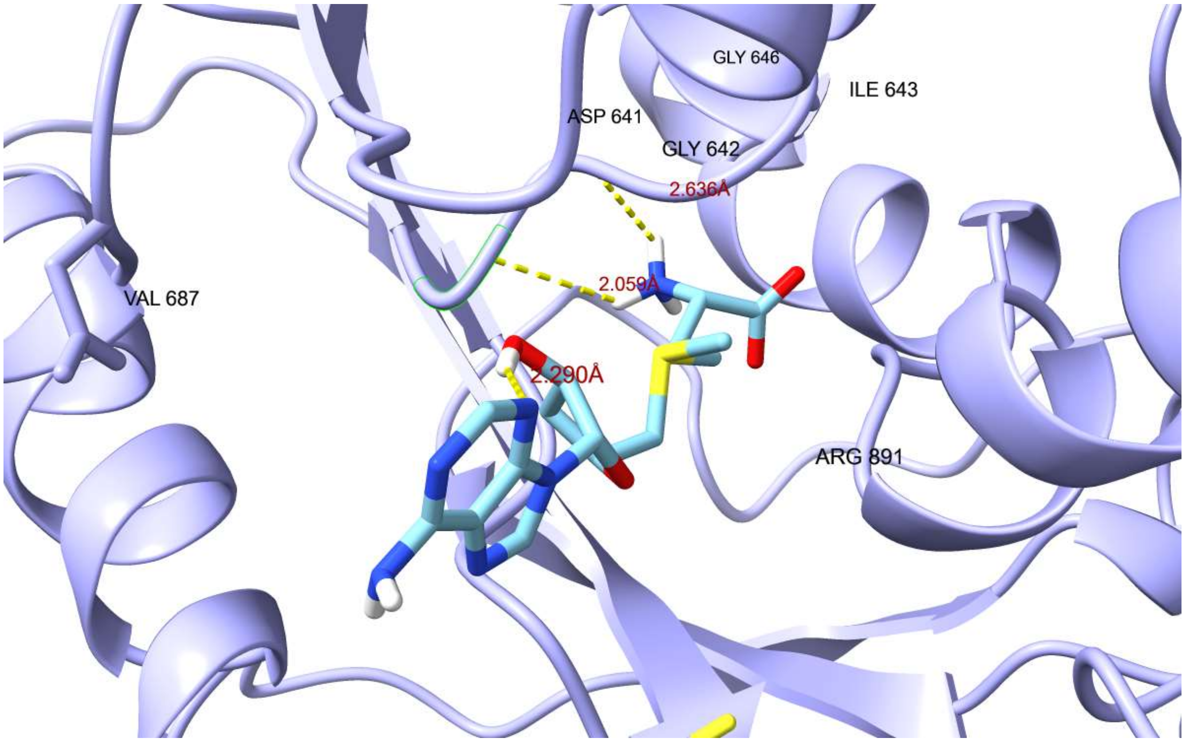

Figure S104. Predicted binding mode of 3’-dSAM with DNMT3A.

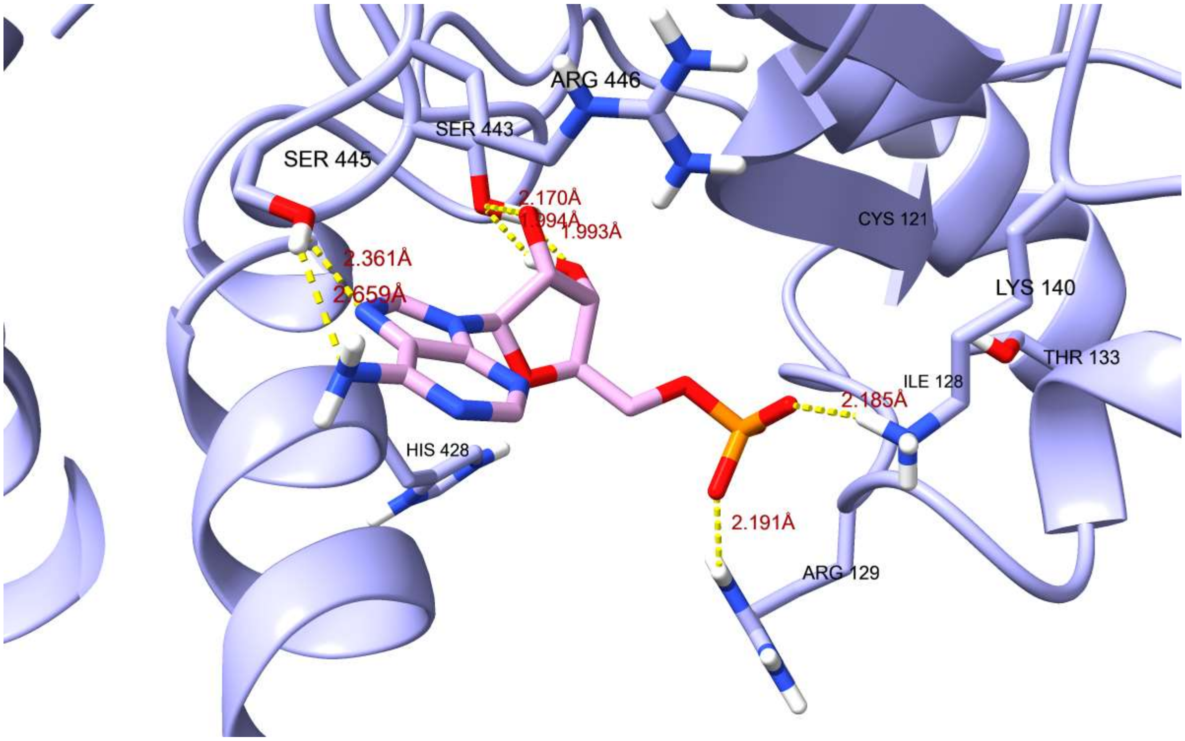

Figure S105. Predicted binding mode of AMP with NT5C2 (site 1).

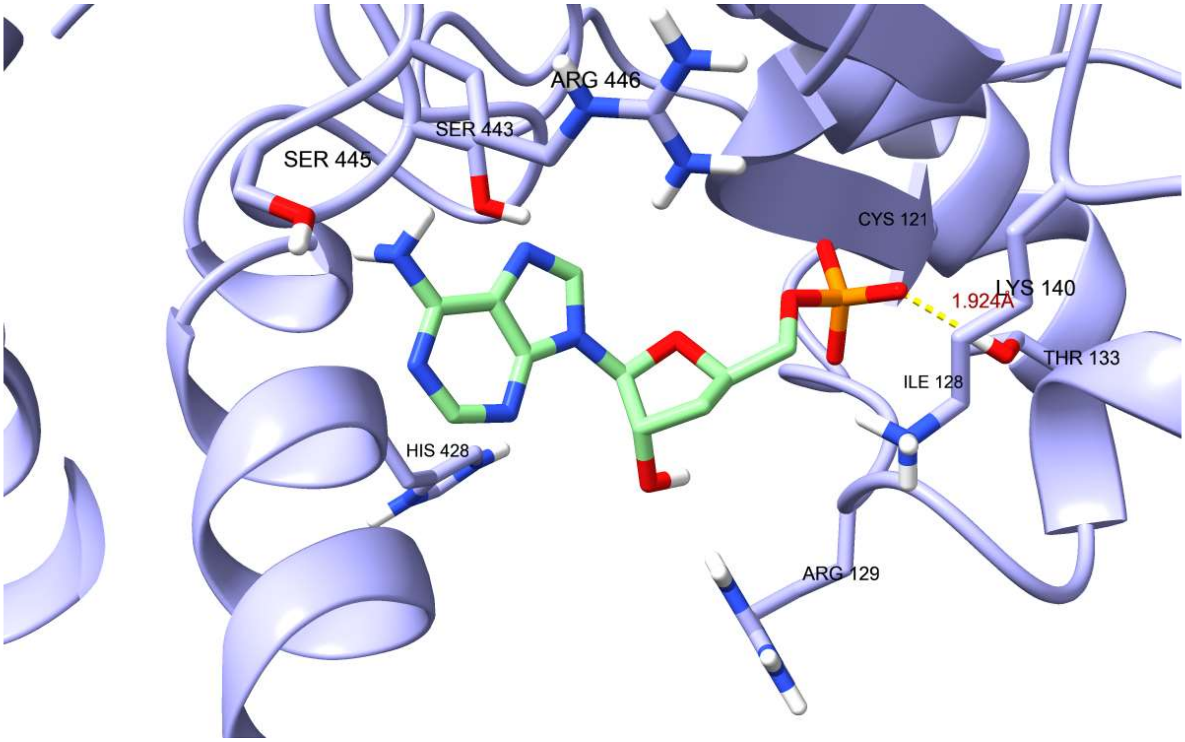

Figure S106. Predicted binding mode of COR-MP with NT5C2 (site 1).

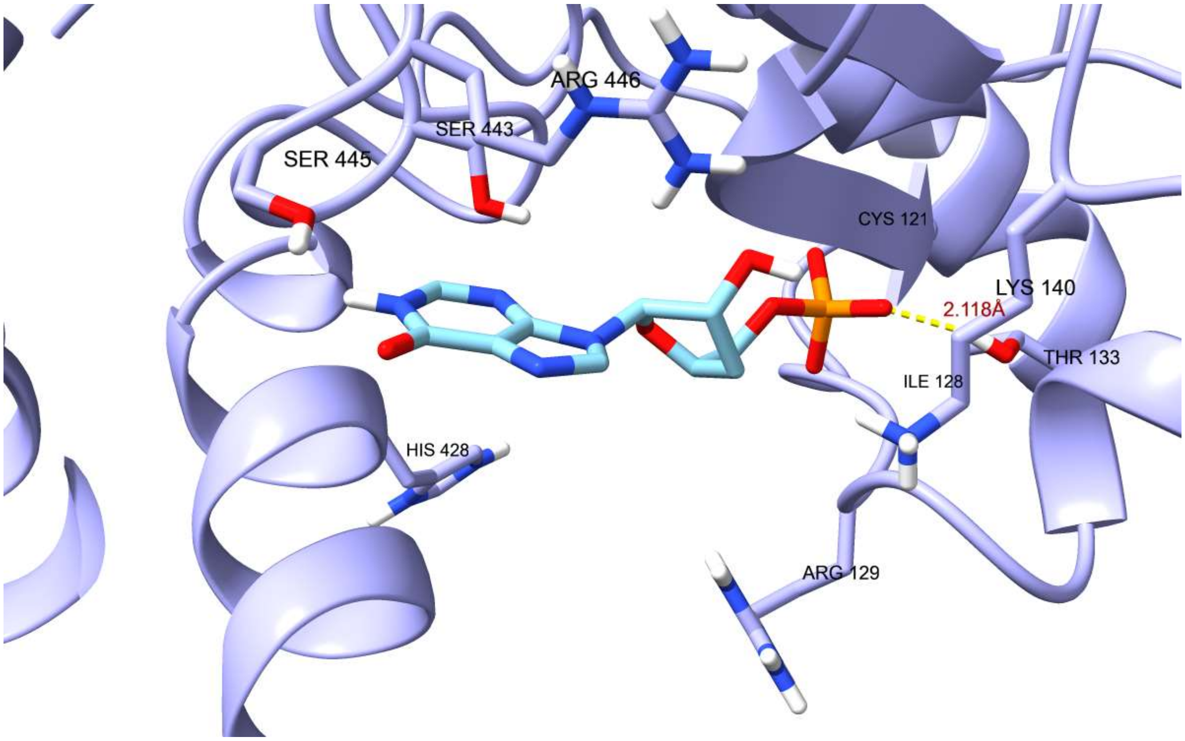

Figure S107. Predicted binding mode of 3'-dIMP with NT5C2 (site 1).

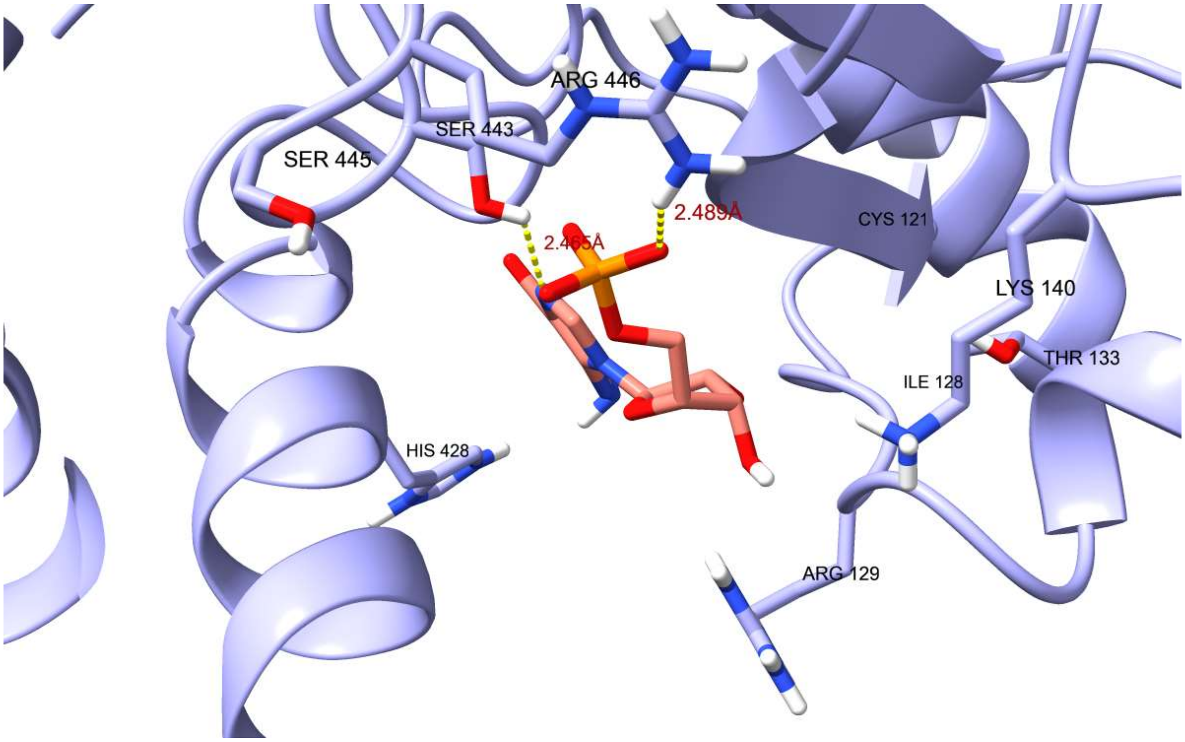

Figure S108. Predicted binding mode of GMP with NT5C2 (site 1).

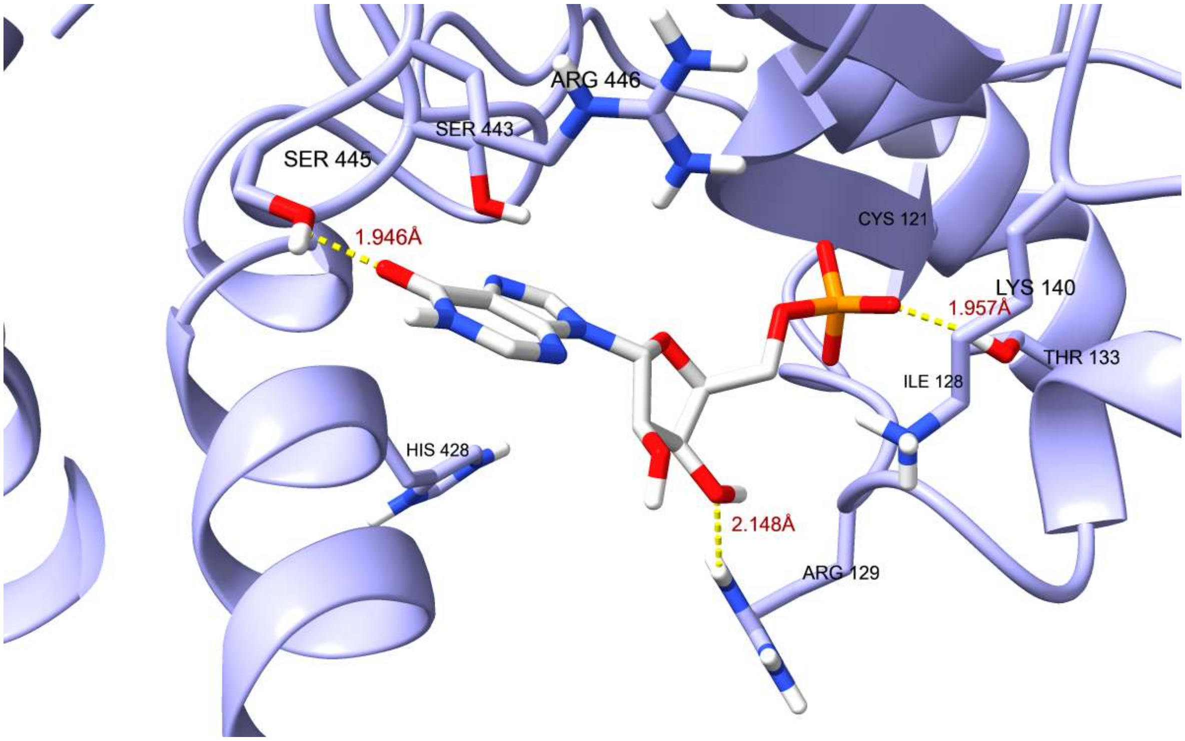

Figure S109. Predicted binding mode of IMP with NT5C2 (site 1).

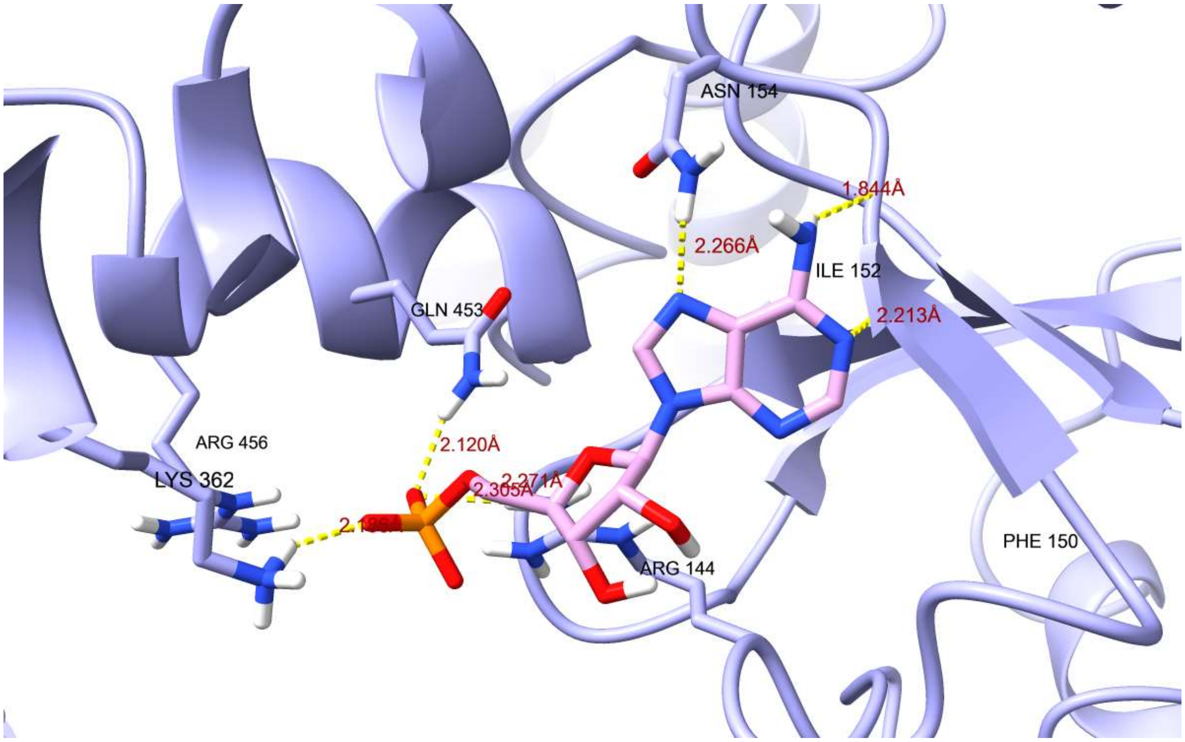

Figure S110. Predicted binding mode of AMP with NT5C2 (site 2).

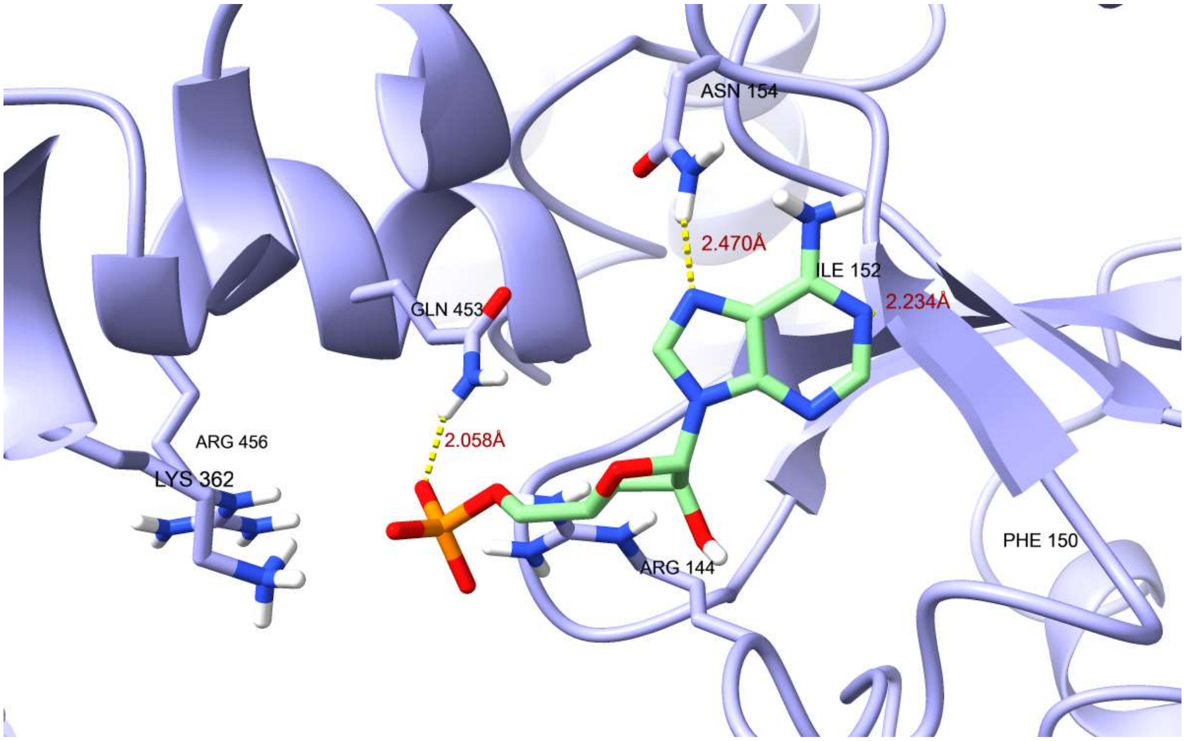

Figure S111. Predicted binding mode of COR-MP with NT5C2 (site 2).

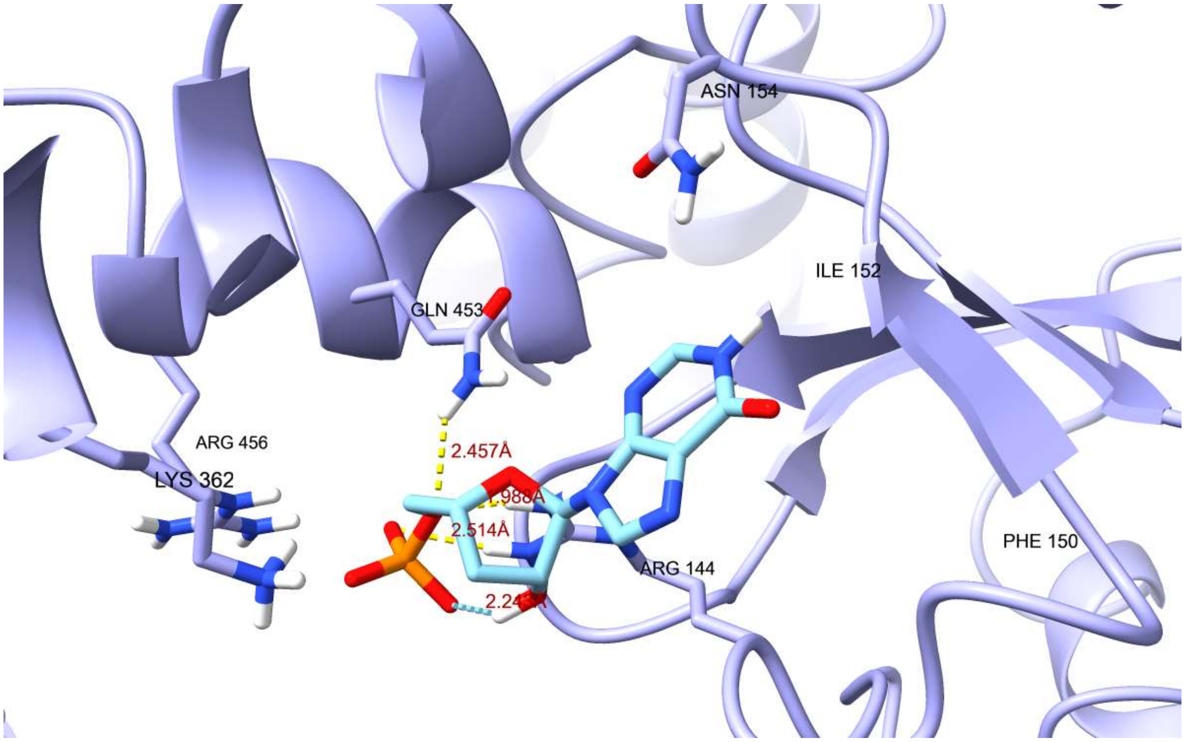

Figure S112. Predicted binding mode of 3’-dIMP with NT5C2 (site 2).

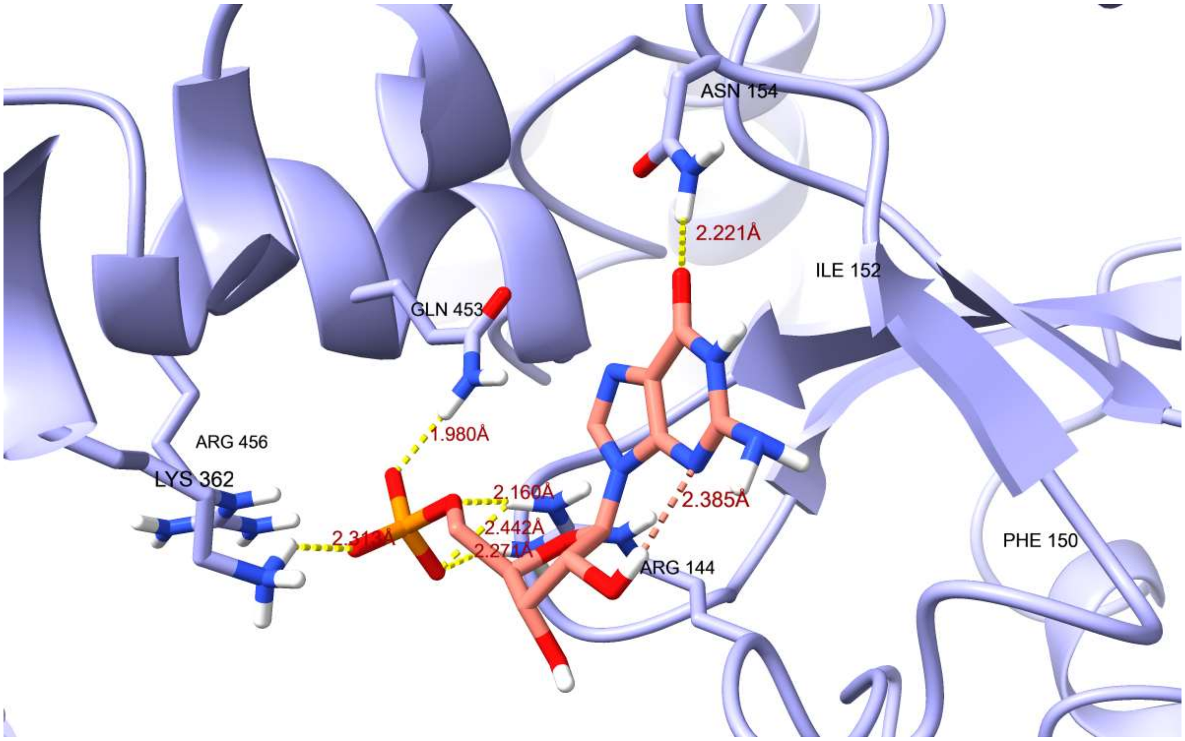

Figure S113. Predicted binding mode of GMP with NT5C2 (site 2).

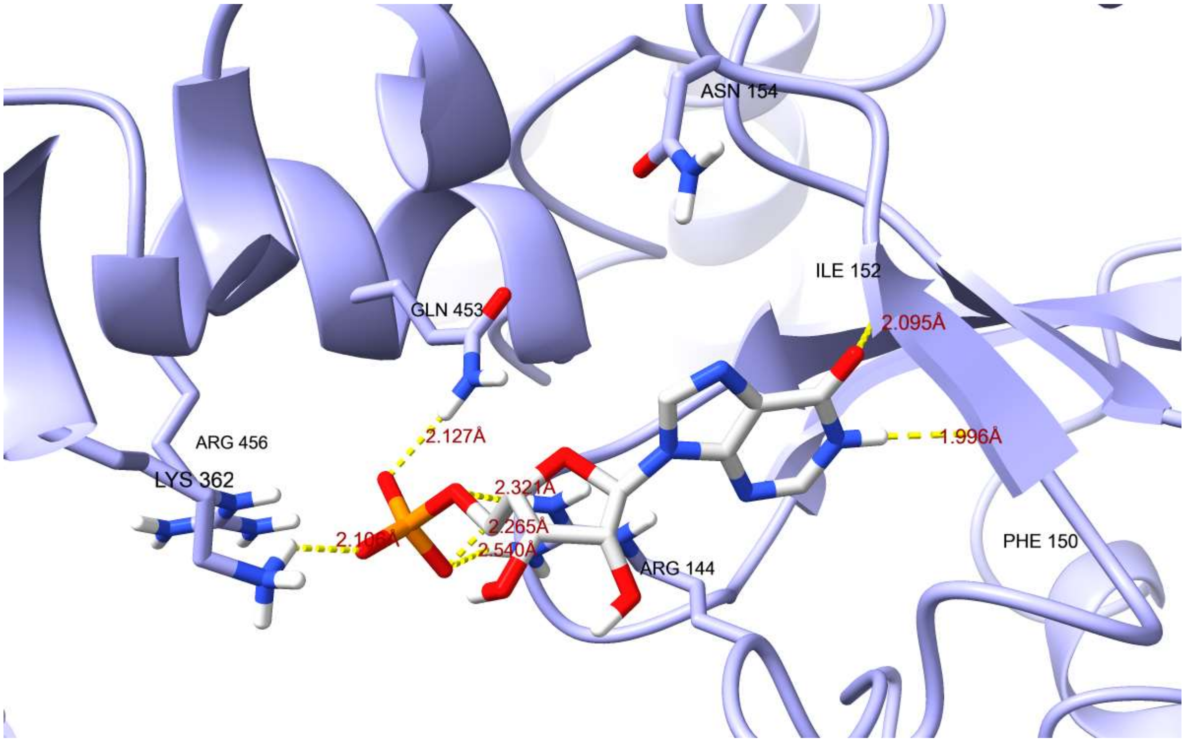

Figure S114. Predicted binding mode of IMP with NT5C2 (site 2).

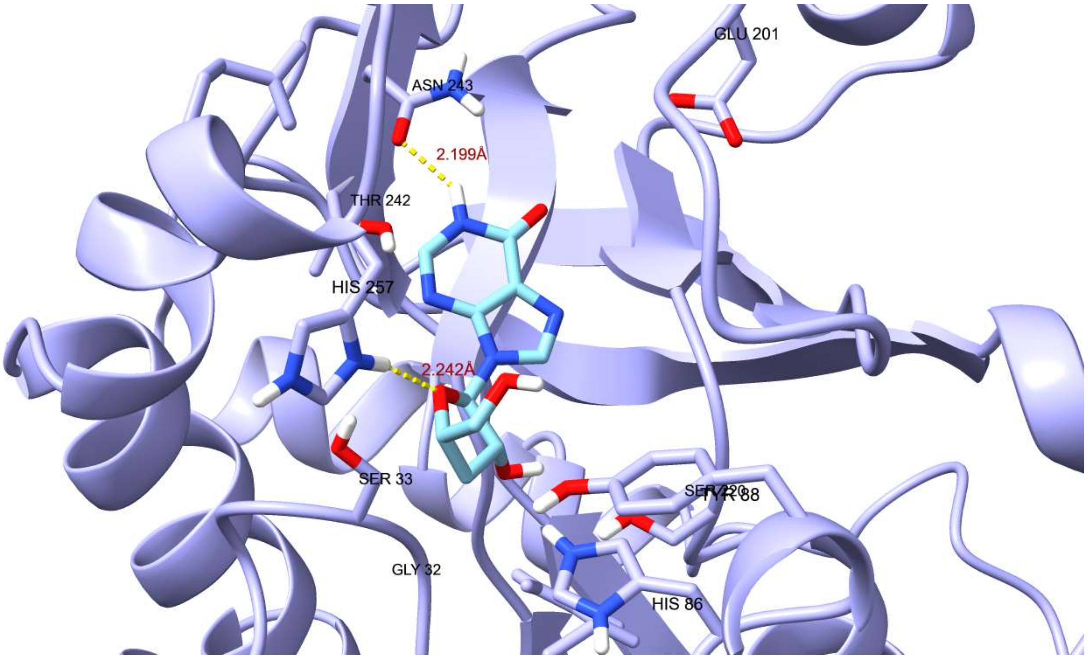

Figure S115. Predicted binding mode of 3’-dINO with PNP.

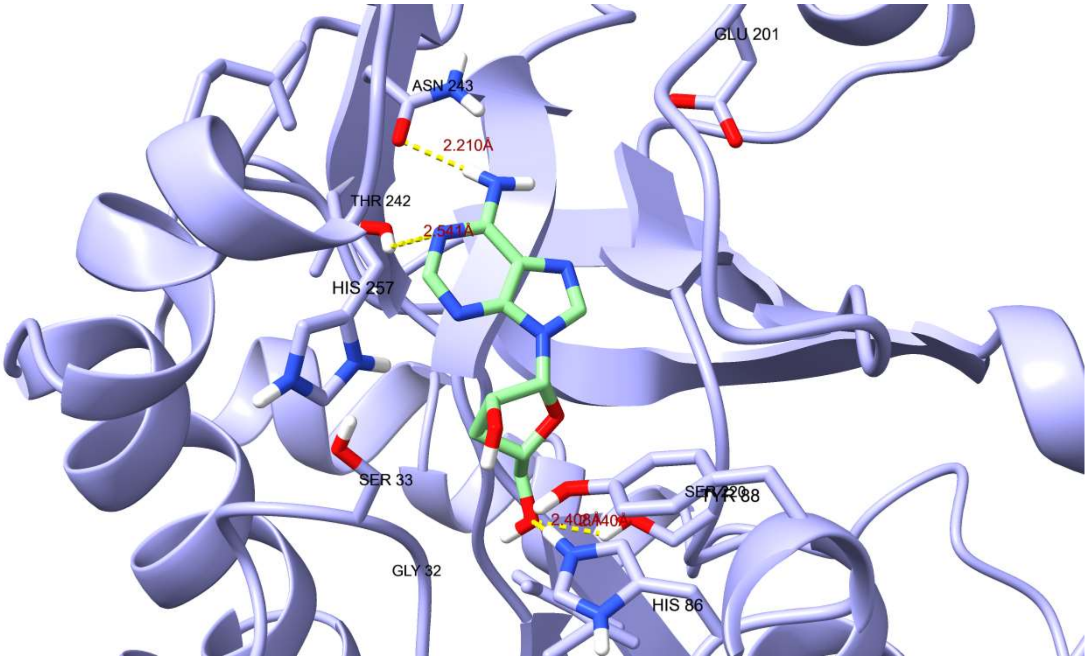

Figure S116. Predicted binding mode of COR with PNP.

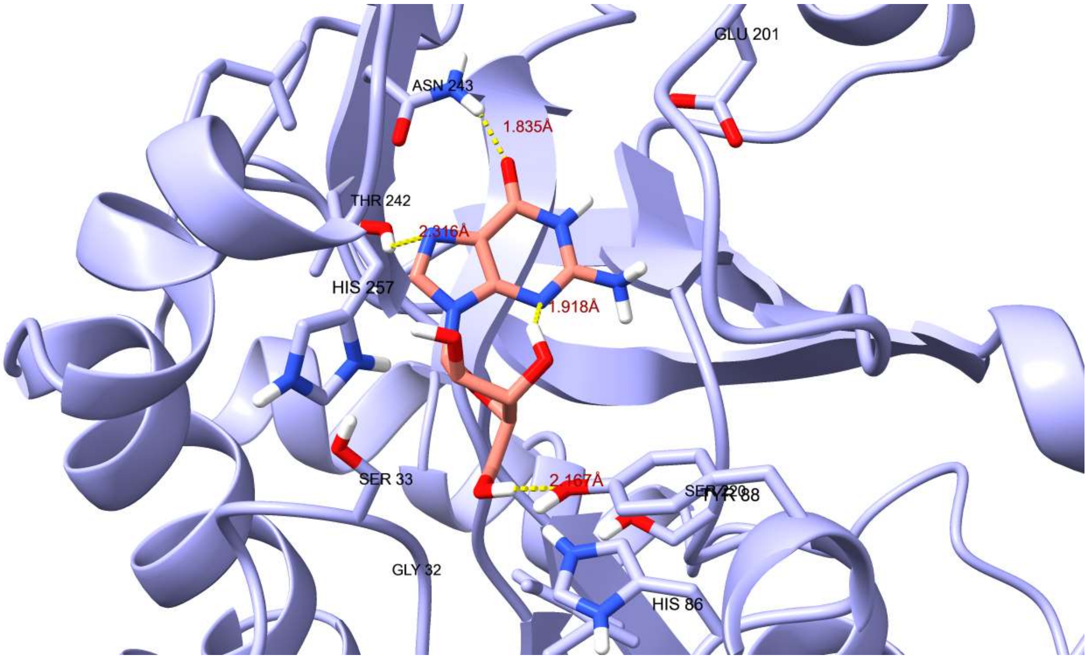

Figure S117. Predicted binding mode of GUA with PNP.

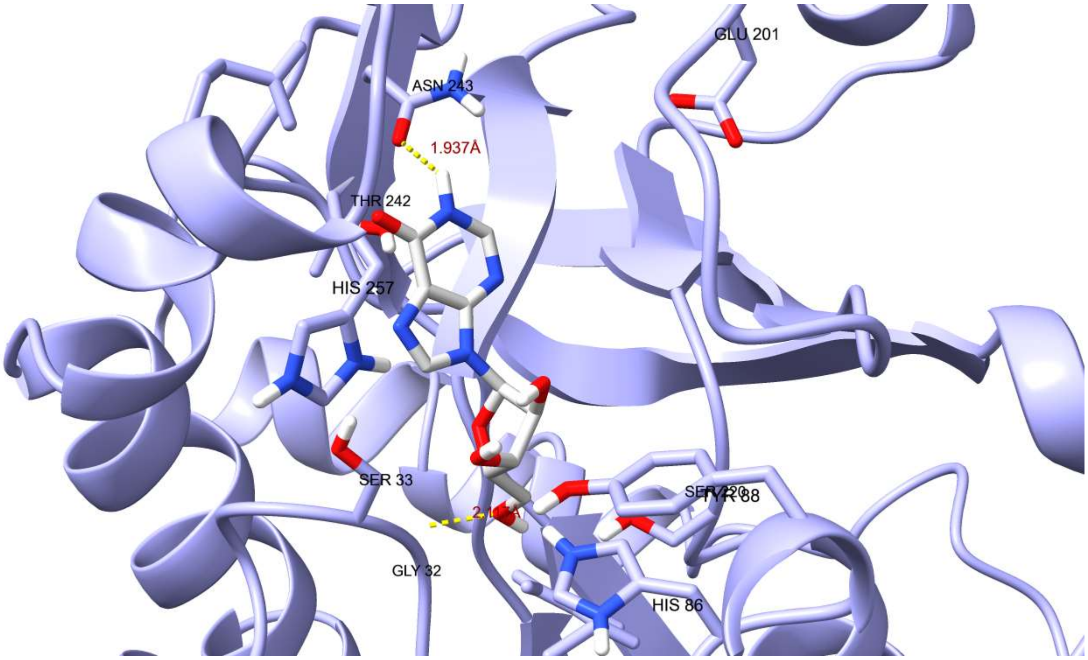

Figure 118. Predicted binding mode of INO with PNP.

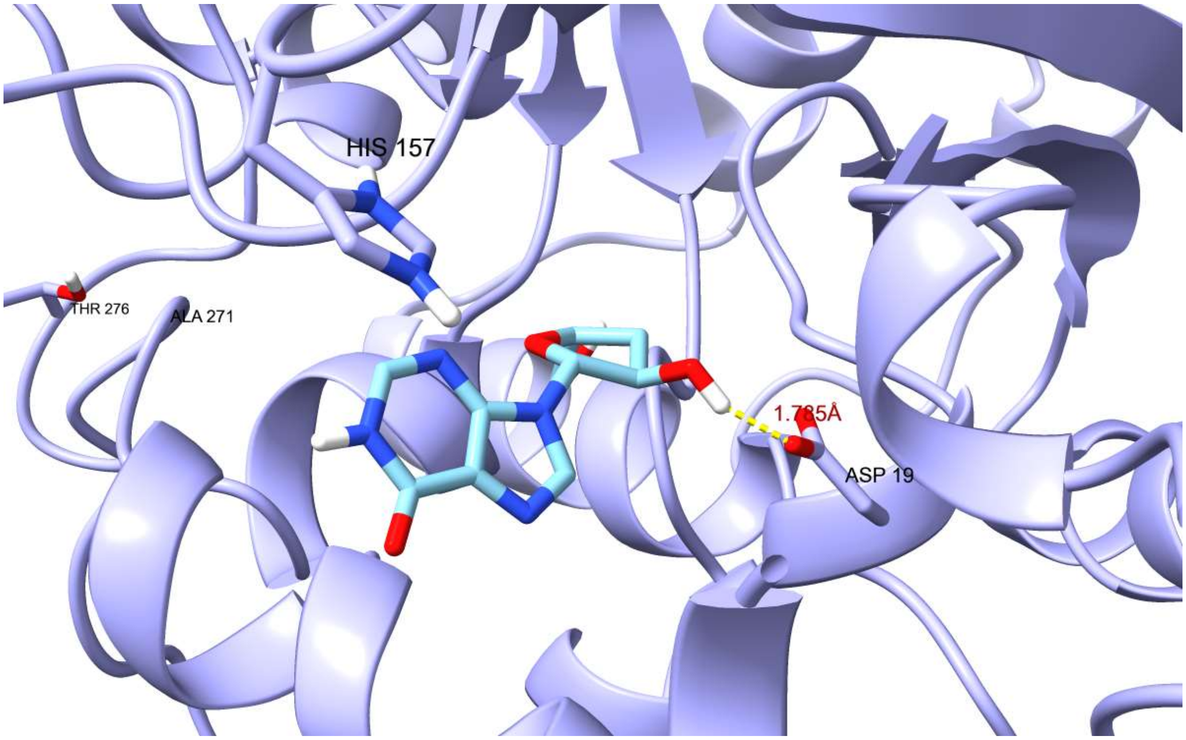

Figure S119. Predicted binding mode of 3'-dINO with ADA.

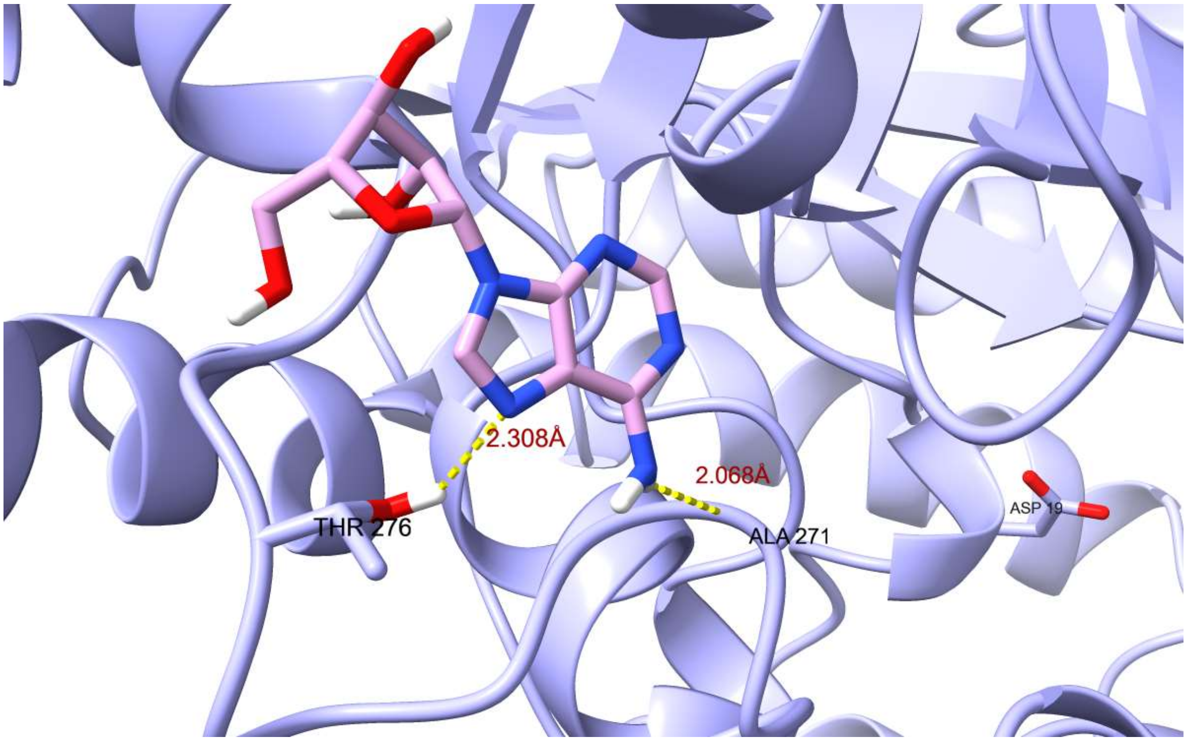

Figure S120. Predicted binding mode of ADO with ADA.

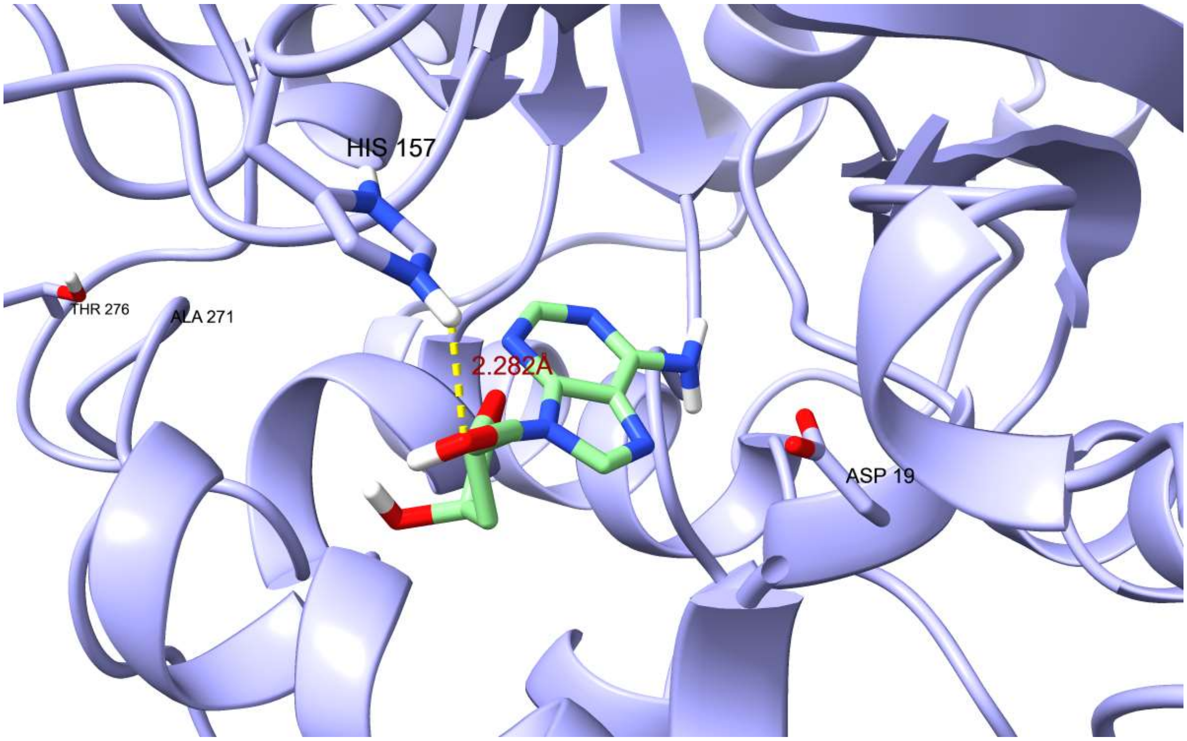

Figure S121. Predicted binding mode of COR with ADA.

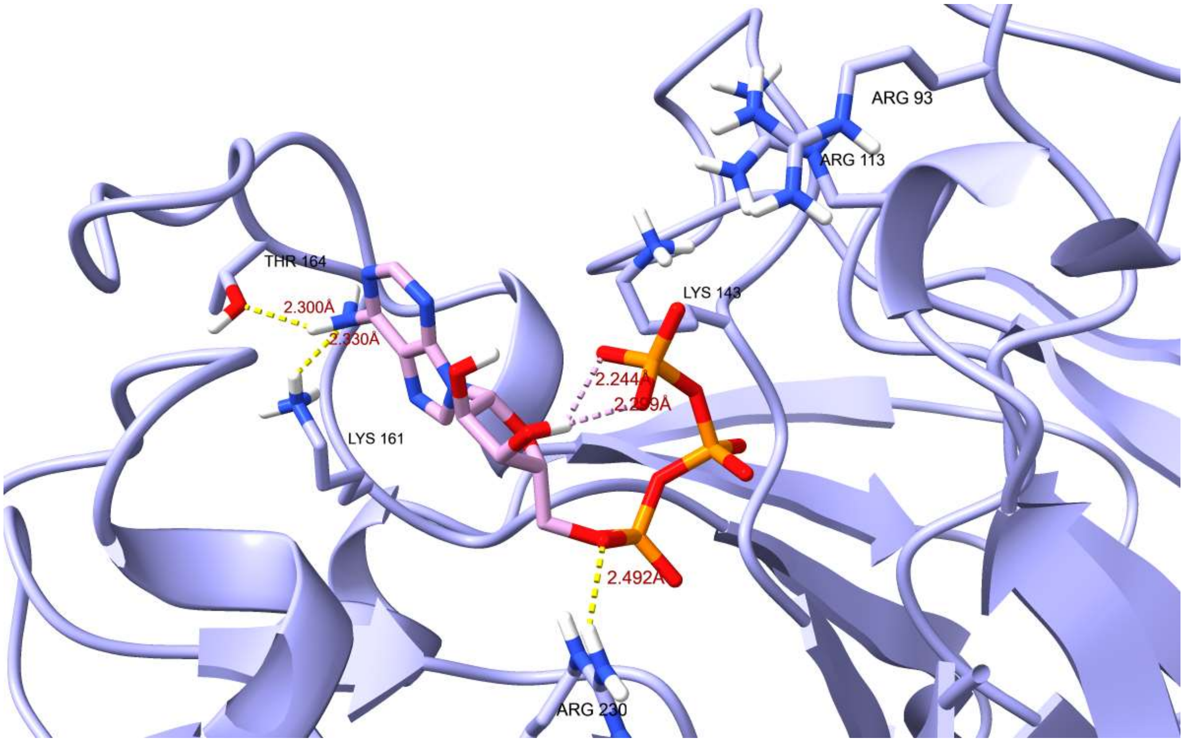

Figure S122. Predicted binding mode of ATP with CD39.

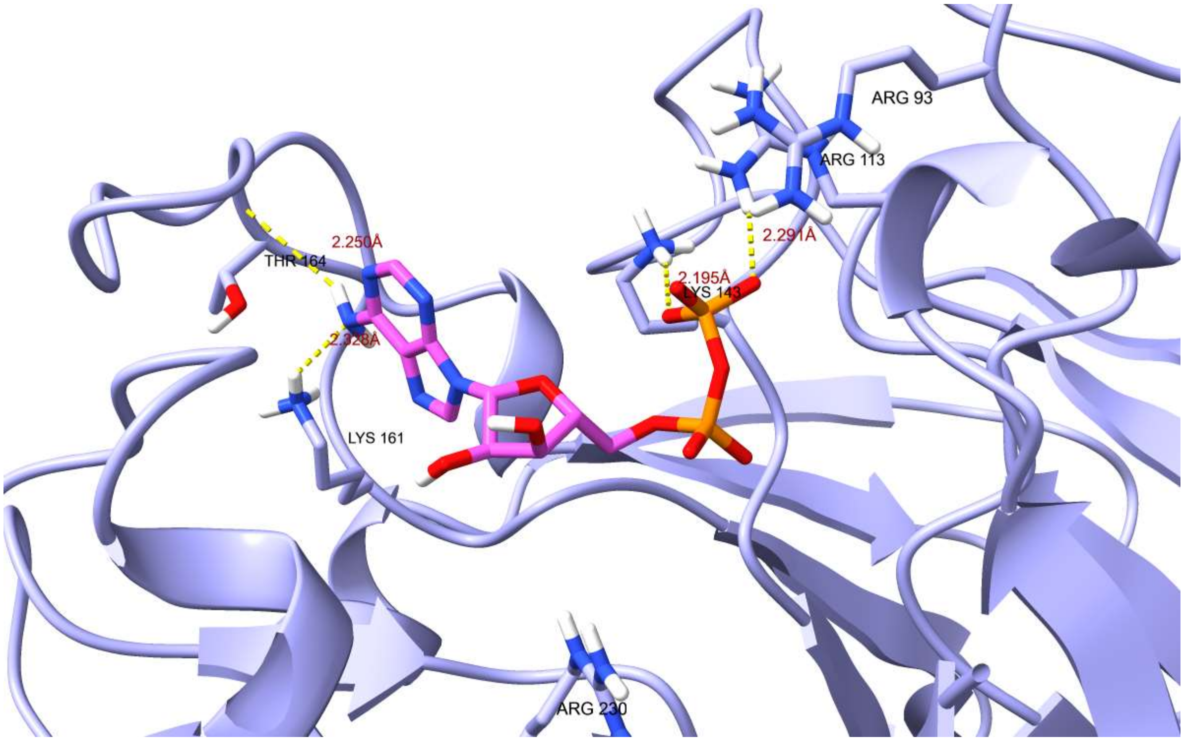

Figure S123. Predicted binding mode of ADP with CD39.

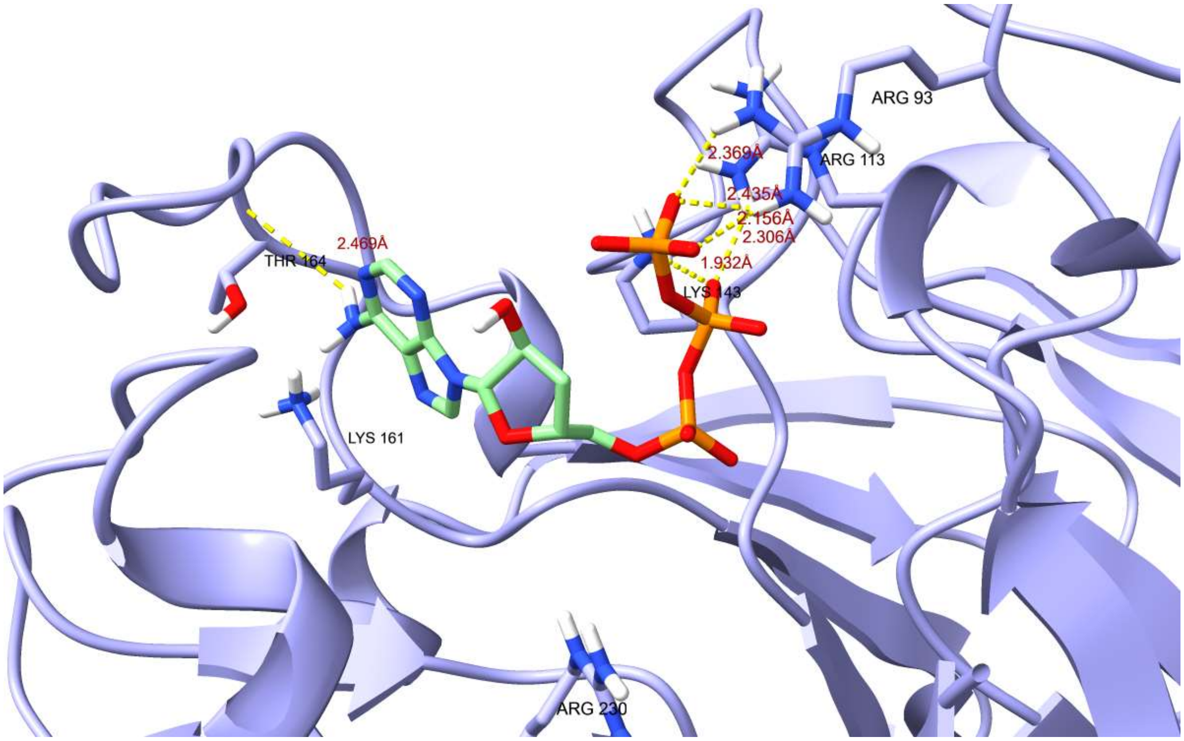

Figure S124. Predicted binding mode of COR-TP with CD39.

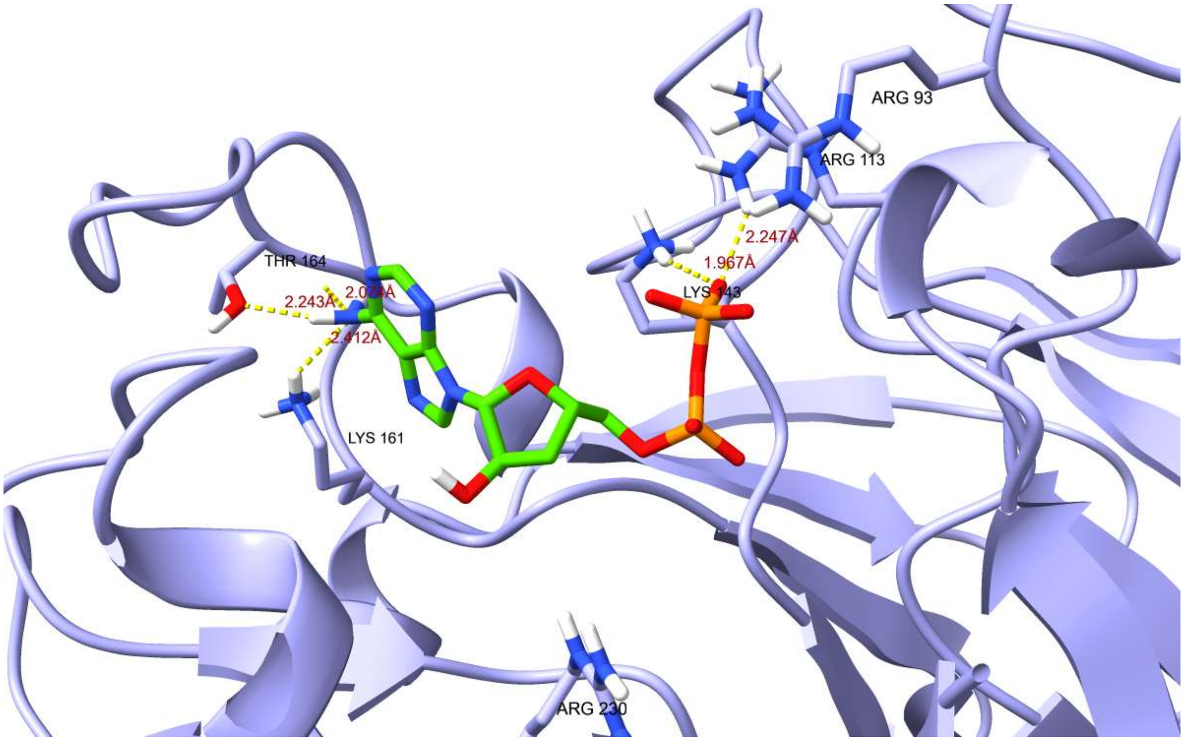

Figure S125. Predicted binding mode of COR-DP with CD39.

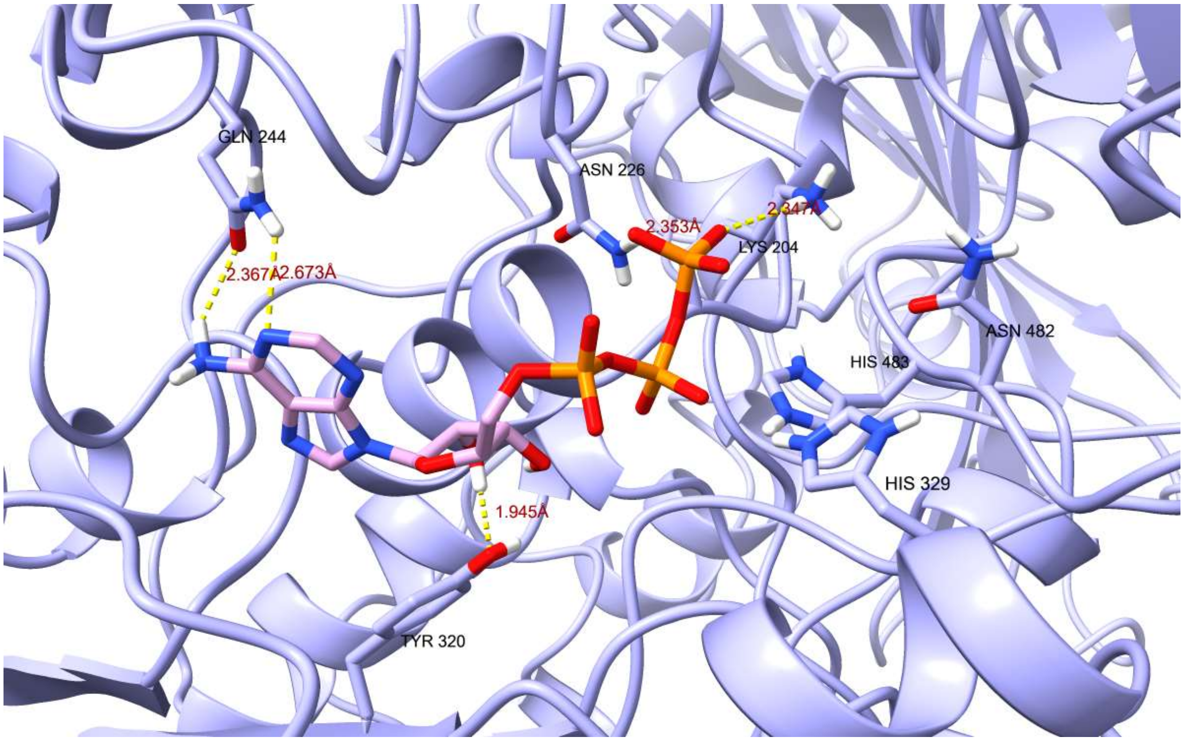

Figure S126. Predicted binding mode of ATP with ENPP3.

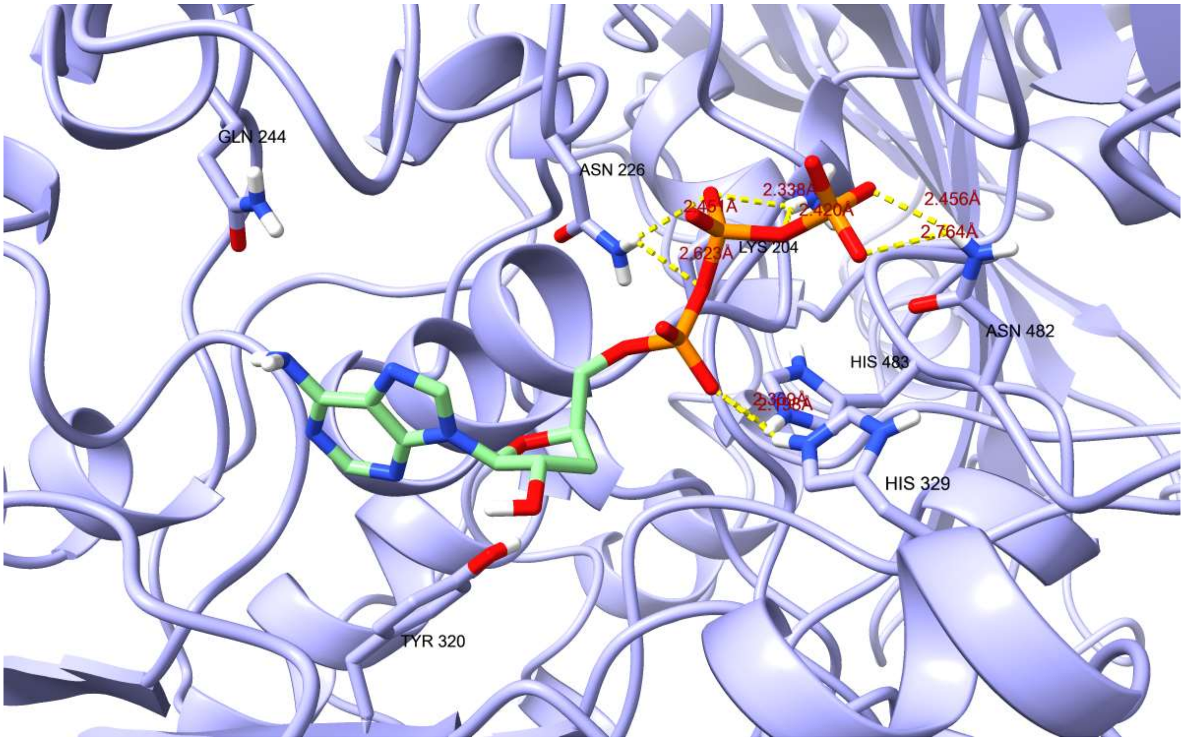

Figure S127. Predicted binding mode of COR-TP with ENPP3.

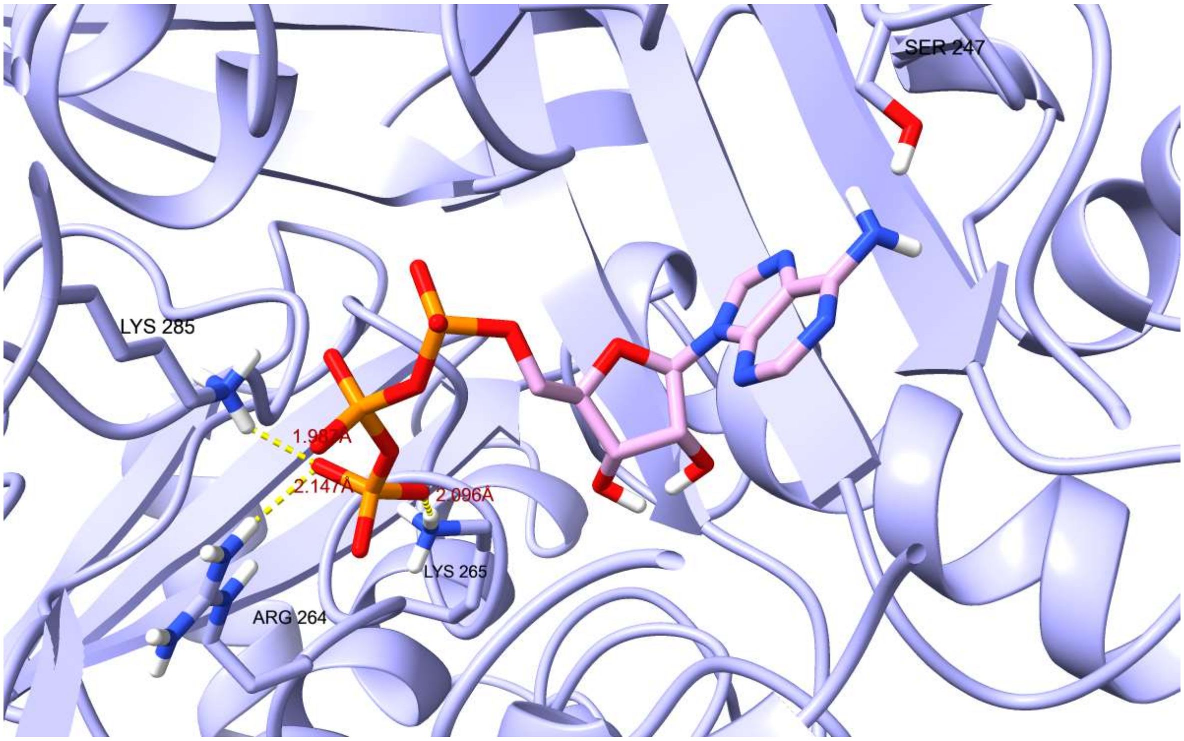

Figure S128. Predicted binding mode of ATP with MAT.

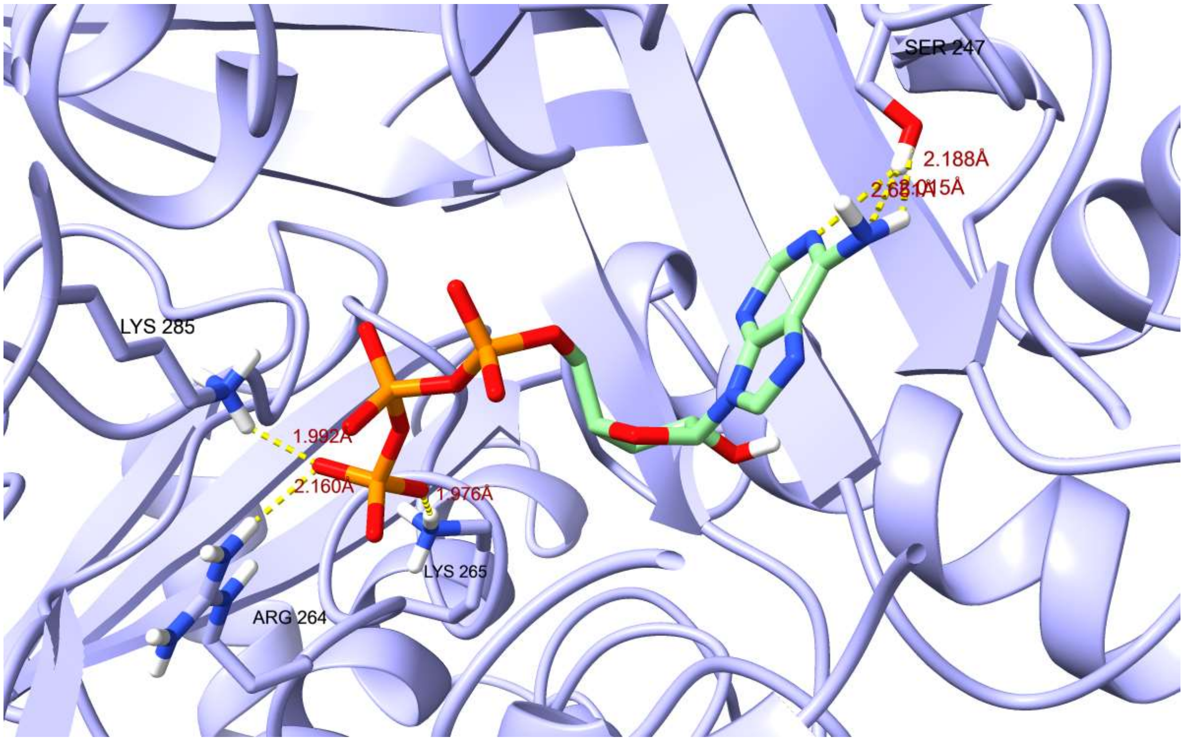

Figure S129. Predicted binding mode of COR-TP with MAT.

Binding modes of biguanides and candidate metabolites in the docked target according to SwissDock.

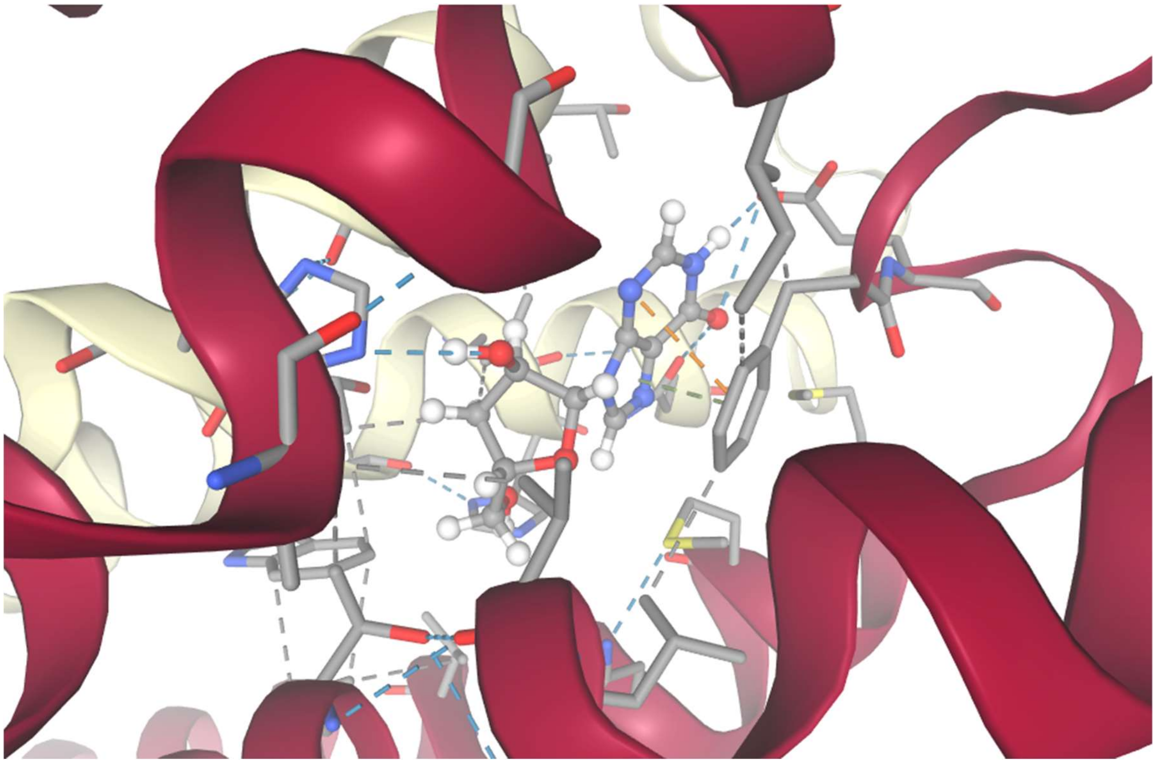

Figure S130. Predicted binding mode of 3’-dINO with A_1R.

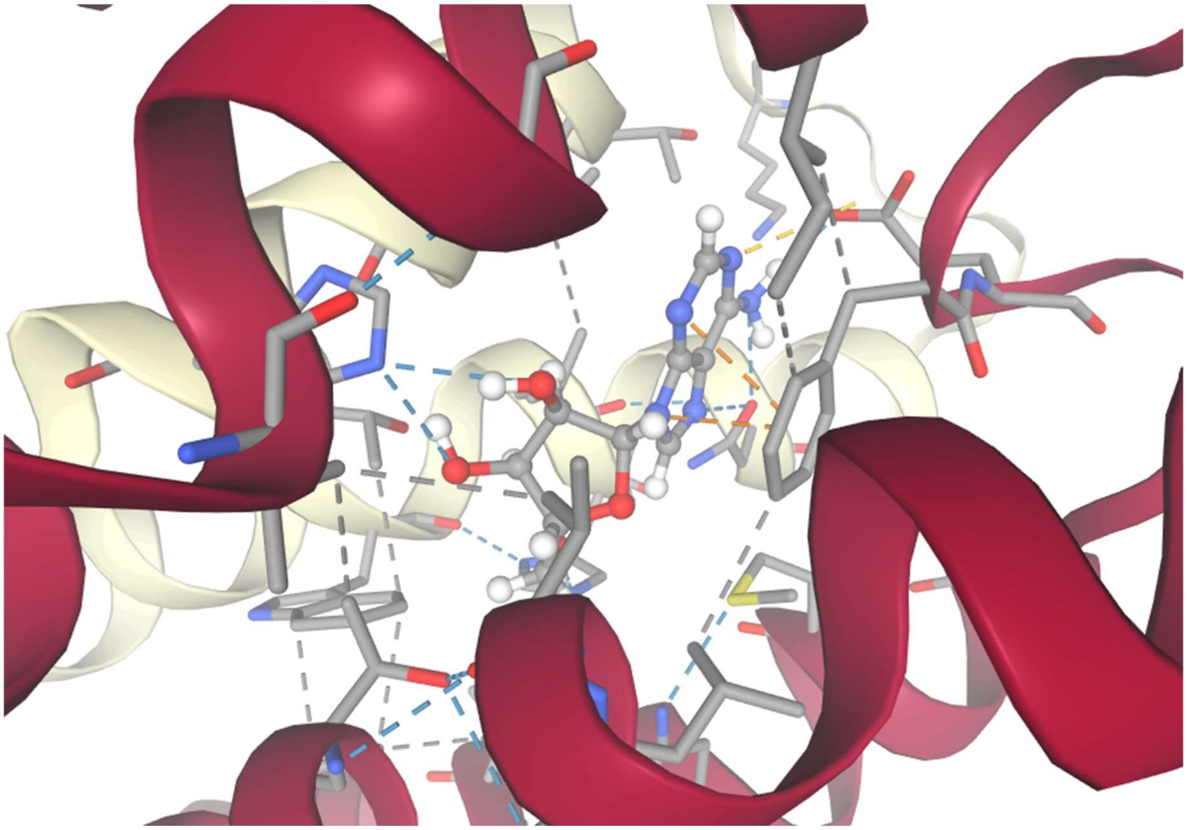

Figure S131. Predicted binding mode of ADO with A_1R.

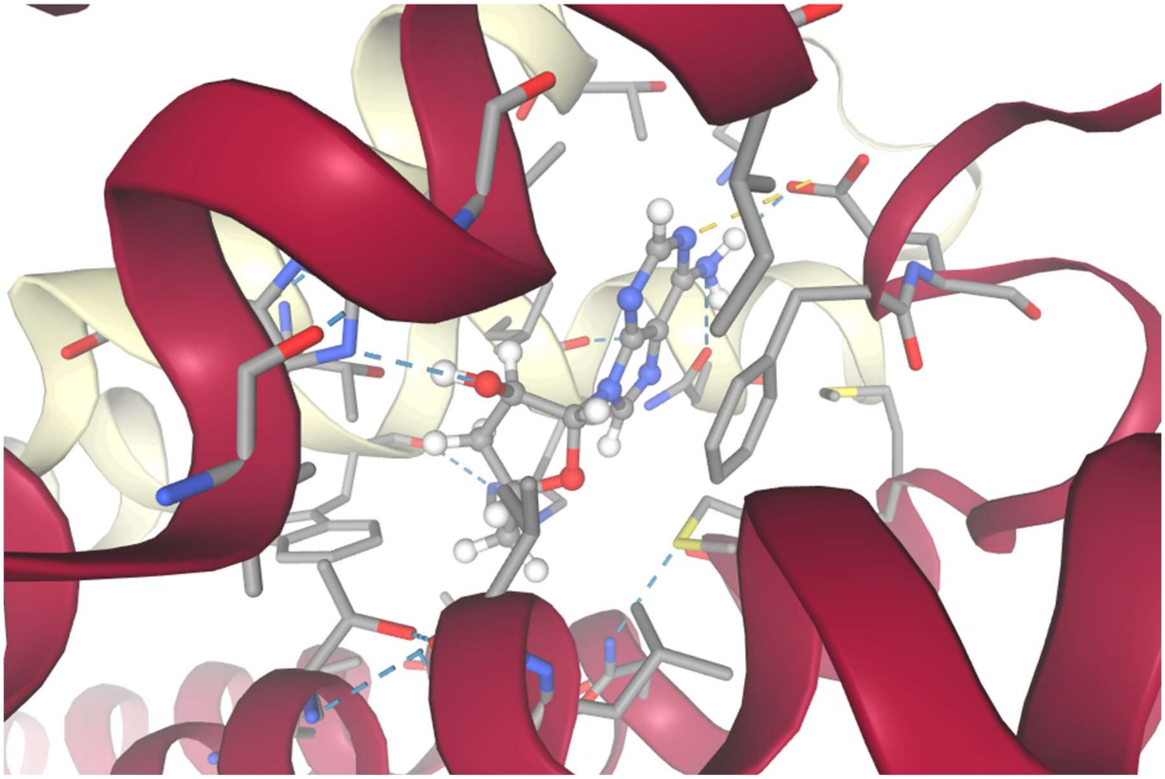

Figure S132. Predicted binding mode of COR with A_1R.

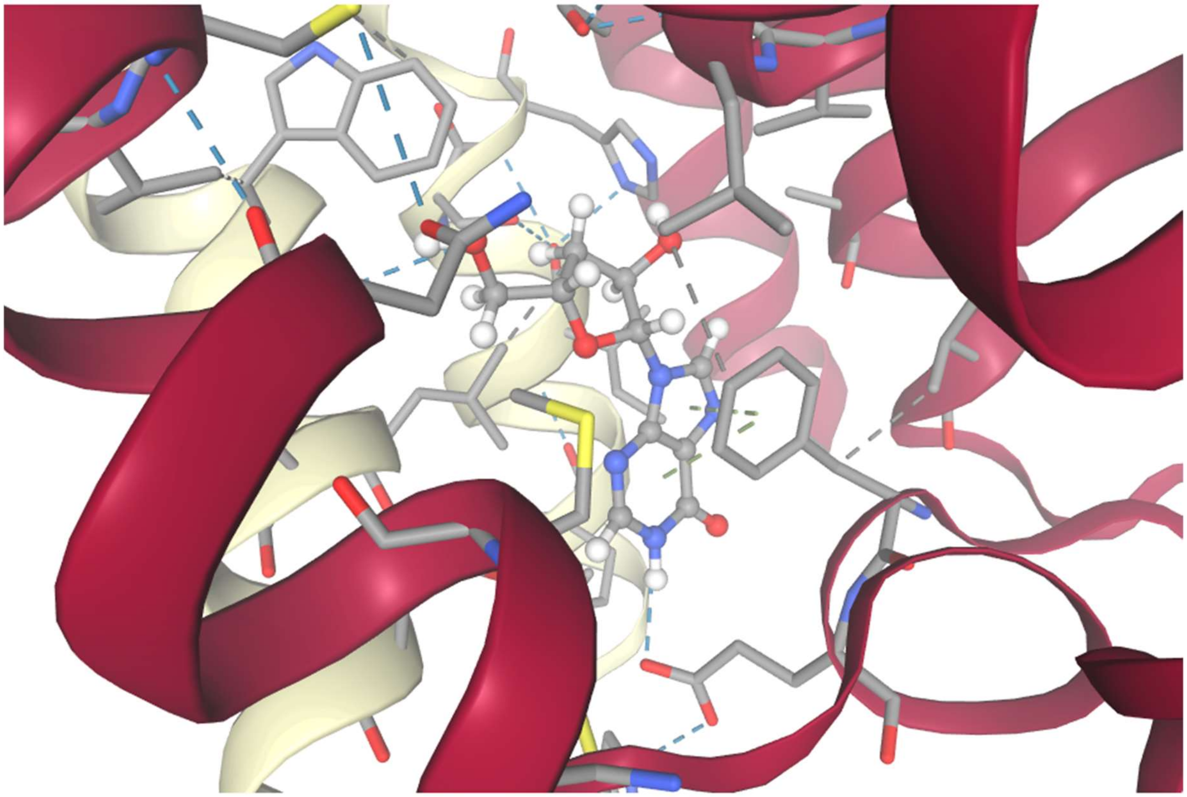

Figure S133. Predicted binding mode of 3’-dINO with $A_{2A}R$.

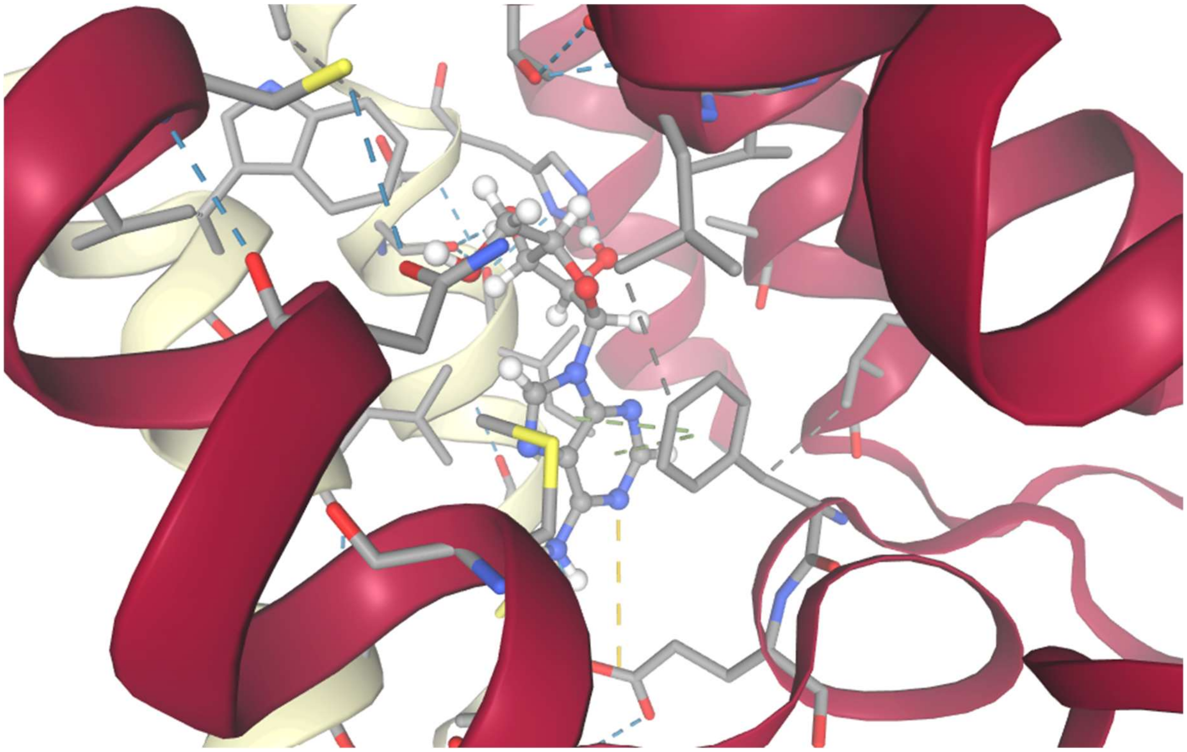

Figure S134. Predicted binding mode of ADO with $A_{2A}R$.

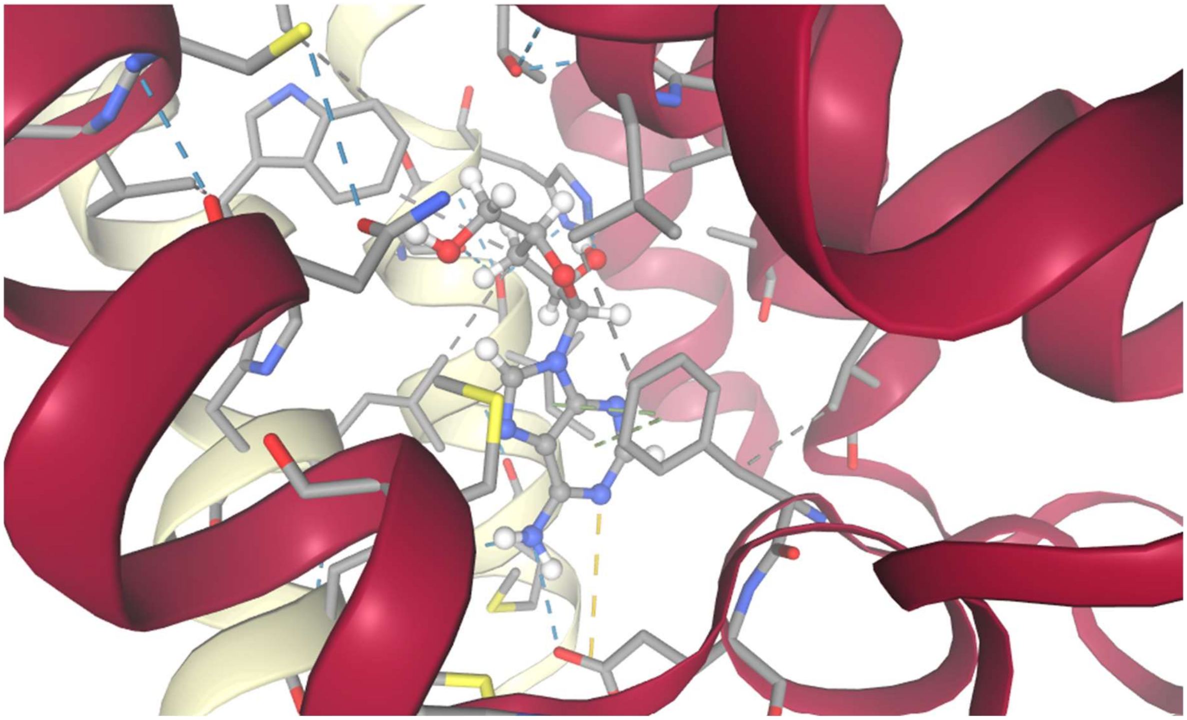

Figure S135. Predicted binding mode of COR with $A_{2A}R$.

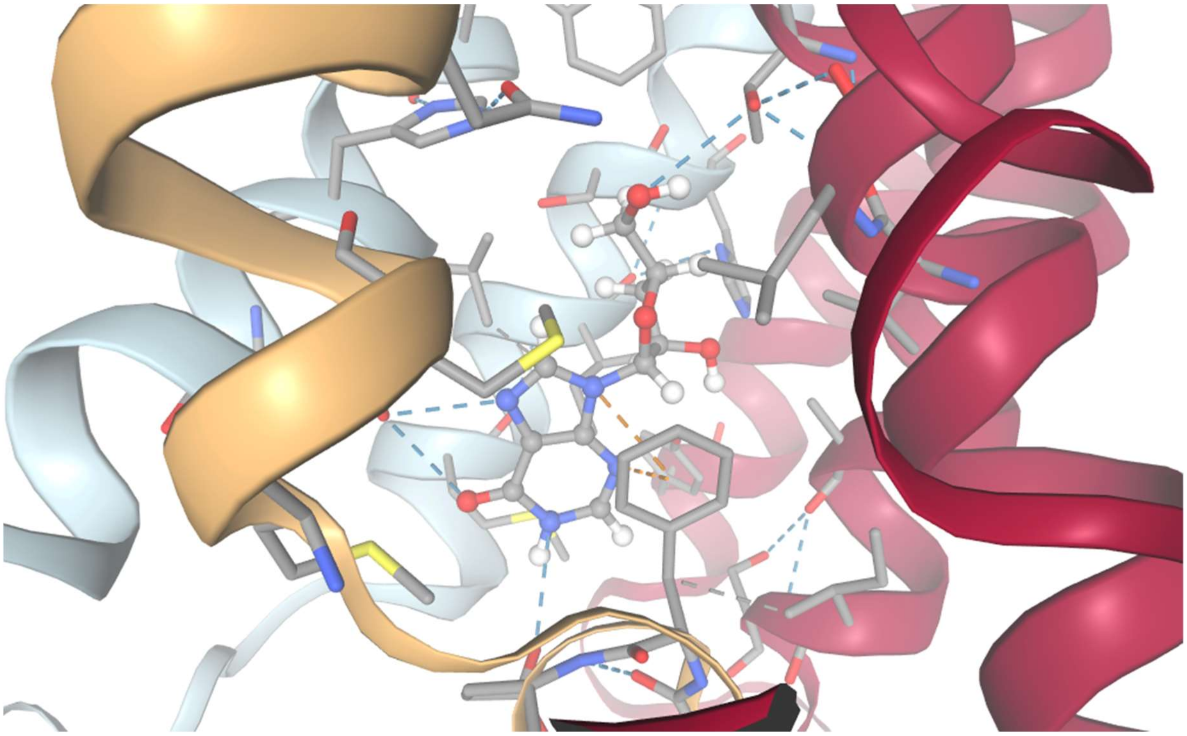

Figure S136. Predicted binding mode of 3’-dINO with $A_{2B}R$.

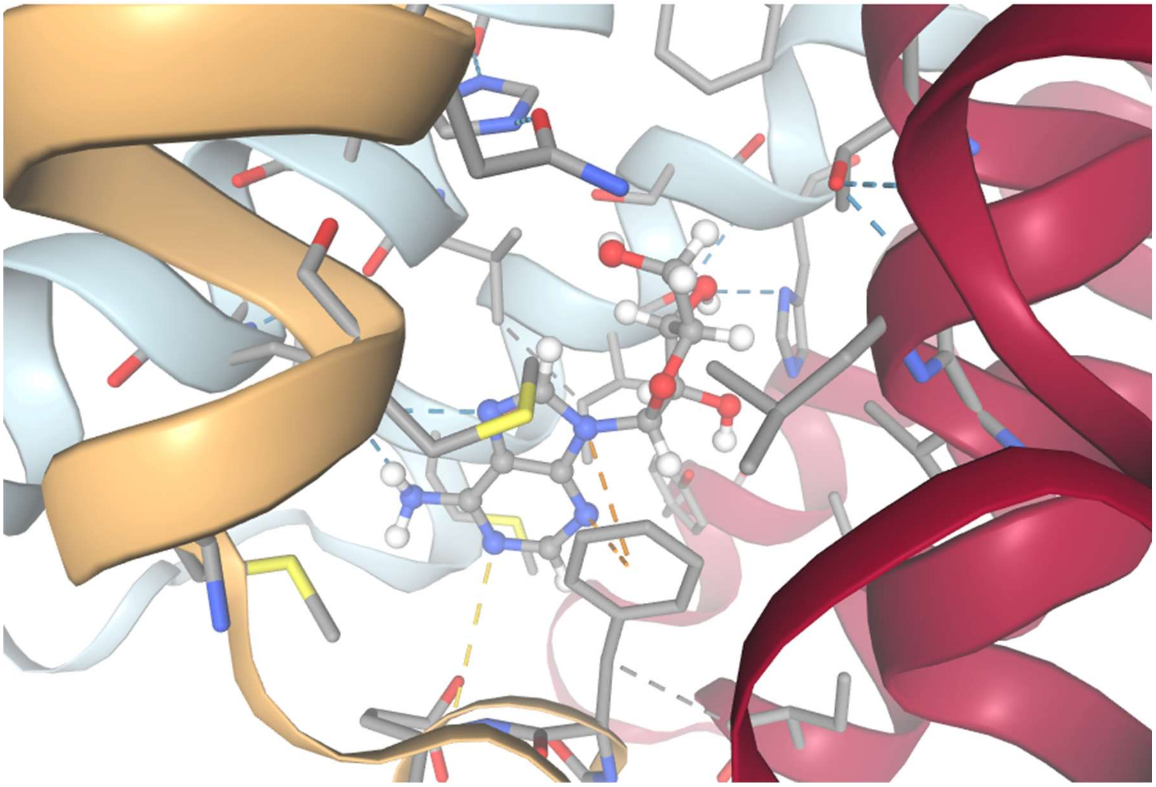

Figure S137. Predicted binding mode of ADO with $A_{2B}R$.

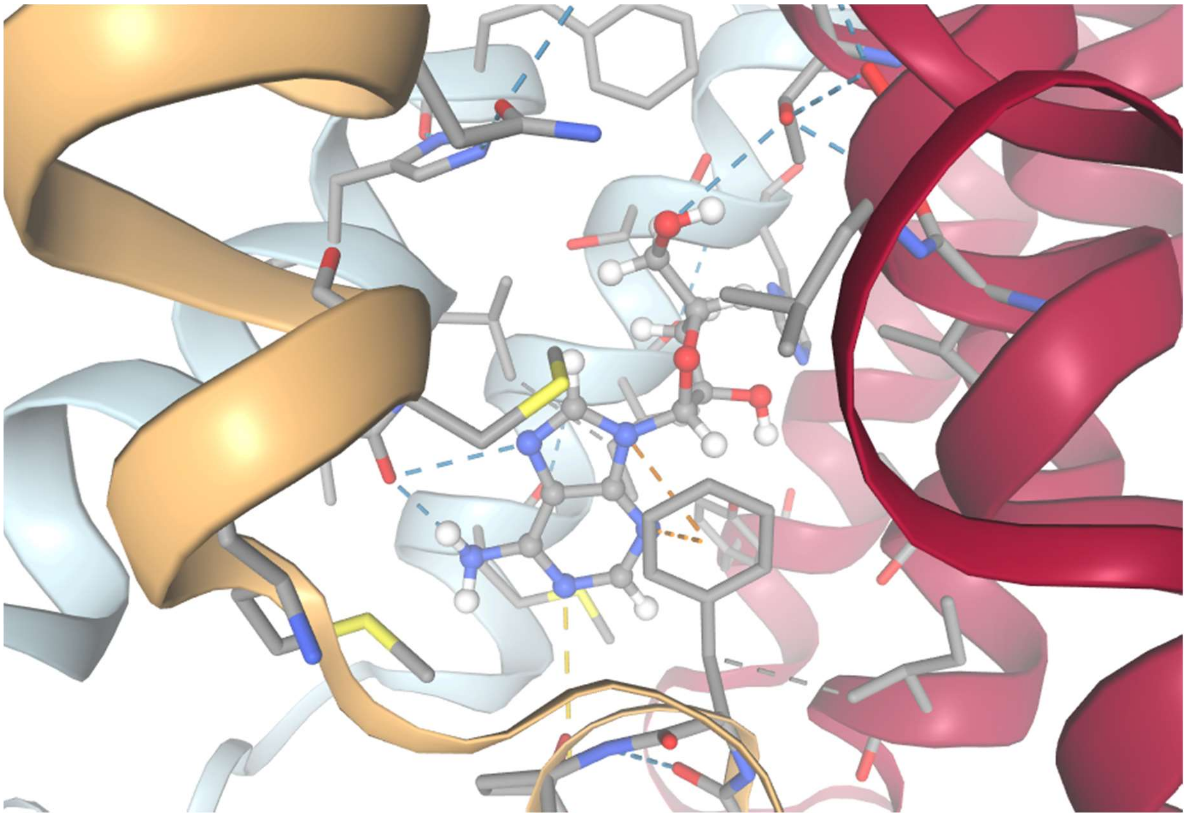

Figure S138. Predicted binding mode of COR with $A_{2B}R$.

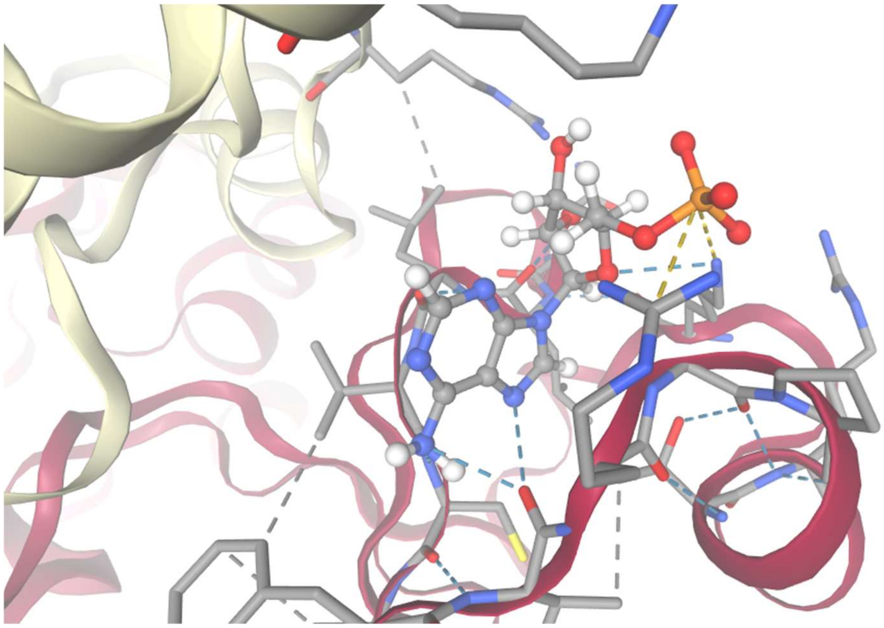

Figure S139. Predicted binding mode of AMP with NT5C2 (site 1).

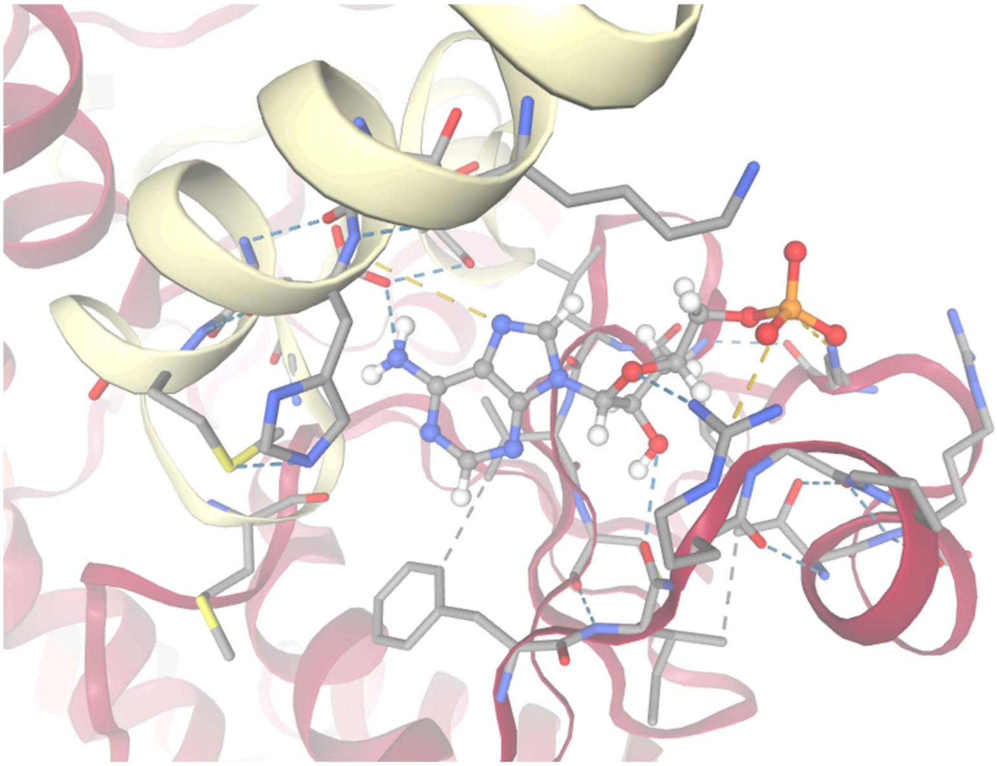

Figure S140. Predicted binding mode of COR-MP with NT5C2 (site 1).

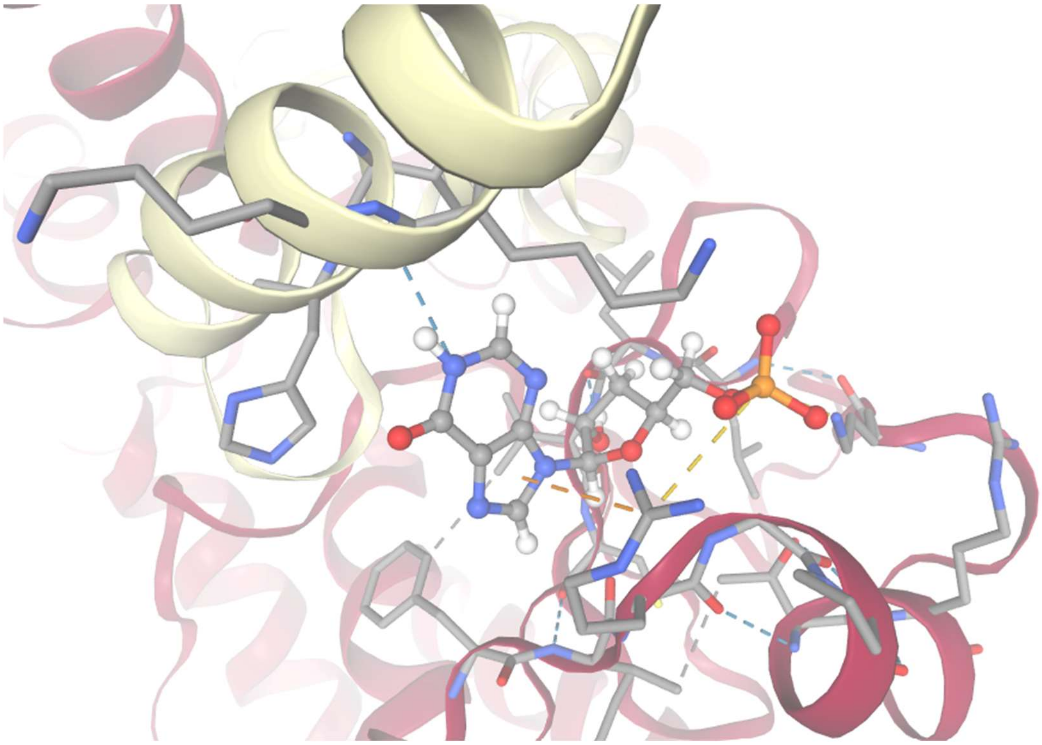

Figure S141. Predicted binding mode of 3’-dIMP with NT5C2 (site 1).

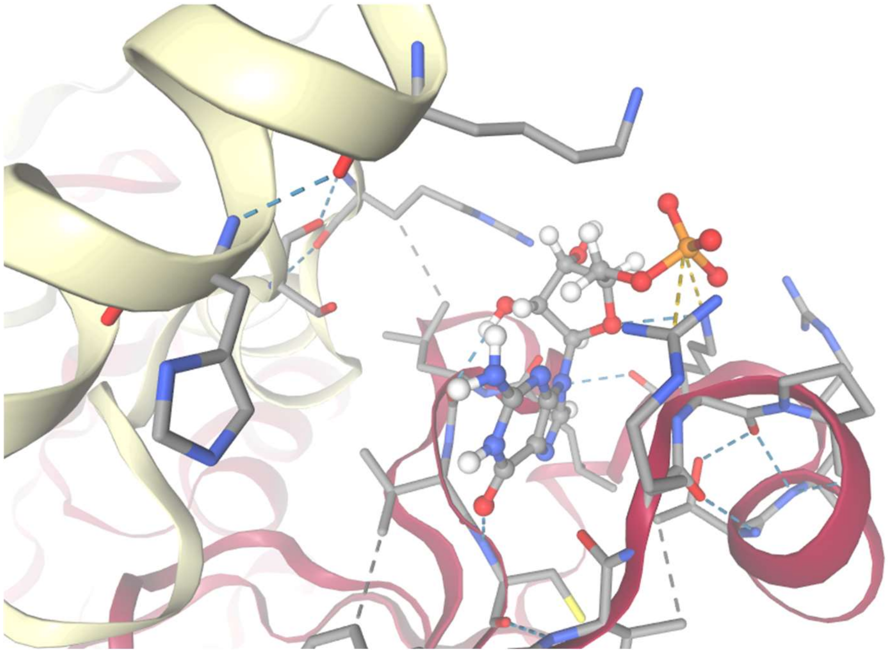

Figure S142. Predicted binding mode of GMP with NT5C2 (site 1).

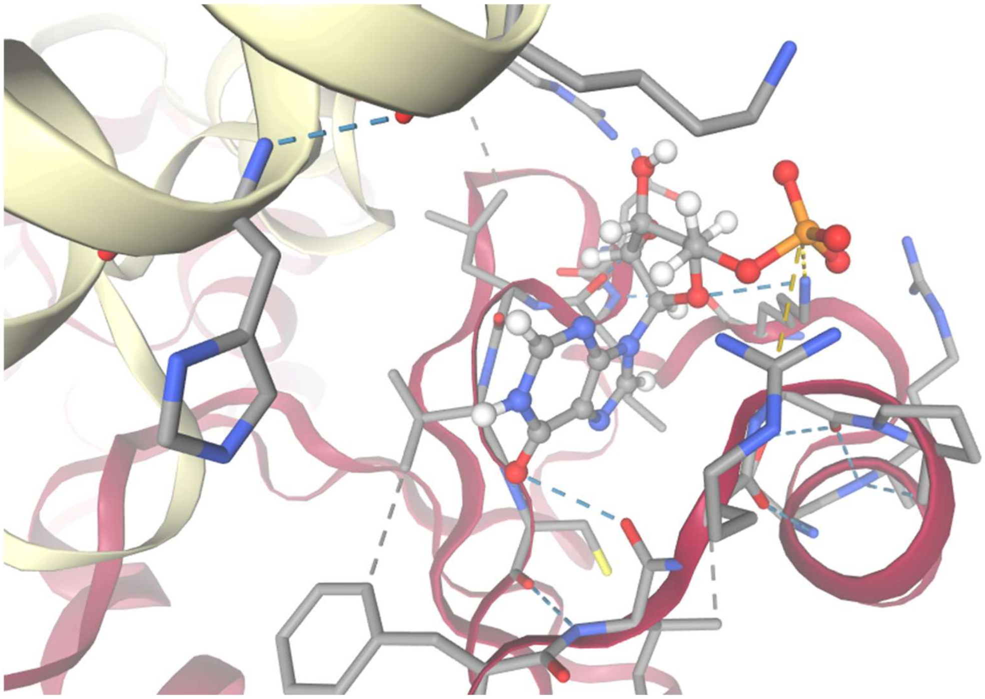

Figure S143. Predicted binding mode of IMP with NT5C2 (site 1).

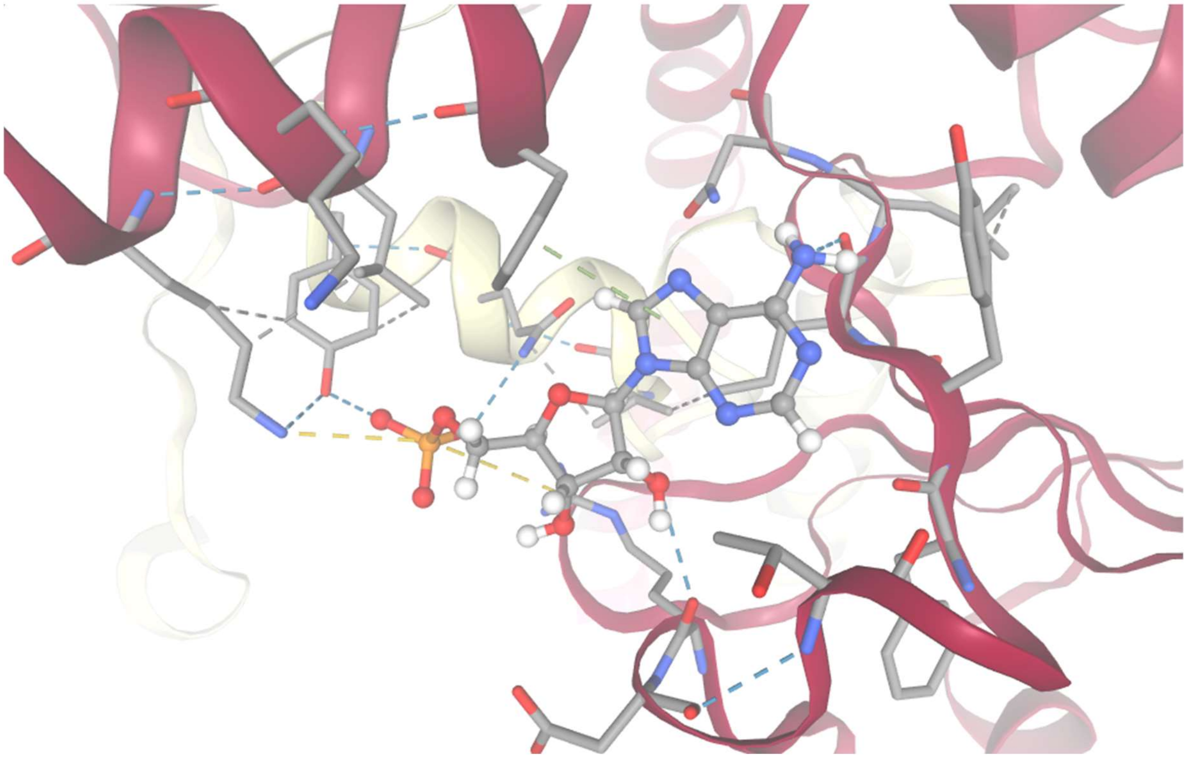

Figure S144. Predicted binding mode of AMP with NT5C2 (site 2).

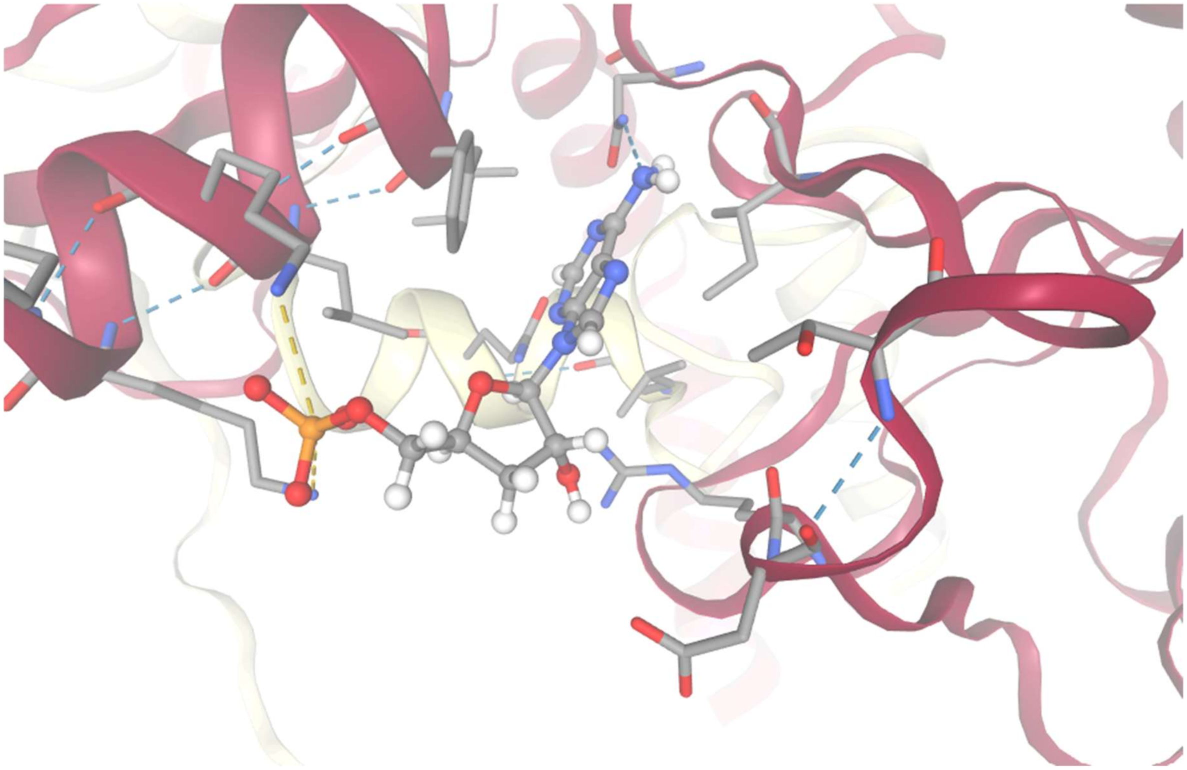

Figure S145. Predicted binding mode of COR-MP with NT5C2 (site 2).

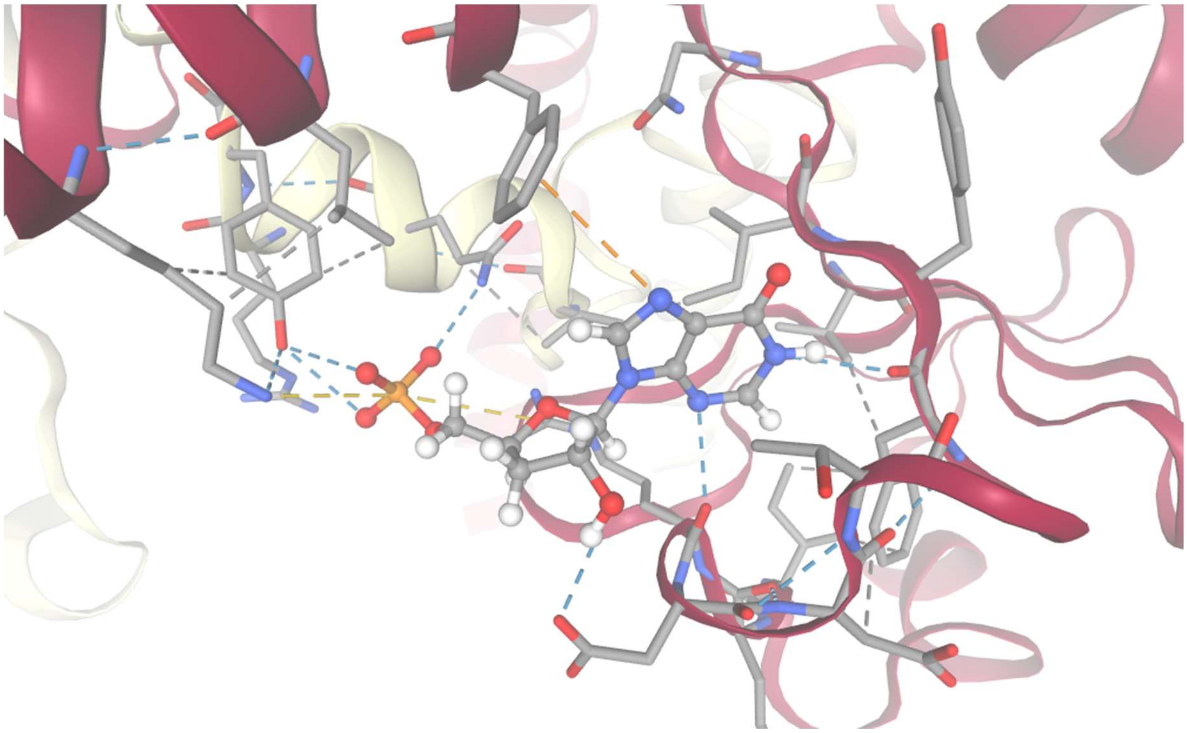

Figure S146. Predicted binding mode of 3’-dIMP with NT5C2 (site 2).

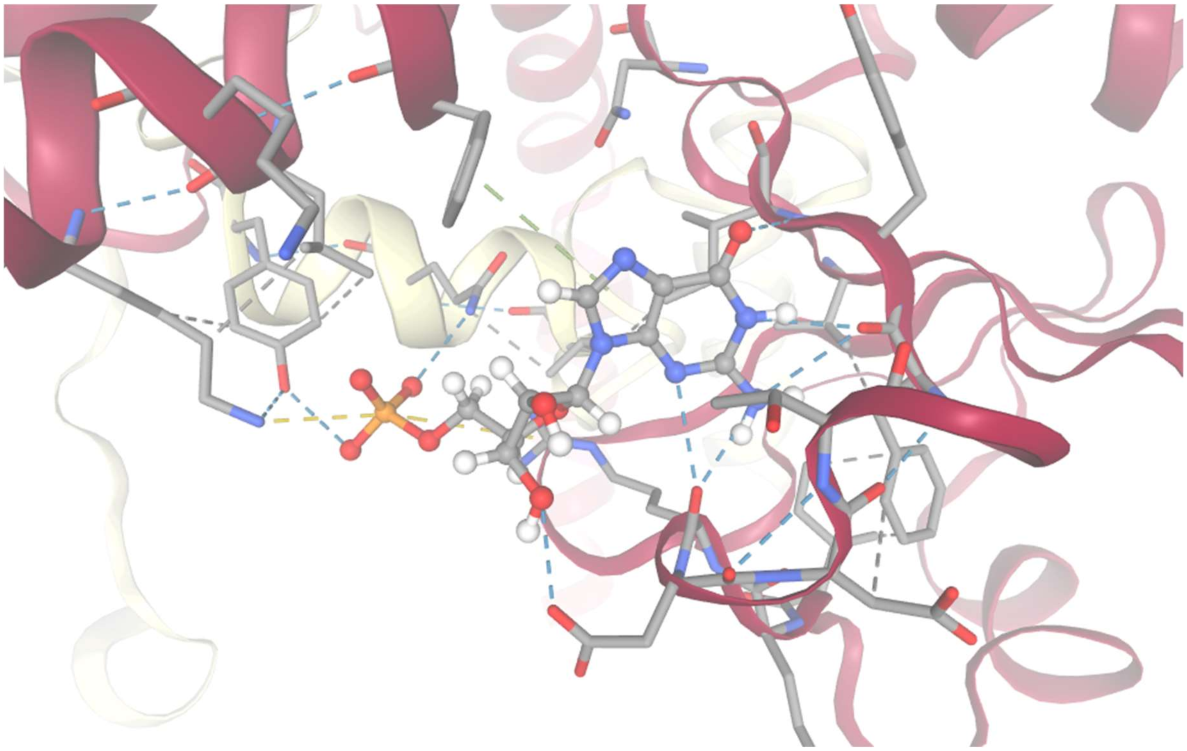

Figure S147. Predicted binding mode of GMP with NT5C2 (site 2).

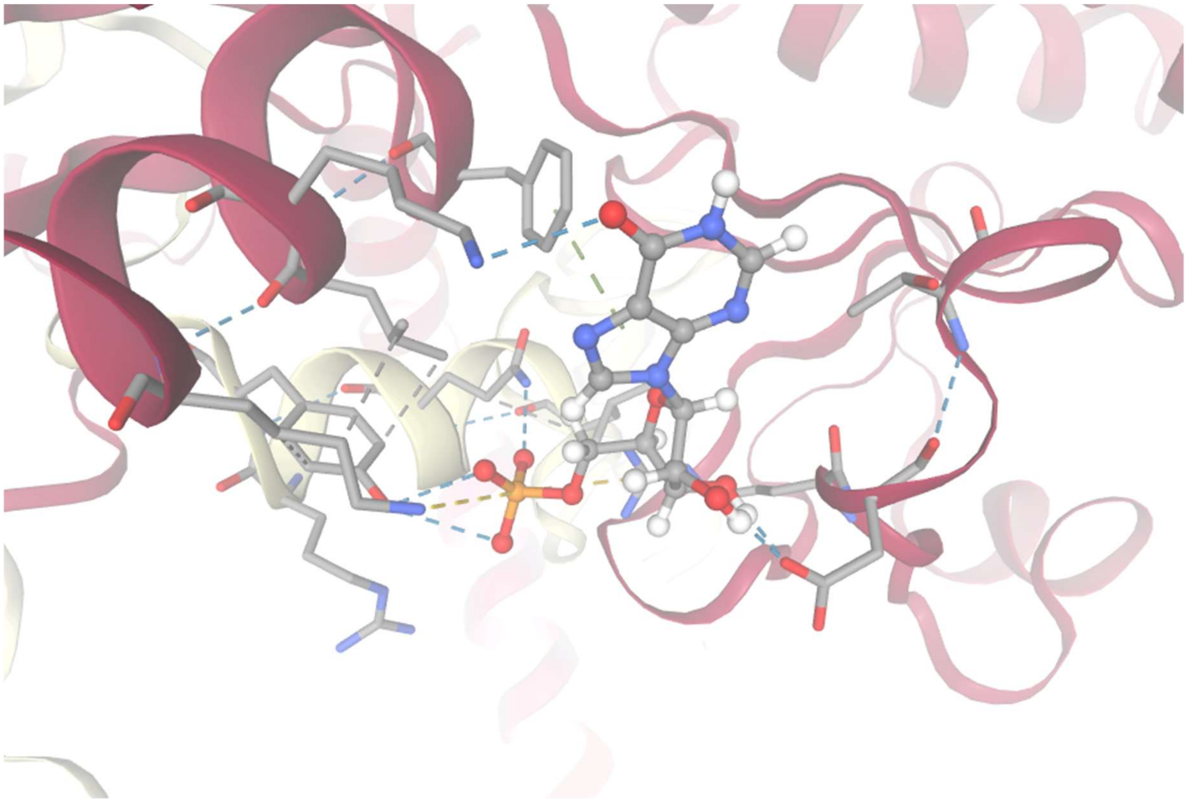

Figure S148. Predicted binding mode of IMP with NT5C2 (site 2).

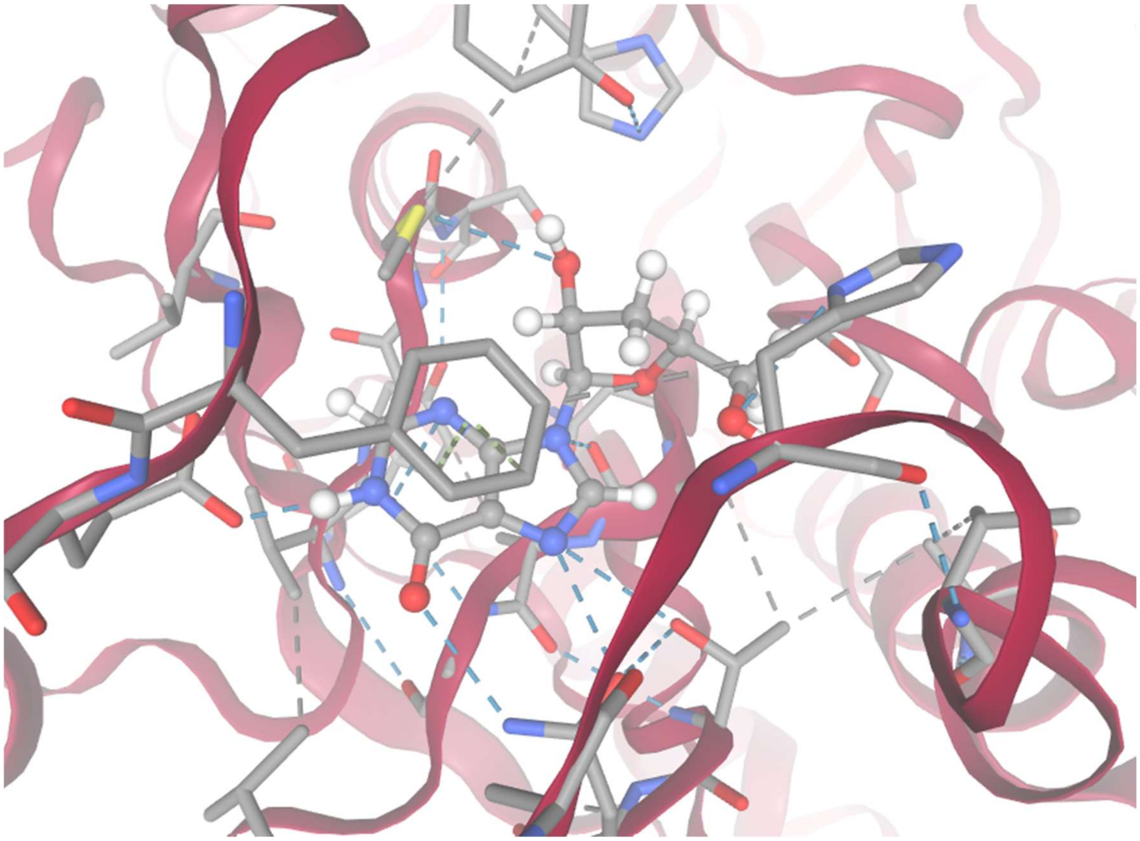

Figure S149. Predicted binding mode of 3’-dINO with PNP.

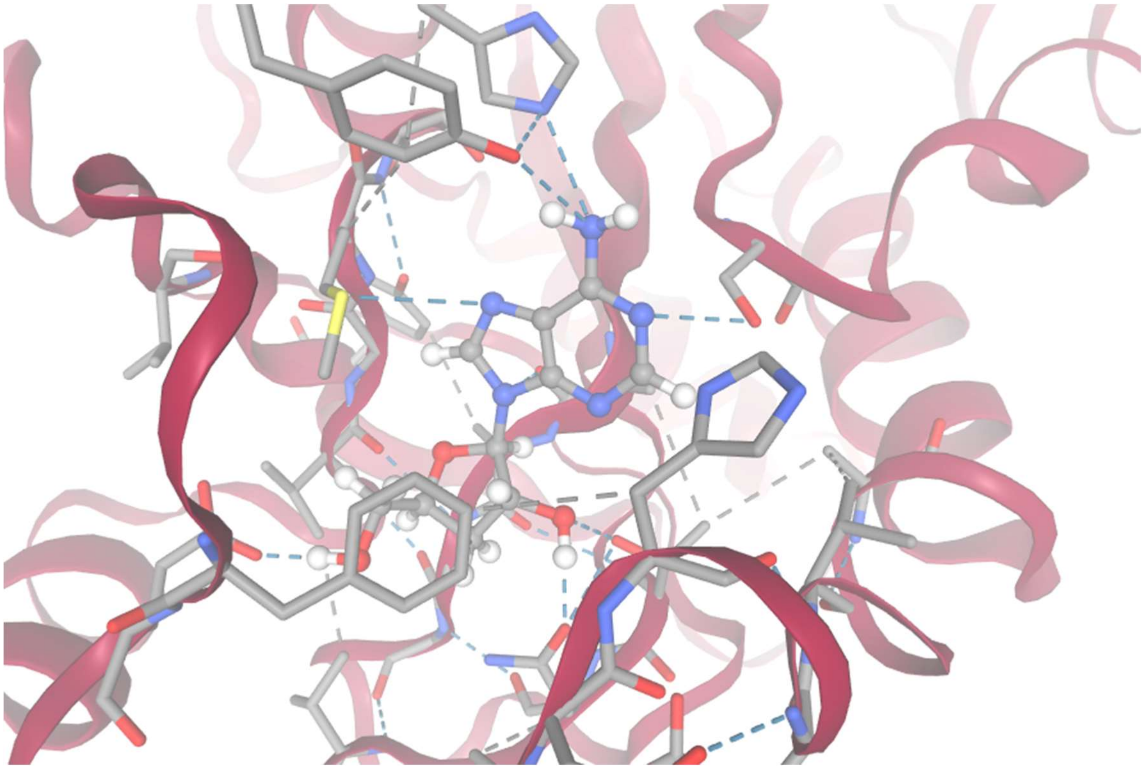

Figure S150. Predicted binding mode of COR with PNP.

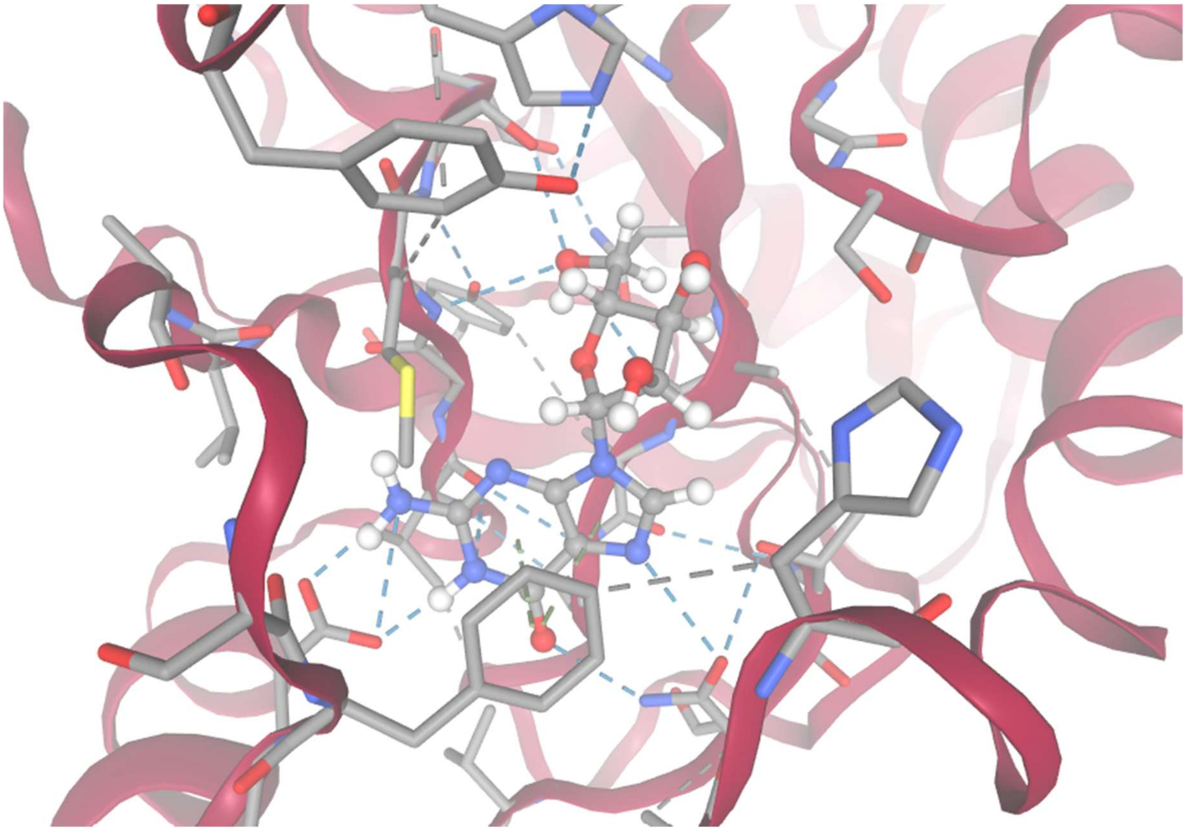

Figure S151. Predicted binding mode of GUA with PNP.

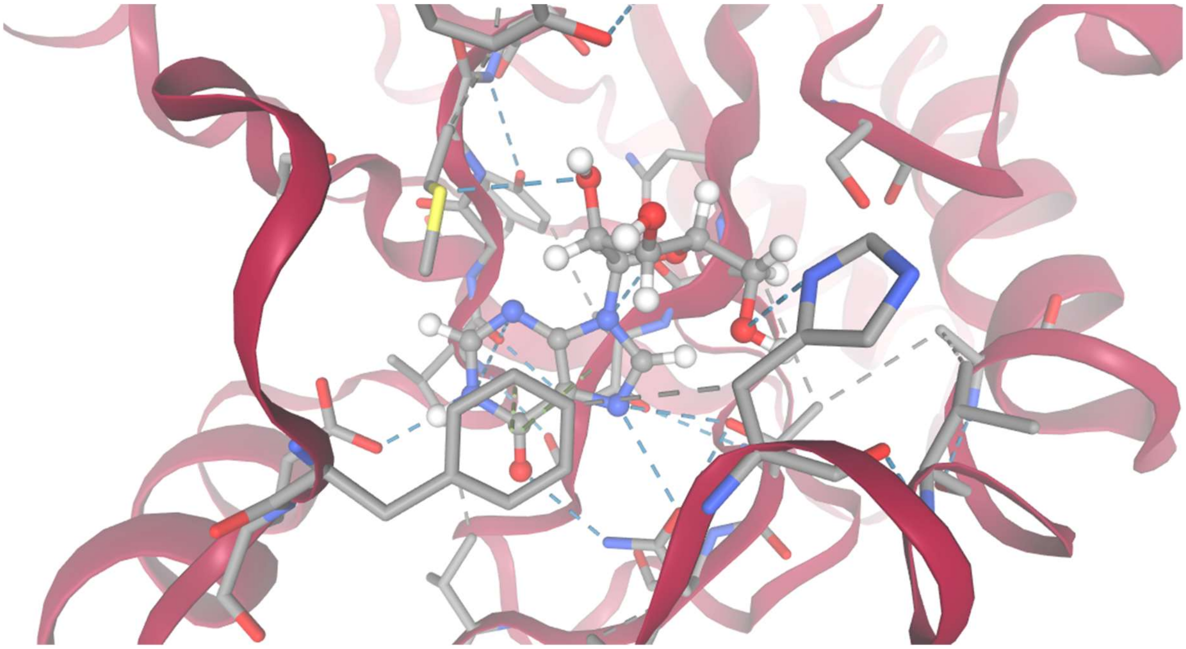

Figure 152. Predicted binding mode of INO with PNP.

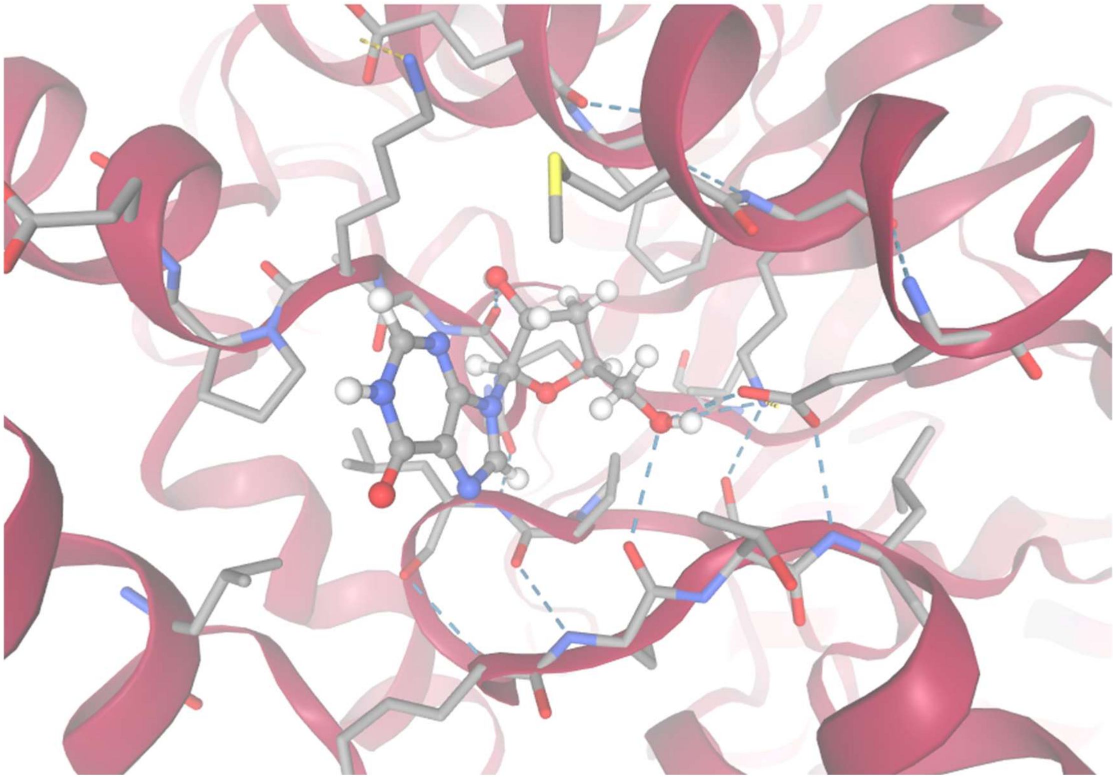

Figure S153. Predicted binding mode of 3’-dINO with ADA.

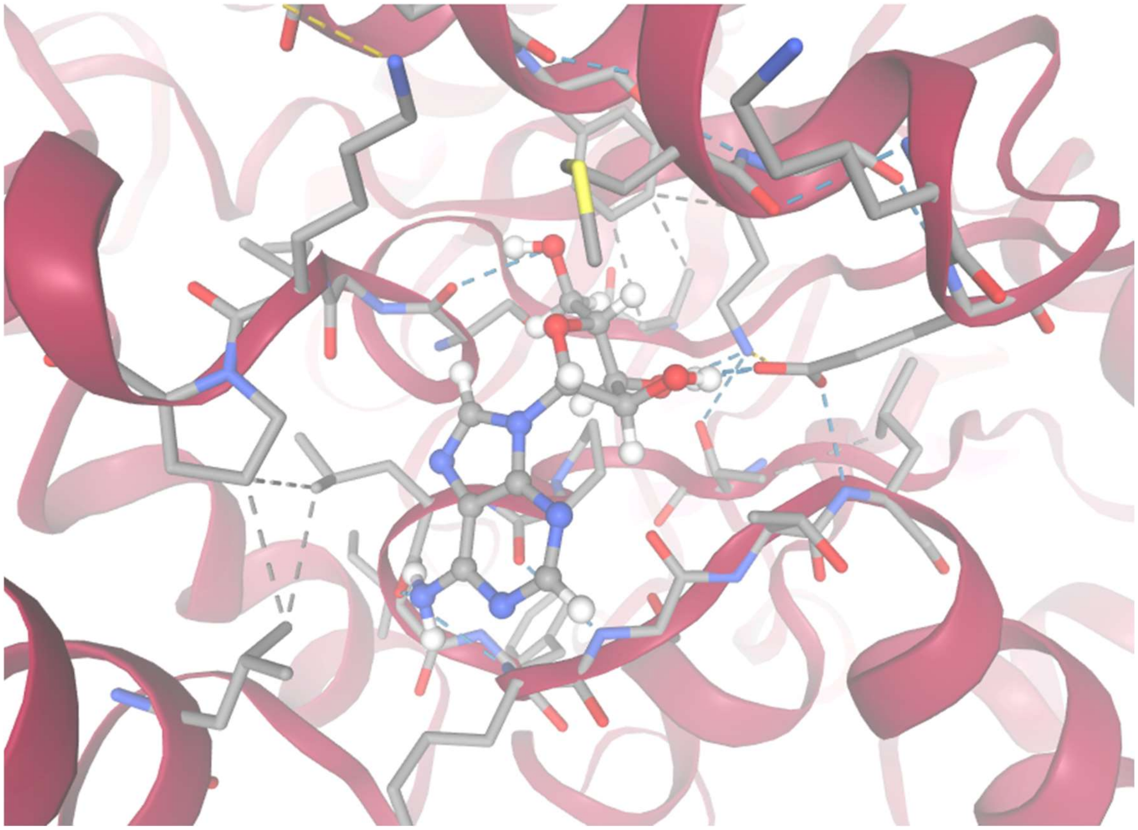

Figure S154. Predicted binding mode of ADO with ADA.

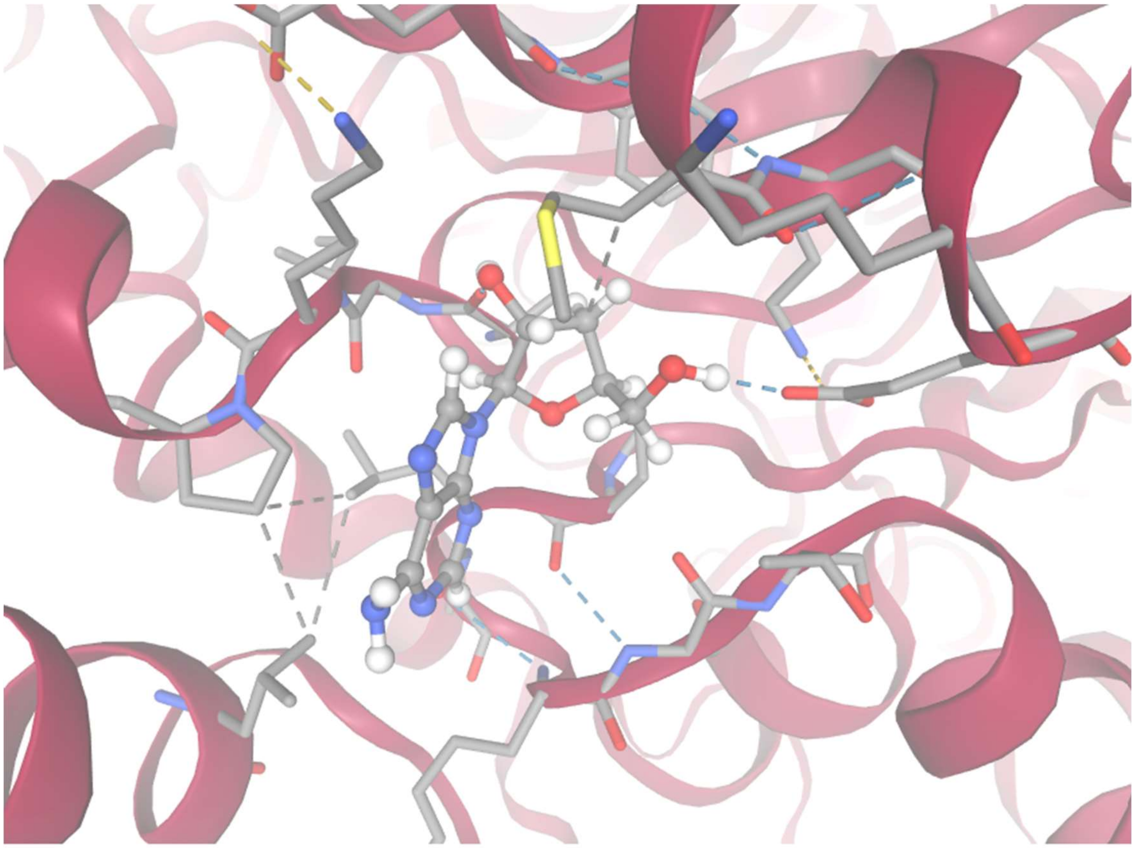

Figure S155. Predicted binding mode of COR with ADA.

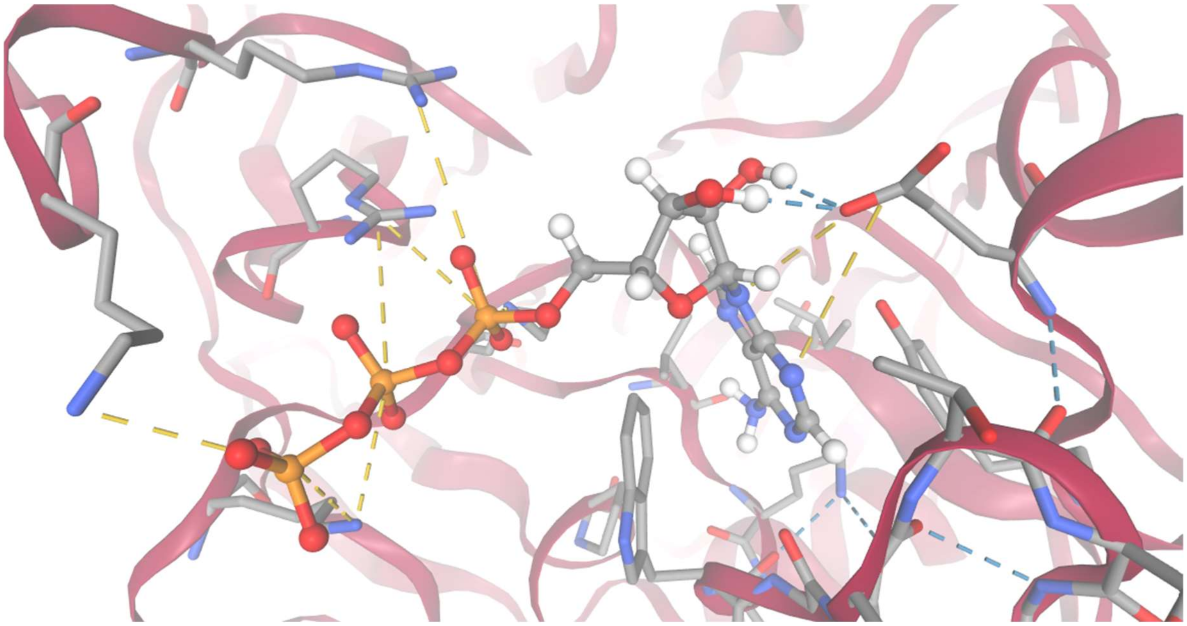

Figure S156. Predicted binding mode of ATP with CD39.

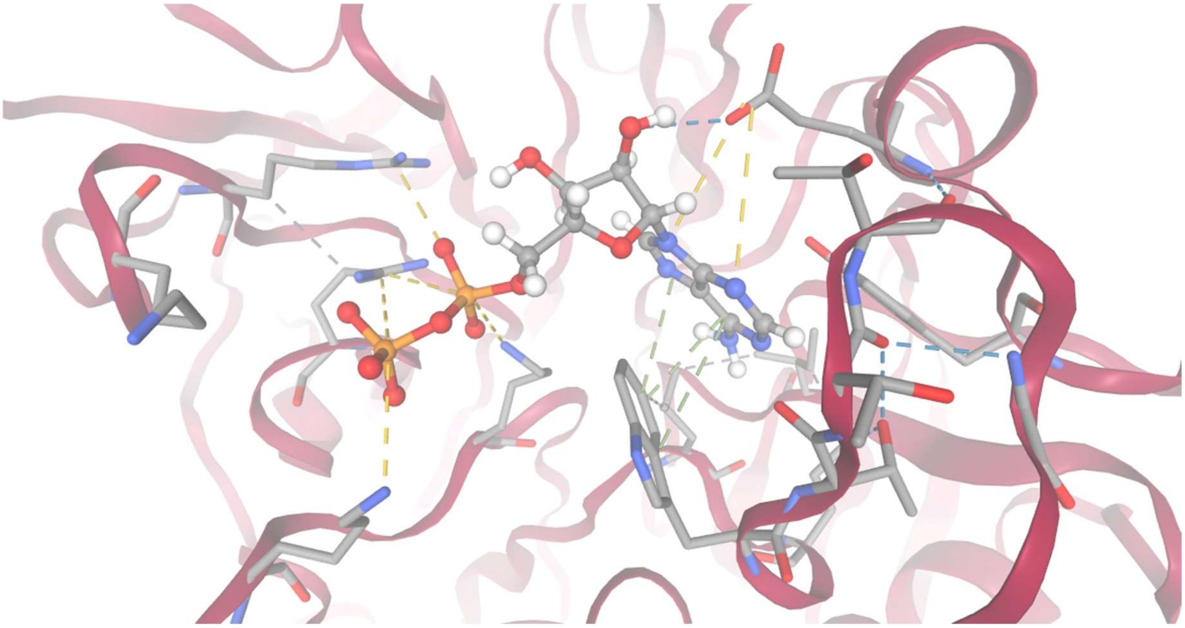

Figure S157. Predicted binding mode of ADP with CD39.

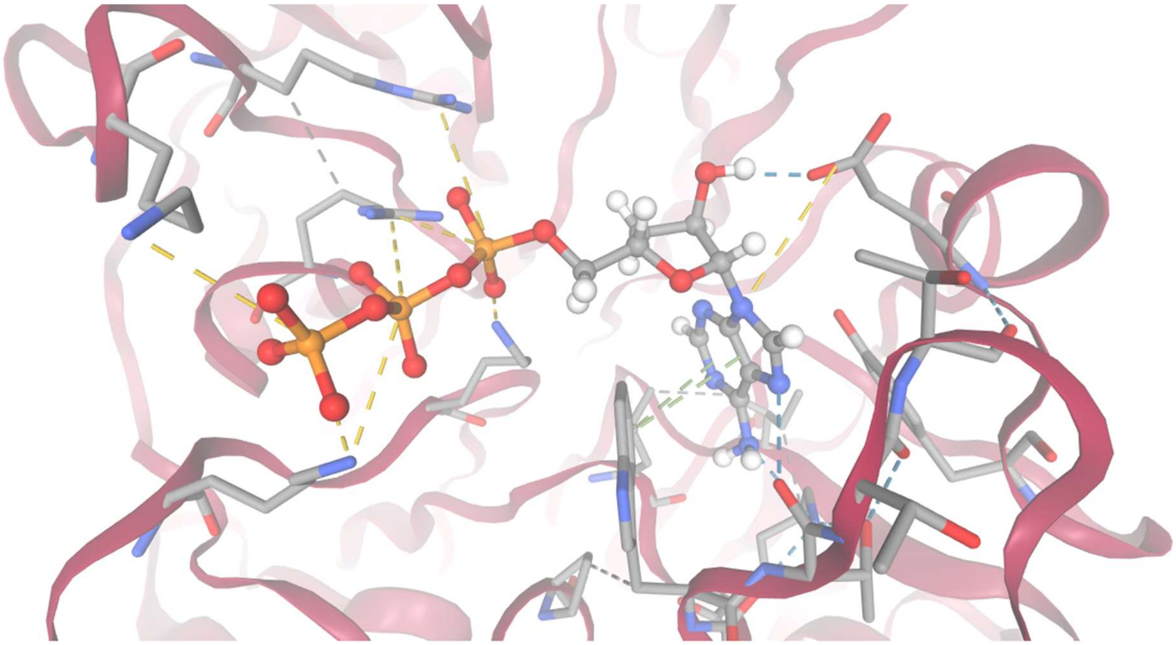

Figure S158. Predicted binding mode of COR-TP with CD39.

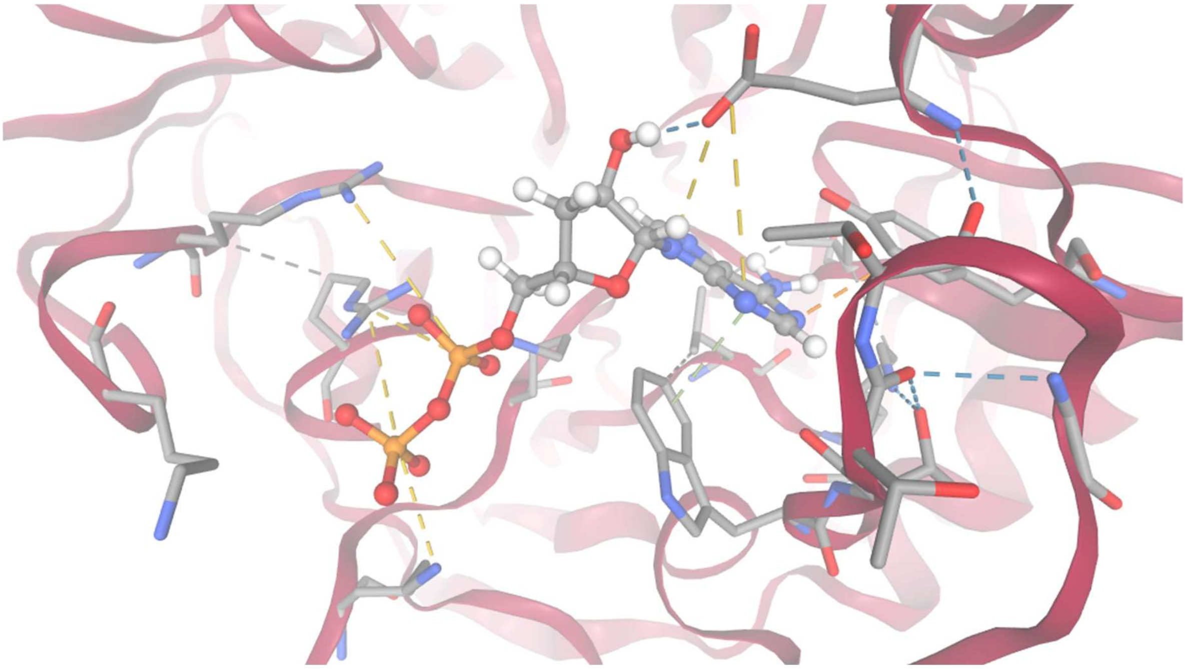

Figure S159. Predicted binding mode of COR-DP with CD39.

S5. Recommended readings to deepen the work related to the modulation of the different effectors in cAMP-mediated signaling.

Article	Findings/Observations	Reference
A novel Epac-specific inhibitor suppresses pancreatic cancer cell migration and invasion	ESI-09 (an EPAC inhibitor) inhibits pancreatic cancer cell migration and EPAC-mediated insulin secretion in INS-1 cells, demonstrating its ability to suppress EPAC-mediated cellular functions.	[1]
CRISPR/Cas9 mediated deletion of the adenosine a2a receptor enhances CAR T cell efficacy.	The removal of A2AR in CAR T cells makes them resistant to adenosine-induced transcriptional changes, resulting in increased production of cytokines such as IFNγ and TNF.	[2]
Identification of Novel Epac2 Antagonists through in Silico and in Vitro Analyses	Computational analyses and laboratory experiments were used to evaluate the efficacy of the identified compounds. The compounds found showed potential as Epac2 antagonists.	[3]
MHY4571, a novel Diarylcyclohexanone derivative, exerts anti-cancer activity by regulating the PKA-cAMP-response element-binding protein pathway in squamous cell lung cancer	MHY4571 inhibited PKA activity and reduced the viability of lung cancer cells, promoting caspase-3-dependent apoptotic cell death.	[4]
In vitro and in vivo reversal of MDR1-mediated multidrug resistance by KT-5720: implications on hematological malignancies	KT-5720 (a PKA inhibitor) significantly reversed MDR1-dependent resistance in primary malignant cells from patients with chronic myeloid leukemia in blastic crisis (CML-BC) and advanced multiple myeloma (MM).	[5]

PKA catalytic subunit is a molecular switch that promotes the pro-tumoral function of macrophages	The overactivation of PKA in tumor-associated macrophages (TAMs) creates a microenvironment that favors cancer progression, inducing the expression of factors such as VEGFA, IL-10, and arginase 1 (ARG1).	[6]
Protein Phosphatase 1 Regulatory Subunit 1A in Ewing Sarcoma tumorigenesis and metastasis	PPP1R1A depletion resulted in a significant decrease in oncogenic transformation and cell migration in vitro, as well as xenograft tumor growth and metastasis in a mouse model	[7]
Targeting CREB pathway suppresses small-cell lung cancer	The study explores the role of the NF-κB and CREB families of transcription factors in the SCLC, finding that NF-κB activity is dispensable while suppression of CREB activity halts the development of the SCLC.	[8]
Breast cancer cell migration is regulated through junctional adhesion molecule-a-mediated activation of Rap1-GTPase	Evidence for a Novel Role of JAM-A in Promoting Breast Cancer Cell Migration Through Activation of Rap1 GTPase and β1-Integrin	[9]
Unbiased proteomic profiling uncovers a targetable GNAS/PKA/PP2A axis in small-cell lung cancer stem cells	The study identifies the GNAS/PKA/PP2A pathway as a growth promoter of small cell lung cancer (SCLC) and suggests that inhibition of PKA or activation of PP2A could suppress the expansion of SCLC.	[11]
Cancer cell employs a microenvironmental neural signal trans-activating nucleus-mitochondria coordination to acquire stemness	This study delves into the signaling mediated by neurotransmitters, such as norepinephrine, which can enhance the capacity of nearby cancer cells by activating the cAMP-CRE axis.	[12]
Cancer cells become less deformable and more invasive with activation of β-adrenergic signaling	The study focuses on how the activation of β-adrenergic signaling in breast cancer cells makes them less deformable and more invasive by affecting cellular deformability through the actin cytoskeleton and myosin II activity.	[13]
Modulation of A1 and A2B adenosine receptor activity: a new strategy to sensitize glioblastoma stem cells to chemotherapy	The A1AR and A2BAR agonists show an anti-proliferative/apoptotic effect on CSCs, and the A1AR agonist also promotes the differentiation of CSCs into a glial phenotype.	[14]
The adenosine-A_{2A} receptor regulates the radioresistance of gastric cancer via PI3K-AKT-mTOR pathway	ADO-A2AR regulates GC cell stemness, which leads to radioresistance. A2aR inhibition improves the radiosensitivity of GC cells.	[15]
Cellular proliferation in prostate cancer cells is mediated by B-RAF/ERK and mTOR signaling cascades	Is discussed how activation of EPAC1 by cAMP, independent of PKA, can stimulate cell proliferation in prostate cancer cells via the B-Raf/ERK and mTOR signaling cascades	[16]
A novel interplay between Rap1 and PKA regulates induction of angiogenesis in prostate cancer	This study provides evidence of a novel interaction between Rap1, Epac, and PKA that regulates the induction of angiogenesis in the tumor microenvironment	[17]
Evidence for a pro-proliferative feedback loop in prostate cancer: the role of Epac1 and COX-2-dependent pathways.	Epac1 acts as a pro-inflammatory modulator in prostate cancer cells and promotes cell proliferation and survival by boosting Ras-MAPK and PI3K-Akt-mTOR signals.	[18]

S6. Recommended readings related to molecular mechanisms of effectors in cAMP signaling.

Article	Findings/Observations	Reference
CpG island methylation of the Rap1GAP gene in medullary thyroid cancer	A high methylation rate was found in medullar thyroid cancer samples that correlate with low expression of the Rap1Gap gene.	[19]
Evaluation of Rap1GAP and EPAC1 gene expression in endometriosis disease	EPAC1 was found to be overexpressed in ectopic tissues compared to eutopic and control tissues, while Rap1GAP expression was lower in ectopic tissues.	[20]
Down-regulation of Rap1GAP via promoter hypermethylation promotes melanoma cell proliferation, survival, and migration	The decrease in Rap1GAP is due to the methylation of its promoter, a gene-silencing mechanism in tumors. Treatment with the demethylating agent 5-aza-2'-deoxycytidine reduces the expression of Rap1GAP	[21]
Rap1GAP inhibits tumor progression in endometrial cancer	CAD cell lines were used to examine the levels of Rap1GAP and its effect on cancer cell invasion and migration1. Low expression of Rap1GAP was found to be related to poorer EAC differentiation.	[22]
Rap1GAP inhibits tumor growth in oropharyngeal squamous cell carcinoma	Rap1GAP expression in oropharyngeal SCCs reduced active rap1, ERK activation, and cell proliferation and appears to slow progression through the cell cycle by downregulating cyclin D1, CDK4, and CDK6.	[23]

References

1. Almahariq, M.; Tsalkova, T.; Mei, F.C.; Chen, H.; Zhou, J.; Sastry, S.K.; Schwede, F.; Cheng, X. A Novel EPAC-Specific Inhibitor Suppresses Pancreatic Cancer Cell Migration and Invasion. *Mol Pharmacol* **2013**, *83*, 122–128, doi:10.1124/mol.112.080689.

2. Giuffrida, L.; Sek, K.; Henderson, M.A.; Lai, J.; Chen, A.X.Y.; Meyran, D.; Todd, K.L.; Petley, E. V.; Mardiana, S.; Mølck, C.; et al. CRISPR/Cas9 Mediated Deletion of the Adenosine A2A Receptor Enhances CAR T Cell Efficacy. *Nat Commun* **2021**, *12*, 3236, doi:10.1038/s41467-021-23331-5.

3. Qureshi, U.; Khan, M.I.; Ashraf, S.; Hameed, A.; Hafizur, R.M.; Rafique, R.; Khan, K.M.; Ul-Haq, Z. Identification of Novel Epac2 Antagonists through in Silico and in Vitro Analyses. *European Journal of Pharmaceutical Sciences* **2020**, *153*, 105492, doi:10.1016/j.ejps.2020.105492.

4. Chung, J.H.; Choi, H.J.; Kang, Y.J.; Kim, Y.S.; Lee, S.-Y.; Kwon, R.J.; Jeong, H.-S.; Park, S.-J.; Jeong, Y.; Kang, D.; et al. MHY4571, a Novel Diarylcyclohexanone Derivative, Exerts Anti-Cancer Activity by Regulating the PKA-CAMP-Response Element-Binding Protein Pathway in Squamous Cell Lung Cancer. *Exp Hematol Oncol* **2022**, *11*, 68, doi:10.1186/s40164-022-00324-8.

5. Galski, H.; Sivan, H.; Lazarovici, P.; Nagler, A. In Vitro and in Vivo Reversal of MDR1-Mediated Multidrug Resistance by KT-5720: Implications on Hematological Malignancies. *Leuk Res* **2006**, *30*, 1151–1158, doi:10.1016/j.leukres.2006.02.016.

6. Na, Y.R.; Kwon, J.W.; Kim, D.Y.; Chung, H.; Song, J.; Jung, D.; Quan, H.; Kim, D.; Kim, J.-S.; Ju, Y.W.; et al. Protein Kinase A Catalytic Subunit Is a Molecular Switch That Promotes the Pro-Tumoral Function of Macrophages. *Cell Rep* **2020**, *31*, 107643, doi:10.1016/j.celrep.2020.107643.

7. Luo, W.; Xu, C.; Ayello, J.; Dela Cruz, F.; Rosenblum, J.M.; Lessnick, S.L.; Cairo, M.S. Protein Phosphatase 1 Regulatory Subunit 1A in Ewing Sarcoma Tumorigenesis and Metastasis. *Oncogene* **2018**, *37*, 798–809, doi:10.1038/onc.2017.378.

8. Xia, Y.; Zhan, C.; Feng, M.; Leblanc, M.; Ke, E.; Yeddula, N.; Verma, I.M. Targeting CREB Pathway Suppresses Small Cell Lung Cancer. *Molecular Cancer Research* **2018**, *16*, 825–832, doi:10.1158/1541-7786.MCR-17-0576.

9. McSherry, E.A.; Brennan, K.; Hudson, L.; Hill, A.D.; Hopkins, A.M. Breast Cancer Cell Migration Is Regulated through Junctional Adhesion Molecule-A-Mediated Activation of Rap1 GTPase. *Breast Cancer Research* **2011**, *13*, R31, doi:10.1186/bcr2853.

10. Jakobsen, E.; Lange, S.C.; Andersen, J. V.; Desler, C.; Kihl, H.F.; Hohnholt, M.C.; Stridh, M.H.; Rasmussen, L.J.; Waagepetersen, H.S.; Bak, L.K. The Inhibitors of Soluble Adenylate Cyclase 2-OHE, KH7, and Bithionol Compromise Mitochondrial ATP Production by Distinct Mechanisms. *Biochem Pharmacol* **2018**, *155*, 92–101, doi:10.1016/j.bcp.2018.06.023.

11. Coles, G.L.; Cristea, S.; Webber, J.T.; Levin, R.S.; Moss, S.M.; He, A.; Sangodkar, J.; Hwang, Y.C.; Arand, J.; Drainas, A.P.; et al. Unbiased Proteomic Profiling Uncovers a Targetable GNAS/PKA/PP2A Axis in Small Cell Lung Cancer Stem Cells. *Cancer Cell* **2020**, *38*, 129-143.e7, doi:10.1016/j.ccell.2020.05.003.

12. He, B.; Gao, R.; Lv, S.; Chen, A.; Huang, J.; Wang, L.; Feng, Y.; Feng, J.; Liu, B.; Lei, J.; et al. Cancer Cell Employs a Microenvironmental Neural Signal Trans-Activating Nucleus-Mitochondria

Coordination to Acquire Stemness. *Signal Transduct Target Ther* **2023**, *8*, 275, doi:10.1038/s41392-023-01487-4.

13. Kim, T.-H.; Gill, N.K.; Nyberg, K.D.; Nguyen, A. V.; Hohlbauch, S. V.; Geisse, N.A.; Nowell, C.J.; Sloan, E.K.; Rowat, A.C. Cancer Cells Become Less Deformable and More Invasive with Activation of β-Adrenergic Signaling. *J Cell Sci* **2016**, doi:10.1242/jcs.194803.

14. Daniele, S.; Zappelli, E.; Natali, L.; Martini, C.; Trincavelli, M.L. Modulation of A1 and A2B Adenosine Receptor Activity: A New Strategy to Sensitise Glioblastoma Stem Cells to Chemotherapy. *Cell Death Dis* **2014**, *5*, e1539–e1539, doi:10.1038/cddis.2014.487.

15. Liu, G.; Yang, S.; Liu, Y.; Xu, Y.; Qiu, H.; Sun, J.; Song, J.; Shi, L. The Adenosine-A2a Receptor Regulates the Radioresistance of Gastric Cancer via PI3K-AKT-MTOR Pathway. *Int J Clin Oncol* **2022**, *27*, 911–920, doi:10.1007/s10147-022-02123-x.

16. Misra, U.K.; Pizzo, S.V. Epac1-induced Cellular Proliferation in Prostate Cancer Cells Is Mediated by B-Raf/ERK and MTOR Signaling Cascades. *J Cell Biochem* **2009**, *108*, 998–1011, doi:10.1002/jcb.22333.

17. Menon, J.; Doebele, R.C.; Gomes, S.; Bevilacqua, E.; Reindl, K.M.; Rosner, M.R. A Novel Interplay between Rap1 and PKA Regulates Induction of Angiogenesis in Prostate Cancer. *PLoS One* **2012**, *7*, e49893, doi:10.1371/journal.pone.0049893.

18. Misra, U.K.; Pizzo, S.V. Evidence for a Pro-Proliferative Feedback Loop in Prostate Cancer: The Role of Epac1 and COX-2-Dependent Pathways. *PLoS One* **2013**, *8*, e63150, doi:10.1371/journal.pone.0063150.

19. Faam, B.; Ghadiri, Ata.A.; Ghaffari, M.A.; Totonchi, M.; Amouzegar, A.; Azizi, F.; Shahbazian, H.; Hashemitabar, M.; Fanaei, S.A.; Khorsandi, L. CpG Island Methylation of the Rap1Gap Gene in Medullary Thyroid Cancer. *Arch Iran Med* **2022**, *25*, 171–177, doi:10.34172/aim.2022.29.

20. Dehghanian, M.; Yarahmadi, G.; Sandoghsaz, R.; Khodadadian, A.; Shamsi, F.; Vahidi Mehrjardi, M. Evaluation of Rap1GAP and EPAC1 Gene Expression in Endometriosis Disease. *Adv Biomed Res* **2023**, *12*, 101, doi:10.4103/abr.abr_86_22.

21. Zheng, H.; Gao, L.; Feng, Y.; Yuan, L.; Zhao, H.; Cornelius, L.A. Down-Regulation of Rap1GAP via Promoter Hypermethylation Promotes Melanoma Cell Proliferation, Survival, and Migration. *Cancer Res* **2009**, *69*, 449–457, doi:10.1158/0008-5472.CAN-08-2399.

22. Tamate, M.; Tanaka, R.; Osogami, H.; Matsuura, M.; Satohisa, S.; Iwasaki, M.; Saito, T. Rap1GAP Inhibits Tumor Progression in Endometrial Cancer. *Biochem Biophys Res Commun* **2017**, *485*, 476–483, doi:10.1016/j.bbrc.2017.02.044.

23. Zhang, Z.; Mitra, R.S.; Henson, B.S.; Datta, N.S.; McCauley, L.K.; Kumar, P.; Lee, J.S.-J.; Carey, T.E.; D'Silva, N.J. Rap1GAP Inhibits Tumor Growth in Oropharyngeal Squamous Cell Carcinoma. *Am J Pathol* **2006**, *168*, 585–596, doi:10.2353/ajpath.2006.050132.
